# Multiomics analysis of soybean meal induced marine fish enteritis in juvenile pearl gentian grouper, *Epinephelus fuscoguttatus ♀* × *Epinephelus lanceolatus ♂*

**DOI:** 10.1038/s41598-021-02278-z

**Published:** 2021-12-02

**Authors:** Wei Zhang, Beiping Tan, Junming Deng, Zhang Haitao

**Affiliations:** 1 Laboratory of Aquatic Animal Nutrition and Feed, College of Fisheries, Guangdong Ocean University, Zhanjiang, 524025 Guangdong People’s Republic of China; 2 Aquatic Animals Precision Nutrition and High Efficiency Feed Engineering Research Center of Guangdong Province, Zhanjiang, 524025 Guangdong People’s Republic of China; 3 Key Laboratory of Aquatic, Livestock and Poultry Feed Science and Technology in South China, Ministry of Agriculture, Zhanjiang, 524025 Guangdong People’s Republic of China; 4 Laboratory of Aquatic Animal Nutrition and Feed, College of Fisheries, Guangdong Ocean University, Zhanjiang, 524088 Guangdong People’s Republic of China

**Keywords:** Gastroenterology, Gastrointestinal diseases, Gastroenteritis, Inflammatory bowel disease, Intestinal diseases

## Abstract

As an important protein source, soybean products can cause intestinal inflammation and injury in many animals including human beings, particularly infants and juvenile individuals. Research in this field has been performed for terrestrial animals and fish, but still lacks integrity and systematicness. In this study, the main biological processes in the intestinal tract of marine fish juvenile pearl gentian grouper in the state of soybean meal-induced enteritis (SBMIE) were analyzed. A total of 720 groupers with an approximate initial weight of 12.5 g were randomly divided into three groups: the fish meal (FM) control group, the 20% SBM group (SBM20), and the SBM40 group (n = 4). Three iso-nitrogenous and iso-lipidic diets were prepared and fed to fish for 10 weeks. Each barrel contained a water volume of about 1 m^3^ in and was exposed to natural light and temperature. Results indicated that the growth and physiology of groupers fed with SBM were significantly negatively affected, with the gene expressions of intestinal structural protein abnormal. 16SrDNA high-throughput sequencing showed that the intestinal microflora played an important role in the pathogenesis of pearl gentian grouper SBMIE, which may activate a variety of pathogen pattern recognition receptors, such as toll-like receptors (TLRs), RIG-I-like receptors, and nod-like receptors. Transcriptome analysis revealed that changes of the SBMIE signaling pathway in pearl gentian groupers were conservative to some extent than that of terrestrial animals and freshwater fish. Moreover, the TLRs-nuclear factor kappa-B signaling pathway becomes activated, which played an important role in SBMIE. Meanwhile, the signal pathways related to nutrient absorption and metabolism were generally inhibited. Metabolomics analysis showed that isoflavones and saponins accounted for a large proportion in the potential biomarkers of pearl gentian grouper SBMIE, and most of the biomarkers had significantly positive or negative correlations with each other; 56 metabolites were exchanged between intestinal tissues and contents, which may play an important role in the development of enteritis, including unsaturated fatty acids, organic acids, amino acids, vitamins, small peptides, and nucleotides, etc. These results provide a basic theoretical reference for solving the intestinal issues of fish SBMIE and research of inflammatory bowel disease in mammals.

## Introduction

Since the beginning of 1990, fishery production has stagnated at about 90 million tons, and aquaculture is expected to meet the global demand for marine food in the future^1^. With the continuous expansion of aquaculture, the demand for fish meals is increasing day by day. However, the rise in fish meal (FM) prices and the decline in production have forced to search for new fish meal substitutes^2^.

Alternative protein sources must be economically viable, environmentally friendly, healthy for fish, and at least maintain the current growth rate of aquaculture. At present, soybean products are the first choice to replace FM protein, among which soybean meal (SBM) is the most commonly used^3^. However, common SBMs can only be added to feed at a low level, otherwise, it may affect the intestinal microflora structure and cause enteritis in the hindgut of fish, such as *Salmo salar*^4^, *Oncorhynchus mykiss*^5^, *Cyprinus carpio*^6^, and *Danio rerio*^7^, etc.

SBM-induced enteritis (SBMIE) is now widely used as a model for the study of intestinal inflammation in fish^8^. SBMIE in fish is known as non-infectious subacute enteritis and has the histological features of reduced mucosal fold height, swollen lamina propria, and sub-mucosa, infiltration of various inflammatory cells, and decreased absorption vacuoles of intestinal epithelial cells^3^. Enteritis mainly occurs in the distal intestine—the main part of protein absorption by endocytosis—and is, therefore, more sensitive to intestinal diseases caused by food infection^9^.

It is generally believed that the effect of SBM on fish intestinal health is related to anti-nutrition factors (ANFs), such as protease inhibitors, phytate, saponins, lectin, phytosterol, and oligosaccharides, *etc*^10^. The potential effects of these ANFs on fish metabolism include increased intestinal permeability, disruption of lipid and protein digestion, changes in cholesterol, bile salts digestion and metabolism, *etc*^8,10^. In addition, the unknown antigen components in SBM may also induce an immune response or change the composition and/or abundance of intestinal microflora. For example, SBM may increase the harmful microflora abundance in the intestines of *Salmo salar*^11^, *Oncorhynchus mykiss*^12^, and *Gadus morhua*^13^, and cause inflammation.

The exact reasons for SBMIE and other negative effects are not fully understood, but related studies have indicated alcohol-soluble ANFs, especially soybean saponins, to be the main potential pathogenic factor^14^. In vitro experiments have shown soybean saponins to enhance the ability of epithelial cells to absorb macromolecules by increasing cell permeability^15^. The increase of permeability can be produced by weakening the tight junction structure or destroying the epithelial monolayer. However, the direct effect of soyasaponins is complex, with no or negligible inflammation observed when soybean saponins were fed alone or in combination with corn gluten, sunflower, rapeseed, or broad bean protein. Soybean saponins combined with pea protein, on the other hand, can cause significant changes in histological and transcriptional levels^16^. Other studies have shown that the enteritis saponins induced enteritis is dose-dependent, where a high content of saponins (2-4 g/kg) can cause enteritis of Atlantic salmon that has nothing to do with the type of protein source in the basal diet^14^. In addition, low levels of saponins seem to promote the growth of some fish. This may be related to the immune system promoting effect of saponin using as an adjuvant ^17^. The content of ANFs has also been shown to be affected by legume varieties and protein extraction methods^18^. The results suggested that the interaction between different ANFs is very important, but the mechanism of the effects of different ANFs on intestinal health is not fully understood.

In mammalian studies, saponins have been found to bind to the membrane cholesterol of intestinal epithelial cells to form pores and change membrane permeability. This may contribute to the absorption of molecules that are usually not absorbed by intestinal cells, including antigens and potential toxins. Therefore, the SBMIE may also be the minor role of soybean saponin on membrane interference characteristics, and the foreign antigen causing enteritis may be the antigenic soy protein or the antigen from intestinal microflora^16,19^. Fish intestinal microorganisms play an important role in regulating nutrient digestion, immune response, intestinal differentiation, disease resistance, and colonization of potential pathogens^20^. Therefore, healthy intestinal microflora is essential for the health and growth of fish. There is no doubt that diet is an important factor affecting intestinal microflora^21^. However, in fish, the overall understanding of the correlation between gut microflora, feed, and intestinal health remains unclear^22^. Through high-throughput sequencing technology, it has been proved that some human health diseases, such as inflammatory bowel diseases (IBD), including Crohn’s disease (CD) and ulcerative colitis (UC), are closely related to intestinal microbial imbalance^23^. A similar phenomenon has been observed in mice^24^. Whilst previous studies have found SBM to affect the intestinal microflora of fish, only a few report the relationship between intestinal microflora and immune function^25^.

The effect of SBM on the intestinal health of fish is complex. Up to now, it is not clear what is the specific cause of SBMIE in fish and there is a lack of systematic and comprehensive explanation. Researchers believe that it may be the result of the interaction of ANFs, antigens, and intestinal microflora^3^. In mammal studies, the eitology of IBD is complex, including host, intestinal microflora, and environmental factors. New high throughput omics technologies such as genomics, metagenomics, transcriptomics, metatranscriptomics, proteomics, metaproteomics, and metabolomics, support us to investigate the different aspects of these factors contributing to IBD pathogenesis, with relatively few multi-omics research incorporating multiple data types from the same subjects^26^. Currently, the interaction between nutrition and the immune system has been widely recognized, but the basic and applied research on the interaction between fish diet and health lags far behind that of mammals^27^.

The hybrid pearl gentian grouper (*Epinephelus fuscoguttatus* ♀ × *Epinephelus lanceolatus* ♂), a carnivorous marine fish, is widely distributed in the Indo-Pacific region and over the world due to its advantages of rapid growth rate, high market value, and good disease resistance^28,29^. The carnivorous fish are believed to be less adapted to diets involving plant ingredients. Previous studies indicated that SBM with standard quality can only be added in fish diets at a relatively low level; otherwise, it will affect the intestinal physiological structure and cause enteriti^20^. A previous study in our lab found that excessive levels of SBM induce pearl gentian grouper enteritis.

To fully understand the characterization of SBMIE in fish, the response mechanism of SBMIE in pearl gentian grouper was investigated in this study via the multi-omics method, including 16S high-throughput sequencing, “3 + 2” full-length transcriptome and metabolome techniques. These results provide the basic theoretical reference for solving the intestinal issues of fish SBMIE and the research of inflammatory bowel disease in mammals.

## Results

### Growth performance

Table 1 shows that the final body weight (FBW), WGR, and SGR significantly decreased in the experimental groups compared with the FM group with dietary SBM increase (*P* < 0.05), and FCR significantly increased in experimental groups (*P* < 0.05). HSI and SR were not significantly affected by dietary SBM (*P* > 0.05).

**Table 1:** Effect of different levels of soybean meal protein substitute for fish meal protein on the growth of pearl gentian grouper (n = 3).

Parameters	FM	SBM20	SBM40
IBW(g)	12.55 ± 0.00	12.55 ± 0.01	12.55 ± 0.04
FBW(g)	73.82 ± 0.52^a^	70.80 ± 1.27^b^	66.08 ± 0.57^c^
WGR(%)	485.14 ± 7.08^a^	464.36 ± 10.12^b^	426.50 ± 9.59^c^
SGR(%d)	2.60 ± 0.02^a^	2.54 ± 0.03^b^	2.44 ± 0.03^c^
FCR	0.84 ± 0.01^a^	0.87 ± 0.02^b^	0.95 ± 0.02^c^
HSI(%)	2.43 ± 0.45	2.21 ± 0.36	2.18 ± 0.25
SR(%)	99.17 ± 0.96	99.58 ± 0.84	99.17 ± 0.96

Results are presented as the mean ± SD. Values in a row with different superscripts represent significantly different at *P* < 0.05. IBW is the initial body weight, FBW is the final body weight, WGR is the weight gain rate, SGR is the special growth rate, FCR is the feed conversion rate, HSI is the hepatosomatic index and SR is the survival rate.

### Histological analysis

**Figure 1:**
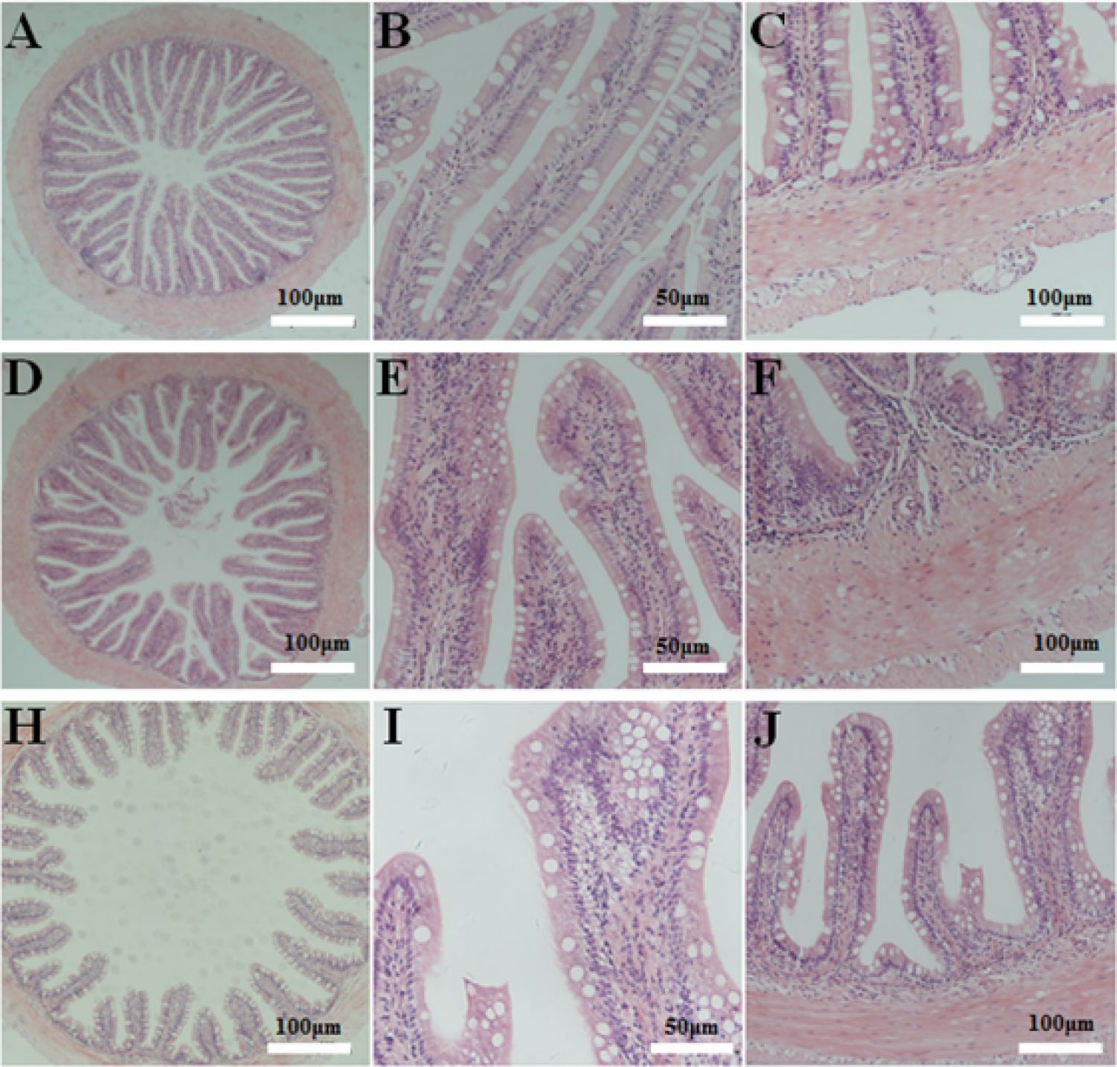
Hematoxylin–eosin staining in the DI tissues of pearl gentian grouper. Note: Representative histomorphological images from hematoxylin and eosin-stained sections of the distal intestine of grouper juvenile depicting the inflammatory changes in grouper on FM (**A**, **B**, **C**), SBM20 (**D**, **E**, **F**) and SBM40 (**H**, **I**, **J**) diets. (**A**, **D**, **H**) Representative images of decreased height and increased fusion of the mucosal folds in SBM20 and SBM40 groups. (**B**, **E**, **I**) Representative images of increased width and cellular (leucocyte) infiltration (asterisk) of the lamina propria (arrows) in SBM20 and SBM40 groups. (**C**, **F**, **J**) Representative images of increased width and cellular (leucocyte) infiltration of the submucosa. FM refers to the fish meal control group, SBM20 refers to the 20% SBM protein replacement level to FM protein, and SBM40 refers to the 40% SBM protein replacement level to FM protein.

**Figure 2:**
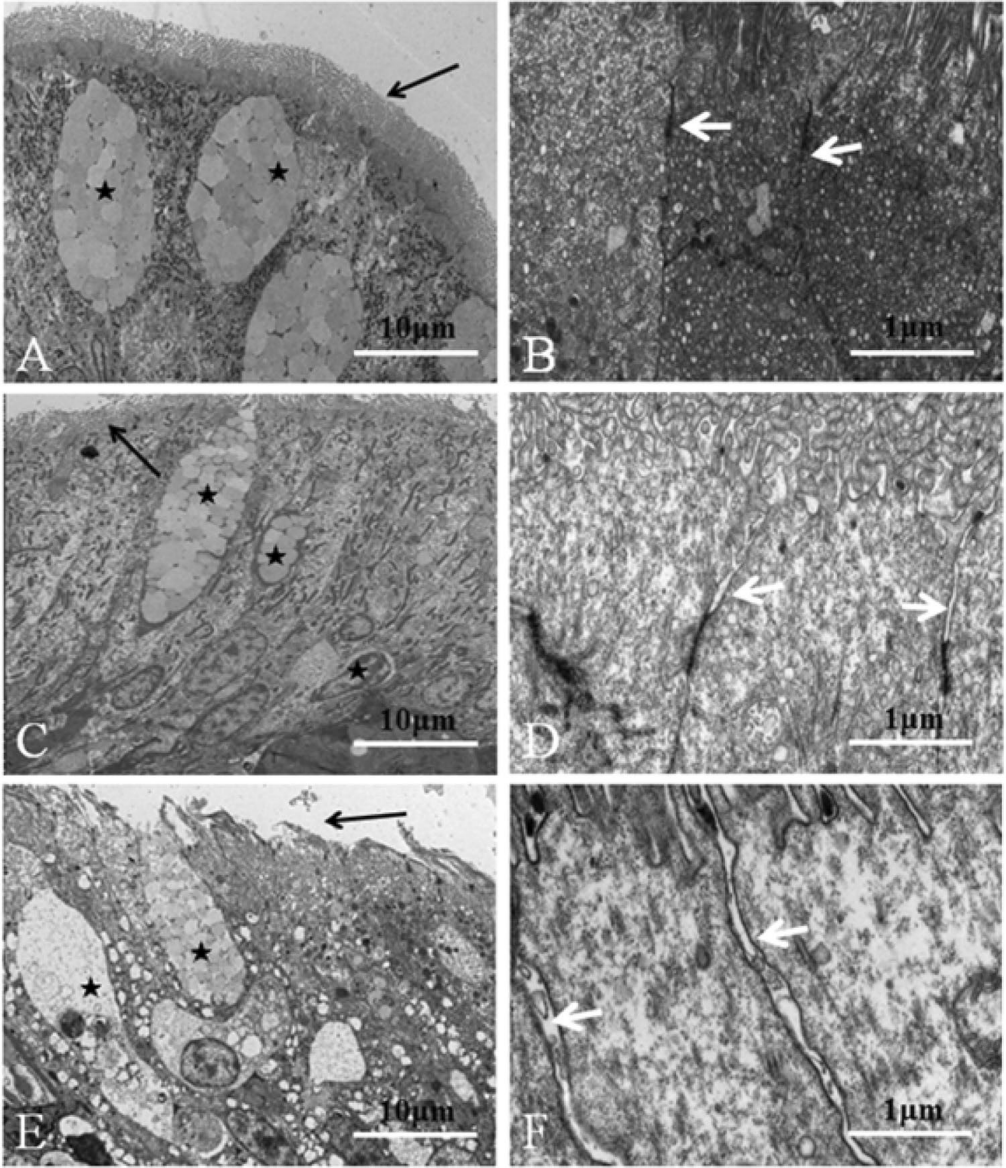
Electron microscopic structures of epithelial cells in the DI tissues of grouper. Note: (**A**), (**B**)-FM control group, (**C**, **D**)-SBM20 group, (**E**, **F**)-SBM40 group. Asterisks represent goblet cells. Black arrows represent the microvilli of intestinal epithelial cells. White arrows represent intercellular junctions between the epithelium cells.

Semi-quantitative analysis of HE staining showed that SBM with different substitution levels induces inflammation in the DI tissue of pearl gentian grouper, in significant contrast to the FM control group (*P* < 0.05). Among them, inflammation in the SBM40 group was very serious, while the severity order of inflammation in the SBM20 group was mild with obvious inflammatory characteristics (Fig. 1 and Table 2). The TEM data shows that the goblet-cell number gradually increases with the increase of SBM substitute level (Fig. 2A,C,E), and the intercellular junctions in experimental groups become progressively worse in comparison to the FM group (Fig. 2B,D,F).

**Table 2:** Semi-quantitative histological evaluation of intestinal sections (n = 10).

Parameters	FM	SBM20	SBM40
Mucosal folds	1.27 ± 0.12^a^	3.20 ± 0.10^b^	4.87 ± 0.15^c^
Lamina propria	1.47 ± 0.35^a^	3.17 ± 0.25^b^	5.00 ± 0.25^c^
Supranuclear vacuoles	1.27 ± 0.21^a^	3.10 ± 0.26^b^	4.90 ± 0.10^c^
Connective tissue	1.47 ± 0.25^a^	3.07 ± 0.15^b^	4.93 ± 0.21^c^

### Determination of enzyme activity

Table 3 shows that, with the increase of SBM substitute level, the enzyme activities of Trypsin and T-SOD in intestinal tissues gradually increased with significant differences among the groups (*P* < 0.05). The concentration of MDA also presents the same significant change trend (*P* < 0.05). On the contrary, the concentrations of C3, C4, and IgM decreased significantly among the groups (*P* < 0.05). In the liver, the activities of ALT and AST increased significantly with the increase of SBM substitution level (*P* < 0.05). Similarly, the activity of LYS in serum increased significantly with increases in the SBM substitution level (*P* < 0.05).

**Table 3:** Effect of soybean meal on the enzyme activities of pearl gentian grouper (n = 3).

Parameters	FM	SBM20	SBM40
Trypsin in intestine (U/mg)	597.33 ± 75.53^a^	828.25 ± 113.58^b^	1050.00 ± 104.06^c^
IgM in intestine (μg/mg)	94.33 ± 4.22^a^	79.47 ± 4.36^b^	64.70 ± 3.63^c^
C3 in intestine (μg/mg)	85.58 ± 5.31^a^	73.31 ± 6.36^b^	61.70 ± 8.21^c^
C4 in intestine (μg/mg)	128.83 ± 10.17^a^	113.97 ± 11.04^b^	92.88 ± 5.62^c^
MDA in intestine (nmol/mg)	2.49 ± 0.41^a^	3.60 ± 0.35^b^	4.25 ± 0.33^c^
ALT in liver (U/g)	25.53 ± 3.34^a^	30.97 ± 3.84^ab^	36.64 ± 4.13^b^
AST in liver (U/g)	26.88 ± 4.02^a^	32.94 ± 4.25^b^	38.94 ± 4.34^b^
LYS in serum (U/g)	5.45 ± 0.47^a^	7.06 ± 1.02^b^	7.95 ± 0.64^b^

### Expression of immune-related genes

Table 4 shows that the expression of pro-inflammatory genes such as *IL1β*, *IL8*, *IL17*, *TNF*α and *CSF1* significantly increased (*P* < 0.05), with the highest value appearing in the SBM40 group.

**Table 4:** Effect of SBM protein substitute for fish meal protein on the pro-inflammatory-related gene expression in DI tissues of pearl gentian grouper (n = 3).

Gene	FM	SBM20	SBM40
*IL1β*	1.16 ± 0.16^a^	1.18 ± 0.25^a^	1.85 ± 0.12^b^
*IL8*	1.00 ± 0.08^a^	1.66 ± 0.14^b^	2.35 ± 0.17^c^
*IL17*	1.00 ± 0.07^a^	1.28 ± 0.22^b^	1.60 ± 0.04^c^
*TNFα*	1.01 ± 0.15^a^	1.04 ± 0.16^a^	1.52 ± 0.23^b^
*CSF1*	1.01 ± 0.13^a^	0.98 ± 0.07^a^	5.33 ± 0.73^b^

Table 5 shows that the expression of anti-inflammatory genes such as *IL4*, *IL10* and *TGFβ1* significantly decreased (*P* < 0.05), with the minimum value appearing in the SBM40 group. The expression of antimicrobial peptide *Hepcidin* significantly increased in the SBM40 group (*P* < 0.05).

**Table 5:** Effect of SBM protein substitute for fish meal protein on the anti-inflammatory-related gene expression in DI tissues of pearl gentian grouper (n = 3).

Gene	FM	SBM20	SBM40
*IL4*	1.01 ± 0.15^a^	0.85 ± 0.21^a^	0.56 ± 0.12^b^
*IL10*	1.03 ± 0.06^a^	0.84 ± 0.11^a^	0.36 ± 0.09^b^
*TGFβ1*	1.00 ± 0.08^a^	1.21 ± 0.23^a^	0.46 ± 0.16^b^
*Hepcidin*	1.02 ± 0.25^a^	0.87 ± 0.21^a^	1.33 ± 0.19^b^

### 16S high-throughput sequencing

#### Sequencing information

**Figure 3:**
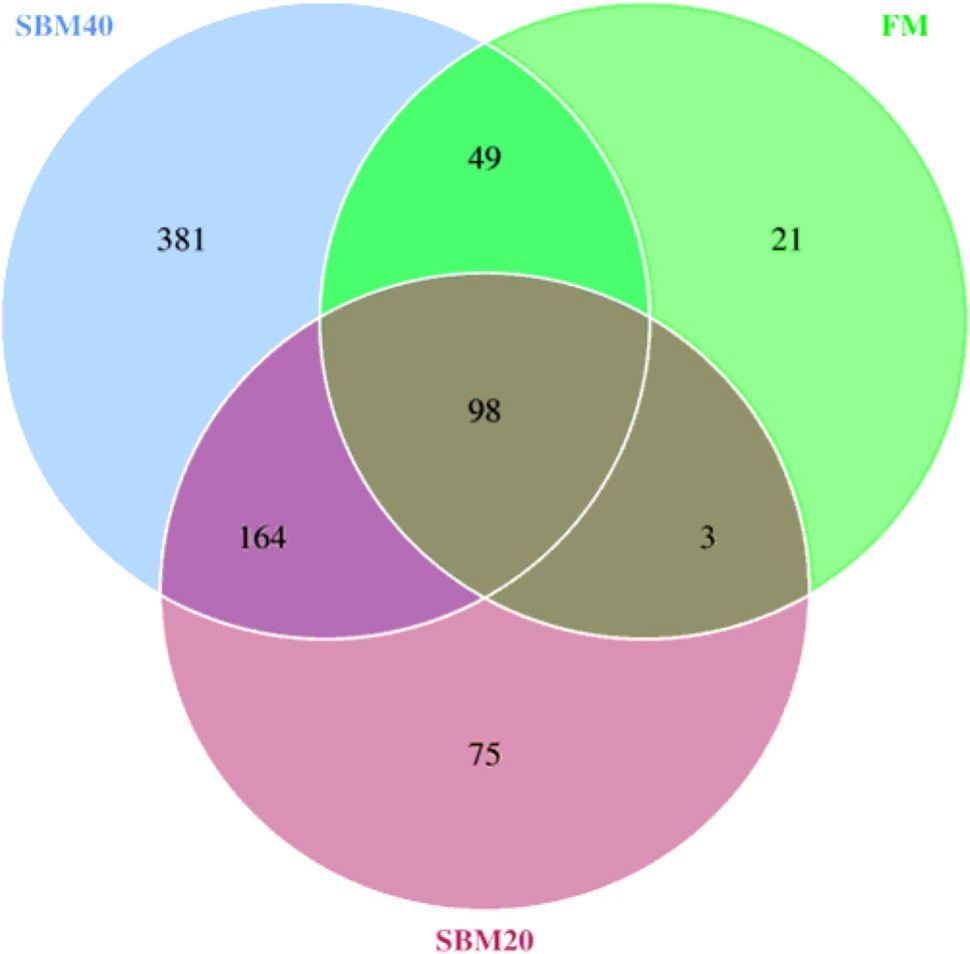
Venn diagram of OUT by high-throughput sequencing after replacing the fish meal with soybean meal in gentian grouper (n = 3).

In total, 735,636 raw reads were obtained from the nine samples. The average number of sequences in the FM, SBM20, and SBM40 groups were 79,339.67 ± 4821.81, 82,021.33 ± 13,477.76, and 83,851.00 ± 10,629.48, respectively. After clustering with 97% consistency, a total of 835 OTU were obtained. The average number of OTU in the FM, SBM20, SBM40 groups were 175.00 ± 32.79, 355.00 ± 64.21, and 608.67 ± 56.84, respectively. A significant difference in the OUT number between the FM and SBM40 groups is observed (*P* < 0.05). The Venn diagram shown in Fig. 3 shows there are 98 OTU co-contained among the three groups.

#### OTU diversity analysis

After OTU homogenization, *α* and *β* diversity of the data were analyzed. α diversity analysis shows that the Good's coverage of all samples exceeded 99%, indicative that most of the bacteria found in this study were OTU. The *α* diversity index included the Observed species, Shannon, Simpson, Chao1, ACE and Good's coverage indexes. Whilst Good's coverage showed no significant differences among the groups (*P* > 0.05), other indexes showed a significant increasing trend with the increase of SBM substitute level (*P* < 0.05) (Table 6).

**Table 6:** Statistical analysis of alpha diversity of intestinal microflora (n = 3).

Parameters	FM	SBM20	SBM40
Observed species	92.33 ± 7.51^a^	144.67 ± 10.60^b^	909.33 ± 13.65^c^
Shannon	2.30 ± 0.08^a^	2.78 ± 0.29^a^	6.27 ± 0.98^b^
Simpson	0.66 ± 0.03^a^	0.73 ± 0.43^b^	0.95 ± 0.22^c^
Chao1	121.10 ± 11.79^a^	186.35 ± 16.89^b^	985.40 ± 11.09^c^
ACE	153.55 ± 6.71^a^	207.67 ± 16.75^a^	966.11 ± 42.41^b^
Good’s coverage	0.99 ± 0.00	0.99 ± 0.00	0.99 ± 0.00

**Figure 4:**
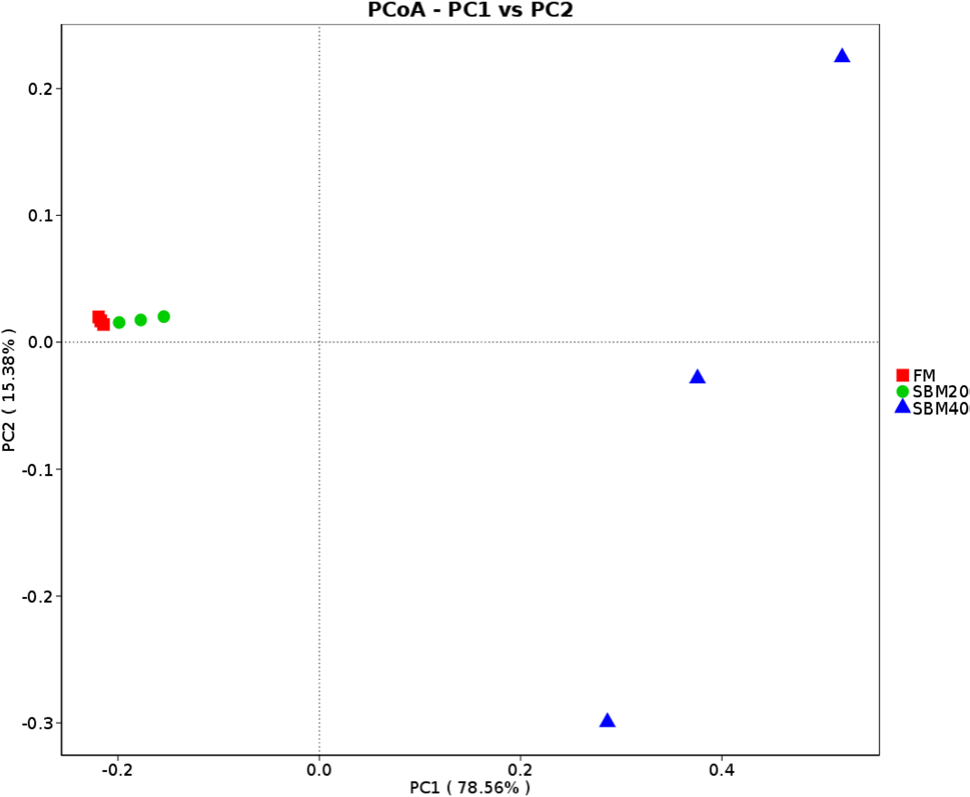
PCoA analysis of intestinal microflora of pearl gentian grouper fed by different levels of soybean meal (n = 3).

**Figure 5:**
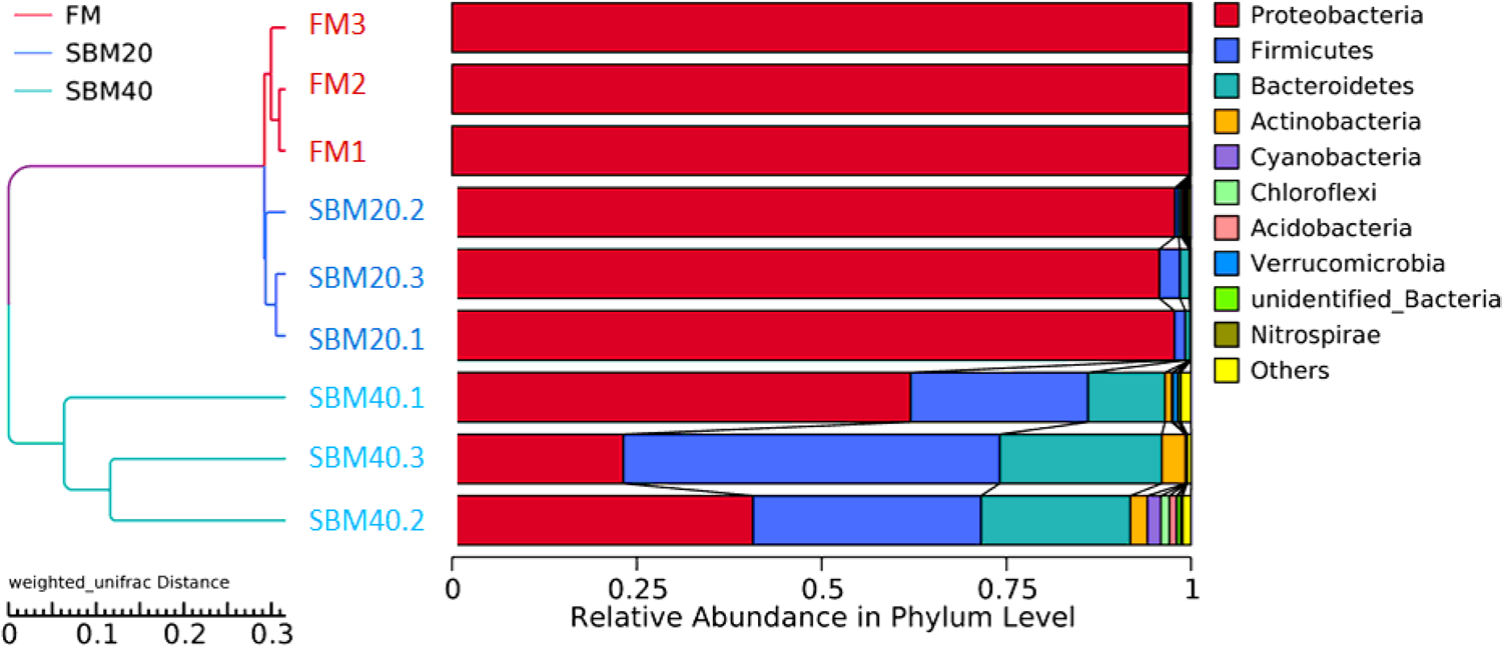
UPMGA clustering analysis of all samples based on the Weighted-Unifrac distance matrix at phylum level (n = 3).

In order to evaluate the overall differences of *β*-diversity among groups, the differences of *β*-diversity indexes among groups and PCoA were used for analysis based on a Weighted Unifrac distance matrix. Differences of *β*-diversity indexes among groups analysis showed little differences between the FM and SBM20 groups (Wilcox, *P* > 0.05) but significant differences among all the groups (Wilcox, *P* < 0.05) (Supplementary Table 1 and Supplementary Fig. 1). PCoA analysis showed that the distance between the FM and SBM20 groups was close and that the SBM40 group could be distinguished from both the FM and SBM20 groups (Fig. 4), as is also reflected in the UPMGA clustering analysis of all samples based on the Weighted-Unifrac distance matrix (Fig. 5).

#### Comparison of composition and abundance of microflora

**Figure 6:**
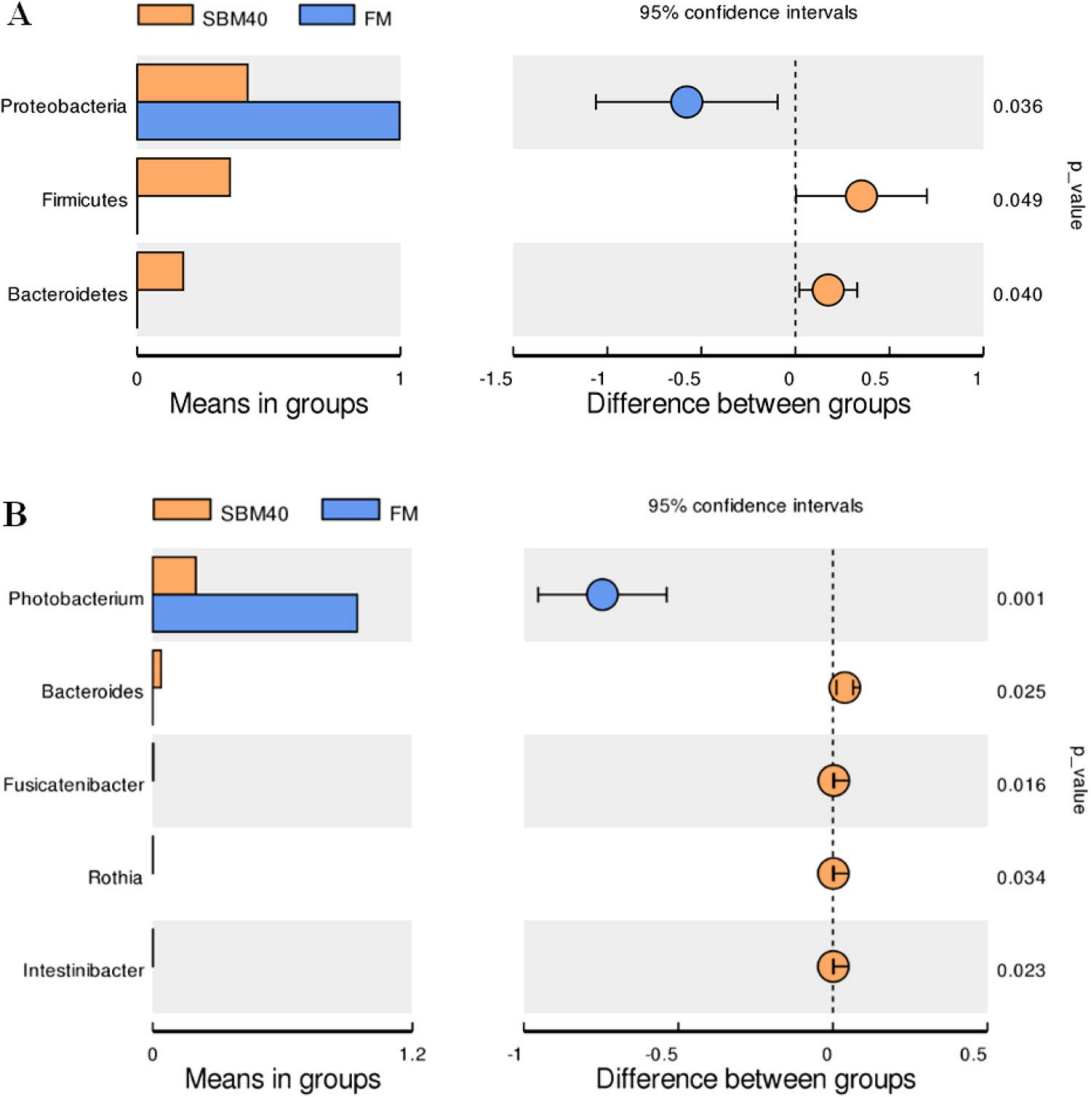
Differential analysis of intestinal microflora abundance of pearl gentian grouper fed by FM and SBM40 diets by T-test. A, phylum level; B, genus level (n = 3).

At the phylum level, the top 10 species of relative abundance were *Proteobacteria*, *Firmicutes*, *Bacteroidetes*, *Actinobacteria* and *Cyanobacteria*, etc*.* (Fig. 5). With the increase of SBM substitute level, the abundance of Proteobacteria significantly decreased in the SBM40 group (T-test, *P* < 0.05), and the abundance of Firmicutes and Bacteroidetes significantly increased in the SBM40 group (T-test, *P* < 0.05) (Fig. 6A and Supplementary Table 2).

At the genus level, the top 10 species of relative abundance were *Photobacterium*, *Faecalibacterium*, *Stenotrophomonas*, *Vibrio* and *Neisseria*, etc*.* (Supplementary Fig. 2). The abundance of *Photobacterium* significantly decreased in the SBM40 group (T-test, *P* < 0.05), while the abundance of *Bacteroides*, *Fusicatenibacter*, *Rothia*, *Intestinibacter*, *unidentified_Prevotellaceae*, *Halioglobus*, *Actinomyces*, *Anoxybacillus* and *Novosphingobium* significantly increased in the SBM40 group (T-test, *P* < 0.05) (Fig. 6B and Supplementary Table 3).

#### Functional prediction

**Figure 7:**
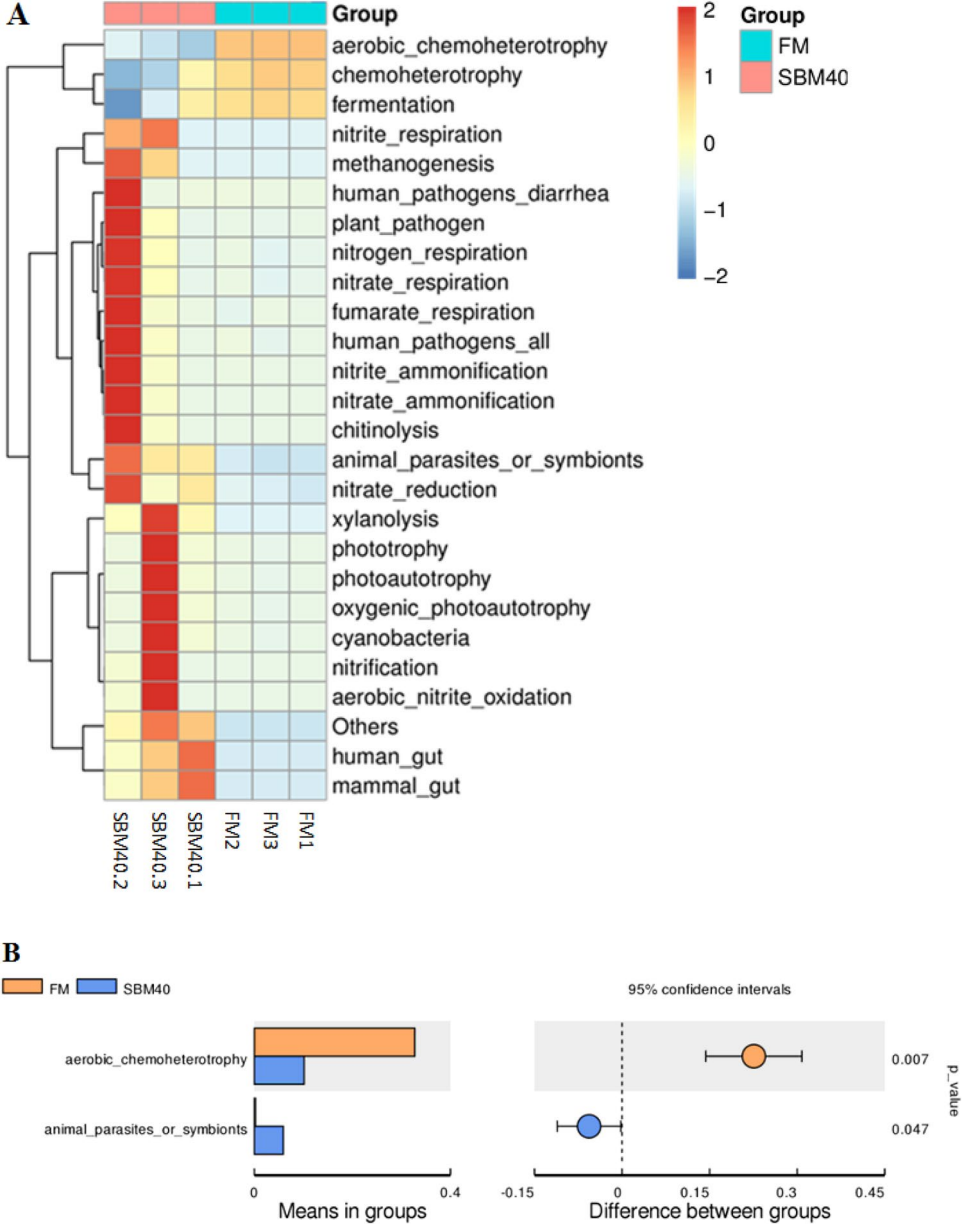
The intestinal microflora functional prediction of pearl gentian grouper fed by FM and SBM40 diets (n = 3). A, functional clustering heatmap by FAPROTAX; B, significance tests of functional difference analysis (n = 3).

Functional prediction analysis was performed by FAPROTAX. The clustering heatmap displayed the top 25 functional abundance of the intestinal microflora, including fermentation, chemoheterotrophy, aerobic_ chemoheterotrophy, human_gut, mammal_gut, fumarate_respiration, nitrification, chitinolysis, nitrate_ammonification, nitrite_respiration, etc. The top 3 functional abundance of intestinal microflora decreased in the SBM40 group, and the remaining 22 functional abundance in the SBM40 group were all higher than that in the FM control group (Fig. 7A). Among all predicted functionals, the aerobic_chemoheterotrophy abundance was significantly decreased in the SBM40 group (*P* < 0.05), while animal_parasites_or_symbiont abundance was significantly increased in the SBM40 group (*P* < 0.05) (Fig. 7B and Supplementary Table 4). The results show that the excessive addition of SBM causes the dysfunction of intestinal microflora and an increase of pathogenic bacteria. The compositions of intestinal microflora in the SBM40 group with significant changes in the functional abundance of aerobic_chemoheterotrophy and animal_parasites_or_symbiont is shown in Supplementary Table 5.

### Transcriptome analysis

#### Statistics of DEGs

**Figure 8:**
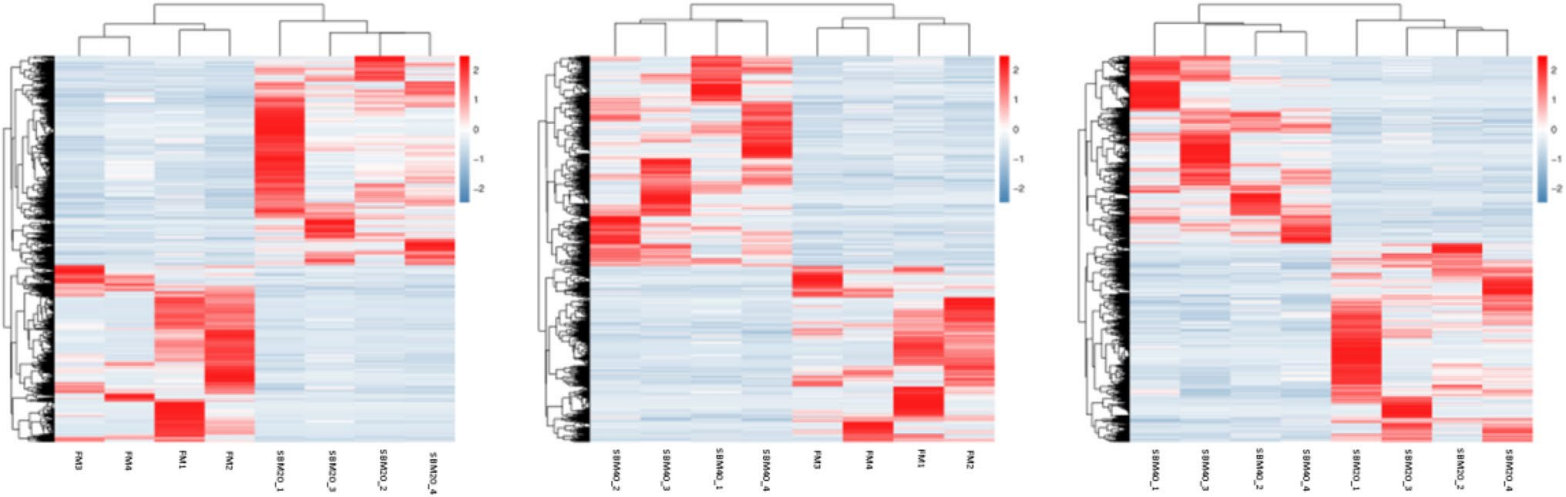
Cluster heat map of significantly DEGs in DI tissues of pearl gentian grouper fed by different substitution levels of SBM diet (n = 4).

The number of DEGs in each group are shown in Table 7. Compared to the FM control group, 3588 genes with significant differences in the SBM20 group were observed (*P* < 0.05), among which 1945 genes were up-regulated and 1643 genes were down-regulated. Similarly, compared to the FM control group, 2305 genes with significant differences in the SBM40 group were observed (*P* < 0.05), among which 1256 genes were up-regulated and 1049 genes were down-regulated. The clustering heatmap displays that the differential genes can cluster well according to the group in the SBM feeding trial, which indicates the sample processing is reasonable and data quality control is good (Fig. 8).

**Table 7:** Statistics of DEGs in DI tissues of pearl gentian grouper fed by different levels of SBM diets (n = 4).

Number	SBM20 *vs*. FM	SBM40 *vs*. FM
Up-regulated	1945	1256
Down-regulated	1643	1049
Total	3588	2305

#### Trend analysis of DEGs

**Figure 9:**
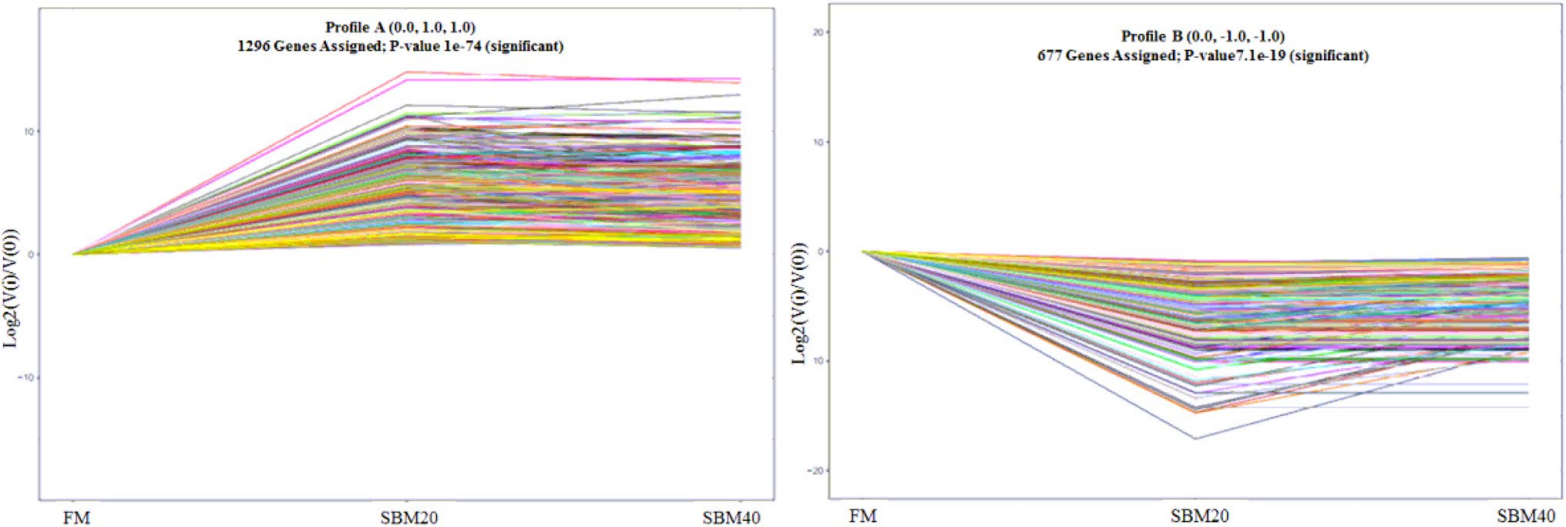
The transcriptome trend analysis of DEGs of (n = 4).

According to the trend analysis of DEGs, 1296 genes showed a significant upward trend (*P* < 0.05), which was recorded as profile A, while 677 genes showed a significant downward trend (*P* < 0.05), which was recorded as profile B (Fig. 9).

#### GO enrichment analysis of DEGs

**Figure 10:**
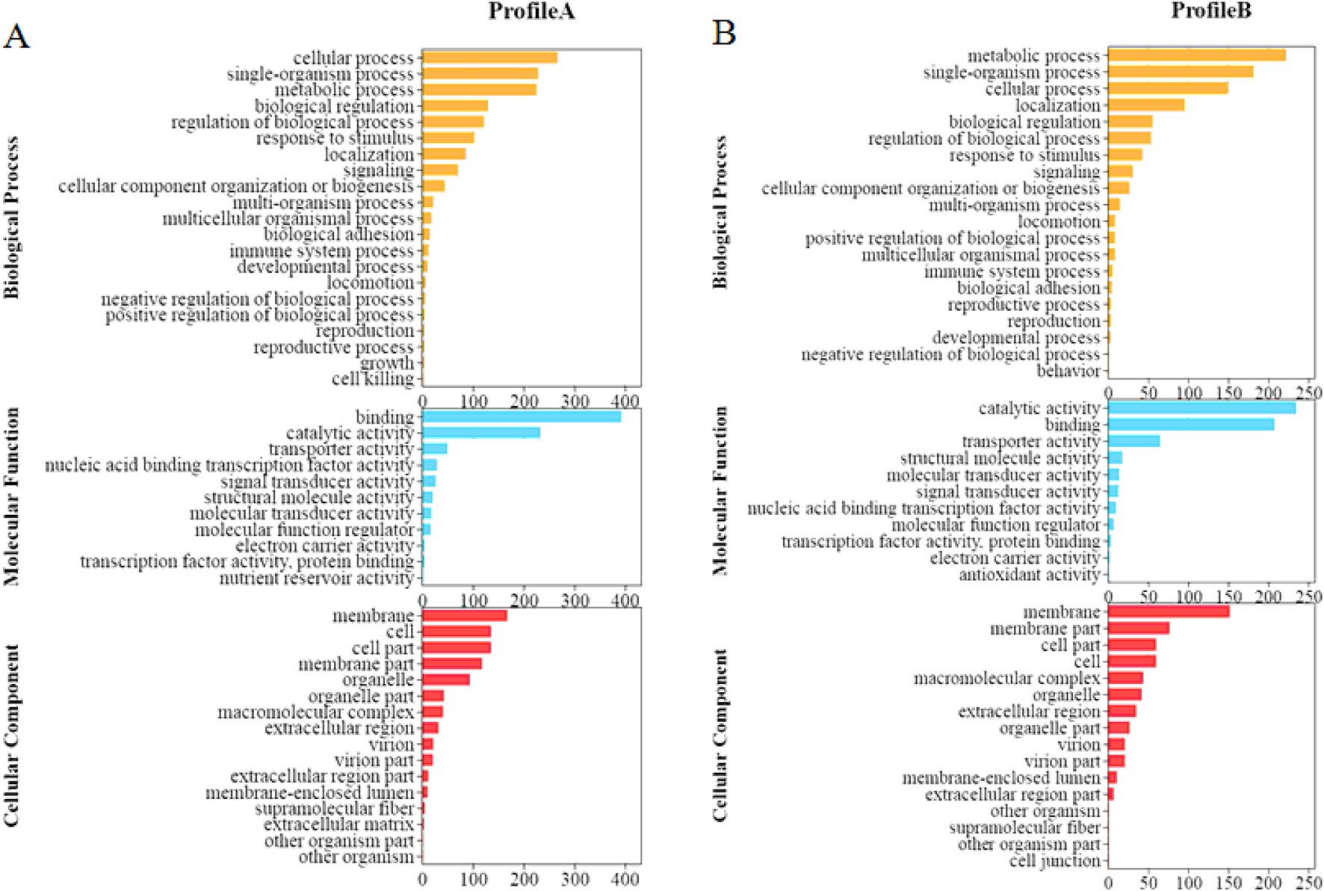
GO enrichment analysis of differential expressed genes.

GO enrichment analysis was carried out on profile A and profile B. Profile A enrichment results show the DEGs were enriched into three primary categories, namely, biological processes, molecular function, and cellular components, with a total of 48 sub-categories. In the sub-categories of biological processes, cellular process (267) was the most dominant group, followed by the single-organism (229), metabolic (226), and regulation of biological processes (122). Within the molecular function sub-categories, binding (393) and catalytic activity (233) were the dominant groups. As for the sub-categories of the cellular component, 168 DEGs were assigned to the membrane, followed by the cell (136), cell part (136), and membrane part (118) (Fig. 10A).

Profile B was enriched into 47 sub-categories of the three primary categories mentioned above. In the subcategories of biological processes, the metabolic process (222) was the most dominant group, followed by single-organism (182) and cellular processes (150). Within the molecular function sub-categories, catalytic activity (235) and binding (208) were the dominant groups. In the sub-categories of the cellular component, the membrane (152) was the most dominant group, followed by the membrane part (77), cell (60) and cell part (60) (Fig. 10B).

#### KEGG enrichment analysis of DEGs

**Figure 11:**
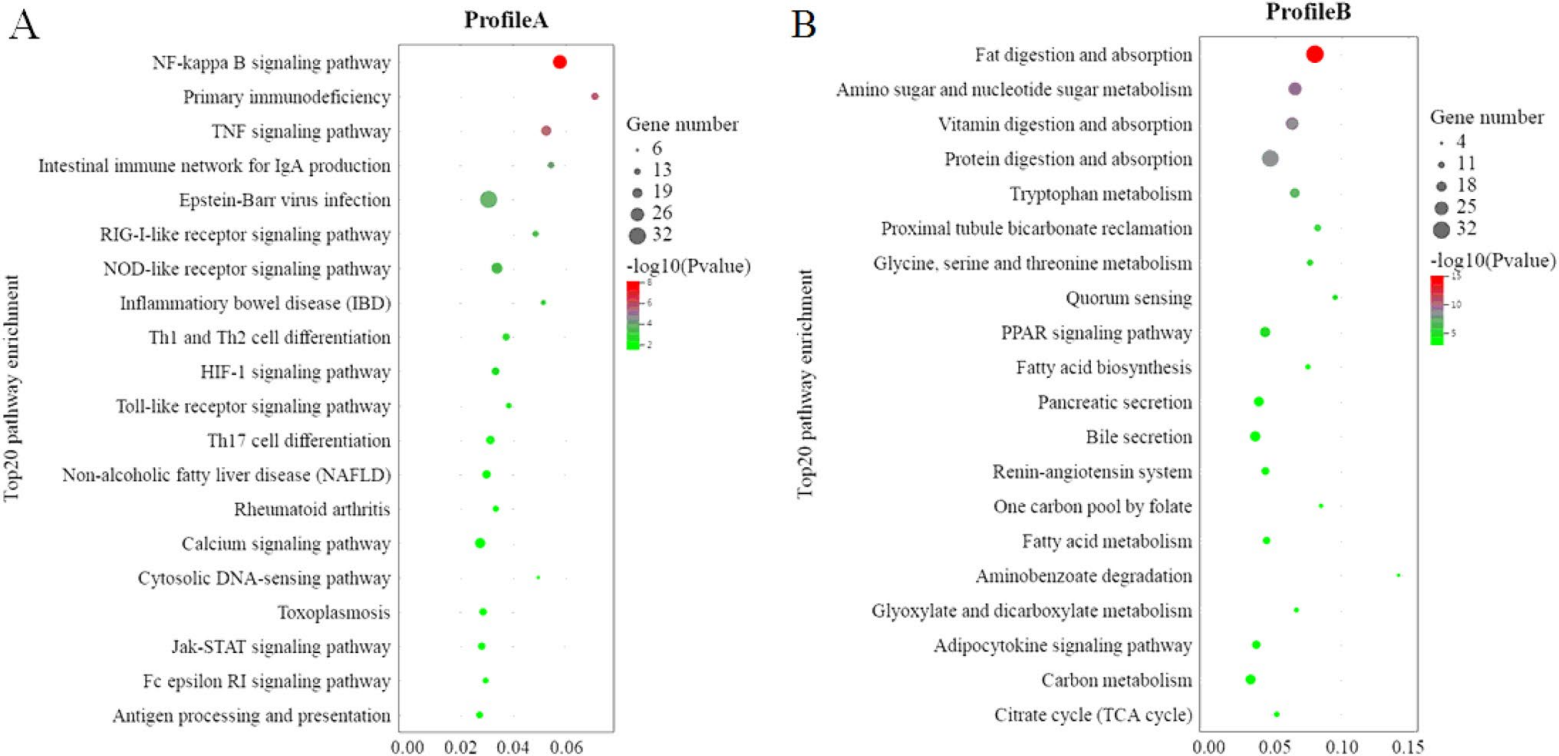
Significant KEGG enrichment pathways of DEGs obtained from the trend analysis. Note: KEGG database was cited from Kanehisa Laboratories^30–32^.

KEGG enrichment analysis was performed on Profile A and Profile B. Profile A enrichment results show that 266 pathways were enriched, 58 of which were significant (*P* < 0.05). Among all the pathways, 78 pathways were associated with immune diseases/system, infectious diseases, and signal transduction, in which 32 pathways were significantly enriched (*P* < 0.05). That is to say, 55.17% (32/58) of all the pathways significantly enriched were related to the immune diseases/system, infectious diseases, and signal transduction. These include the NF-kappaB (NF-κB) signaling pathway, TNF signaling pathway, Intestinal immune network for IgA production, NOD-like receptor signaling pathway, Inflammatory bowel disease (IBD), Th1 and Th2 cell differentiation, Toll-like receptor signaling pathway, Th17 cell differentiation, Jak-STAT signaling pathway, and Antigen processing and presentation. Most of these significantly enriched pathways are closely related to the development of immune responses such as inflammation (Fig. 11A and Supplementary Table 6).

Profile B enrichment results show that 270 pathways were enriched, 44 of which were significant (*P* < 0.05). Among all the pathways, 69 pathways were associated with immune diseases/system, infectious diseases, and signal transduction, in which 3 pathways were significantly enriched (*P* < 0.05) including the AMPK signaling pathway, two-component system, and African trypanosomiasis. That is, only 6.81% (3/44) of all the pathways significantly enriched were related to immune diseases/system, infectious diseases, and signal transduction. However, 65.91% (29/44) of the pathways significantly enriched were related to the digestive system, carbohydrate metabolism, amino acid/protein metabolism, lipid metabolism, and the metabolism of cofactors and vitamins including fat digestion and absorption, fatty acid metabolism, fatty acid biosynthesis, amino sugar and nucleotide sugar metabolism, vitamin digestion and absorption, protein digestion and absorption, citrate cycle (TCA cycle), starch and sucrose metabolism, and carbohydrate digestion and absorption. Most of these pathways significantly enriched are closely related to the digestion and absorption of nutrients (Fig. 11B and Supplementary Table 7).

#### Validation of the transcriptome data by RT-qPCR

**Figure 12:**
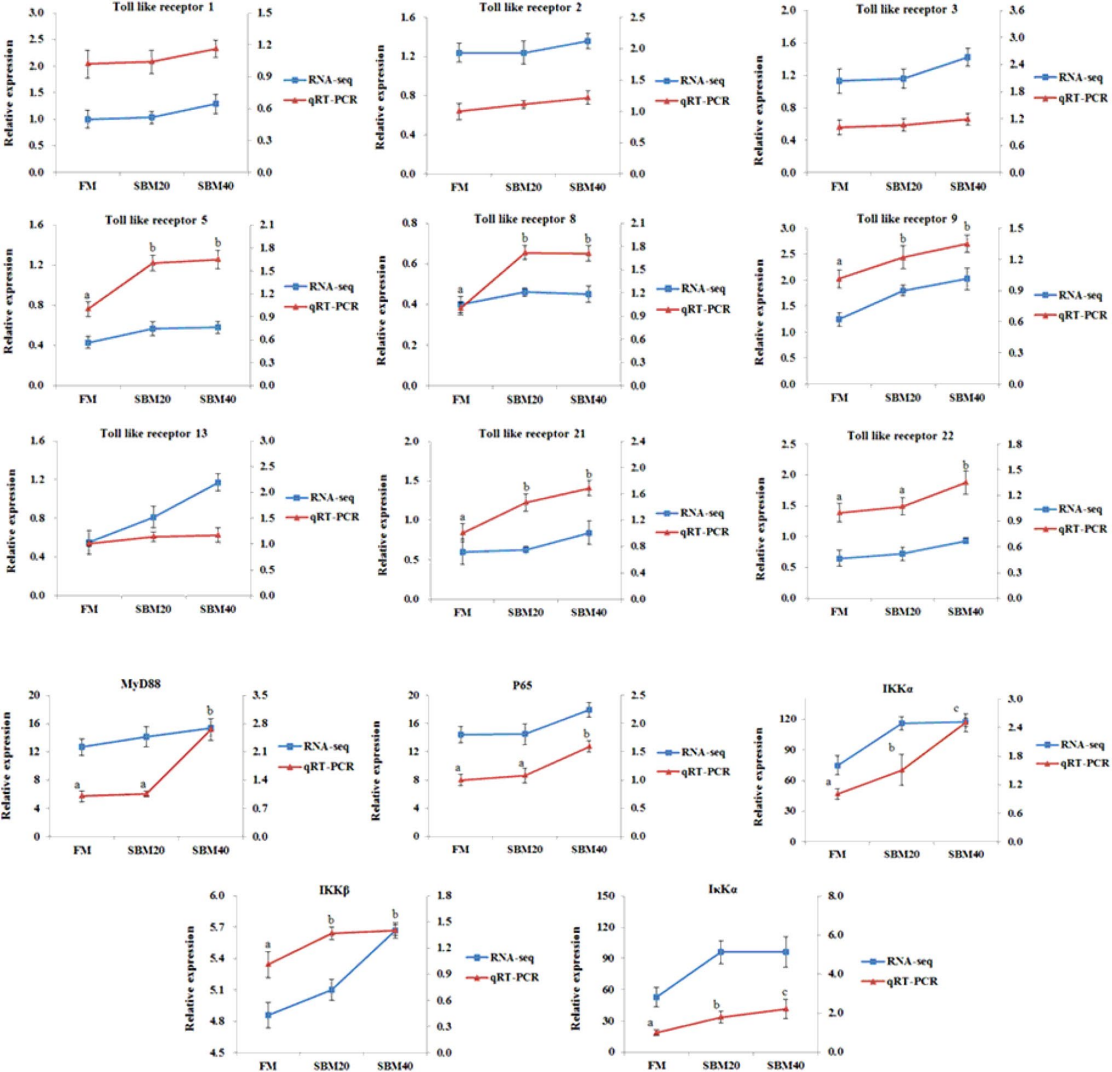
Comparison of RNA Seq and RT-qPCR results (n = 4). Note: in order to validate RNA-seq data, RT-qPCR was used to detect the gene expression level of the TLR-myD88-NF-κB pathway in DI tissue of pearl gentian grouper. The mRNA expression level of RT-qPCR was normalized by *β*-actin. The relative expression level in RNA-seq analysis was calculated by the FPKM value. The statistical results are expressed as mean ± SD. Different letters assigned to the lines represent significant differences between the groups at *P* < 0.05. FM, fish meal control group; SBM20, 20% SBM protein replacement level to FM protein; SBM40, 40% SBM protein replacement level to FM protein.

In order to validate the accuracy of “3 + 2” transcriptome data, in this study, 14 key genes in toll-like receptor signal transduction and the NF-κB signaling pathway related to immune and inflammatory development were selected for RT-qPCR validation. A total of 14 pairs of primers were designed based on the third-generation sequencing data. Overall, trends of RT-qPCR results were consistent with that of transcriptome, indicating that the transcriptome sequencing results were rather accurate (Fig. 12). These results further confirmed the reliability of the "3 + 2" transcriptome sequencing strategy.

### Western blotting

**Figure 13:**
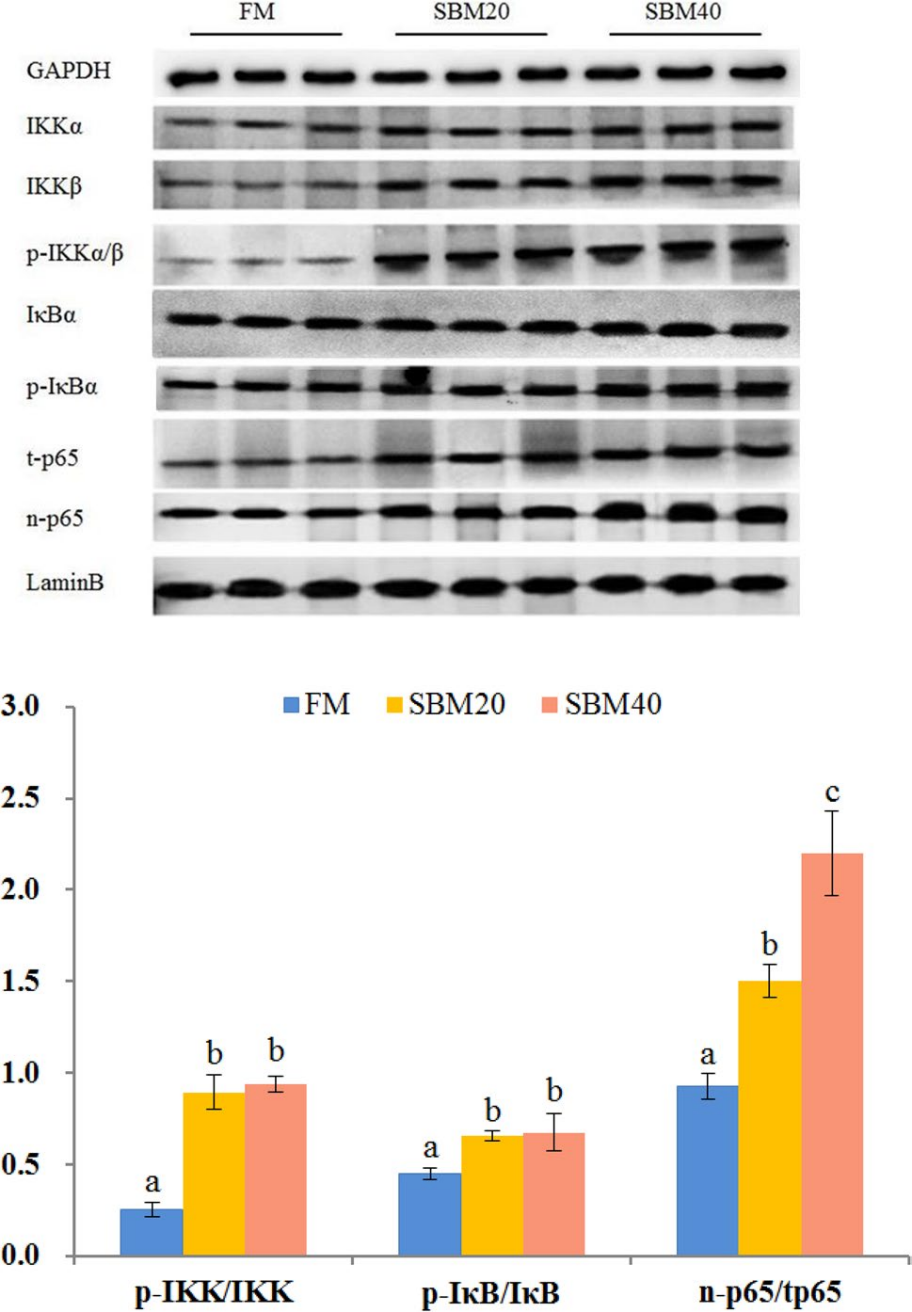
Western blotting analysis of activation of NF-κB signaling pathway in the hindgut of pearl gentian grouper fed by different substitution levels of soybean meal (n = 4). Note: Different letters assigned to the bars represent significant differences between the groups at *P* < 0.05. the The blots were cut prior to hybridisation with antibodies during blotting. GAPDH and LaminB were selected as the total protein and nuclear protein internal parameters, respectively. IKKα/β,inhibitor of IKKα/β kinase α/β; p-IKKα/β, phosphorylation inhibitor of nuclear factor kappa-B kinase α/β; IkBα, inhibitor of NF-κBα; p-IkBα, phosphorylation inhibitor of NF-κBα; t-p65, total p65; n-p65, nucleus p65. GAPDH, glyceraldehyde-3-phosphate dehydrogenase. All Western blotting’s were obtained from a complete gel band.

Expression analysis of key proteins in the NF-κB signaling pathway shows that the ratio of p-IKKα/β to IKKα and IKKβ, the ratio of p-IκBα to IκBα, and the ratio of n-p65 to t-p5 significantly increased with the increase of SBM substitution level (*P* < 0.05) (Fig. 13).

### Analysis of UPLC-MS profile

#### Metabolic profile of diets and contents

All DI diets and content samples were analyzed by UPLC-MS in positive and negative ion modes. Quality control shows that the data quality meets the analysis requirements. The detailed results are displayed in Supplementary file S1.

#### Filtration of differential metabolites in diets and contents

**Figure 14:**
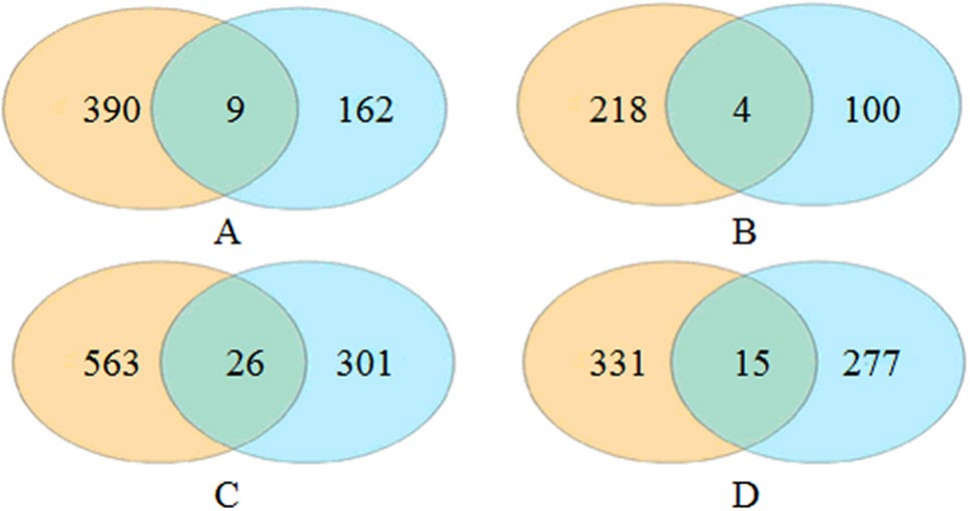
The Venn plot of the differential metabolites between diets and distal intestine contents groups in positive (**A**, **C**) and negative (**B**, **D**) modes (n = 12). Note: The red area represents the specific differential metabolites in intestinal contents of the SBM20_N group (**A**, **B**) or SBM40_N group (**C**, **D**) compared to the FM_N group, the blue area represents the specific differential metabolites in the diets of the SBM20_N (A, B) or SBM40_N (C, D) groups compared to the FM_N group. The overlapping area represents the co-contained metabolites of diets and DI contents.

The Venn plots show that, compared to the FM_N control group in positive ion mode, there were 399 differential metabolites in DI contents in the SBM20_N group, 9 of which were co-contained metabolites of diets and DI contents in the SBM20_N group. In the SBM40_N group, there were 589 differential metabolites in DI contents, 26 of which were co-contained metabolites of diets and DI contents. In negative ion mode, there were 222 differential metabolites in the SBM20_N group, 4 of which were co-contained metabolites of diets and DI contents. In the SBM40_N group, there were 346 differential metabolites in DI contents, 15 of which were co-contained metabolites of diets and DI contents (Fig. 14). Venn plots analysis show that the differential metabolites in DI contents were mainly derived from the physiological response of the intestine to diet metabolism.

In positive ion mode, 5 of 9 co-contained differential metabolites in the SBM20_N group showed contrary trends before and after feeding, which were considered to have changed significantly in the intestine. The 4 remaining showing the same trend and a variation range of Log_2_FC of less than 2 were removed from the total differential metabolites of DI contents (Supplementary Table 8). In the SBM40_N group, 16 of 26 co-contained differential metabolites showed contrary trends before and after feeding or the variation range of Log_2_FC was more than 2, and thus considered to be changed significantly in DI contents. The10 remaining were removed from the total differential metabolites of DI contents (Supplementary Table 9). Similarly, in negative ion mode, 1 of 4 co-contained differential metabolites in the SBM20_N group showed a contrary trend before and after feeding, which were considered to have changed significantly in the intestine, and the 3 remaining were removed from the total differential metabolites in DI contents (Supplementary Table 10). In the SBM40_N group, 8 of 15 co-contained differential metabolites showed a contrary trend before and after feeding or the variation range of Log_2_FC was more than 2, and thus considered to be changed significantly in DI contents. The 7 remaining were removed from the total differential metabolites of DI contents (Supplementary Table 11). Finally, the remaining differential metabolites in the DI contents were considered to be significantly changed due to the physiological metabolism of fish, and subsequent analysis was performed on these metabolites.

#### Differential metabolites in DI contents

**Figure 15:**
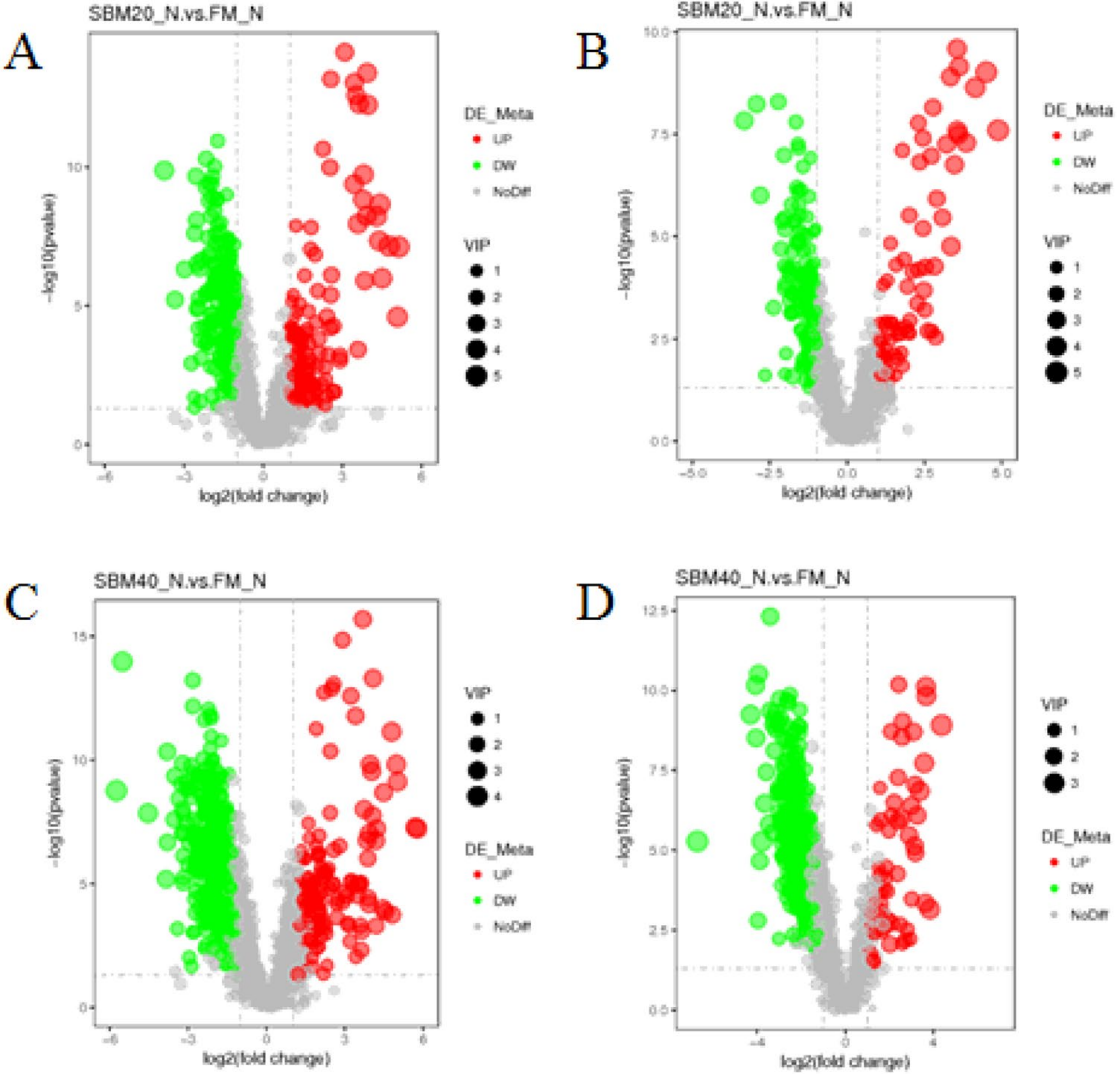
Volcano plot of *P* values between the FM, SBM20_N, and SBM40_N groups in positive (**A**, **C**) and negative (**B**, **D**) modes (n = 12). Note: Each dot represents a metabolite. Red dots represent significantly up-regulated metabolites, green dots represent significantly down-regulated metabolites, and gray dots represent metabolites with no significant difference. The point size represents the VIP value. FM_N, fish meal control group; SBM20_N, 20% SBM protein replacement level to FM protein; SBM40_N, 40% SBM protein replacement level to FM protein.

Compared to the FM_N control group, in positive and negative ion modes, 3160 metabolites were identified from the DI contents both in the SBM20_N and SBM40_N groups (2034 in the positive ion mode and 1126 in the negative ion mode). Subsequently, the retention time, *m/z*, and fragmentation patterns of the identified metabolites were compared with the standard substances. Then, the differential metabolites that did not change significantly before and after feeding were filtered. Volcano plots of the metabolites with significant changes in DI contents are shown in Fig. 15. In the positive ion mode, 151 differential metabolites in the SBM20_N group showed significant up-regulation and 244 differential metabolites showed significant down-regulation (Supplementary Table 12), whilst 150 differential metabolites in the SBM40_N group showed significant up-regulation and 429 differential metabolites showed significant down-regulation (Supplementary Table 13). Meanwhile, in negative ion mode, 73 differential metabolites in the SBM20_N group showed significant up-regulation and 146 differential metabolites showed significant down-regulation (Supplementary Table 14), whilst 58 differential metabolites in the SBM40_N group showed significant up-regulation and 281 differential metabolites showed significant down-regulation (Supplementary Table 15).

In positive ion mode, 395 differential metabolites in the SBM20_N group were enriched to 81 pathways (Supplementary Table 16), the top 5 of which are “Isoflavonoid biosynthesis”, “Phenylalanine metabolism”, “Tropane, piperidine and pyridine alkaloid biosynthesis”, “Biosynthesis of phenylpropanoids”, “Biosynthesis of alkaloids derived from shikimate pathway”, with the top 4 pathways significantly enriched (*P* < 0.05). A total of 579 differential metabolites in the SBM40_N group were enriched to 89 pathways (Supplementary Table 17), the top 5 of which are “Isoflavonoid biosynthesis”, “Linoleic acid metabolism”, “Bile secretion”, “Arachidonic acid metabolism” and “Methane metabolism”, with the top 2 pathways significantly enriched (*P* < 0.05). In negative ion mode, 219 differential metabolites in the SBM20_N group were enriched to 107 pathways (Supplementary Table 18, the top 5 of which are “Chlorocyclohexane and chlorobenzene degradation”, “Tyrosine metabolism”, “Isoflavonoid biosynthesis”, “Aminobenzoate degradation” and “Benzoate degradation”, with the top 3 pathways significantly enriched (*P* < 0.05). A total of 339 differential metabolites in the SBM40_N group were enriched to 85 pathways (Supplementary Table 19), the top 6 of which are “Pentose and glucuronate interconversions”, “Secondary bile acid biosynthesis”, “Arachidonic acid metabolism”, “Flavone and flavonol biosynthesis”, “Isoflavonoid biosynthesis” and “Biosynthesis of phenylpropanoids” and significantly enriched (*P* < 0.05).

#### Potential “core biomarkers” of enteritis in DI contents

According to the screening index mentioned above, in positive ion mode, the top 10 most influential metabolites distinguishing the SBM20_N group from the FM_N group were “5-methylbenzimidazole (C_8_H_8_N_2_)”, “Glycitin (C_22_H_22_O_10_)”, “Byakangelicol (C_17_H_16_O_6_)”, “Genistin (C_21_H_20_O_10_)”, “Malonylglycitin (C_25_H_24_O_13_)”, “Genistein 4'-O-glucuronide (C_21_H_18_O_11_)”, “Glycitein (C_16_H_12_O_5_)”, “amfonelic acid (C_18_H_16_N_2_O_3_)”, “3,4-dihydroxyphenylacetic acid (C_8_H_8_O_4_)” and “Daidzein (C_15_H_10_O_4_)”. (Supplementary Table 20). The top 10 most influential metabolites distinguishing the SBM40_N group from the FM_N group were “amfonelic acid”, “Genistin”, “Glycitin”, “3-Methoxyflavone”, “Daidzin”, “5-methylbenzimidazole”, “Glycitein”, “Malonylglycitin”, “Genistein 4'-O-glucuronide” and “Genistein” (Supplementary Table 21). Similarly, in negative ion mode, the top 10 most influential metabolites distinguishing the SBM20_N group from the FM_N group were “FMNH_2_ (C_17_H_23_N_4_O_9_P)”, “Glycitein (C_16_H_12_O_5_)”, “Soyasaponin I (C_48_H_78_O_18_)”, “olmelin (C_16_H_12_O_5_)”, “Genistein (C_15_H_10_O_5_)”, “Ginsenoside Ro (C_48_H_76_O_19_)”, “4-Phenolsulfonic acid (C_6_H_6_O_4_S)”, “Daidzein (C_15_H_10_O_4_)”, “Baicalin (C_21_H_18_O_11_)” and “Glycitin (C_22_H_22_O_10_)” (Supplementary Table 22). The top 10 most influential metabolites distinguishing the SBM40_N group from the FM_N group were “FMNH2”, “Soyasaponin I”, “Ginsenoside Ro”, “4-Phenolsulfonic acid”, “Glycitein”, “Genistein”, “Cys-Tyr”, “Indole-3-butyric acid”, “Glycitin” and “olmelin” (Supplementary Table 23).

**Figure 16:**
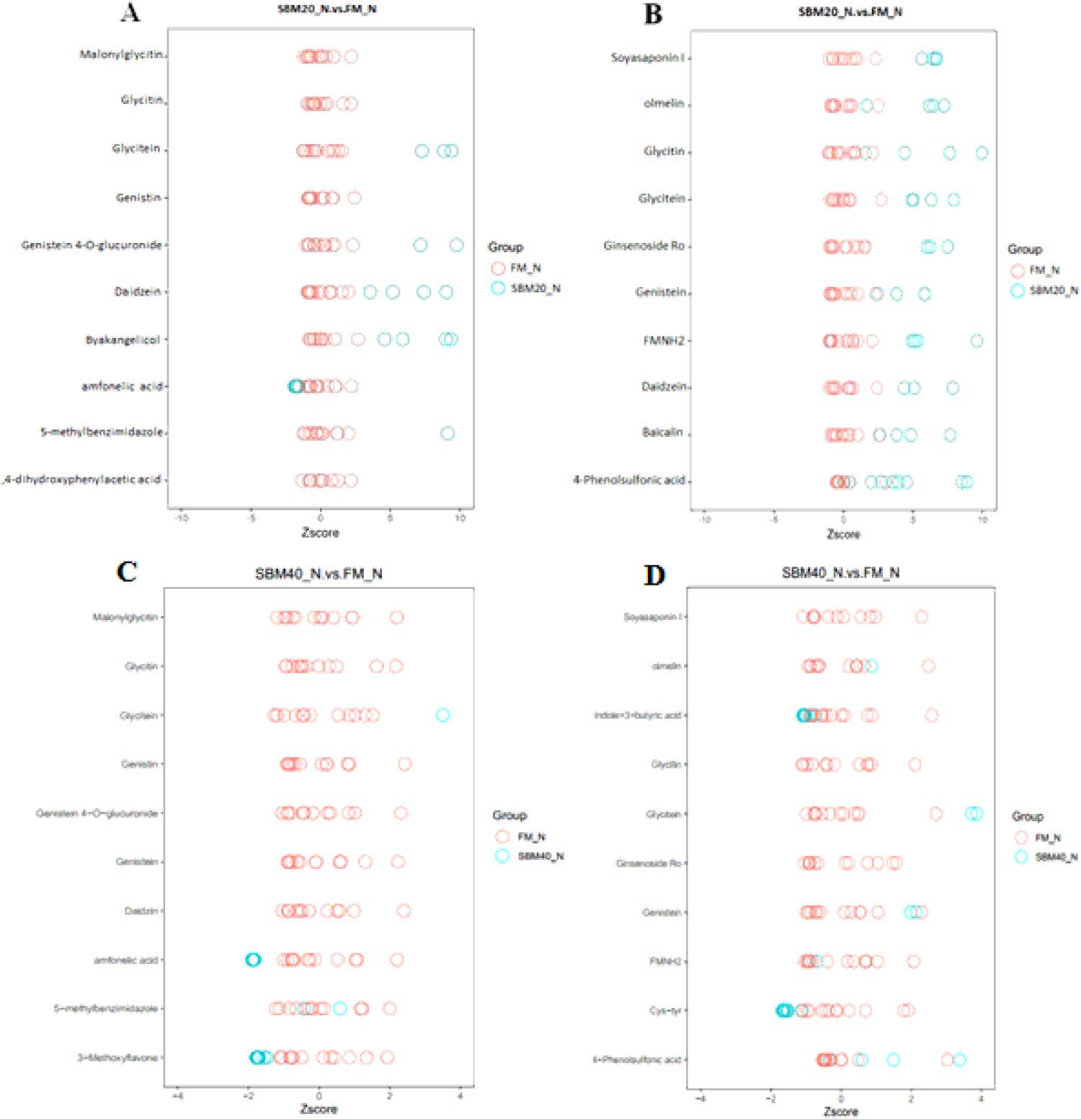
*z*-Score plot of the potential biomarkers for comparison between the FM, SBM20_N, and SBM40_N groups in positive (**A**, **C**) and negative (**B**, **D**) modes (n = 12). Note: FM_N, fish meal control group; SBM20_N, 20% SBM protein replacement level to FM protein; SBM40_N, 40% SBM protein replacement level to FM protein.

**Figure 17:**
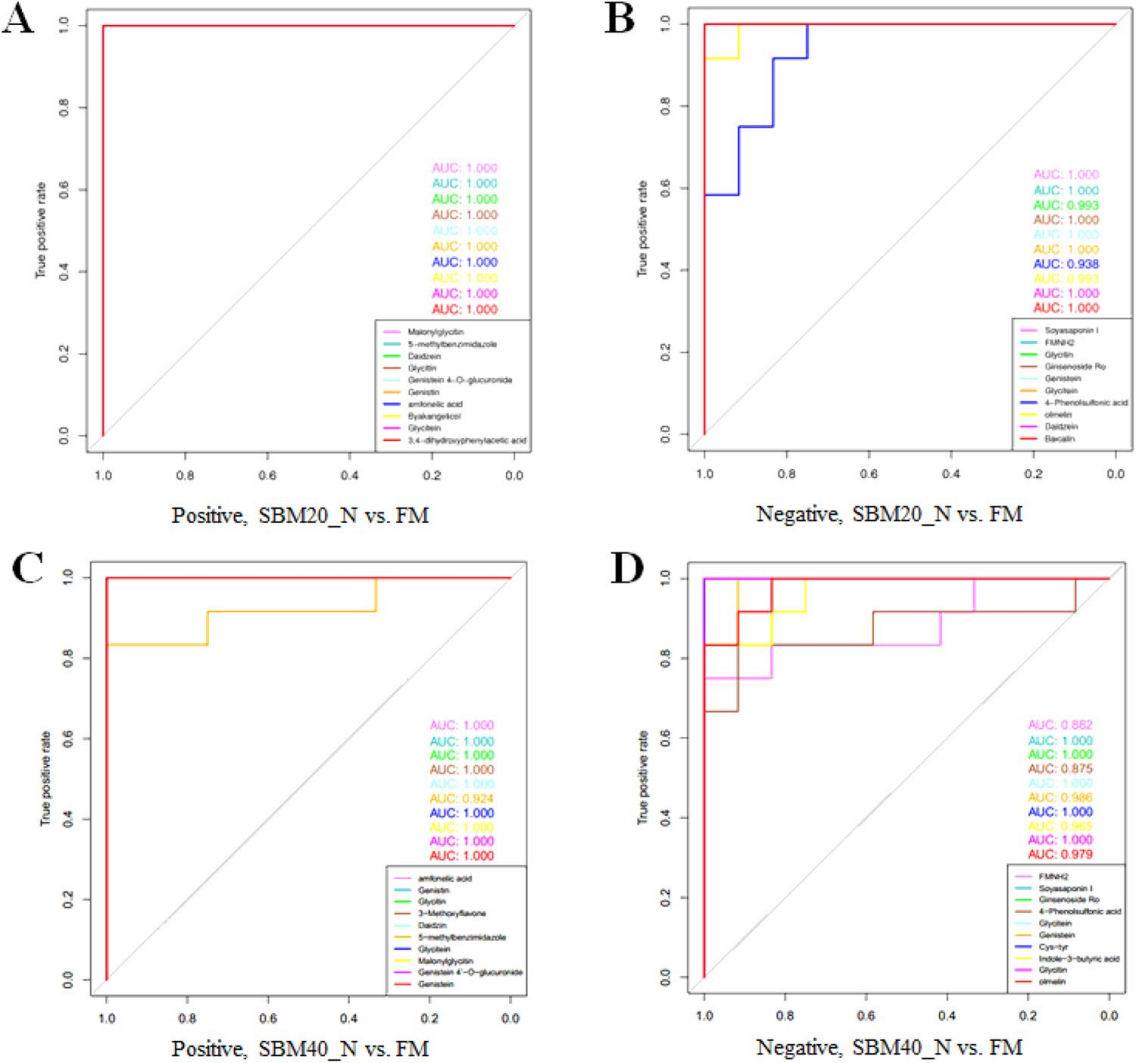
ROC analysis for discrimination among the groups for the potential biomarker metabolites in positive (**A**, **C**) and negative (**B**, **D**) modes (n = 12). Note: FM_N, fish meal control group; SBM20_N, 20% SBM protein replacement level to FM protein; SBM40_N, 40% SBM protein replacement level to FM protein.

In order to further clarify the characteristics of potential biomarkers, Fig. 16 shows the *z*-score plot in positive and negative ion modes. The intensities of the potential biomarkers in the positive and negative ion modes for the SBM20_N group are shown in Supplementary Table 24 and Supplementary Table 25, respectively. The ion intensities of the potential biomarkers in the positive and negative ion modes for the SBM40_N group are shown in Supplementary Table 26 and Supplementary Table 27, respectively. Compared to the FM control group, the ion intensity of amfonelic acid in SBM20_N group significantly decreased (*P* < 0.05), whereas the ion intensities of the other 16 metabolites significantly increased (*P* < 0.05). In the SBM40_N group, the ion intensities of amfonelic acid, 3-Methoxyflavone, and Cys-Tyr, Indole-3-butyric acid significantly decreased (*P* < 0.05), whereas the ion intensities of the other 13 metabolites significantly increased (*P* < 0.05). ROC analysis shows that the AUC of all the potential biomarkers exceeds 0.9 at the 95% confidence interval (Fig. 17), indicating a good predictive ability for the potential biomarkers.

**Figure 18:**
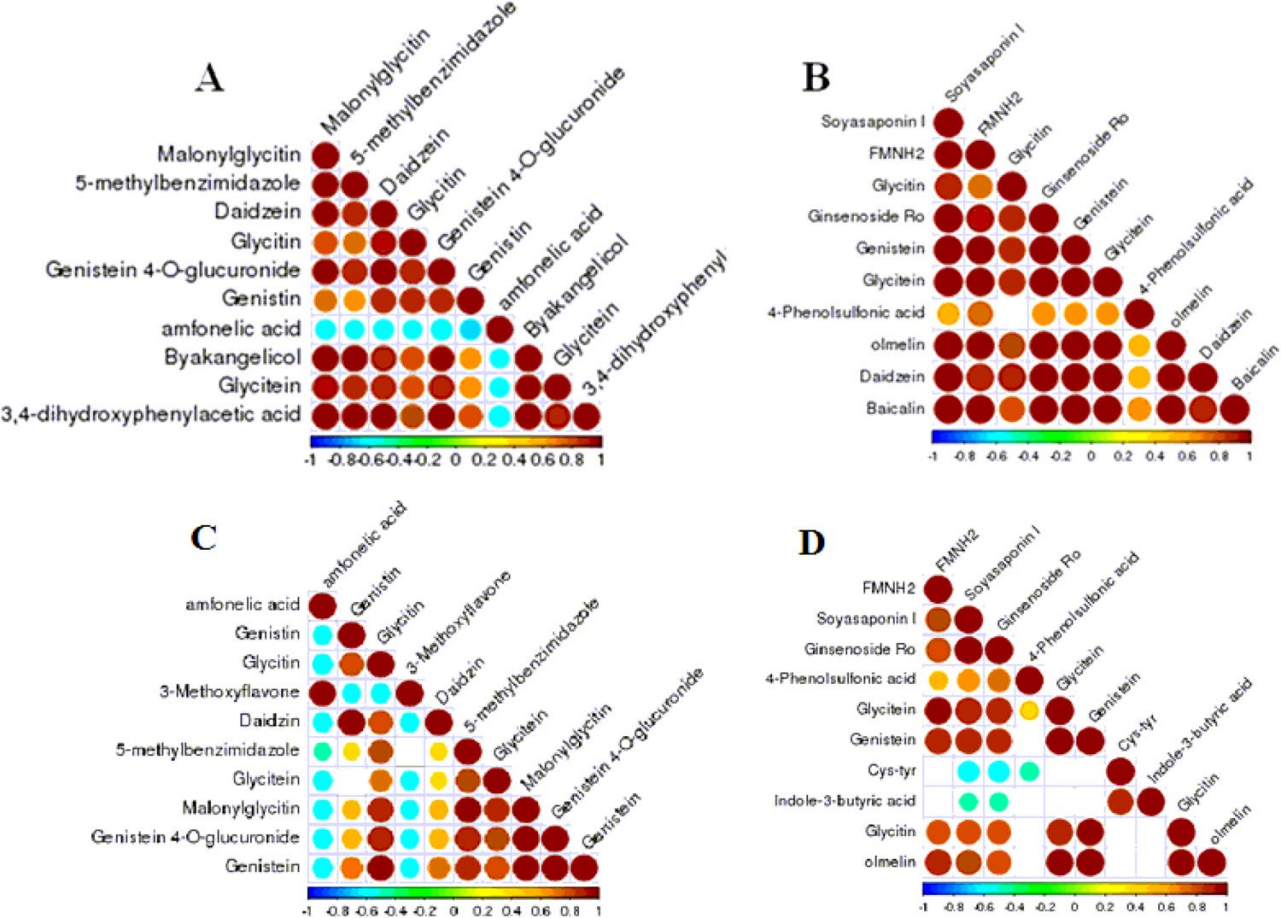
Correlation analysis of the potential biomarkers between FM and SBM20_N groups and FM and SBM40_N groups in positive (**A**, **C**) and negative (**B**, **D**) modes (n = 12). Note: The blank part is the significance level of the correlation statistical test *P* > 0.05, and the color marked part is the significance level *P* < 0.05. The highest correlation is 1, which is a complete positive correlation (red); the lowest correlation is—1, which is a complete negative correlation (blue). FM_N, fish meal control group; SBM20_N, 20% SBM protein replacement level to FM protein; SBM40_N, 40% SBM protein replacement level to FM protein.

In addition, correlation analysis in the SBM20_N group showed that there was a significant negative correlation between amfonelic acid and the other potential biomarkers in the positive ion mode, and a significant positive correlation between other markers in the positive and negative ion modes (Fig. 18A,B). In the SBM40_N group in positive mode, amfonelic acid was significantly positively correlated with 3-methoxyflavone and negatively correlated with other potential enteritis biomarkers. Similarly, 3-methoxyflavone was significantly negatively correlated with other potential enteritis biomarkers. In negative ion mode, Cys-Tyr was only negatively correlated with Soyasaponin I, Ginsenoside RO and 4-Phenolsulfonic acid, while Indole-3-butyric acid was only significantly negatively correlated with Soyasaponin I and Ginsenoside RO but positively correlated with Cys-Tyr. The rest of the other potential enteritis biomarkers mainly presented significant positive correlation among each other (except the blank, with no significant correlation) (Fig. 18C,D).

Statistical analysis among the 20 potential SBMIE biomarkers in positive and negative ion modes of the SBM20_N and SBM40_N groups show 3 metabolites to be co-contained in the positive and negative mode, respectively. Finally, 17 metabolites in each group were selected as potential biomarkers of enteritis in DI contents in the positive and negative ion modes, respectively (Table 8). Compared with the potential biomarkers of SBMIE at different substitute levels, 14 SBMIE biomarkers were relatively conservative in the SBM20_N and SBM40_N groups, which were called "core biomarkers". In addition to the “core biomarkers”, the specific biomarkers of the SBM20_N group included “Byakangelicol”, “3,4-dihydroxyphenylacetic acid” and “Baicalin”, while the specific biomarkers of the SBM40_N group included “Cys-Tyr”, “Indole-3-butyric acid” and “3-Methoxyflavone”.

**Table 8:** Comparison of potential biomarkers of enteritis in intestinal content at different substitute levels (n = 12).

SBM20_N vs. FM		SBM40_N vs. FM
1	5-methylbenzimidazole	5-methylbenzimidazole
2	Glycitin	Glycitin
3	Genistin	Genistin
4	Malonylglycitin	Malonylglycitin
5	Genistein 4'-O-glucuronide	Genistein 4'-O-glucuronide
6	Glycitein	Glycitein
7	Daidzein	Daidzein
8	FMNH_2_	FMNH_2_
9	Soyasaponin I	Soyasaponin I
10	olmelin	olmelin
11	Genistein	Genistein
12	Ginsenoside Ro	Ginsenoside Ro
13	4-Phenolsulfonic acid	4-Phenolsulfonic acid
14	amfonelic acid	amfonelic acid
15	Byakangelicol	Cys-Tyr
16	3,4-dihydroxyphenylacetic acid	Indole-3-butyric acid
17	Baicalin	3-Methoxyflavone

No. 1–14 metabolites are the potential “core biomarkers” in DI contents of SBMIE in pearl gentian grouper.

#### Metabolic profile of intestine tissues

In order to explore the metabolite exchanges between DI contents and intestinal tissues in the state of enteritis, UPLC-MS spectra of intestinal tissues were also obtained. Similar to the metabolic profile of diets and contents, quality control of the intestine tissue metabolic profile shows that the data quality also meets the analysis requirements. The detailed results are displayed in Supplementary file S1.

### Differential metabolites in intestinal tissues

PLS-DA score plots show that the FM control group and experimental groups could be separated without over-fitting the discovery set. Compared to the FM control group, 2260 metabolites were identified from intestinal tissues in both SBM20 and SBM40 groups (1404 in the positive ion modes and 856 in the negative ion modes). The retention time, *m/z*, and fragmentation patterns of the identified metabolites were compared with the standard substances. The volcano plots of the metabolites with significant changes in intestinal tissues are shown in Supplementary Fig. 3. In positive ion mode, 53 differential metabolites in the SBM20 group showed significant up-regulation and 120 differential metabolites showed significant down-regulation (Supplementary Table 28), whilst 69 differential metabolites in SBM40 group showed significant up-regulation and 293 differential metabolites showed significant down-regulation (Supplementary Table 29). Meanwhile, in negative ion mode, 52 differential metabolites in the SBM20 group showed significant up-regulation and 77 differential metabolites showed significant down-regulation (Supplementary Table 30), whilst 46 differential metabolites in the SBM40 group showed significant up-regulation and 190 differential metabolites showed significant down-regulation (Supplementary Table 31).

#### Inverse correlation analysis between DI contents and tissue

The intestinal contents and tissues of pearl gentian grouper in the SBM40 and FM control groups show inverse correlation analysis of the differential metabolites. First, the co-contained differential metabolites of DI contents and intestinal tissues were counted. Among them, the ion intensities of 38 differential metabolites significantly increased in DI contents, while significantly decreased in intestinal tissues (*P* < 0.05). The ion intensities of 18 differential metabolites significantly decreased in DI contents, while significantly increased in intestinal tissues (*P* < 0.05).

The top 10 representative metabolites with significantly higher ion intensities in DI contents and significantly lower ion intensities in intestinal tissues are shown in Table 9, including “Docosahexaenoic acid”, “Araloside A”, “Embelin”, “Leu-arg”, “Ginkgoic acid”, “Carglumic acid”, “L-Norleucine”, “Leucylproline”, “L-(-)-Asparagine” and “Asp-leu”. The top 10 representative metabolites with significantly lower ion intensities in DI contents and significantly higher ion intensities in intestinal tissues are shown in Table 10, which include “Cytosine”, “Cytidine”, “Indole-3-carbidol”, “3-(Methylsulfinyl)-L-alanine”, “2-Quinolinecarboxylic acid”, “Testosterone isocaproate”, “Tetrahydrodeoxycorticosterone”, “Estradiol enanthate, Nicotinamide” and “Valeroidine”. These metabolites may exchange between DI contents and intestinal tissues and play an important role in the development of enteritis.

**Table 9:** 10 representative metabolites of the intensities increased significantly in DI contents and decreased significantly in DI tissues (n = 12).

Metabolites	FM_N	SBM40_N	Trend	P value	FM	SBM40	Trend	P value
Docosahexaenoic acid	430,387,933	583,271,866	up	5.66E-05	238,785,562	200,036,643	down	0.047525
Araloside A	3678.8247	19,220.584	up	0.000958	5359.7504	2446.7346	down	0.014338
Embelin	3347.9281	7157.7361	up	0.044519	17,008.312	10,600.861	down	0.056358
Leu-arg	30,402.535	74,371.235	up	0.000818	273,255.73	116,795.89	down	0.007869
Ginkgoic acid	36,804.431	106,204.22	up	0.003378	38,980.066	16,631.79	down	0.000675
carglumic acid	719,426.5	2,170,944.2	up	0.003613	1,403,067.2	187,029.51	down	1.11E-11
L-Norleucine	26,973,473	49,992,149	up	0.00412	89,281,831	43,446,832	down	0.000194
Leucylproline	1,236,607.4	2,332,670.6	up	0.013949	1,930,426.2	907,885.44	down	0.000969
L-(-)-Asparagine	268,364.2	754,026.74	up	0.014648	721,920.86	388,931.09	down	0.002853
Asp-leu	892,558.05	1,608,231.6	up	0.02594	3,402,511.1	1,853,689	down	0.000603

FM_N, fish meal control group DI contents; SBM40_N, 40% substitute level group DI contents; FM, fish meal control group DI tissue; SBM40, 40% substitute level group DI tissues.

**Table 10:** 10 representative metabolites of the intensities decreased significantly in DI contents and increased significantly in DI tissues (n = 12).

Metabolites	FM_N	SBM40_N	Trend	P value	FM	SBM40	Trend	P value
Cytosine	74,390.77	25,886.01	down	0.002471	2765.373	4832.989	up	0.028388
Cytidine	1,329,923	568,340.1	down	0.045209	38,378.71	57,443.91	up	0.068651
Indole-3-carbidol	2,518,362	607,860.9	down	2.79E-06	49,120.75	85,215.99	up	0.02546
3-(Methylsulfinyl)-L-alanine	116,643.8	40,506.25	down	0.000317	10,994.99	22,349.21	up	0.004779
2-Quinolinecarboxylic acid	67,407.39	33,841.96	down	0.003245	20,500.78	50,827.34	up	0.000291
Testosterone isocaproate	1,565,093	598,249.9	down	0.003264	465,055.5	874,153	up	0.021607
Tetrahydrodeoxycorticosterone	882,945.4	131,680.4	down	0.003446	261,081.9	589,995.1	up	0.003542
Estradiol enanthate	2,538,970	833,770	down	0.005138	1,539,054	3,029,863	up	0.012127
Nicotinamide	5,662,292	2,868,804	down	0.035091	43,105,748	93,606,025	up	7.92E-11
Valeroidine	98,929.59	50,391.05	down	0.046873	3919.717	14,900.88	up	0.000232

Same as Table 9.

## Discussion

The present study shows that the growth performance, intestinal morphology, intestinal microflora composition and abundance, and immune-related gene expressions of pearl gentian grouper were clearly affected by the SBM substitute for FM. An et al.^33^ found that the substitute level of SBM for FM protein (65% basal FM protein) in the diet of *Epinephelus coioides* (initial weight of 84 ± 2.5 g) should not exceed 20%. Unpublished data of our laboratory also indicate that the optimum substitute level of SBM to FM in the diet of pearl gentian (initial weight of 17.01 ± 0.01 g) was 12.05% (basal FM protein 50%). This study shows that the FBW, WGR, and SGR of pearl gentian grouper (initial weights of about 12.55 g) are significantly lower than those of the FM control at 20% and 40% SBM substitute levels.

The intestine is not only an important organ for digestion and absorption of nutrients but also plays an important role in immune regulation, mucosal barrier, signal recognition, and endogenous active molecule production^34^. The digestive function of the intestine depends on the development of the intestine. At the same time, the structure and function of the intestine are also sensitive to the changes in the quality and quantity of feed nutrition, especially in the state of nutritional deficiency, which will show the most rapid and significant changes^35^. SBMIE is a common model to study enteritis in fish^8^. The histopathological changes of SBMIE have been widely studied, the characteristics of which are reduced mucosal fold height, swelling of lamina propria and submucosa, loss of absorption vacuoles in normal intestinal nuclei, and serious infiltration of various inflammatory cells, all of which result in a decreased ability of DI to digest and absorb nutrients^13^. The severity of intestinal histopathological changes in fish induced by SBM depends on soy protein content and inclusion body levels^36^. The present study shows that 20%-40% SBM is what causes the DI morphological changes of pearl gentian grouper, such as decreased plica, widened lamina propria, shortened microvilli, and increased inflammatory infiltration, decreased intercellular connectivity and increased apoptosis, the latter two of which gradually aggravated with the increase of SBM level. The enteritis index also significantly increased with the elevation of SBM substitute level, suggesting that the SBM used in this study induced enteritis in pearl gentian grouper.

The effect of SBMIE on pearl gentian grouper was also reflected in the changes in DI enzyme activities. Trypsin is a serine protein kinase that is an important component of intestinal immunity in mammals and fish^37^. One characteristic of SBMIE in fish is the increase of trypsin activity in the DI intestine^38^. Studies in mammals have also proved that trypsin can activate the pro-inflammatory mediator protease receptor 2^39^. In this study, trypsin activity in DI tissues of pearl gentian grouper also significantly increased with the increase of SBM content in the diet. The fish immune state depends on humoral immunity and cellular immunity to a great extent. The humoral immunity of fish includes nonspecific immunity and specific immunity. Complement and LYS are important components of nonspecific immunity. IgM is an important component of specific humoral immunity. C3 and C4 play a key role in the activation of the complement system^40,41^. The present study found that the addition of SBM in the diet reduced the contents of C3, C4, and IgM, which may be that SBM impaired the intestinal immune function of pearl gentian grouper. MDA is a kind of lipid peroxide produced by oxygen-free radicals attacking PUFA in biofilm. The content of MDA reflects the rate or intensity of lipid peroxidation in tissue cells, so the content of MDA can directly reflect the degree of oxidative damage^42^. The content of MDA in this study significantly increased with SBM addition in diets, indicating that SBM addition in the experimental diet results in the intestinal injury of pearl gentian grouper.

Cytokines can be divided into pro-inflammatory factors and anti-inflammatory factors according to their properties^43^. Related research has found that SBM can cause enteritis by increasing the expression of pro-inflammatory cytokines and reducing the expression of anti-inflammatory cytokines^35^. In some fish, SBM added into the diet can up-regulate the expression of pro-inflammatory genes, such as *Salmo salar*^44^, *Cyprinus carpio*^6^, *Danio rerio*^45^, *Scophthalmus maximus*^43^, *Oncorhynchus mykiss*^46^ and *Epinephelus coioides*^35^. Similar results were obtained in this study. The expression levels of pro-inflammatory factors such as *IL1*, *IL8*, *IL17D*, *TNF*α, and *CSF1* significantly increased along with SBM addition, while the expression levels of anti-inflammatory factors such as *IL4*, *IL10,* and *TGFβ1* significantly decreased along with SBM addition. The increase of anti-inflammatory gene expression may also inhibit the expression of the tight junction protein gene. Alsadi et al.^47^ indicated that most of the anti-inflammatory factors such as *IL1β*, *IFN-γ2*, and *TNFα* can lead to the destruction of the tight junction barrier of epithelial cells. Pan et al.^48^ also found that the down-regulation of inflammatory genes *IL-1β*, *IL-6*, *IL-8*, *IL-15*, *IL-17*, *IFN-γ2*, and *TNFα* were negatively correlated with the expressions of tight junction protein genes *claudin-3*, *-b*, *-c*, *occludin*, and *ZO-1*.

Intestinal microflora is a hot topic in this field of research. Microorganisms not only colonize in the host but also have effects on host physiology and immunity^49^. Therefore, healthy intestinal microflora is essential to promote the health of fish. Intestinal microflora is affected by many factors, such as breed, growth stage, and environmental factors, etc. Of course, diet composition is also an important factor^21^. A related study pointed out that the dominant bacterial phyla of intestinal microflora in pearl gentian grouper are Proteobacteria, Bacteroidetes, Firmicutes, and Actinobacteria^50^. Similar results were obtained in this study. At the phylum level, the abundance of Proteobacteria was dominant in both the FM control and experimental groups, followed by Firmicutes, Bacteroidetes, and Actinobacteria. However, the abundance of dominant microflora varied with SBM addition. In general, the abundance of Proteobacteria decreased with SBM increase, while the abundance of Firmicutes and Bacteroidetes increased gradually. Consistent with the results of this study, Hu et al.^51^ pointed out that 55% SBM significantly increases the abundance of Firmicutes and Bacteroidetes in the intestine of turbot. The reason may be that Firmicutes and Bacteroidetes can use unabsorbable oligosaccharides, phytoestrogens and non-starch polysaccharides in SBM as energy sources to promote their proliferation^52^. At the same time, some studies have pointed out that the occurrence of enteritis may be accompanied by the abundant decrease of Proteobacteria and the abundant increase of Firmicutes and Bacteroidetes^51^. However, most research about patients with Ulcerative Colitis (UC) found that the abundance of Proteobacteria increased^53^, which may be due to the different species. The intestinal microflora of healthy people was mainly Firmicutes and Bacteroidetes, but less Proteobacteria and Actinobacteria^54,55^. At the genus level, *Photobacterium* belongs to Vibrionaceae and facultative anaerobic bacteria, which live in seawater, body surfaces and digestive tracts of some marine fish, and usually produce acetylmethyl methanol instead of indole^56^. Although the abundance of *Photobacterium* significantly decreased in the SBM group, it was not clear whether the difference was mainly due to the influence of FM. *Bacteroides*, *Fusicatenibacter*, *Rothia*, and *Intestinibacter* are mainly anaerobes, the abundance of which significantly increased in the SBM experimental group. Corresponding to this, in the functional prediction, the aerobic_chemoheterotrophy abundance in functional annotation significantly decreased in the SBM experimental group. Anaerobic and aerobic are a pair of contradictory unity that depend on and restrict each other in a normal microbial community^57^. In the present study, the animal_parasites_or_symbiont functional abundance of intestinal microflora significantly increased in the SBM40 group. Results showed that excessive addition of SBM caused the dysfunction of intestinal microflora and an increase of pathogenic bacteria. This is also reflected in our unpublished comparative transcriptome results, which show that the two-component system pathway plays an important role in the bacterial response to external stimuli and the completion of the pathogenic process^58^. Currently, 16S high-throughput sequencing technology is mainly used in the study of fish intestinal microflora, but many sequences obtained cannot be annotated at the species level. Metagenomic sequencing technology can identify the intestinal microflora at a species level and even at a strain level, which can provide an important reference for the regulation of fish intestinal health through intestinal microflora, which will be further explored in the future research.

In order to have a comprehensive understanding of SBMIE in fish, "3 + 2" transcriptome analysis was carried out in this study. Previously, the transcriptome analysis of plant protein-induced enteritis in fish mainly used DNA microarrays, which found conservative changes in genes and signal pathways. Kortner et al.^59^ found that pea protein concentrate combined with soyasaponin can induce DI enteritis and immune gene expression changes in Atlantic salmon such as the up-regulation of inflammatory cytokines, NF-κB signaling and TNF-α signaling pathway-related genes and T cell function regulatory factors. Sahlmann et al.^3^ revealed that SBM can cause DI tissue enteritis of Atlantic salmon and increase the expression of immune-related genes including the GTPase IMAP family, the NF-κB signaling pathway, the IL-8 signaling pathway, and regulatory factors of T cell and B cell function, and then down-regulate transcripts related to endocytosis, exocytosis, detoxification, transportation, and metabolism, thus suggesting intestinal barrier and function damage. De Santis et al.^60^ reported that the diet containing 30% SBM changed the expression of immune-related genes in DI tissue of Atlantic salmon including phagocytosis and antigen processing and presentation. Romanheim et al.^61^ revealed that probiotics added to diets containing SBM may prevent enteritis by normalizing the DI tissue barrier function of Atlantic salmon. Grammes et al.^62^ indicated that probiotics added to diets containing SBM could normalize the expression of NOD-like signaling pathways, the Chemokine signaling pathway, and the gene expression encoding antimicrobial peptide in DI tissue of Atlantic salmon to prevent enteritis. In addition to the NF-κB signaling pathway, the TNF signaling pathway, Th17 cell differentiation, the NOD-like receptor signaling pathway, Th1 and Th2 cell differentiation, Th17 cell differentiation and antigen processing and presentation, the Intestinal immune network for IgA production, Inflammatory bowel disease (IBD), the Toll-like receptor signaling pathway and JaK-STAT signaling pathway were also significantly affected by SBM addition in diets. In the significantly up-regulated signaling pathways, NAFLD—a signal pathway related to liver disease—was also observed in this study, suggesting that liver lesions may occur in the process of SBMIE. This can also be reflected by the significant increases in ALT and AST enzyme activities in the liver of pearl gentian grouper. ALT and AST are two common sensitive indicators of hepatocyte injury. When liver lesions occur, necrotic tissue cells release a large number of enzymes, which will lead to a significant increase in ALT and AST content^63^. The pathological changes of liver tissue were also found in both Atlantic salmon and grass carp SBMIE^60,64^.

This study further analyzed the down-regulated genes in trend analysis of the DI intestine. The results found that most of the significantly enriched metabolic pathways related to the digestion and absorption of amino acids/proteins, fats, carbohydrates and vitamins were inhibited (65.91%), indicating that the intestinal function for nutrient digestion and absorption was impaired. The effects of plant proteins on intestinal digestion and absorption in fish were rarely reported at the omics level, but previous studies at single or multiple gene levels showed that it might be related to anti-nutritional factors such as protease inhibitors, phytate, saponins, lectins, gossypol, phytosterol and oligosaccharide^62,65^. However, our unpublished transcriptome comparative study of soy protein concentrate (SPC, ethanol extraction) and SBM found that anti-nutritional factors may not be the only major influential factors on the formation of fish SBMIE. SBM may also contain other unknown ingredients that have important impacts on the development of enteritis. In the comparative experiment, the contents of anti-nutritional factors in SBM and SPC diets were detected. It was found that most of the anti-nutritional factors were removed from the SPC diet, including soyasaponin, soybean globulin (7S, 11S), and soybean isoflavones, which were considered to be the main contribution to SBMIE in fish. However, transcriptome comparative analysis showed that SPC with a high substituted level still had a wide range of significant effects on the signal pathway of intestinal nutrient digestion and absorption function in pearl gentian grouper 69.23% (27/39), while only 5.13% (2/39) of the SPC group had significant effects on immune and disease-related pathways. This was much lower than 43.55% (27/62) in the SBM group. Therefore, the present study speculates that SBMIE in pearl gentian grouper may be the synactic results of intestinal immune dysfunction and imbalance of intestinal nutrition metabolism together. The determination of which one plays a leading role requires further study.

SBMIE in fish is a complex process. According to the above analysis, the NF-κB signaling pathway, which is closely related to the development of enteritis, is quite conservative in the process of SBMIE in pearl gentian grouper. Meanwhile, intestinal microflora plays an important role in the regulation of the intestinal mucosal immune response. The role of intestinal microflora has been confirmed in the pathogenesis of inflammatory bowel disease (IBD) in humans and other animals^22,66,67^. For example, Crohn's disease patients treated with sterile ultrafiltration of small intestinal exudate did not show intestinal enteritis, however, the re-entry of intestinal exudate led to enteritis^68^. Pathogen infection can induce a host inflammatory response due to the host’s ability to sense pathogen signals and thus initiate response through pattern recognition receptors (PRRS). Four PRRS have been identified, including Toll-like receptors (TLRs), RIG-I-like receptors, NOD-like receptors, and C-type lectin receptors. Among them, TLRs are the most deeply studied in mammals^69^. Transcriptome analysis in this study found that the first three PRRs signaling pathways were significantly activated and enriched, indicating that intestinal pathogenic microflora may affect the development of enteritis in pearl gentian grouper.

TLRs play an indispensable role in congenital immunity. They are the first line of defense against pathogen invasion and are important for inflammation, immune cell regulation, survival, and proliferation. In the study of (sterile) *Danio rerio* as a model of enteritis, it was found that the induction of enteritis depended on intestinal microflora and the transduction of TLRs signaling pathway^70,71^. Other researchers discussed a mechanism of intestinal microorganisms in regulating intestinal immunity and suggested that TLRs could recognize symbiotic bacteria under normal conditions. The interaction between TLRs and symbiotic bacterial products plays an important role in the stabilization of the intestinal environment and the resistance to epithelial injury. It can recognize pathogen-related molecular patterns, such as lipopolysaccharide (LPS), double-stranded RNA (dsRNA), flagella, etc., and promote a range of immune defense mechanisms^72^. The dysfunctional interaction between bacteria and TLRs may promote the development of chronic inflammation. TLR-NF-κB is the main component of inflammation and immune response in organisms^73^. Based on the above analysis, the present study focuses on the role of the TLR-NF-κB signaling pathway in the development of SBMIE in pearl gentian grouper.

By full-length transcriptome sequencing, the present study found nine TLR members in the DI tissues of pearl gentian grouper, including TLR1, TLR2, TLR3, TLR5, TLR8, TLR9, TLR13, TLR21, and TLR22. TLRs are different in different fish species. With the exception of TLR8, the members of the above eight TLR species were reported in *Epinephelus coioides*. TLR8 was also found in channel catfish, grass carp and zebrafish. Currently, at least 20 kinds of TLRs have been found in fish. Among the TLRs found in this experiment, TLR1, TLR2, TLR3, TLR5, TLR9, TLR21, and TLR22 have been reported in fish as sensors for bacterial ligands. Wei et al.^74^ pointed out that bacterial lipopolysaccharide and poly (I:C) could up regulate the expressions of *TLR1* and *TLR2* in the spleen and head kidney of *Epinephelus coioides*. Tsujita et al.^75^ found that *Vibrio anguillarum* or its flagellum could activate the expression of *TLR5* in rainbow trout, and flagellin mediated the significant activation of the NF-κB signaling pathway. Byadgi et al.^76^ revealed that CpG ODN can significantly promote the expression of *TLR9* and pro-inflammatory factors in the liver and spleen of *Rachycentron canadum*. Yeh et al.^77^ indicated that *TLR9* and *TLR21* have similar expression profiles in zebrafish, and synergistically mediated the antibacterial activity of CpG ODN. *TLR22* is a fish-specific TLR. Unlike *TLR3*, *TLR3* is located in the endosome/lysosome, which can recognize short dsRNA, while *TLR22* is located on the cell surface to recognize long dsRNA^78^. The present study showed that the expression levels of *TLR5*, *TLR8*, *TLR9*, *TLR21* and *TLR22* significantly increased with an SBM increase, indicating that intestinal pathogenic microflora activates TLRs signal transduction through a variety of bacterial components/products.

When TLRs are activated, they recruit relevant adaptor proteins in cytoplasm and trigger different signaling cascades. There are two kinds of TLRs signaling pathways: MyD88-dependent and MyD88-independent (TRIF dependent)^79^. The intracellular components of TLRs downstream are highly conserved between mammals and teleost^80^. Studies have shown that MyD88 can be recruited by all TLR members except *TLR3* to activate downstream the NF-κB signaling pathway and the mitogen-activated protein kinase signaling pathway^81,82^. However, in the MyD88-independent signaling pathway, TRIF can only be recruited by *TLR3* and *TLR4* to activate the NF-κB signaling pathway and transcription factor *IFR3*^83^. In this study, the *TLR4* gene was not found in pearl gentian grouper, and the expression level of *TLR3* did not significantly change with SBM addition. However, *MyD88* and the expression and protein content of key genes (*IKKα*, *IKKβ*, *IκBα* and *p65*) in the NF-κB signaling pathway significantly increased with an increase in SBM, indicating that the TLR-MyD88-NF-κB signaling pathway plays an important role in the pathogenesis of SBMIE in pearl gentian grouper.

In order to reveal the characteristics of SBMIE in pearl gentian grouper from multiple dimensions, the present study applied UPLS-MS to analyze the changes of the metabolizable spectrum in DI contents of pearl gentian grouper after SBM substitution for FM. In metabolomics of DI contents, there were significant pattern differences between the FM group and the experimental group. In the PLS-DA score plot, the FM_N group was obviously separated from the SBM20_N and SBM40_N groups, indicating that the change of the metabolism spectrum of DI contents might be due to the addition of SBM in diets, and SBMIE could be reflected at the metabolic level.

The VIP value is used to reflect the variable importance in the projection and is often used in metabolomics to screen potential biomarkers^84^. In the metabolomics of DI contents, 17 metabolites were screened as the potential biomarkers of SBMIE in pearl gentian grouper at different substitution levels of SBM. By comparison, there were 14 rather conservative "core biomarkers" in the SBM20_N group and SBM40_N group, mainly isoflavones and saponins. The present study discusses the biological relationship among the main potential biomarkers and their roles in intestinal health.

Isoflavones are a class of molecules called flavonoids, belonging to the polyphenol family^85^. Isoflavones are similar to natural estrogens in structure and exert estrogen-like biological effects in animals^86^. SBM also contains phytoestrogens, known as isoflavones, a class of molecules that belong to polyphenols called flavonoids^85^. Studies have found that phytoestrogens are harmful to the reproductive system of animals^87^ and beneficial to human health^88^. At present, it is generally believed that soybean isoflavones mainly include 12 compounds, which can be divided into two types: free aglucons and conjugated glycosides. The free aglucons mainly include genistein, daidzein and glycitein; the conjugated glycosides mainly include genistin, daidzin, malonylgenistin and malonyldaidzin. In this study, among the 14 potential "core biomarkers", 8 were flavonoids, including Glycitin, Genistin, Malonylglycitin, Genistein 4'-O-glucuronide, Glycitein, Daidzein, Olmelin and Genistein. In mammals, studies have shown that isoflavones have a wide range of biological activities, such as anti-estrogen, antioxidantion, anti-inflammation, anticancer, cardioprotective, enzyme-inhibition, antifungal, enhancing non-specific immunity and anti-stress^89–91^, etc. Soybean isoflavones, also known as growth promoters, are used in poultry farming to increase yields^92^. However, the effects of soybean isoflavones on fish growth performance, feed utilization and immune response are controversial, which may be related to the type and sex of the subjects and the dosage of soybean isoflavones, *etc*^93^. In addition, an appropriate amount of soybean isoflavones can ameliorate the composition of human intestinal microflora and promote the proliferation of colon epithelial cells^94,95^. However, Chen^96^ found that when dietary soybean isoflavones reached 0.8%, the hindgut integrity of Japanese flounder was damaged. The content of isoflavones in SBM ranged from 0.1% to 0.35%. When the content of soybean isoflavones was less than 0.4%, there was no negative effect on the growth and intestinal physiology of *allogynogenetic crucian carp* and Japanese flounder^97^. However, when SBM is added as a whole to replace FM, the negative effects of soy isoflavones cannot be excluded, because there may be synergistic effects among anti-nutritional factors in diets, such as soyasaponins.

The content of saponins in SBM ranged from 5–7 g/kg. The present study found that 2 of the 17 potential biomarkers were saponins, including Soyasaponin I and Ginsenoside Ro. Recent studies pointed out that the main anti-nutritional factor causing enteritis in Atlantic salmon is soybean saponin. However, the direct effect of soyasaponins is complex, and the interactions among different ANFs are also very important^27^. Zhang et al.^97^ found that although the negative effects of SBM replacing FM on the *Allogynogenetic crucian carp* may not be related to soybean isoflavones, there is a synergistic effect between soybean saponin and soybean isoflavones. Few literatures report the application of Ginsenoside Ro in aquaculture, but similar to saponins, the appropriate use of Ginsenoside Ro in mammals has shown to exhibit immune-stimulating effects^98,99^. This study, however, shows there is a strong positive correlation between Ginsenoside Ro and Soyasaponin I. It is, therefore, speculated that Ginsenoside Ro may cause enteritis, however, the specific situation requires further study.

Except for the above reports, the remaining 7 of the 17 potential biomarkers in the SBM20_N group are FMNH_2_, 5-methylbenzimidazole, 4-Phenolsulfonic acid, amfonelic acid, Byakangelicol, 3,4-dihydroxyphenylacetic acid and Baicalin. Among them, Baicalin also belongs to flavonoids. The remaining 7 of the 17 potential biomarkers in the SBM40_N group are FMNH_2_, 5-methylbenzimidazole, 4-Phenolsulfonic acid, amfonelic acid, Cys-Tyr, Indole-3-butyric acid and 3-Methoxyflavone. Only a few studies on these metabolites for fish SBMIE exist. Compared with the FM control group, the present study found that the ion intensities of these metabolites in the two substitution groups were significantly different. FMNH_2_ (i.e. the reduced flavin mononucleotide), plays an important role in electron transport during respiration and other biological oxidation processes^100^, suggesting that the biological oxidation process in enteritis is disordered. Indole compounds have anti-inflammation, anticancer, antibiosis, antiviral, free radical scavenging and neuroprotective effects. Related studies have pointed out that indole-3-propionic acid (IPA) is a metabolite produced entirely by microorganisms that metabolize tryptophan in the diet, which accumulates in the host serum and shows a high degree of inter-individual variation^101^. Previous studies have shown that a few bacteria, including *Clostridium sporogenes* and *Clostridium botulinum*, can produce IPA from tryptophan, which has a certain strengthening effect on the intestinal barrier function^102,103^. The symbiotic bacteria in the digestive tract of mammals and birds can metabolize tryptophan to produce IPA^104^. Currently, the application of indole compounds in aquaculture is rarely reported. Studies on mammals indicate that external IPA can directly inhibit NF-κB signaling in rats with fatty liver disease and reduce inflammation factor levels to alleviate hepatitis and liver injury^105^. Indole, which is metabolized by human intestinal flora, can also inhibit the NF-κB signaling pathway, reduce inflammatory factors and increase the level of anti-inflammatory factors, alleviate intestinal inflammation, reduce the chemotaxis, translocation and epithelial cell adhesion of pathogenic *Escherichia coli*^102^. The small peptide is the main product of protein hydrolysis and the main form of absorption after the animal digesting protein. It is composed of two or more amino acids with a relative molecular weight between 1000 and 1800. Generally speaking, small peptides refer to dipeptides and tripeptides. Small peptides have a variety of biological functions, such as protecting intestinal structure and function, promoting protein absorption and utilization, improving animal growth performance, ameliorating animal immune performance and antioxidant effect. In vitro experiments showed that sardine muscle hydrolyzed small peptides, such as Val-Tyr, Ile-Tyr, Tyr-Val and Trp-His, could induce relaxation of the constricted aorta to prevent atherosclerosis^106^. Small peptides from food sources, such as egg (Ile-Arg-Trp, Ile-Gln-Trp), fermented SBM (Val-Pro-Pro, Ile-Pro-Pro and Leu-Pro-Pro) and SBM (Val-Pro-Tyr), have also been found to have anti-inflammatory, antihypertensive, hypoglycemic, and renal function improvement effects, *etc*^107^. The present study found that the contents of indole-3-butyric acid and small peptide Cys-Tyr were significantly decreased in the experimental group, suggesting that the intestinal structure and function may be damaged. In this experiment, although most of these potential markers have obvious correlation, further studies are still required to determine whether their effects on enteritis are independent, synergistic or antagonistic.

Through the inverse correlation analysis of metabolomics of DI contents and metabolomics of DI tissue, this study explored which metabolites absorbed or excreted in the DI intestine had significant changes in the development of enteritis and its possible role. Docosahexaenoic acid, namely DHA, as an omega-3 fatty acid derived from fish has an anti-inflammatory effect and can reduce the expression of inflammatory factors *PGE2* and *IL6*^108^. Araloside A can effectively inhibit gastric lesion formation and ulcers in rats^109^. In human studies, it was also found that Araloside A could promote apoptosis and anti-inflammatory effects in fibroblast-like synoviocytes of rheumatoid arthritis by inhibiting the NF-κB pathway^110^. Embelin can inhibit the expression of inflammatory factors and promote apoptosis^111^. In the study of arthritis in rats, Embelin can reduce serum IL-6 and IL-17A and relieve the symptoms of arthritis^112^. Ginkgoic acid is an important bioactive component in Ginkgo biloba leaves except flavonoids and lactones^113^. Studies have shown that Ginkgoic acid has a strong antioxidant capability and a good inhibitory effect on high capillary permeability, inflammatory exudation and edema in the early stage of inflammation^114^. Citric acid is a good antioxidant and antioxidant synergist, which can effectively remove superoxide anion and lipid peroxide and reduce the oxidative stress of animals^115^. In addition to the substances mentioned above, the contents of some amino acids and small peptides in DI tissues of SBM group were significantly decreased, suggesting that these substances play an important role in the development of enteritis. For example, L-Norleucine, although rarely reported on the role of L-Norleucine on inflammation, studies have pointed out that L-Norleucine and its derivatives can inhibit the metastasis of tumor cells^116^. Meanwhile, the present study also discovered that some substances in DI tissues increased significantly. It was found that the content of cytosine in gastritis tissue of rats significantly increased, with some studies indicating that both cytosine riboside and cytosine nucleotide had the effect of increasing leukocyte^117,118^. Indol-3-carbidol (I3C) is an indole compound found in cruciferous vegetables such as cabbage and broccoli. In fish and mammals, it has been confirmed that I3C has a good protective effect on hormonal or chemical carcinogenic models^119^ and can significantly relieve the severity of colitis in rats by down-regulating pro-inflammatory mediators^120^. Estradiol is involved in a variety of cardiovascular diseases, immune responses and inflammatory stress responses^121^. In human studies, inflammation has been found to promote the synthesis of estradiol in the cerebellum in early childhood^122^. Nicotinamide, also known as vitamin B5, has been found to inhibit the expression of inflammatory genes through the NF-κB pathway^123^. Although the above substances exhibited certain anti-inflammatory effects, the pearl gentian grouper showed enteritis symptoms on the whole after feeding SBM. The role of these substances in the process of intestinal immune balance needs to be further revealed.

There are also certain limitations in this research. In metabolomics, only LC–MS method was used. In future research, the advantages of GC–MS and NMR technologies could be combined for more detailed and in-depth analysis. There is no model animal of marine fish at present and the cell culture of marine fish intestine still lacks understanding, therefore it is impossible to carry out corresponding research at the cell level. For example, the dual luciferase reporter assay was used to study the "dialogue" mechanism (NF-κB signaling pathway) between intestinal microflora and host in enteritis. To study the mechanism of FM substitution and SBMIE is of great significance both in aquaculture and in most terrestrial animals, including human beings. In the future, it is necessary for researchers to continuously work together in this field.

## Methods

### Experimental design

**Figure 19:**
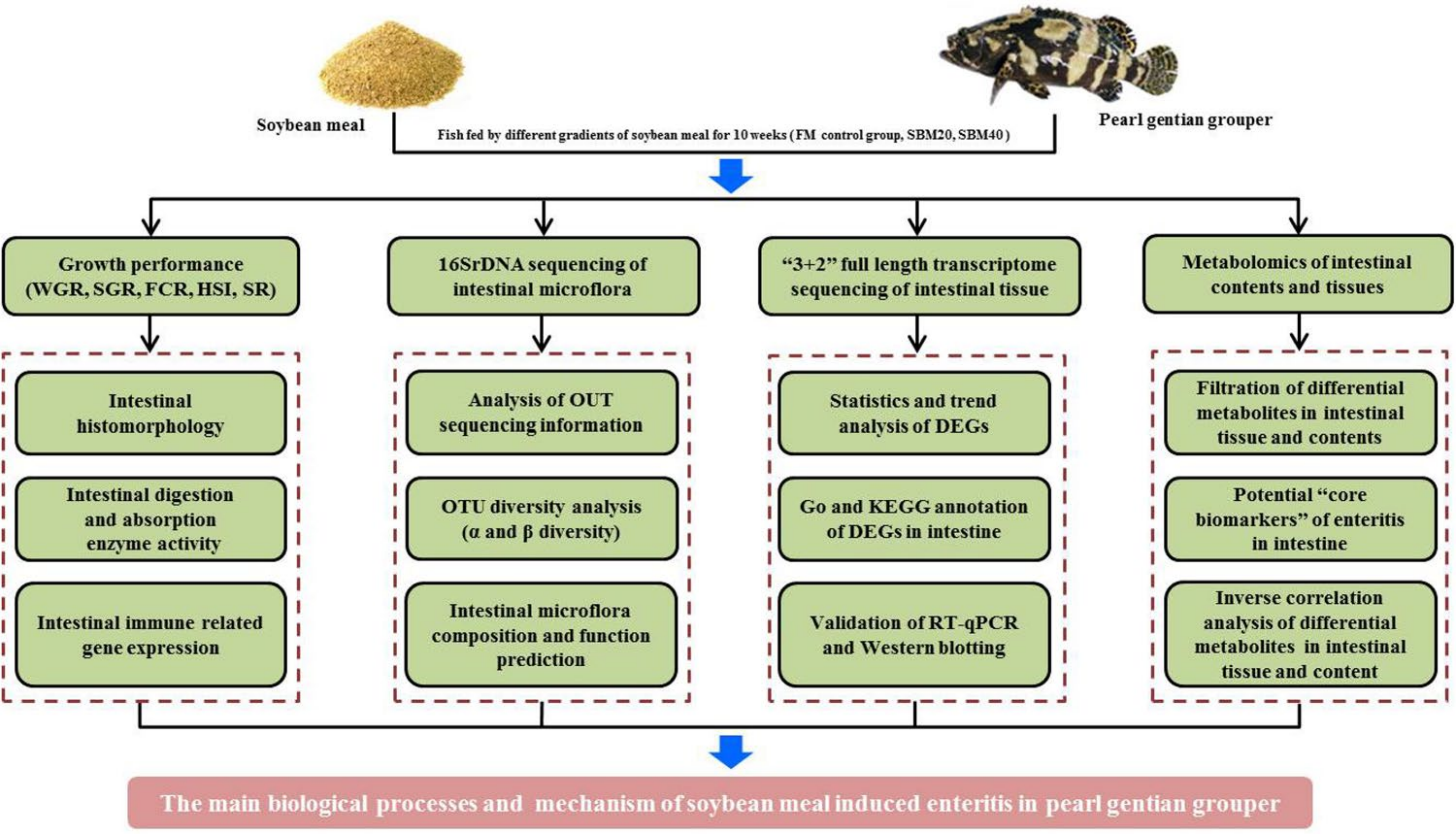
The brief flow chart of this experiment.

The protocol of this study was carried out from March 2019 to April 2020 and approved by the Expert Committee of Fisheries College of Guangdong Ocean University. All methods were carried out in accordance with relevant guidelines and regulations. Furthermore, the study was carried out in compliance with the ARRIVE guidelines (http://www.nc3rs.org.uk/page.asp?id=1357). Present study analyzed the main biological events in the intestine of pearl gentian grouper under the state of SBMIE. Three experimental groups were set up in this experiment, namely FM control group 20% SBM substitution for FM (SBM20) and SBM40 group. The main contents of this study were divided into four parts, including the effect of SBM on the growth and physiology of grouper; 16S high-throughput sequencing analysis of intestinal microflora; the "3 + 2" transcriptome evaluation of intestinal tissue; metabolomics of intestinal contents to analyze the potential "core biomarkers" of enteritis, and the inverse correlation analysis with intestinal metabolomics to explore the substance exchange between intestinal contents and intestinal tissues under the state of SBMIE in pearl gentian grouper (Fig. 19).

The healthy grouper juveniles (100% female) with an average body weight about 9 g were obtained from a commercial hatchery (Zhanjiang, China). When the juvenile grouper adapted to the culture environment, 720 healthy fish with similar size were randomly assigned to a 1000 L cylindrical fiberglass tank with 60 fish per tank and 4 replicates in each group (n = 4). Each group diet fed twice a day (8:00 and 16:00) using the corresponding experimental diets to apparent satiety level for 10 weeks. During the experimental period, the light cycle used the natural conditions, the temperature was 29 ± 1 °C, the ammonia and nitrate remained below 0.03 mg L^-1^, and the dissolved oxygen was not less than 7 mg L^-1^. In the first two weeks, 60% of the water of each tank was changed every day, and in the later period, all the water was changed every day. The detailed preparation process of the experimental diets and feeding trial are described in Supplementary file S2. Present study selected a relatively enough sample size because the main biological events in the intestinal tract of marine fish pearl gentian grouper in the state of SBMIE analyzed by multi-omics were evaluated in vivo for the first time, and the preliminary intention was to investigate the main evidence regarding the SBMIE characteristics of pearl gentian grouper in more complex experimental designs.

### Sample collecting and processing

During the process of experiment, if the fish was found to be sick, the sample was not used. There was no outbreak of disease in the process of experiment, so no samples were removed. For the analysis of transcriptome sequencing, n = 4; for the analysis of metabolomics, n = 12. For the analysis of the rest indexes in this research, the value of n was no less than 3, to satisfy the significance of biological statistics. Samples were collected at the end of the experiment. Before sampling, all fish in each tank were counted and weighed to determine weight gain (WGR), specific growth rate (SGR), feed conversion ratio (FCR), and survival rate (SR). All fish were anesthetized with eugenol (1:10,000) before sampling. Six fish were randomly taken from each tank and collected the blood from the caudal vein and stored at 4 °C overnight. After that, the blood was centrifuged at 15,000 rpm for 10 min to obtain the serum, and stored at -80 °C for enzyme analysis. Then, cutting abdomen along the midline and pulling out the intestine gently, and then cleared of any mesentery and adipose tissue, and wash off the external residue using deionized water. Some distal intestine (DI) and liver samples were put into liquid nitrogen immediately after being put into cryopreservation tube. After sampling, the samples were transferred to -80 °C for enzyme activity analysis. Some DI samples were cut into pieces and placed in the tube containing RNAlater. After being placed overnight at 4 °C, the samples were transferred to -80 °C for gene expression determination.

For 16S high-throughput sequencing, three fish were randomly taken from each tank, and removed the mesentery and adipose tissue. Then DI tissue was washed with deionized water to remove the residue. The DI samples were placed in the cryopreservation tube and immediately put into liquid nitrogen for intestinal microflora analysis.

For the “3 + 2” transcriptome sequencing, four fish were randomly taken from each tank and DI sample tissues were prepared according to the above method, then stored in liquid nitrogen.

For the metabolomics analysis, fish were fed normally and sampled at the last feeding time point. The abdomen of the fish was cut along the midline, and the complete intestine was removed. Samples were taken only from fish with digesta throughout the DI tract. 12 samples were taken from each treatment, and each sample was obtained by mixing the intestinal contents of three fish from the same tank. The feces were gently squeezed out of the DI tract, and only feces with intact capsule were collected. Then, the corresponding intestinal tissue samples were taken from each fish. In DI content samples, the FM control group, 20% SBM group and 40% SBM group named FM_N, SBM20_N and SBM40_N, respectively. Similarly, in DI tissue samples, the corresponding names are FM, SBM20 and SBM40, respectively. Finally, the cryopreservation tubes containing feces and DI tissues were immediately put into liquid nitrogen and transferred to -80 °C for metabolomics analysis.

### Histology

When feeding trial finished, three fish were randomly selected from each tank. The DI samples were divided into two parts, one part was placed in 4% paraformaldehyde universal tissue fixative (Servieobio Technology Co., Ltd., Wuhan, China) for 24 h until further treatment (HE and Tunnel staining)^43^; the other part was stored in electron microscope stationary solution (Servieobio Technology Co., Ltd., Wuhan, China) for transmission electron microscopy (TEM) analysis^124^. The histological evaluation of enteritis was according to semi quantitative method. The detailed scoring criteria of histological morphology of different indicators are shown in Supplementary Table 32. The scores were recorded by visual analog scoring method^40^.

### Physiological indexes

The following parameters were assessed, which including the enzyme activity of trypsin (Try) in DI tissue, and alanine aminotransferase (ALT) and aspartate aminotransferase (AST) in liver; the Immunoglobulin M (IgM), complement 3 (C3), complement 4 (C4) and malondialdehyde (MDA) concentrations in DI tissue were determined by fish ELISA Kit (Jianglai Biotechnology Co., Ltd., Shanghai, China). The expressions of pro-inflammatory related genes (*IL1β*, *IL8*, *IL17*, *TNF*α and *CSF1*) and anti-inflammatory related genes (*IL4*, *IL10*, *TGFβ1* and *Hepcidin*) in DI tissue were determined, and the primers of the genes were designed by Primer Premier 5.0 software (Supplementary Table 33). The internal reference gene is *β*-actin. All the detection methods were carried out according to the instructions.

### Multiomics determination

A detailed procedures were supplied in Supplementary file S2. Briefly, by 16S high-throughput sequencing, present study analyzed the OTU diversity of intestinal microflora with dietary SBM addition, including Alpha diversity indices, such as Observed species, Shannon, Simpson, Chao 1 and ACE; Beta diversity indices among groups, such as Principal coordinate analysis (PCoA). Furthermore, functional prediction of the intestinal microflora was predicted by FAPROTAX^125^. The original reads of intestinal microflora are stored in the NCBI sequential read Archive (SRA) database and the accession number is PRJNA666309.

By “3 + 2” PacBio SMRT full-length transcriptome sequencing, the differentially expressed genes (DEGs) were identified by DESeq R software package (https://cran.r-project.org/doc/FAQ/R-FAQ.html#Citing-R). Among them, the genes with |log2FC|> 1 and *P* < 0.05 were identified as DEGs. On the basis of identification of differential genes, the trend analysis of the DEGs in FM, SBM20 and SBM40 groups was carried out in this study. The DEGs genes with significant differences (*P* < 0.05) in trend analysis were annotated with GO and KEGG, and the signal pathways with significant differences related to Immune diseases/system, Infectious diseases and Signal transduction were further analyzed (*P* < 0.05). Then, the typical signaling pathway was validation of RT-qPCR and western blotting. The raw PacBio SMRT sequencing raw reads and Illumina sequencing raw reads are deposited in NCBI Sequence Read Archive (SRA) and the accession numbers are PRJNA664623 and PRJNA664416, respectively.

By Metabolomic determination, the co-contained differential metabolites in SBM20_N and SBM40_N group were considered as the potential “core biomarker” in DI content for SBMIE of pearl gentian grouper. Then, the inverse correlation analysis between metabolomics of DI contents and DI tissues were combined to analyze the co-contained metabolites with opposite trend and *P* < 0.05. These metabolites may exchange between DI contents and intestinal tissues and play an important role in the development of SBMIE.

### Statistics analyses

Growth performance was calculated by the following formulas:

$$ {\text{Weight}}\;{\text{gain}}\;{\text{rate}}\left( {{\text{WGR}},\% } \right) = {1}00 \times \left( {{\text{final}}\;{\text{body}}\;{\text{weight}}{-}{\text{initial}}\;{\text{body}}\;{\text{weight}}} \right)/{\text{initial}}\;{\text{body}}\;{\text{weight}}; $$

$$ {\text{Specific}}\;{\text{growth}}\;{\text{rate}}\left( {{\text{SGR}},\% /{\text{d}}} \right) = {1}00 \times \left[ {{\text{Ln}}\left( {{\text{finial}}\;{\text{body}}\;{\text{weight}}} \right) - {\text{Ln}}\left( {{\text{initial}}\;{\text{body}}\;{\text{weight}}} \right)} \right]/{\text{days}}; $$

$$ {\text{Feed}}\;{\text{conversion}}\;{\text{ratio}}\left( {{\text{FCR}}} \right) = {\text{feed}}\;{\text{intake}}/\left( {{\text{final}}\;{\text{body}}\;{\text{weight}}{-}{\text{initial}}\;{\text{weight}}} \right); $$

$$ {\text{Hepatosomatic}}\;{\text{index}}\left( {{\text{HSI}},\% } \right) = {1}00 \times ({\text{hepatic}}\;{\text{weight}}/{\text{body}}\;{\text{weight}}); $$

$$ {\text{Survival}}\;{\text{rate}}\left( {{\text{SR}},\% } \right) = {1}00 \times \left( {{\text{final}}\;{\text{fish}}\;{\text{number}}/{\text{initial}}\;{\text{fish}}\;{\text{number}}} \right). $$

$$ {\text{Statistical}}\;{\text{analysis}}\;{\text{was}}\;{\text{performed}}\;{\text{by}}\;{\text{SPSS}}\;{22}.0\left( {{\text{SPSS}}\;{\text{Inc}},{\text{Chicago}},{\text{IL}},{\text{USA}}} \right). $$

Results were expressed as mean ± standard deviation (‾x ± SD). To examine the differences between the groups, one-way ANOVA was conducted after homogeneity variance test. The significance threshold was *P* < 0.05. The statistics of 16S high-throughput sequencing, transcriptomics and metabolomics are described in the corresponding section mentioned above. The metabolites were analyzed by Receiver operator characteristic (ROC) to determine area under the curve (AUC) to compare the predictive ability of metabolites.

## Supplementary Information

Supplementary Figures.

Supplementary Files.

Supplementary Tables.
