# Supplementary material for: Multiomics analysis of soybean meal induced marine fish enteritis in juvenile pearl gentian grouper, Epinephelus fuscoguttatus ♀ × Epinephelus lanceolatus ♂
Source: Sci Rep. 2021 Dec 2;11:23319. doi: 10.1038/s41598-021-02278-z (PMC8640039; doi:10.1038/s41598-021-02278-z)
Supplement: Supplementary file 1 — Supplementary Figures. [file 41598_2021_2278_MOESM1_ESM.docx]

**Multiomics analysis of soybean meal induced marine fish enteritis in juvenile pearl gentian grouper,**

***Epinephelus fuscoguttatus ♀× Epinephelus lanceolatus ♂***

**Wei Zhang^1,2,3^, Beiping Tan^1,2,3,^*, Junming Deng^1,2,3^, Zhang Haitao^3^**

^1^*Laboratory of Aquatic Animal Nutrition and Feed,* *College of Fisheries, Guangdong Ocean University, Zhanjiang, Guangdong 524025, China*

^2^*Aquatic Animals Precision Nutrition and High Efficiency Feed Engineering Research Center of Guangdong Province, Zhanjiang, Guangdong 524025, China*

^3^*Key Laboratory of Aquatic, Livestock and Poultry Feed Science and Technology in South China, Ministry of Agriculture, Zhanjiang, Guangdong 524025, China*

**Supplementary Figures:**


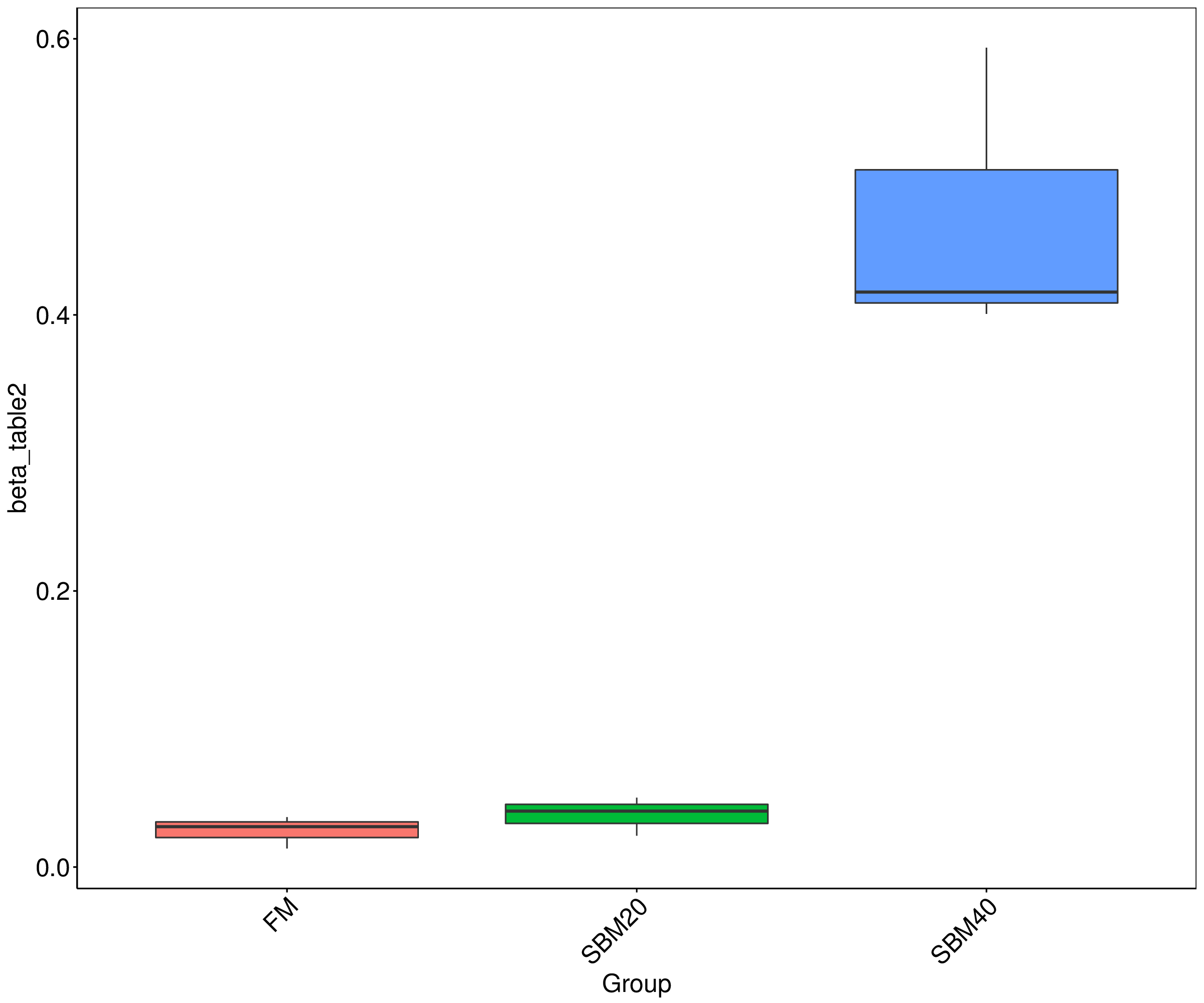


**Supplementary Figure 1.** The *β* diversity index differences of intestinal microflora of pearl gentian grouper fed by different levels of soybean meal diets (n=3)


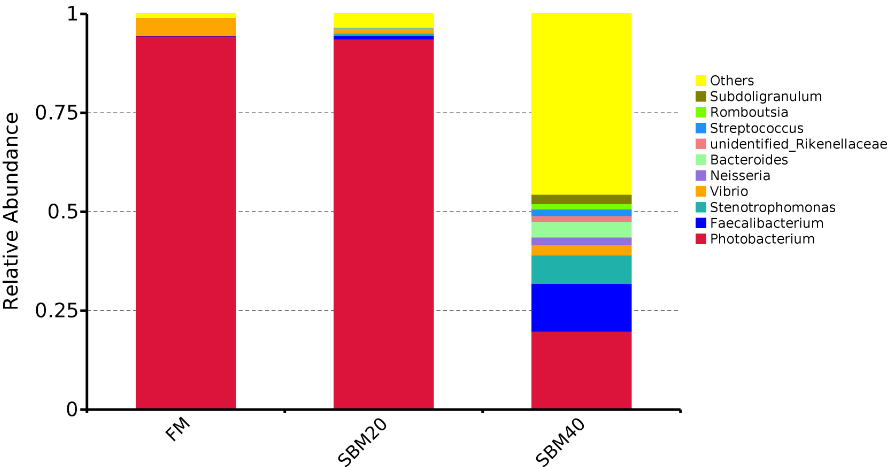


**Supplementary Figure 2.** The top 10 species of relative abundance of intestinal microflora at genus level in pearl gentian grouper fed by different soybean meal diets (n=3)


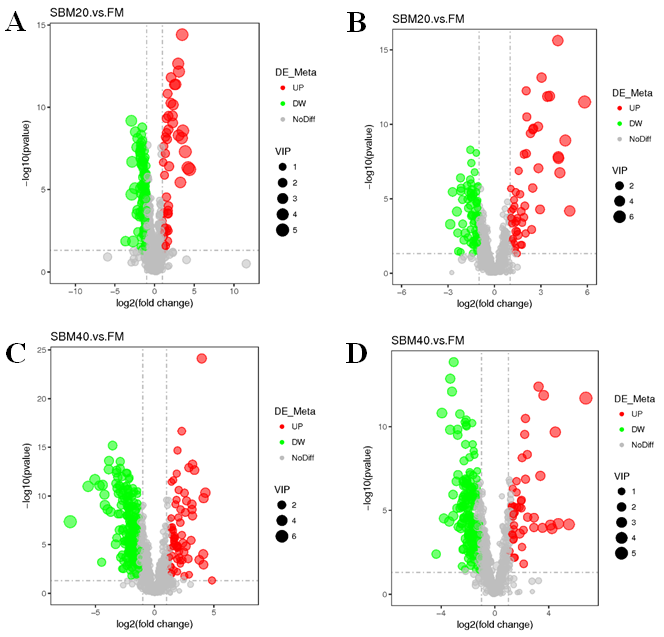


**Supplementary Figure 3.** Volcano plot of *P* values between FM, SBM20 and SBM40 groups of DI intestinal tissues in positive (A, C) and negative (B, D) modes (n=12).

Note: Each dot represents a metabolite. Red dots represent significantly up-regulated metabolites, green dots represent significantly down-regulated metabolites, and gray dots represent metabolites with no significant difference. The point size represents the VIP value. FM, fish meal control group; SBM20, 20% SBM protein replacement level to FM protein; SBM40, 40% SBM protein replacement level to FM protein.
