# Supplementary material for: Multiomics analysis of soybean meal induced marine fish enteritis in juvenile pearl gentian grouper, Epinephelus fuscoguttatus ♀ × Epinephelus lanceolatus ♂
Source: Sci Rep. 2021 Dec 2;11:23319. doi: 10.1038/s41598-021-02278-z (PMC8640039; doi:10.1038/s41598-021-02278-z)
Supplement: Supplementary file 2 — Supplementary Files. [file 41598_2021_2278_MOESM2_ESM.docx]

**Multiomics analysis of soybean meal induced marine fish enteritis in juvenile pearl gentian grouper,**

***Epinephelus fuscoguttatus ♀× Epinephelus lanceolatus ♂***

**Wei Zhang^1,2,3^, Beiping Tan^1,2,3,^*, Junming Deng^1,2,3^, Zhang Haitao^3^**

^1^*Laboratory of Aquatic Animal Nutrition and Feed,* *College of Fisheries, Guangdong Ocean University, Zhanjiang, Guangdong 524025, China*

^2^*Aquatic Animals Precision Nutrition and High Efficiency Feed Engineering Research Center of Guangdong Province, Zhanjiang, Guangdong 524025, China*

^3^*Key Laboratory of Aquatic, Livestock and Poultry Feed Science and Technology in South China, Ministry of Agriculture, Zhanjiang, Guangdong 524025, China*

**Supplementary file S1**

**Blinding**

For the whole experiment, four different investigators were involved as follows: a first investigator (T.B.P.) is responsible for the experimental design and supply of experimental feed, a first investigator (Z.W.) is responsible for the breeding experiment part, a first investigator (D.J.M.) is responsible for the sample detection and a first investigator (Z.H.T.) is responsible for the data analysis, with their own independence. The influence of subjective factors on the experimental results was avoided as far as possible.

**The detailed analysis results of** **UPLC-MS profile**

*Metabolic profile of diets and contents*

The representative spectra of DI contents and diets are displayed in Supplementary Fig.S1 and Supplementary Fig.S2. All DI content and diets samples were analyzed by UPLC-MS in positive and negative ion mo\des. The score plot of PCA in two modes was shown in Supplementary Fig.S3 and Supplementary Fig.S4. The QC quality control samples are located in the center and clustered closely, which indicates that the data quality is good and the model is stable and reliable. The correlation of QC samples was exhibited in Supplementary Fig.S5 and Supplementary Fig.S6.

Afterwards, the metabolites profiles were characterized by PLS-DA. The results of PLS-DA model for diets and DI contents in positive and negative ion modes were shown in Supplementary Fig.S7 and Supplementary Fig.S8. The separation between the two groups was clear, and the samples in each group were basically in 95% confidence ellipse. Subsequently, positive and negative ion modes were used for permutation test to prevent models over-fitting (Supplementary Fig.S9 and Supplementary Fig.S10). The parameters of permutation tests R2Y and Q2Y in FM and SBM20 diet samples were 1 and 0.97 in the positive ion mode and 1 and 0.98 in the negative ion mode, respectively; the R2Y and Q2Y in FM and SBM40 diet samples were 1.00 and 0.99 in the positive ion mode and 1.00 and 0.99 in the negative ion mode, respectively. Similarly, in DI content samples, the R2Y and Q2Y in FM_N and SBM20_N samples were 0.97 and 0.93 in the positive ion mode and 0.96 and 0.92 in the negative ion mode, respectively; the R2Y and Q2Y in FM_N and SBM40_N diet samples were 0.98 and 0.93 in the positive ion mode and 0.99 and 0.97 in the negative ion mode, respectively. All of the permutation test values were more than 0.5. The red line (Q2) and the blue line (R2Y) on the left were lower than the original point on the right, indicating a low risk of over-fitting these models.


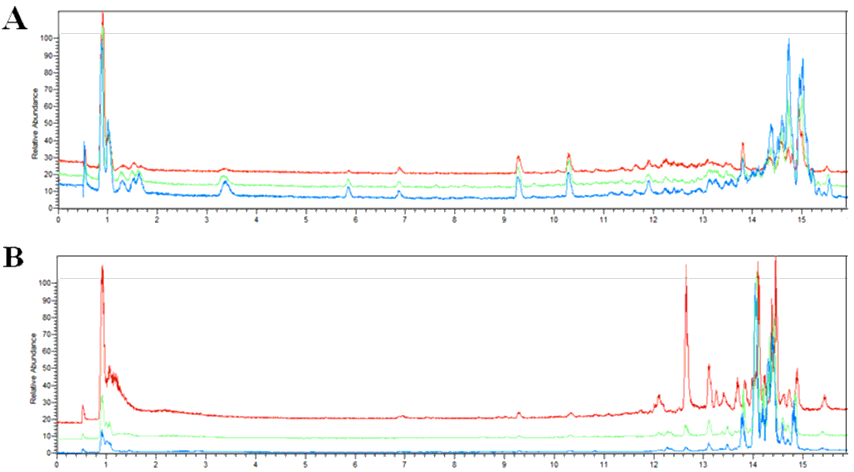


**Supplementary** **Fig. S1** The representative UPLC-MS spectra of the distal intestine contents

in positive (A) and negative modes (B).

Note: The red curve was the representative spectrum of FM group; the green curve was the representative spectrum of SBM20 group; the blue curve was the representative spectrum of SBM40 group. FM_N, fish meal control group; SBM20_N, 20% SBM protein replacement level to FM protein; SBM40_N, 40% SBM protein replacement level to FM protein.


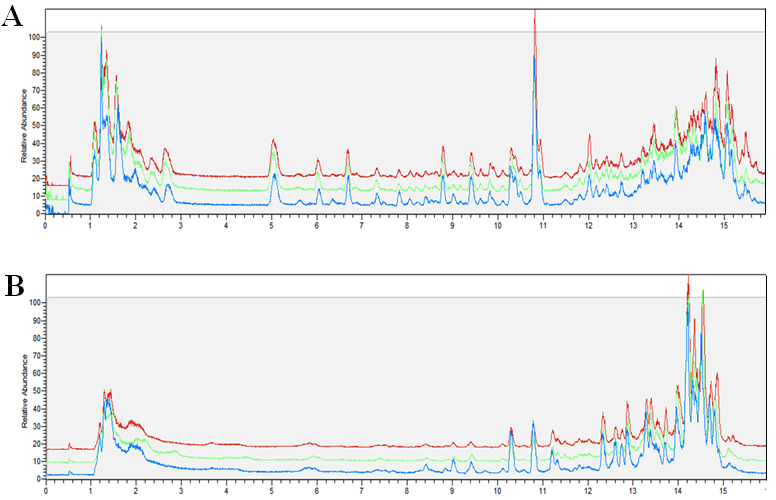


**Supplementary Fig. S2** The representative UPLC-MS spectra of the SBM diets

in positive (A) and negative modes (B).

Note: The red curve was the representative spectrum of FM group; the green curve was the representative spectrum of SBM20 group; the blue curve was the representative spectrum of SBM40 group. FM, fish meal control group; SBM20, 20% SBM protein replacement level to FM protein; SBM40, 40% SBM protein replacement level to FM protein.


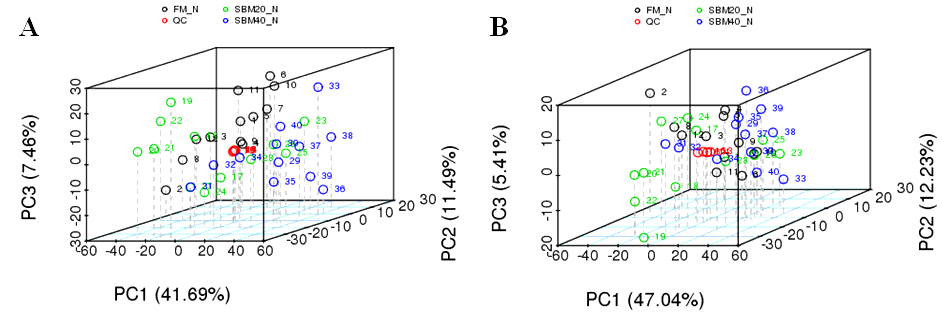


**Supplementary Fig. S3** The total sample PCA score plots resulting from UPLC-MS spectra of the distal intenstine contents in positive (A) and negative modes (B) (n=12).

Note: FM_N, fish meal control group; SBM20_N, 20% SBM protein replacement level to FM protein; SBM40_N, 40% SBM protein replacement level to FM protein. QC, quality control.


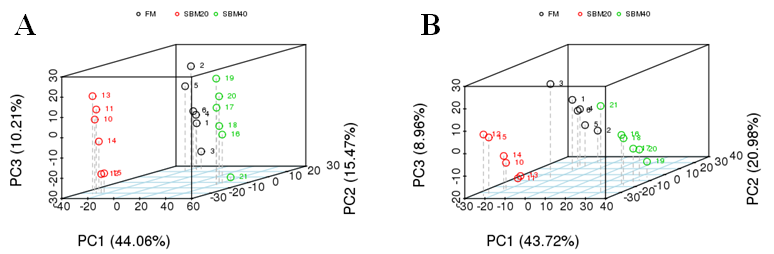


**Supplementary Fig. S4.** The total sample PCA score plots resulting from UPLC-MS spectra of the SBM diets contents in positive (A) and negative modes (B) (n=12).

Note: FM, fish meal control group; SBM20, 20% SBM protein replacement level to FM protein; SBM40, 40% SBM protein replacement level to FM protein. QC, quality control.


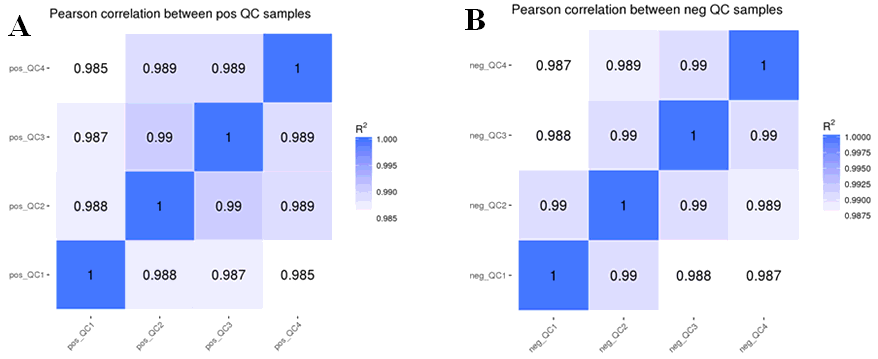


**Supplementary Fig. S5** The correlation of QC samples of distal intestine contents in positive (A) and negative modes (B) (n=12).

Note: Abscissa is log10(Peak. Area + 1), ordinate is log(Peak. Area + 1), R2 is the square of Pearson correlation coefficient.


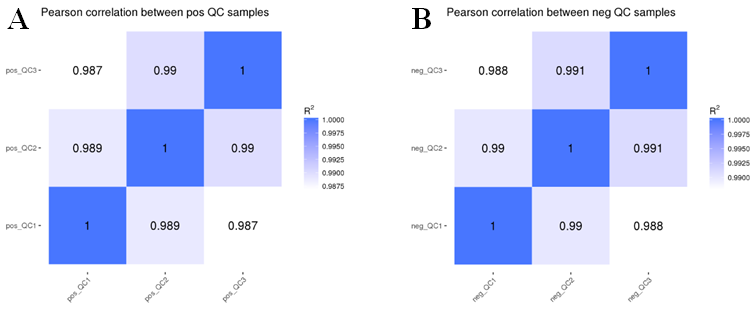


**Supplementary Fig. S6** The correlation of QC samples of distal intestine contents in positive (A) and negative modes (B) (n=12).

Note: Abscissa is log10(Peak. Area + 1), ordinate is log(Peak. Area + 1), R2 is the square of Pearson correlation coefficient.


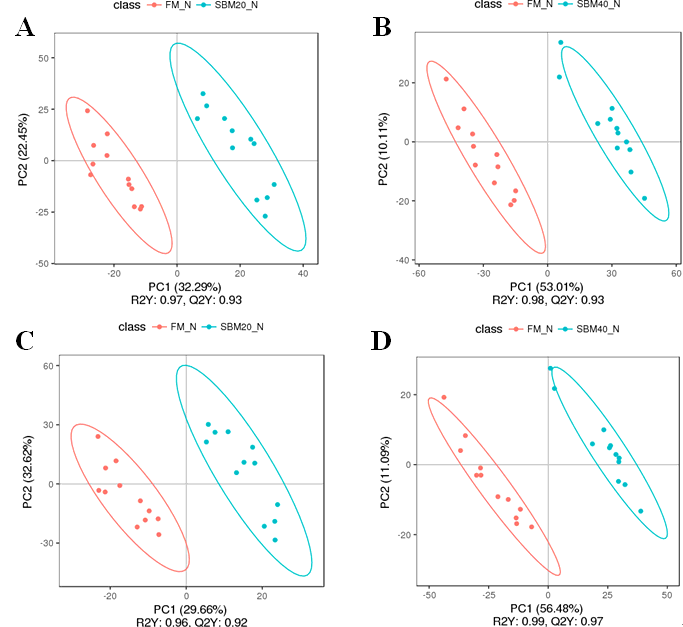


**Supplementary Fig. S7** The total sample PLS-DA score plots resulting from UPLC-MS spectra of distal intenstine contents in positive (A, C) and negative modes (B, D) (n=12).

Note: FM_N, fish meal control group; SBM20_N, 20% SBM protein replacement level to FM protein; SBM40_N, 40% SBM protein replacement level to FM protein.


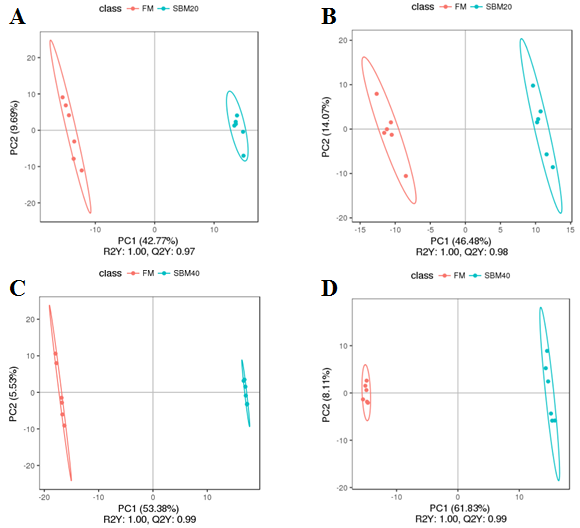


**Supplementary Fig. S8** The total sample PLS-DA score plots resulting from UPLC-MS spectra of distal intenstine contents in positive (A, C) and negative modes (B, D) (n=12).

Note: FM, fish meal control group; SBM20, 20% SBM protein replacement level to FM protein; SBM40, 40% SBM protein replacement level to FM protein.


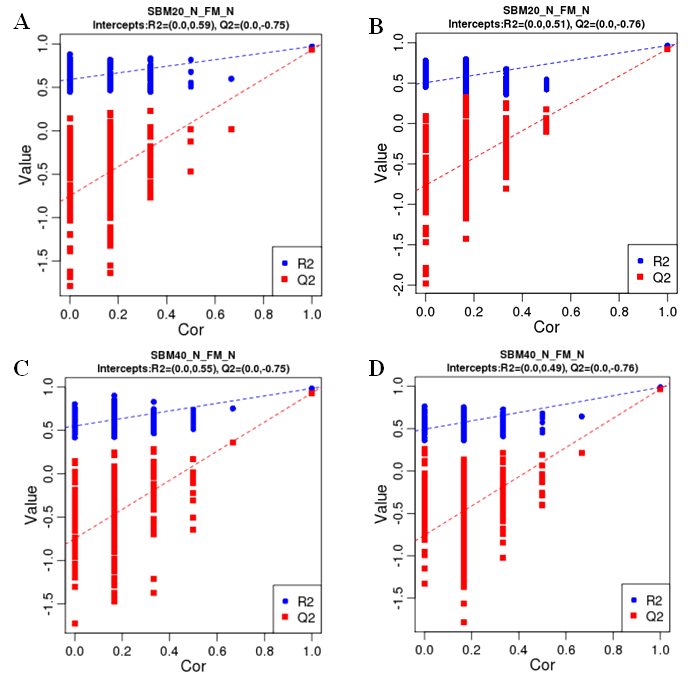


**Supplementary Fig. S9** Permutation test result of the PLS-DA models of distal intestine contents after 10 weeks treatment in the positive (A, C) and negative (B, D) modes (n=12).

Note: The R2Y value represents the goodness of fit of the model. The Q2 value indicates the predictability of the model. FM_N, fish meal control group; SBM20_N, 20% SBM protein replacement level to FM protein; SBM40_N, 40% SBM protein replacement level to FM protein.


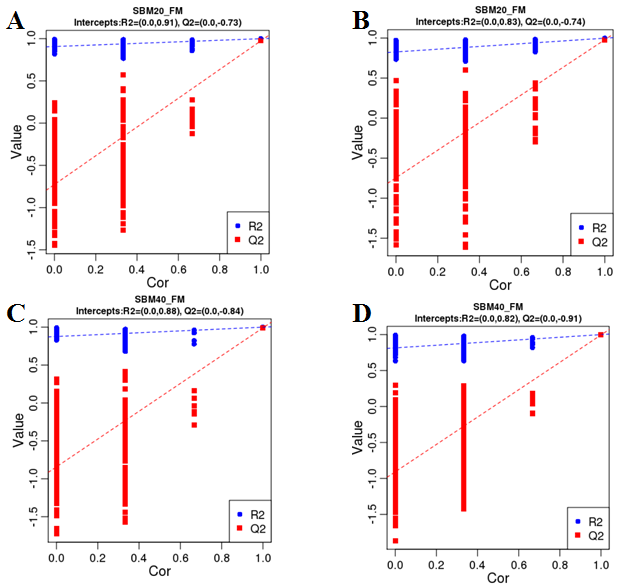


**Supplementary** **Fig.** **S10** Permutation test result of the PLS-DA models of SBM diets after 10 weeks treatment in the positive (A, C) and negative (B, D) modes (n=12).

Note: The R2Y value represents the goodness of fit of the model. The Q2 value indicates the predictability of the model. FM, fish meal control group; SBM20, 20% SBM protein replacement level to FM protein; SBM40, 40% SBM protein replacement level to FM protein.

*Metabolic profile of intestine tissues*

In order to explore the metabolite exchanges between DI contents and intestinal tissues in the state of enteritis, the UPLC-MS spectra of intestinal tissues was also performed. The representative spectra of intestinal tissue were shown in Supplementary Fig.S11. The PCA score plots of intestinal tissue samples were displayed in Supplementary Fig.S12. The QC quality control samples are located in the center and clustered closely, which indicates that the data quality is good and the model is stable and reliable. The correlation of QC samples was exhibited in Supplementary Fig.S13.

Similarly, the metabolites profiles were characterized by PLS-DA. The results of PLS-DA model for intestinal tissues in positive and negative ion modes were shown in Supplementary Fig.S14. The separation between the two groups was clear, and the samples in each group were basically in 95% confidence ellipse. Subsequently, positive and negative ion modes were used for permutation test to prevent models over-fitting (Supplementary Fig.S15). The parameters of permutation tests R2Y and Q2Y in FM and SBM20 intestinal tissue samples were 0.97 and 0.90 in the positive ion mode and 0.98 and 0.93 in the negative ion mode, respectively; the R2Y and Q2Y in FM and SBM40 diet samples were 0.99 and 0.97 in the positive ion mode and 0.99 and 0.96 in the negative ion mode, respectively. All of the permutation test values were more than 0.5. The red line (Q2) and the blue line (R2Y) on the left were lower than the original point on the right, indicating a low risk of over-fitting these models.


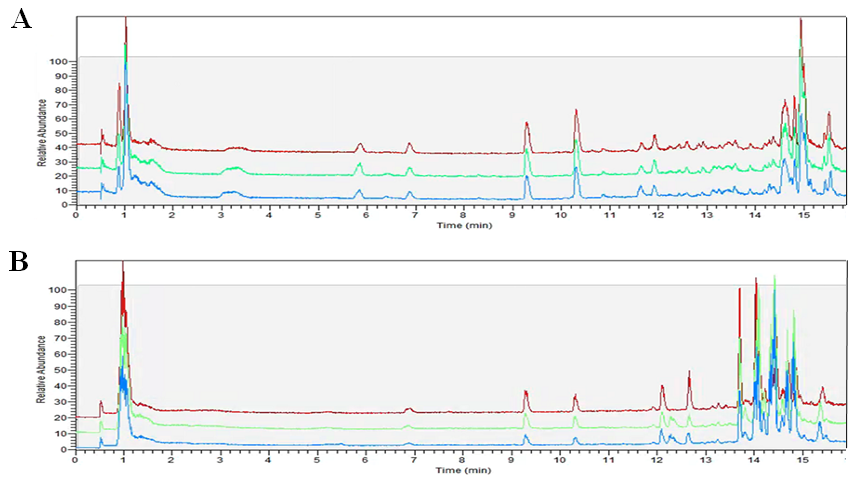


**Supplementary Fig. S11** The representative UPLC-MS spectra of the distal intestine tissues in positive (A) and negative modes (B)

Note: The red curve was the representative spectrum of FM group; the green curve was the representative spectrum of SBM20 group; the blue curve was the representative spectrum of SBM40 group. FM, fish meal control group; SBM20, 20% SBM protein replacement level to FM protein; SBM40, 40% SBM protein replacement level to FM protein.

*
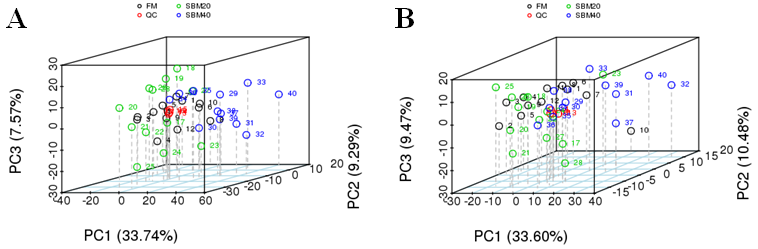
*

**Supplementary Fig. S12** The total sample PCA score plots resulting from UPLC-MS spectra of the distal intenstine tissues in positive (A) and negative modes (B) (n=12).

Note: FM, fish meal control group; SBM20, 20% SBM protein replacement level to FM protein; SBM40, 40% SBM protein replacement level to FM protein. QC, quality control.


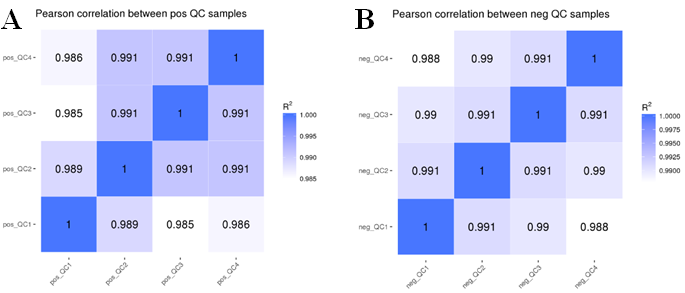


**Supplementary Fig. S13** The correlation of QC samples of distal intestine tissues in positive (A) and negative modes (B)

Note: Abscissa is log10(Peak. Area + 1), ordinate is log(Peak. Area + 1), R2 is the square of Pearson correlation coefficient.


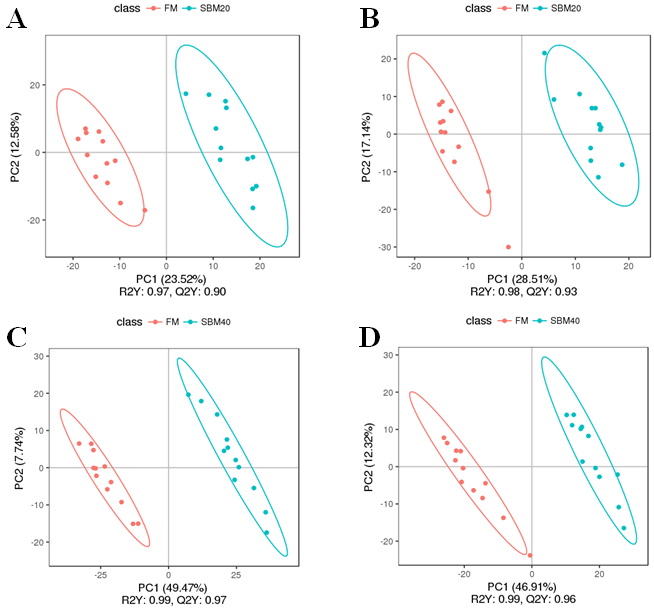


**Supplementary Fig. S14** The total sample PLS-DA score plots resulting from the UPLC-MS spectra of distal intenstine tissues in positive (A,C) and negative modes (B,D) (n=12).

Note: FM, fish meal control group; SBM20, 20% SBM protein replacement level to FM protein; SBM40, 40% SBM protein replacement level to FM protein.


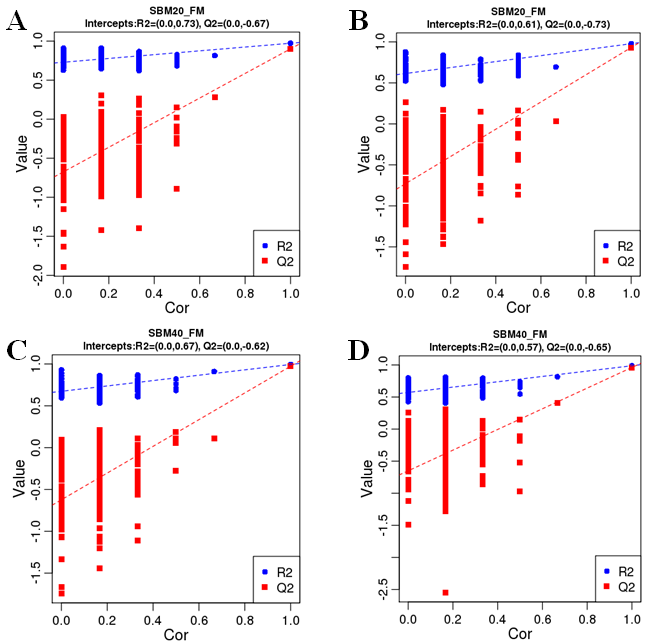


**Supplementary Fig. S15** Permutation test result of the PLS-DA models of DI intestinal tissues after 10 weeks treatment in the positive (A, C) and negative (B, D) modes (n=12).

Note: The R2Y value represents the goodness of fit of the model. The Q2 value indicates the predictability of the model. FM, fish meal control group; SBM20, 20% SBM protein replacement level to FM protein; SBM40, 40% SBM protein replacement level to FM protein.

**Supplementary file S2**

**The detailed procedures of this experiment**

**The preparation process and storage condition of the diets**

The composition and chemical analysis of the basic experimental diet is presented in Supplementary Table S1. The red FM (72.53% crude protein and 8.82% total lipid on a dry matter basis) used in this study was supplied by Corporación Pesquera Inca S.A.C., Bayovar Plant, Peru. The SBM in this study was supplied by Zhanjiang Haibao Feed Co. Ltd. (Zhanjiang, China; 48.92% crude protein on dry matter basis). Three iso-nitrogenous (approximately 50% crude protein) and iso-lipidic (10% total lipid) experimental diets were formulated to replace 0, 20% and 40% of FM protein by a corresponding amount of SBM protein to form the experimental diets named FM (control), SBM20 and SBM40, respectively. Lysine and methionine were added to experimental feed to compensate for imbalance. Ingredients were ground into fine powder and sieved through 60-mesh size and weigh accurately according to the formula. The micro constituents were mixed homogenously by sequential expansion method. Then, deionized water and the lipids were added and thoroughly mixed to obtain a homogenous mixture. Subsequently, the dough was passed through the pelletizer with 2.0 and 3.0 mm diameter. The pellets were air-dried to 10% moisture and placed in plastic bags and stored at -20^o^C until used. The detection of essential amino acid in the diets was supplied in Supplementary Table S2.

**Supplementary Table S1. Formulation and proximate composition of the experimental diets (%, dry matter)**

| Ingredients (%) | Diets | | |
| --- | --- | --- | --- |
|  | FM | SBM20 | SBM40 |
| Red fish meal | 50.00 | 40.00 | 30.00 |
| Soybean meal | 0.00 | 14.83 | 29.65 |
| Vital wheat gluten | 5.00 | 5.00 | 5.00 |
| Wheat flour | 18.00 | 18.00 | 18.00 |
| Casein | 4.60 | 4.60 | 4.60 |
| Gelatin | 1.00 | 1.00 | 1.00 |
| Fish oil | 3.02 | 3.75 | 4.48 |
| Soybean oil | 2.00 | 2.00 | 2.00 |
| Soybean lecithin | 2.00 | 2.00 | 2.00 |
| Microcrystalline cellulose | 11.48 | 5.74 | 0.00 |
| Calcium monophosphate | 1.50 | 1.50 | 1.50 |
| Ascorbic acid | 0.05 | 0.05 | 0.05 |
| Choline chloride | 0.50 | 0.50 | 0.50 |
| Vitamin premix^a^ | 0.30 | 0.30 | 0.30 |
| Mineral premix^b^ | 0.50 | 0.50 | 0.50 |
| Ethoxyquin | 0.05 | 0.05 | 0.05 |
| Lysine^c^ | 0.00 | 0.12 | 0.24 |
| Methionine^c^ | 0.00 | 0.06 | 0.13 |
| Proximate composition) (%, dry matter) | | | |
| Crude protein | 50.97 | 50.56 | 50.85 |
| Crude lipid | 10.15 | 10.50 | 10.44 |

**Supplementary Table S2. The content of 17 amino acids in diets (%)**

| Amino acids | Diets | | |
| --- | --- | --- | --- |
|  | FM | SBM20 | SBM40 |
| Lysine | 3.02 | 2.93 | 2.88 |
| Methionine | 1.01 | 0.95 | 0.86 |
| Arginine | 2.53 | 2.43 | 2.47 |
| Threonine | 1.78 | 1.75 | 1.73 |
| Isoleucine | 1.86 | 1.78 | 1.82 |
| Histidine | 1.28 | 1.27 | 1.20 |
| Valine | 2.10 | 2.00 | 1.98 |
| Leucine | 3.28 | 3.16 | 3.20 |
| Phenylalanine | 1.94 | 1.88 | 1.96 |
| Tyrosine | 1.47 | 1.38 | 1.43 |
| Aspartate | 3.86 | 3.78 | 3.91 |
| Serine | 1.89 | 1.87 | 1.97 |
| Glutamate | 8.17 | 7.83 | 8.33 |
| Glycine | 2.48 | 2.42 | 2.29 |
| Alanine | 2.37 | 2.33 | 2.20 |
| Proline | 2.78 | 2.61 | 2.82 |
| Cystine | 0.49 | 0.49 | 0.51 |

**The detailed feeding trial and culture conditions**

Healthy grouper juveniles (100% female) with an average body weight about 9 g were obtained from a commercial hatchery (Zhanjiang, China). Upon arrival, the fish were acclimated to experimental condition for one week while feeding with commercial diet (Haida Aquatic Feed Co. Ltd., Zhanjiang, China). Before experiment, the fish were fasted for 24 h and grouped after anesthesia by eugenol (1:10000). The similar size of fish was randomly distributed into 1,000 L cylindrical fiberglass tanks and each tank has 60 fish. Each experimental diet was fed to four replicates twice daily at 8:00 and 16:00 until apparent satiation level for ten weeks. The feed consumption was recorded as described by our previous method. The experiment was conducted at the indoor farming systems of Marine Biological Research Base, Zhanjiang, China. All tanks were provided with continuous aeration through air stones. During the experimental period, the light cycle used the natural conditions, the temperature was 29 ± 1^o^C, the ammonia and nitrate remained below 0.03 mg L^-1^, and the dissolved oxygen was not less than 7 mg L^-1^. In the first two weeks, 60% of the water of each tank was changed every day, and in the later period, all the water was changed every day.

**Expression of intestinal immune related genes**

Total RNA of pearl gentian grouper DI tissue was extracted using Trizol Kit (Invitrogen, USA) according to the instructions. The RNA integrity was checked by 1% agarose gel. The qualified RNA samples were prepared with Evo M-MLV reverse transcription Kit (Takara, Japan), and then the cDNA prepared was stored at -20^o^C for use. The primers were designed by using Primer Premier 5.0 software. The primers designed in this experiment were synthesized by Shanghai Shenggong Bioengineering Co., Ltd. (Shanghai, China), and the template sequence was obtained from the third generation full-length transcriptome sequence database of the DI tissue of pearl gentian grouper. The primers of pro-inflammatory (*IL1β*, *IL8*, *IL17*, *TNF*α and *CSF1*) and anti-inflammatory genes (*IL4*, *IL10*, *TGFβ1* and *Hepcidin*) were designed (Supplementary Table 33), respectively. The internal reference gene is *β*-actin. The expression levels of these genes were detected by qRT-PCR (Mastercycler ep realplex，Eppendorf，Germany). The PCR reaction conditions were as follows: 95 ^o^C for 2 min, 1 cycle; 95 ^o^C for 15 s, 60 ^o^C for 10 s, 72 ^o^C for 20 s, 40 cycles. The expression of the target genes were determined by 2^-ΔΔ T^ method.

**16S analysis of intestinal microflora**

*Genome DNA extraction and amplification*

Total RNA of pearl gentian grouper DI microflora was extracted by using E.Z.N.A.^TM^ Kit (Omega Bio-Tek, Norcross, GA, U.S.) according to the instructions. The concentration and purity testing of extracted genomic DNA were tested by using 1% agarose gel. Then the DNA was diluted to 1 ng/μL using sterile water according to its concentration. The above DNA templates were amplified by primers 338F: ACTCCTACGGGAGGCAGCA and 806R: GGACTACHVGGGTATCTAAT of V3-V4 region of bacterial16SrDNA gene. All PCR reactions were carried out in 30 μL reaction system, using 15 μL Phusion® High-Fidelity PCR Master Mix（New England Biolabs), 0.2 μm forward and reverse primers, and about 10 ng template DNA. The PCR reaction conditions were as follows: pre-denaturation at 98 ^o^C for 1 min, denaturation at 98 ^o^C for 10 s, annealing at 50 ^o^C for 30 s, extension at 72 ^o^C for 30 s, 30 cycles, and extension was at 72 ^o^C for 5 min.

*PCR product purification*

The PCR product was mixed with an equal volume of 1 x loading buffer (containing SYB green) and detected by 2% agarose gel electrophoresis. The sample was mixed equally according to the concentration of PCR product. Then, purification was performed using the Gene JET^TM^ Gel Ectraction Kit (Thermo Scientific).

*Library construction and sequencing*

According to the operation instructions, the library was constructed with Ion Plus Fragment Library Kit 48 rxns Kit (Thermo Scientific) . After quantification and detection by Qubit2.0 Fluorometer Kit (Thermo Scientific), the library was sequenced using IonS5^TM^XL platform and 400 bp/600 bp single-end were generated. The original reads are stored in the NCBI sequential read Archive (SRA) database and the accession number is PRJNA666309.

*Data processing and analysis*

Exporting IonS5TMXL offline data to fastq file. The data of each sample is distinguished according to the barcode sequence. In order to obtain high-quality and effective data (clean reads), Cutadapt software (V1.9.1, http://cutadapt.readthedocs.io/en/stable/) is used to filter the raw data. Subsequently, the preliminary filtered reads were compared to the Silva database using UCHIME algorithm (UCHIME Algorithm, http://www.drive5.com/usearch/manual/uchime_algo.html) to detect the chimeric sequence, and then the chimeric filtering is carried out to obtain the effective data that can be used for subsequent analysis, i.e. clean reads. After that, Uparse software (http://drive5.com/uparse/) was used to cluster all clean reads of all samples, and the sequences with similarity ≥ 97% were clustered into the same Operational Taxonomic Units(OTUs). Finally, Mothur algorithm (version 1.39.1, http://www.mothur.org/) and Silva database (https://www.arb-silva.de/) were used for species annotation analysis of OTUs sequences. Intra group sample complexity analysis was performed using Alpha diversity indices such as Observed species, Shannon, Simpson, Chao 1 and ACE. Multi sample comparative analysis of Beta diversity among groups was carried out by Principal coordinate analysis (PCoA).

*Functional prediction*

The functions of the intestinal microflora were predicted by FAPROTAX. FAPROTAX database integrates the prokaryotic function database of many published culturable bacteria articles. The database contains more than 7600 functional annotation information of more than 4600 species, and contains more than 80 functional classifications such as carbon, nitrogen, phosphorus, sulfur cycle, animal and plant pathogens, methane generation, fermentation, etc. The detailed procedures of sequencing and analysis were carried out by Gene Denovo Co., Ltd. (Guangzhou, China).

**Transcriptome analysis**

*RNA extraction and detection*

Total RNA of pearl gentian grouper DI tissue was extracted using Trizol Kit (Invitrogen, USA) following manufacturer's recommendations. First, genome DNA was removed using DnaseI (Invitrogen, USA). Then, 1% agarose gel electrophoresis was used to analyze RNA degradation degree and whether there was contamination. Subsequently, Nanodrop 2000 (Thermo Fisher Scientific, USA) was used to detect RNA purity (OD260/280 ratio). Finally, Qubit was used to accurately quantify RNA concentration, and Agilent 2000 (Agilent Technologies, USA) was used to accurately test RNA integrity.

*Library construction*

(1) Third-generation library construction and sequencing

The mRNA was enriched by the magnetic beads which contain Oligo (dT) (Tiangen, China). The mRNA was reversed transcription the mRNA into cDNA using SMARTer PCR cDNA Synthesis Kit. PCR was used to amplify and enrich the synthesized cDNA, and the optimal PCR conditions were determined by cycle optimization. Partial cDNA was screened by BluePippin and enriched with more than 4 kb fragments, and large scale PCR was carried out to obtain enough total amount of cDNA. the full-length cDNA was used for damage repair, end repair, and connection of SMRT dumbbell shaped connector, and the equimolar library of non-screened fragments and fragments larger than 4kb was constructed; exonuclease digestion was used to remove the sequence of unconnected junctions at both ends of cDNA; finally, a complete SMRT bell library was constructed by binding primers and DNA polymerase. After passing the library inspection, the library was sequenced by PacBio Sequel platform according to the effective concentration of the library and data output requirements. The raw PacBio SMRT sequencing raw reads and Illumina sequencing raw reads are deposited in NCBI Sequence Read Archive (SRA) and the accession numbers are PRJNA664623 and PRJNA664416, respectively.

(2) Second-generation library construction and sequencing

After total RNA was extracted, the mRNA was enriched by Oligo(dT) beads, while the mRNA was enriched by removing rRNA by Ribo-Zero^TM^ Magnetic Kit (Epicentre). Then, the enriched mRNA was fragmented into short fragments using fragmentation buffer and reverse transcripted into cDNA with random primers. Second-strand cDNA was synthesized by DNA polymerase I, RNase H, dNTP and buffer. Then the cDNA fragments were purified with QiaQuick PCR extraction kit, end repaired, poly(A) added, and ligated to Illumina sequencing adapters. The ligation products were size selected by agarose gel electrophoresis, PCR amplified, and sequenced using Illumina HiSeq^TM^ 4000 by Gene Denovo Co., Ltd. (Guangzhou, China).

*PacBio SMART data processing*

After the sequence completing, the offline raw data are de spliced and read with low quality. The output is filtered and processed by the software SMRTlink V5.1. The parameters are: - minlength = 200, - minreadscore = 0.65, and then the final data is the valid data. In order to obtain the full-length transcripts, first, the subreads sequence was self-corrected to form CCS (parameter: - minpasses = 2, minpredicted accuracy = 0.8), and high quality transcript consistent sequence was obtained. The non-chimeric sequence with 5 'primer, 3' primer and PolyA tail is called full-length non-chimeric sequence (FLNC). IEC algorithm was used to cluster the FLNC sequences of the same transcript to obtain CCS, and then non full length sequences were used to correct the CCS. Then, the fused consensus sequences (CS) were obtained for subsequent analysis. After that, the Illumina RNA sequencing data were used to correct the polished consensus sequence by LoRDEC software (parameters: -k21, -s3) to further improve the accuracy of sequencing. Finally, CD-HIT-v4.6.7 (-c0.95 -T6 -G0 -aL0.00 -aS0.99) software was used to cluster and compare protein or nucleic acid sequences by sequence alignment, and remove redundant and similar sequences.

*Analysis of differentially expressed genes (DEGs)*

Clean reads obtained from Illumina sequencing were mapped to SMART sequence library by hisat2v2.05 software. RSEM software was used to calculate the gene expression level of each sample. The read count of each gene was obtained from the comparison results and converted into FPKM value. The DEGs were identified by DESeq R software package. Among them, the genes with |log2FC|＞1 and *P* < 0.05 were identified as DEGs. On the basis of identification of differential genes, the trend analysis of the DEGs in FM, SBM20 and SBM40 groups was carried out in this study. The DEGs genes with significant differences (*P* < 0.05) in trend analysis were annotated with GO and KEGG, and the signal pathways with significant differences related to Immune diseases/system, Infectious diseases and Signal transduction were further analyzed (*P* < 0.05).

*Validation of real-time quantitative PCR*

In order to verify the accuracy of RNA-seq results, RNA samples stored at -80 ^o^C for transcriptome sequencing were selected for RT-qPCR analysis. In this study, 14 genes related to inflammation were selected, which including *TLR1*, *TLR2*, *TLR3*, *TLR5*, *TLR8*, *TLR9*, *TLR13*, *TLR21*, *TLR22*, *MyD88*, *IKKα*, *IKKβ*, *IκBα* and *p65*. The primer design, synthesis and sequence source of all the genes are the same as those mentioned in section 2.7. The internal control gene is *β-actin* (Supplementary Table 34). The expression levels of these genes were detected by RT-qPCR. The PCR reaction conditions were 95 ^o^C for 2 min, 1 cycle; followed by 40 cycles of 95 ^o^C for 15 s, 60 ^o^C annealing for 10 s, and 72 ^o^C for 20 s. All reactions were done in triplicates. Melting curve analysis was performed to determine the target specificity. The RT-qPCR data were calculated using the 2^-ΔΔCT^ method.

*Western blotting*

The total protein of intestinal tissue was extracted according to the procedure of piercetm BCA protein assay Kit (Thermo Scientific, USA). The nuclear protein of intestinal tissue was extracted by NE-PER Nuclear and Cytoplasmic Ectraction Reagents Kit (Thermo Scientific，USA) according to the instructions. The concentration of the protein was determined by Bradford Protein Assay Kit (Beyotime, China). Preparation of gel in advance, 15 μL sample and 2 μL marker was added into per hole, respectively. Concentrated gel 90 V, 30 min; separation gel 110 V, 90 min. After the running gel, the protein in the gel is transferred to the PVDF film (Millipore, USA). Closed for 1 hour with TBST containing 5% fetal bovine serum, and then washed three times with TBST, each time for 10 min. The PVDF film and the first antibody were incubated overnight in a 4 ^o^C refrigerator. After incubation, the PVDF membrane was washed three times with TBST, each time for 5 min. Then PVDF film was incubated with horseradish peroxidase (HRP)-labeled secondary antibody for 1 hour. After incubation, the PVDF film was washed three times with TBST three times, each time for 10 min. Finally, a chemiluminescent imaging system (Shanghai Tianneng, China) was used to develop and preserve the strips for subsequent analysis.

**Metabolomic analysis**

*Metabolite extraction and* *UPLC-MS analysis*

In order to detect differential metabolites, FM, SBM20 and SBM40 groups of the diet samples were taken at different time points. In each group, 10 g of diet samples were taken at 1w, 2w, 4w, 6w, 8w and 10w, respectively. There were six replicates in each group. Then, the samples were stored at -80 ^o^C for LC-MS/MS analysis. The diet samples of FM control group, 20% SBM protein replacement group and 40% SBM protein replacement group were named FM, SBM20 and SBM40, respectively.

Diet, DI contents and DI tissue samples were performed in the same way. First, the samples were fully ground in liquid nitrogen and resuspended with 80% methanol and 0.1% formic acid. The samples were then incubated on ice for 5 min and centrifuged at 15000 rpm and 4 ^o^C for 5 min. Then, part of the supernatant was diluted with LC-MS water to the final concentration containing 60% methanol. Subsequently, the sample was then transferred to a fresh Eppendorf tube with a 0.22 μM filter and centrifuged at 15000 g and 4 ^o^C for 10 min. Finally, the filtrate was injected into the LC-MS/MS system for analysis.

*Quality control*

In order to control sample quality, quality control (QC) samples were prepared at the same time while sample processing. QC sample is a mixture of the same amount of experimental samples, which is used to balance the performance of LC-MS system, monitor the performance of the system, and evaluate the stability of the system in the whole experiment process. The higher the correlation of QC samples (the closer to 1), the better the stability of the whole method. What is reflected in the PCA diagram is that the distribution of QC samples will gather together. Meanwhile, a blank sample was established to remove background ions.

*Data processing and metabolite identification*

The raw data of UPLC-MS/MS were processed with Compound Discoverer 3.0 (CD 3.0, Thermo Fisher), and the peaks were aligned, extracted and quantified. The main parameters are as follows: retention time tolerance is 0.1 min; actual mass tolerance is 5ppm; signal intensity tolerance is 30%; signal/noise ratio is 3; minimum intensity is 100000. On this basis, the peak intensity is normalized to the total spectral intensity. The normalized data were used to predict the molecular formula according to additive ions, molecular ion peaks and fragment ions. Compare the peak value with the database *mz*Cloud (https://www.mzcloud.org/) and ChemSpider (<http://www.chemspider.com/>) to obtain accurate qualitative and relative quantitative results. Then, the selected data is imported into EZinfo software (version 2.0; Umetrics AB, Umeå, Sweden) for multivariate statistical analysis, such as principal component analysis (PCA) and partial least-squares discrimination analysis (PLS-DA). Before multivariate statistical analysis, the data were processed by Pareto scale, and PCA analysis was used to evaluate the reliability of data sets (including QC samples), and provide the visualized overall separation results of all samples. In order to distinguish different populations better, PLS-DA model was established for analysis. In addition, a 7-fold cross-validation and testing with 200 random permutations were performed by SIMCA-P+14.0 software (Umetrics AB, Umea, Sweden) in order to avoid over-fitting of supervised PLS-DA model. Then, Variable Importance in the Projection (VIP) value of PLS-DA model and *P* value of T-test were used to find differential metabolites between control and experimental groups. The VIP value reflects the contribution of each variable to the model. The metabolites with VIP > 1 and *P* value < 0.05 and fold change≥2 or FC≤0.5 were considered to be the differential metabolites. The metabolites in the diets, DI contents and DI tissues were analyzed and identified in the same way, respectively.

*Filtration of the differential metabolites in DI contents*

The Venn plot of the differential components between diets and intestinal contents was made to search for the co-contained differential substances, i.e. the overlaps in Venn plot. The log2FC values of differential components of diets and DI contents in the overlapping areas were calculated, respectively, and the co-contained differential metabolites changed with contrary trends were considered to be significantly affected before and after ingestion by fish, and the left of the co-cotained metabolites with same trend and differential times of Log2FC < 2 were considered to be no-significantly changed and removed in the whole differential metabolites of DI contents. Then, the left differential metabolites in the DI contents were considered to be altered by fish digestion rather than dietary concentration differences. After that, volcano plots were used to filter metabolites of interest which based on log2(FC) and -log10(*P*-value) of the metabolites. The *z*-Score plot was used to calculate the relative contents of metabolites on the same level. The cor.mtest() function in R packag was used to calculate the synergistic or mutex relationship among differential metabolites, and the threshold level of significant correlation was *P* < 0.05.

*Analysis of potential “core biomarkers” of enteritis*

After filtration of the differential metabolites in DI contents, the potential biomarkers for SBMIE in pearl gentian grouper were selected in SBM20_N and SBM40_N group, respectively.

First, in positive and negative ion mode, the differential metabolites were filtered according to VIP > 1 and *P* < 0.05; then, the top 10 potential biomarkers for SBMIE were screened in DI contents in SBM20_N and SBM40_N groups according to VIP value, and the co-contained differential metabolites in SBM20_N and SBM40_N group were considered as the potential “core biomarker” for SBMIE of pearl gentian grouper.

*The inverse correlation analysis*

The metabolomics of DI contents and DI tissues were combined to analyze the co-contained metabolites (among them, the metabolites of DI contents which were significantly affected by diet sources were removed.). The co-contained metabolites with opposite trend and *P* < 0.05 were counted. These metabolites may exchange between DI contents and intestinal tissues and play an important role in the development of enteritis.

**Supplementary file S3**

**Western Blotting description**

The western blotting results of this experiment were obtained through the traditional method. There are ten holes in one gel. Each group had three repeated holes. The examples of each group are as follows：


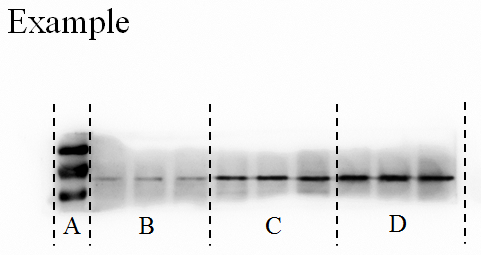


A, Marker; B, FM control group; C, SBM20 group; D, SBM40 group

Note: the black box indicates the edge of the blots

The original images of each protein by chemiluminescence are shown below.


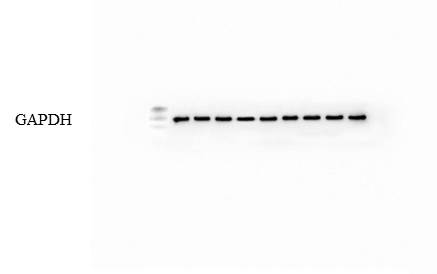

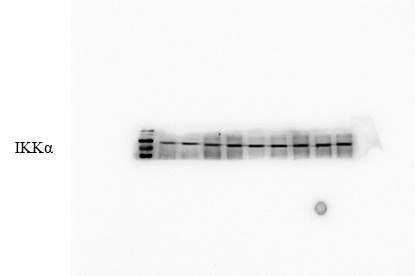

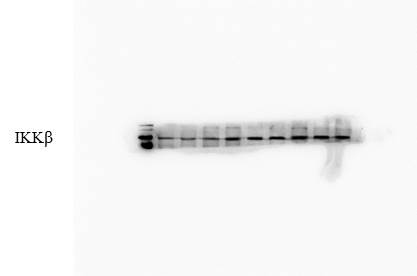

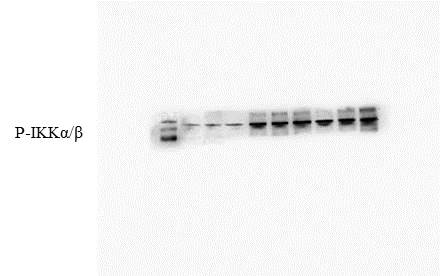

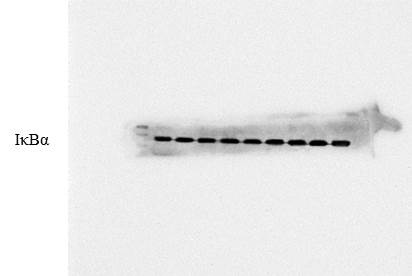

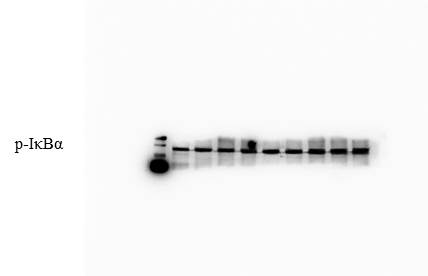

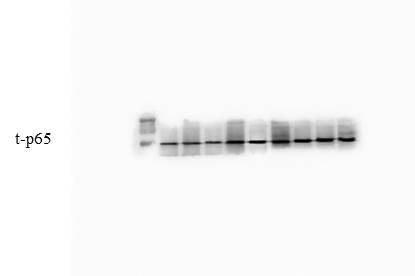

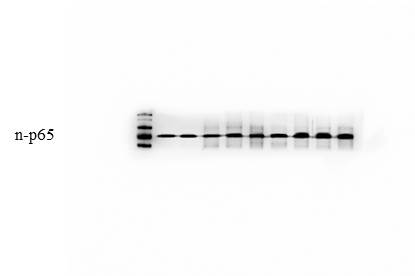

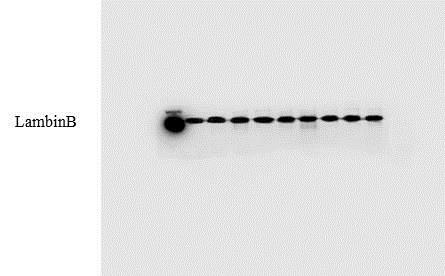


60 kDa

80 kDa

100 kDa

50 kDa

30 kDa

40 kDa

100 kDa

80 kDa

60 kDa

100 kDa

60 kDa

80 kDa

40 kDa

30 kDa

50 kDa

50 kDa

30 kDa

40 kDa

80 kDa

60 kDa

80 kDa

40 kDa

60 kDa

80 kDa

60 kDa
