# Supplementary material for: Multiomics analysis of soybean meal induced marine fish enteritis in juvenile pearl gentian grouper, Epinephelus fuscoguttatus ♀ × Epinephelus lanceolatus ♂
Source: Sci Rep. 2021 Dec 2;11:23319. doi: 10.1038/s41598-021-02278-z (PMC8640039; doi:10.1038/s41598-021-02278-z)
Supplement: Supplementary file 3 — Supplementary Tables. [file 41598_2021_2278_MOESM3_ESM.docx]

**Supplementary Tables**

**Supplementary Table 1. The *β* diversity index differences of intestinal microflora of pearl gentian grouper fed by different levels of soybean meal diets (n=3)**

| Group-Pair | Difference | pvalue | sig. | LCL | UCL |
| --- | --- | --- | --- | --- | --- |
| FM-SBM20 | -1.66667 | 0.2488 |  | -4.86052 | 1.52719 |
| FM-SBM40 | -5.33333 | 0.0065 | ** | -8.52719 | -2.13948 |
| SBM20-SBM40 | -3.66667 | 0.0308 | * | -6.86052 | -0.47281 |

| **Supplementary Table 2. The top 10 species of relative abundance of DI microflora at phylum level in pearl gentian grouper fed by different soybean meal diets (n=3)** | | | | | | | | |
| --- | --- | --- | --- | --- | --- | --- | --- | --- |
| Taxa | avg(SBM40) | sd(SBM40) | avg(FM) | sd(FM) | p.value | q.values | interval lower | interval upper |
| k__Bacteria;p__Proteobacteria; | 0.419844819 | 0.194512754 | 0.997241493 | 0.000561603 | 0.035808552 | -0.088680652 | -1.060587435 | -0.094205912 |
| k__Bacteria;p__Firmicutes; | 0.3526781 | 0.140003511 | 0.001561198 | 0.000785328 | 0.049120683 | -0.088680652 | 0.003344402 | 0.698889402 |
| k__Bacteria;p__Bacteroidetes; | 0.174924581 | 0.061993246 | 0.000316935 | 0 | 0.039542991 | -0.088680652 | 0.020607886 | 0.328607406 |
| k__Bacteria;p__Actinobacteria; | 0.021481142 | 0.011593221 | 0.000234767 | 0.00014232 | 0.086533484 | -0.088680652 | -0.007546637 | 0.050039389 |
| k__Bacteria;p__Cyanobacteria; | 0.007125166 | 0.009598028 | 8.22E-05 | 0.0001132 | 0.331559705 | -0.074415995 | -0.016795129 | 0.030881124 |
| k__Bacteria;p__Chloroflexi; | 0.003967555 | 0.006539408 | 0 | 0 | 0.403565527 | -0.068072125 | -0.012277236 | 0.020212346 |
| k__Bacteria;p__Acidobacteria; | 0.003451069 | 0.005550467 | 5.87E-05 | 5.38E-05 | 0.400743991 | -0.068072125 | -0.010393914 | 0.017178669 |
| k__Bacteria;p__Verrucomicrobia; | 0.002324189 | 0.003324228 | 1.17E-05 | 2.03E-05 | 0.351477968 | -0.068072125 | -0.005944951 | 0.010569853 |
| k__Bacteria;p__unidentified_Bacteria; | 0.002993274 | 0.002628652 | 3.52E-05 | 6.10E-05 | 0.190547933 | -0.088680652 | -0.003566904 | 0.009483022 |
| k__Bacteria;p__Nitrospirae; | 0.001162095 | 0.001550659 | 0 | 0 | 0.323802671 | -0.079281806 | -0.002689957 | 0.005014146 |
| k__Archaea;p__Euryarchaeota; | 0.001079926 | 0.001214449 | 0 | 0 | 0.263410492 | -0.088680652 | -0.001936933 | 0.004096785 |
| k__Bacteria;p__Tenericutes; | 0.000856898 | 0.001220052 | 0 | 0 | 0.347876283 | -0.072072114 | -0.00217388 | 0.003887676 |
| k__Bacteria;p__Fusobacteria; | 0.000669085 | 0.00073193 | 3.52E-05 | 6.10E-05 | 0.271949134 | -0.083803118 | -0.001166905 | 0.002434645 |
| k__Bacteria;p__Gemmatimonadetes; | 0.000328673 | 0.000480699 | 0 | 0 | 0.357973765 | -0.068072125 | -0.000865448 | 0.001522795 |
| k__Archaea;p__Thaumarchaeota; | 0.000387365 | 0.000288247 | 0 | 0 | 0.145375934 | -0.088680652 | -0.000328681 | 0.00110341 |
| k__Bacteria;p__Spirochaetes; | 0.000187813 | 0.000325302 | 0 | 0 | 0.422649731 | -0.068072125 | -0.000620282 | 0.000995909 |
| k__Archaea;p__Crenarchaeota; | 0.000164337 | 0.000284639 | 0 | 0 | 0.422649731 | -0.068072125 | -0.000542747 | 0.00087142 |
| k__Bacteria;p__Gracilibacteria; | 0.000129122 | 0.00016642 | 0 | 0 | 0.31115328 | -0.083803118 | -0.000284287 | 0.000542531 |
| k__Bacteria;p__Rokubacteria; | 5.87E-05 | 0.000101657 | 0 | 0 | 0.422649731 | -0.068072125 | -0.000193838 | 0.000311221 |
| k__Bacteria;p__Latescibacteria; | 3.52E-05 | 6.10E-05 | 1.17E-05 | 2.03E-05 | 0.581314764 | -0.068072125 | -0.000111618 | 0.000158571 |
| k__Bacteria;p__Planctomycetes; | 2.35E-05 | 4.07E-05 | 0 | 0 | 0.422649731 | -0.068072125 | -7.75E-05 | 0.000124489 |
| k__Bacteria;p__Armatimonadetes; | 1.17E-05 | 2.03E-05 | 0 | 0 | 0.422649731 | -0.068072125 | -3.88E-05 | 6.22E-05 |
| Others | 0.006115669 | 0.001856846 | 0.000410842 | 0.000206341 | 0.0322086 | -0.088680652 | 0.00116932 | 0.010240336 |

| **Supplementary Table 3. The top 10 species of relative abundance of DI microflora at genus level in pearl gentian grouper fed by different soybean meal diets (n=3)** | | | | | | | | |
| --- | --- | --- | --- | --- | --- | --- | --- | --- |
| Taxa | avg(SBM40) | sd(SBM40) | avg(FM) | sd(FM) | p.value | q.values | interval lower | interval upper |
| k__Bacteria;p__Proteobacteria;c__Gammaproteobacteria;o__Vibrionales;f__Vibrionaceae;g__Photobacterium; | 0.199070324 | 0.10046477 | 0.94456 | 0.075836 | 0.000732 | 0.011419 | -0.953382 | -0.5376 |
| k__Bacteria;p__Firmicutes;c__Clostridia;o__Clostridiales;f__Ruminococcaceae;g__Faecalibacterium; | 0.120857837 | 0.15262364 | 0.000657 | 0.000553 | 0.305761 | 0.029845 | -0.258931 | 0.499332 |
| k__Bacteria;p__Proteobacteria;c__Gammaproteobacteria;o__Xanthomonadales;f__Xanthomonadaceae;g__Stenotrophomonas; | 0.071439471 | 0.10175867 | 0.000763 | 0.000845 | 0.352064 | 0.029845 | -0.182081 | 0.323434 |
| k__Bacteria;p__Proteobacteria;c__Gammaproteobacteria;o__Vibrionales;f__Vibrionaceae;g__Vibrio; | 0.025800848 | 0.03514555 | 0.04531 | 0.076986 | 0.718182 | 0.047896 | -0.18152 | 0.142502 |
| k__Bacteria;p__Proteobacteria;c__Gammaproteobacteria;o__unidentified_Gammaproteobacteria;f__Neisseriaceae;g__Neisseria; | 0.019755608 | 0.03367026 | 0 | 0 | 0.416442 | 0.029845 | -0.063886 | 0.103397 |
| k__Bacteria;p__Bacteroidetes;c__Bacteroidia;o__Bacteroidales;f__Bacteroidaceae;g__Bacteroides; | 0.038619103 | 0.010783 | 4.70E-05 | 2.03E-05 | 0.025074 | 0.029845 | 0.011786 | 0.065358 |
| k__Bacteria;p__Bacteroidetes;c__Bacteroidia;o__Bacteroidales;f__Rikenellaceae;g__unidentified_Rikenellaceae; | 0.015142445 | 0.02622748 | 0 | 0 | 0.42265 | 0.029845 | -0.05001 | 0.080295 |
| k__Bacteria;p__Firmicutes;c__Bacilli;o__Lactobacillales;f__Streptococcaceae;g__Streptococcus; | 0.016527567 | 0.02177159 | 3.52E-05 | 3.52E-05 | 0.319866 | 0.029845 | -0.037591 | 0.070576 |
| k__Bacteria;p__Firmicutes;c__Clostridia;o__Clostridiales;f__Peptostreptococcaceae;g__Romboutsia; | 0.014156425 | 0.02120504 | 3.52E-05 | 3.52E-05 | 0.36796 | 0.029845 | -0.038555 | 0.066797 |
| k__Bacteria;p__Firmicutes;c__Clostridia;o__Clostridiales;f__Ruminococcaceae;g__Subdoligranulum; | 0.023030602 | 0.01861311 | 5.87E-05 | 5.38E-05 | 0.165992 | 0.029845 | -0.023265 | 0.069209 |
| k__Bacteria;p__Firmicutes;c__Bacilli;o__Lactobacillales;f__Lactobacillaceae;g__Lactobacillus; | 0.01475508 | 0.01743476 | 9.39E-05 | 0.000133 | 0.282549 | 0.029845 | -0.028646 | 0.057968 |
| k__Bacteria;p__Firmicutes;c__Negativicutes;o__Selenomonadales;f__Veillonellaceae;g__Dialister; | 0.011644423 | 0.01950164 | 1.17E-05 | 2.03E-05 | 0.410095 | 0.029845 | -0.036812 | 0.060077 |
| k__Bacteria;p__Proteobacteria;c__Alphaproteobacteria;o__Rhizobiales;f__Rhizobiaceae;g__Phyllobacterium; | 0.012219601 | 0.01655206 | 0.000153 | 0.000206 | 0.33397 | 0.029845 | -0.029042 | 0.053176 |
| k__Bacteria;p__Firmicutes;c__Clostridia;o__Clostridiales;f__Lachnospiraceae;g__Agathobacter; | 0.012524797 | 0.01477857 | 2.35E-05 | 4.07E-05 | 0.280495 | 0.029845 | -0.02421 | 0.049213 |
| k__Bacteria;p__Actinobacteria;c__unidentified_Actinobacteria;o__Bifidobacteriales;f__Bifidobacteriaceae;g__Bifidobacterium; | 0.011562254 | 0.01471904 | 0.000129 | 7.33E-05 | 0.310743 | 0.029845 | -0.02513 | 0.047996 |
| k__Bacteria;p__Proteobacteria;c__Gammaproteobacteria;o__unidentified_Gammaproteobacteria;f__Burkholderiaceae;g__Delftia; | 0.010271038 | 0.01584175 | 0.000141 | 0.000214 | 0.383414 | 0.029845 | -0.029213 | 0.049473 |
| k__Bacteria;p__Firmicutes;c__Clostridia;o__Clostridiales;f__Ruminococcaceae;g__unidentified_Ruminococcaceae; | 0.014273808 | 0.01264808 | 5.87E-05 | 2.03E-05 | 0.190962 | 0.029845 | -0.017204 | 0.045635 |
| k__Bacteria;p__Bacteroidetes;c__Bacteroidia;o__Bacteroidales;f__Rikenellaceae;g__Anaerocella; | 0.006878661 | 0.01188371 | 0 | 0 | 0.421664 | 0.029845 | -0.022642 | 0.036399 |
| k__Bacteria;p__Bacteroidetes;c__Bacteroidia;o__Bacteroidales;f__Rikenellaceae;g__Alistipes; | 0.012501321 | 0.00826488 | 0 | 0 | 0.120022 | 0.029845 | -0.00803 | 0.033032 |
| k__Bacteria;p__Firmicutes;c__Clostridia;o__Clostridiales;f__Lachnospiraceae;g__Roseburia; | 0.010505805 | 0.00856016 | 4.70E-05 | 4.07E-05 | 0.168563 | 0.029845 | -0.010805 | 0.031723 |
| k__Bacteria;p__Firmicutes;c__Clostridia;o__Clostridiales;f__Lachnospiraceae;g__Lachnoclostridium; | 0.007242549 | 0.01027568 | 1.17E-05 | 2.03E-05 | 0.347164 | 0.029845 | -0.018295 | 0.032757 |
| k__Bacteria;p__Cyanobacteria;c__unidentified_Cyanobacteria;o__unidentified_Cyanobacteria;f__unidentified_Cyanobacteria;g__unidentified_Cyanobacteria; | 0.007125166 | 0.00959803 | 8.22E-05 | 0.000113 | 0.33156 | 0.029845 | -0.016795 | 0.030881 |
| k__Bacteria;p__Firmicutes;c__Clostridia;o__Clostridiales;f__Lachnospiraceae;g__Blautia; | 0.009566738 | 0.00401209 | 5.87E-05 | 2.03E-05 | 0.054538 | 0.029845 | -0.000458 | 0.019474 |
| k__Bacteria;p__Firmicutes;c__Clostridia;o__Clostridiales;f__unidentified_Clostridiales;g__Proteiniclasticum; | 0.004718808 | 0.00814274 | 0 | 0 | 0.421212 | 0.029845 | -0.015509 | 0.024946 |
| k__Bacteria;p__Firmicutes;c__Clostridia;o__Clostridiales;f__Lachnospiraceae;g__Lachnospira; | 0.005000528 | 0.00739448 | 1.17E-05 | 2.03E-05 | 0.363025 | 0.029845 | -0.01338 | 0.023357 |
| k__Bacteria;p__Bacteroidetes;c__Bacteroidia;o__Sphingobacteriales;f__Lentimicrobiaceae;g__Lentimicrobium; | 0.004448827 | 0.00764468 | 0 | 0 | 0.419595 | 0.029845 | -0.014542 | 0.023439 |
| k__Bacteria;p__Firmicutes;c__Clostridia;o__Clostridiales;f__unidentified_Clostridiales;g__unidentified_Clostridiales; | 0.006409128 | 0.0066147 | 1.17E-05 | 2.03E-05 | 0.235889 | 0.029845 | -0.010034 | 0.022829 |
| k__Bacteria;p__Firmicutes;c__Clostridia;o__Clostridiales;f__Lachnospiraceae;g__unidentified_Lachnospiraceae; | 0.006819969 | 0.00438541 | 5.87E-05 | 5.38E-05 | 0.116254 | 0.029845 | -0.00413 | 0.017653 |
| k__Bacteria;p__Proteobacteria;c__Alphaproteobacteria;o__Rhodobacterales;f__Rhodobacteraceae;g__Nautella; | 0.005786996 | 0.00544198 | 1.17E-05 | 2.03E-05 | 0.207431 | 0.029845 | -0.007743 | 0.019294 |
| k__Bacteria;p__Bacteroidetes;c__Bacteroidia;o__Bacteroidales;f__Paludibacteraceae;g__Paludibacter; | 0.003908864 | 0.00677035 | 0 | 0 | 0.42265 | 0.029845 | -0.01291 | 0.020727 |
| k__Bacteria;p__Firmicutes;c__Negativicutes;o__Selenomonadales;f__Veillonellaceae;g__Megamonas; | 0.004331443 | 0.00619393 | 0 | 0 | 0.349504 | 0.029845 | -0.011055 | 0.019718 |
| k__Bacteria;p__Proteobacteria;c__Alphaproteobacteria;o__Rhizobiales;f__Hyphomicrobiaceae;g__Hyphomicrobium; | 0.003838434 | 0.00655708 | 0 | 0 | 0.417329 | 0.029845 | -0.01245 | 0.020127 |
| k__Bacteria;p__Firmicutes;c__Clostridia;o__Clostridiales;f__unidentified_Clostridiales;g__Sedimentibacter; | 0.003486284 | 0.00603842 | 0 | 0 | 0.42265 | 0.029845 | -0.011514 | 0.018487 |
| k__Bacteria;p__Firmicutes;c__Clostridia;o__Clostridiales;f__Peptostreptococcaceae;g__Acetoanaerobium; | 0.003333685 | 0.00577411 | 0 | 0 | 0.42265 | 0.029845 | -0.01101 | 0.017677 |
| k__Bacteria;p__Proteobacteria;c__Gammaproteobacteria;o__Enterobacteriales;f__Enterobacteriaceae;g__Serratia; | 0.003192826 | 0.00496198 | 0 | 0 | 0.381034 | 0.029845 | -0.009133 | 0.015519 |
| k__Bacteria;p__Firmicutes;c__Erysipelotrichia;o__Erysipelotrichales;f__Erysipelotrichaceae;g__unidentified_Erysipelotrichaceae; | 0.004554472 | 0.00389191 | 0 | 0 | 0.179889 | 0.029845 | -0.005114 | 0.014223 |
| k__Bacteria;p__Proteobacteria;c__Gammaproteobacteria;o__Pasteurellales;f__Pasteurellaceae;g__Haemophilus; | 0.003310209 | 0.00393448 | 1.17E-05 | 2.03E-05 | 0.283616 | 0.029845 | -0.006475 | 0.013072 |
| k__Bacteria;p__Proteobacteria;c__Gammaproteobacteria;o__unidentified_Gammaproteobacteria;f__Rhodocyclaceae;g__Thauera; | 0.002570694 | 0.00442211 | 0 | 0 | 0.420007 | 0.029845 | -0.008414 | 0.013556 |
| k__Bacteria;p__Proteobacteria;c__Alphaproteobacteria;o__Sphingomonadales;f__Sphingomonadaceae;g__Sphingomonas; | 0.004249275 | 0.00314072 | 0.000129 | 0.000133 | 0.150771 | 0.029845 | -0.003662 | 0.011902 |
| k__Bacteria;p__Bacteroidetes;c__Bacteroidia;o__Chitinophagales;f__Chitinophagaceae;g__Ferruginibacter; | 0.002312451 | 0.00400528 | 0 | 0 | 0.42265 | 0.029845 | -0.007637 | 0.012262 |
| k__Bacteria;p__Bacteroidetes;c__Bacteroidia;o__Bacteroidales;f__Tannerellaceae;g__Parabacteroides; | 0.003345424 | 0.00226118 | 2.35E-05 | 4.07E-05 | 0.125876 | 0.029845 | -0.002293 | 0.008936 |
| k__Bacteria;p__Firmicutes;c__Clostridia;o__Clostridiales;f__Ruminococcaceae;g__Fournierella; | 0.001795964 | 0.0031107 | 0 | 0 | 0.42265 | 0.029845 | -0.005931 | 0.009523 |
| k__Bacteria;p__Bacteroidetes;c__Bacteroidia;o__Bacteroidales;f__Prevotellaceae;g__Paraprevotella; | 0.001725534 | 0.00298871 | 0 | 0 | 0.42265 | 0.029845 | -0.005699 | 0.00915 |
| k__Bacteria;p__Verrucomicrobia;c__Verrucomicrobiae;o__Verrucomicrobiales;f__Akkermansiaceae;g__Akkermansia; | 0.001784226 | 0.0029076 | 0 | 0 | 0.399205 | 0.029845 | -0.005439 | 0.009007 |
| k__Bacteria;p__Proteobacteria;c__Gammaproteobacteria;o__Oceanospirillales;f__unidentified_Oceanospirillales;g__Marinobacterium; | 0.001608151 | 0.00266434 | 0 | 0 | 0.405555 | 0.029845 | -0.00501 | 0.008227 |
| k__Bacteria;p__Firmicutes;c__Negativicutes;o__Selenomonadales;f__Veillonellaceae;g__Megasphaera; | 0.001713796 | 0.00257198 | 0 | 0 | 0.367734 | 0.029845 | -0.004675 | 0.008103 |
| k__Bacteria;p__Bacteroidetes;c__Bacteroidia;o__Bacteroidales;f__Prevotellaceae;g__Alloprevotella; | 0.002465049 | 0.00209196 | 1.17E-05 | 2.03E-05 | 0.179307 | 0.029845 | -0.002743 | 0.007649 |
| k__Bacteria;p__Proteobacteria;c__Alphaproteobacteria;o__Rhizobiales;f__Xanthobacteraceae;g__Bradyrhizobium; | 0.001889871 | 0.00220743 | 1.17E-05 | 2.03E-05 | 0.278483 | 0.029845 | -0.003605 | 0.007361 |
| k__Bacteria;p__Proteobacteria;c__Gammaproteobacteria;o__unidentified_Gammaproteobacteria;f__Nitrosomonadaceae;g__unidentified_Nitrosomonadaceae; | 0.001349908 | 0.00227739 | 0 | 0 | 0.412523 | 0.029845 | -0.004307 | 0.007007 |
| k__Bacteria;p__Actinobacteria;c__Acidimicrobiia;o__unidentified_Acidimicrobiia;f__unidentified_Acidimicrobiia;g__Candidatus_Microthrix; | 0.001314693 | 0.00227711 | 0 | 0 | 0.42265 | 0.029845 | -0.004342 | 0.006971 |
| k__Bacteria;p__Firmicutes;c__Clostridia;o__Clostridiales;f__Lachnospiraceae;g__Fusicatenibacter; | 0.002911106 | 0.00063907 | 0 | 0 | 0.015687 | 0.029845 | 0.001324 | 0.004499 |
| k__Bacteria;p__Proteobacteria;c__Gammaproteobacteria;o__Enterobacteriales;f__Enterobacteriaceae;g__Klebsiella; | 0.001760749 | 0.00162104 | 0 | 0 | 0.200656 | 0.029845 | -0.002266 | 0.005788 |
| k__Bacteria;p__Firmicutes;c__Clostridia;o__Clostridiales;f__Ruminococcaceae;g__Intestinimonas; | 0.001326431 | 0.00171881 | 1.17E-05 | 2.03E-05 | 0.31633 | 0.029845 | -0.002954 | 0.005584 |
| k__Bacteria;p__Bacteroidetes;c__Bacteroidia;o__Bacteroidales;f__Tannerellaceae;g__Macellibacteroides; | 0.00126774 | 0.00167706 | 0 | 0 | 0.320634 | 0.029845 | -0.002898 | 0.005434 |
| k__Bacteria;p__Firmicutes;c__Clostridia;o__Clostridiales;f__Ruminococcaceae;g__Oscillibacter; | 0.002007254 | 0.00114272 | 0 | 0 | 0.093179 | 0.029845 | -0.000831 | 0.004846 |
| k__Bacteria;p__Nitrospirae;c__Nitrospira;o__Nitrospirales;f__Nitrospiraceae;g__unidentified_Nitrospiraceae; | 0.001068188 | 0.00152702 | 0 | 0 | 0.349386 | 0.029845 | -0.002725 | 0.004862 |
| k__Bacteria;p__Firmicutes;c__Clostridia;o__Clostridiales;f__Lachnospiraceae;g__Tyzzerella; | 0.001032973 | 0.00154519 | 0 | 0 | 0.366496 | 0.029845 | -0.002805 | 0.004871 |
| k__Bacteria;p__Bacteroidetes;c__Bacteroidia;o__Chitinophagales;f__Chitinophagaceae;g__Terrimonas; | 0.000903851 | 0.00156552 | 0 | 0 | 0.42265 | 0.029845 | -0.002985 | 0.004793 |
| k__Bacteria;p__Firmicutes;c__Clostridia;o__Clostridiales;f__Lachnospiraceae;g__Dorea; | 0.001537721 | 0.00124121 | 0 | 0 | 0.165029 | 0.029845 | -0.001546 | 0.004621 |
| k__Bacteria;p__Proteobacteria;c__Gammaproteobacteria;o__Xanthomonadales;f__Xanthomonadaceae;g__Arenimonas; | 0.000856898 | 0.00148419 | 0 | 0 | 0.42265 | 0.029845 | -0.00283 | 0.004544 |
| k__Bacteria;p__Firmicutes;c__Bacilli;o__Lactobacillales;f__Enterococcaceae;g__Enterococcus; | 0.001103403 | 0.00128924 | 1.17E-05 | 2.03E-05 | 0.280135 | 0.029845 | -0.00211 | 0.004293 |
| k__Bacteria;p__Firmicutes;c__Erysipelotrichia;o__Erysipelotrichales;f__Erysipelotrichaceae;g__Turicibacter; | 0.001032973 | 0.00131307 | 0 | 0 | 0.306161 | 0.029845 | -0.002229 | 0.004295 |
| k__Bacteria;p__Firmicutes;c__Negativicutes;o__Selenomonadales;f__Acidaminococcaceae;g__Phascolarctobacterium; | 0.001749011 | 0.00083804 | 0 | 0 | 0.068732 | 0.029845 | -0.000333 | 0.003831 |
| k__Bacteria;p__Proteobacteria;c__Gammaproteobacteria;o__Pseudomonadales;f__Pseudomonadaceae;g__Pseudomonas; | 0.00119731 | 0.00125348 | 2.35E-05 | 4.07E-05 | 0.246178 | 0.029845 | -0.001935 | 0.004283 |
| k__Bacteria;p__Firmicutes;c__Bacilli;o__Lactobacillales;f__Carnobacteriaceae;g__Granulicatella; | 0.00084516 | 0.00143347 | 0 | 0 | 0.414576 | 0.029845 | -0.002716 | 0.004406 |
| k__Bacteria;p__Firmicutes;c__Clostridia;o__Clostridiales;f__unidentified_Clostridiales;g__Candidatus_Arthromitus; | 0.000821683 | 0.0014232 | 0 | 0 | 0.42265 | 0.029845 | -0.002714 | 0.004357 |
| k__Bacteria;p__Proteobacteria;c__Gammaproteobacteria;o__Pseudomonadales;f__Moraxellaceae;g__Moraxella; | 0.000833421 | 0.00141314 | 4.70E-05 | 8.13E-05 | 0.436839 | 0.030708 | -0.002708 | 0.004281 |
| k__Bacteria;p__Proteobacteria;c__Alphaproteobacteria;o__Rhodobacterales;f__Rhodobacteraceae;g__Salinihabitans; | 0.000927328 | 0.00124819 | 2.35E-05 | 4.07E-05 | 0.336447 | 0.029845 | -0.002192 | 0.004 |
| k__Bacteria;p__Proteobacteria;c__Gammaproteobacteria;o__Pasteurellales;f__Pasteurellaceae;g__Actinobacillus; | 0.000868636 | 0.00129212 | 0 | 0 | 0.364379 | 0.029845 | -0.002341 | 0.004078 |
| k__Bacteria;p__unidentified_Bacteria;c__unidentified_Bacteria;o__unidentified_Bacteria;f__unidentified_Bacteria;g__unidentified_Bacteria; | 0.000786468 | 0.0013622 | 0 | 0 | 0.42265 | 0.029845 | -0.002597 | 0.00417 |
| k__Archaea;p__Euryarchaeota;c__Methanomicrobia;o__Methanosarcinales;f__Methanosaetaceae;g__Methanosaeta; | 0.00105645 | 0.00117641 | 0 | 0 | 0.260105 | 0.029845 | -0.001866 | 0.003979 |
| k__Bacteria;p__Bacteroidetes;c__Bacteroidia;o__Bacteroidales;f__unidentified_Bacteroidales;g__Petrimonas; | 0.000739515 | 0.00128088 | 0 | 0 | 0.42265 | 0.029845 | -0.002442 | 0.003921 |
| k__Bacteria;p__Actinobacteria;c__unidentified_Actinobacteria;o__Micrococcales;f__Micrococcaceae;g__Rothia; | 0.001690319 | 0.00055345 | 0 | 0 | 0.033927 | 0.029845 | 0.000315 | 0.003065 |
| k__Bacteria;p__unidentified_Bacteria;c__unidentified_Bacteria;o__Campylobacterales;f__Helicobacteraceae;g__Helicobacter; | 0.00091559 | 0.00110857 | 3.52E-05 | 6.10E-05 | 0.302627 | 0.029845 | -0.001862 | 0.003623 |
| k__Bacteria;p__Proteobacteria;c__Alphaproteobacteria;o__Rhodobacterales;f__Rhodobacteraceae;g__Donghicola; | 0.00098602 | 0.00097844 | 0 | 0 | 0.223021 | 0.029845 | -0.001445 | 0.003417 |
| k__Bacteria;p__Firmicutes;c__Erysipelotrichia;o__Erysipelotrichales;f__Erysipelotrichaceae;g__Erysipelatoclostridium; | 0.000716038 | 0.00120985 | 0 | 0 | 0.413109 | 0.029845 | -0.002289 | 0.003721 |
| k__Bacteria;p__Proteobacteria;c__Gammaproteobacteria;o__unidentified_Gammaproteobacteria;f__Burkholderiaceae;g__Parasutterella; | 0.000868636 | 0.00109185 | 0 | 0 | 0.302135 | 0.029845 | -0.001844 | 0.003581 |
| k__Bacteria;p__Bacteroidetes;c__Bacteroidia;o__Flavobacteriales;f__unidentified_Flavobacteriales;g__unidentified_Flavobacteriales; | 0.000680823 | 0.00117922 | 0 | 0 | 0.42265 | 0.029845 | -0.002249 | 0.00361 |
| k__Bacteria;p__Proteobacteria;c__Gammaproteobacteria;o__Alteromonadales;f__Alteromonadaceae;g__Glaciecola; | 0.000833421 | 0.00099624 | 0 | 0 | 0.28436 | 0.029845 | -0.001641 | 0.003308 |
| k__Bacteria;p__Firmicutes;c__Clostridia;o__Clostridiales;f__Ruminococcaceae;g__Butyricicoccus; | 0.000821683 | 0.00097866 | 0 | 0 | 0.283097 | 0.029845 | -0.001609 | 0.003253 |
| k__Bacteria;p__Firmicutes;c__Clostridia;o__Clostridiales;f__Peptostreptococcaceae;g__Intestinibacter; | 0.001467291 | 0.00039581 | 0 | 0 | 0.023408 | 0.029845 | 0.000484 | 0.002451 |
| k__Bacteria;p__Proteobacteria;c__Alphaproteobacteria;o__Caulobacterales;f__Caulobacteraceae;g__Brevundimonas; | 0.001185571 | 0.00095167 | 5.87E-05 | 5.38E-05 | 0.17636 | 0.029845 | -0.001227 | 0.00348 |
| k__Bacteria;p__Proteobacteria;c__Gammaproteobacteria;o__unidentified_Gammaproteobacteria;f__Burkholderiaceae;g__Candidatus_Profftella; | 0.000575178 | 0.00099624 | 0 | 0 | 0.42265 | 0.029845 | -0.0019 | 0.00305 |
| k__Bacteria;p__Chloroflexi;c__unidentified_Chloroflexi;o__unidentified_Chloroflexi;f__unidentified_Chloroflexi;g__unidentified_Chloroflexi; | 0.00056344 | 0.00097591 | 0 | 0 | 0.42265 | 0.029845 | -0.001861 | 0.002988 |
| k__Bacteria;p__Proteobacteria;c__Gammaproteobacteria;o__unidentified_Gammaproteobacteria;f__Hydrogenophilaceae;g__Thiobacillus; | 0.00021129 | 0.00021129 | 0 | 0 | 0.225403 | 0.029845 | -0.000314 | 0.000736 |
| k__Bacteria;p__Proteobacteria;c__Gammaproteobacteria;o__unidentified_Gammaproteobacteria;f__Burkholderiaceae;g__Pelomonas; | 0.00077473 | 0.00077232 | 2.35E-05 | 2.03E-05 | 0.234006 | 0.029845 | -0.001165 | 0.002668 |
| k__Bacteria;p__Proteobacteria;c__Gammaproteobacteria;o__Enterobacteriales;f__Enterobacteriaceae;g__Mangrovibacter; | 0.000927328 | 0.0008454 | 0 | 0 | 0.197835 | 0.029845 | -0.001173 | 0.003027 |
| k__Bacteria;p__Proteobacteria;c__Gammaproteobacteria;o__Pseudomonadales;f__Moraxellaceae;g__Acinetobacter; | 0.000739515 | 0.00076749 | 1.17E-05 | 2.03E-05 | 0.242151 | 0.029845 | -0.001177 | 0.002632 |
| k__Bacteria;p__Proteobacteria;c__Gammaproteobacteria;o__unidentified_Gammaproteobacteria;f__Burkholderiaceae;g__Achromobacter; | 0.000516486 | 0.00089458 | 2.35E-05 | 2.03E-05 | 0.440557 | 0.03083 | -0.001728 | 0.002714 |
| k__Bacteria;p__Bacteroidetes;c__Bacteroidia;o__Bacteroidales;f__Marinifilaceae;g__Butyricimonas; | 0.000586916 | 0.00078533 | 0 | 0 | 0.324817 | 0.029845 | -0.001364 | 0.002538 |
| k__Bacteria;p__Proteobacteria;c__Gammaproteobacteria;o__unidentified_Gammaproteobacteria;f__unidentified_Gammaproteobacteria;g__unidentified_Gammaproteobacteria; | 0.000340412 | 0.00035274 | 0 | 0 | 0.236578 | 0.029845 | -0.000536 | 0.001217 |
| k__Bacteria;p__Proteobacteria;c__Alphaproteobacteria;o__Rhizobiales;f__unidentified_Rhizobiales;g__Nordella; | 0.000516486 | 0.00069216 | 1.17E-05 | 2.03E-05 | 0.333847 | 0.029845 | -0.001213 | 0.002222 |
| k__Bacteria;p__unidentified_Bacteria;c__unidentified_Bacteria;o__unidentified_Bacteria;f__unidentified_Bacteria;g__Candidatus_Saccharimonas; | 0.00042258 | 0.00073193 | 0 | 0 | 0.42265 | 0.029845 | -0.001396 | 0.002241 |
| k__Bacteria;p__Proteobacteria;c__Gammaproteobacteria;o__Enterobacteriales;f__Enterobacteriaceae;g__Enterobacter; | 0.000481271 | 0.00065913 | 1.17E-05 | 2.03E-05 | 0.342557 | 0.029845 | -0.001166 | 0.002105 |
| k__Bacteria;p__Proteobacteria;c__Gammaproteobacteria;o__unidentified_Gammaproteobacteria;f__Rhodocyclaceae;g__Denitratisoma; | 0.000410842 | 0.0007116 | 0 | 0 | 0.42265 | 0.029845 | -0.001357 | 0.002179 |
| k__Bacteria;p__Firmicutes;c__Negativicutes;o__Selenomonadales;f__Veillonellaceae;g__Veillonella; | 0.000645608 | 0.00050338 | 0 | 0 | 0.156438 | 0.029845 | -0.000605 | 0.001896 |
| k__Bacteria;p__Firmicutes;c__Clostridia;o__Clostridiales;f__Christensenellaceae;g__unidentified_Christensenellaceae; | 0.000399103 | 0.00069127 | 0 | 0 | 0.42265 | 0.029845 | -0.001318 | 0.002116 |
| k__Bacteria;p__Firmicutes;c__Erysipelotrichia;o__Erysipelotrichales;f__Erysipelotrichaceae;g__Holdemanella; | 0.000446057 | 0.00062235 | 0 | 0 | 0.340302 | 0.029845 | -0.0011 | 0.001992 |
| k__Bacteria;p__Firmicutes;c__Clostridia;o__Clostridiales;f__Lachnospiraceae;g__Anaerostipes; | 0.000598655 | 0.0004605 | 1.17E-05 | 2.03E-05 | 0.157713 | 0.029845 | -0.000554 | 0.001728 |
| k__Bacteria;p__Proteobacteria;c__Gammaproteobacteria;o__unidentified_Gammaproteobacteria;f__Burkholderiaceae;g__Sutterella; | 0.000739515 | 0.000307 | 0 | 0 | 0.052926 | 0.029845 | -2.31E-05 | 0.001502 |
| k__Bacteria;p__Bacteroidetes;c__Bacteroidia;o__Bacteroidales;f__Marinifilaceae;g__Odoribacter; | 0.000434318 | 0.00057362 | 0 | 0 | 0.320041 | 0.029845 | -0.000991 | 0.001859 |
| k__Bacteria;p__Proteobacteria;c__Deltaproteobacteria;o__Desulfovibrionales;f__Desulfovibrionaceae;g__Desulfovibrio; | 0.000610393 | 0.00042453 | 0 | 0 | 0.13043 | 0.029845 | -0.000444 | 0.001665 |
| k__Bacteria;p__Proteobacteria;c__Deltaproteobacteria;o__Desulfovibrionales;f__Desulfovibrionaceae;g__Bilophila; | 0.000575178 | 0.00043464 | 0 | 0 | 0.148951 | 0.029845 | -0.000505 | 0.001655 |
| k__Bacteria;p__Actinobacteria;c__Acidimicrobiia;o__unidentified_Acidimicrobiia;f__Iamiaceae;g__Iamia; | 0.000316935 | 0.00054895 | 0 | 0 | 0.42265 | 0.029845 | -0.001047 | 0.001681 |
| k__Bacteria;p__Firmicutes;c__Bacilli;o__Bacillales;f__Staphylococcaceae;g__Staphylococcus; | 0.000446057 | 0.00046496 | 3.52E-05 | 6.10E-05 | 0.264452 | 0.029845 | -0.000718 | 0.001539 |
| k__Bacteria;p__Actinobacteria;c__Coriobacteriia;o__Coriobacteriales;f__Atopobiaceae;g__Olsenella; | 0.00035215 | 0.00052113 | 0 | 0 | 0.362423 | 0.029845 | -0.000942 | 0.001647 |
| k__Bacteria;p__Verrucomicrobia;c__Verrucomicrobiae;o__Verrucomicrobiales;f__Rubritaleaceae;g__Rubritalea; | 0.000434318 | 0.0004807 | 0 | 0 | 0.258068 | 0.029845 | -0.00076 | 0.001628 |
| k__Bacteria;p__Actinobacteria;c__unidentified_Actinobacteria;o__Corynebacteriales;f__Mycobacteriaceae;g__Mycobacterium; | 0.000305197 | 0.00049843 | 1.17E-05 | 2.03E-05 | 0.415131 | 0.029845 | -0.000942 | 0.001529 |
| k__Bacteria;p__Firmicutes;c__Clostridia;o__Clostridiales;f__Ruminococcaceae;g__Flavonifractor; | 0.000387365 | 0.00042696 | 2.35E-05 | 4.07E-05 | 0.277354 | 0.029845 | -0.000684 | 0.001411 |
| k__Bacteria;p__Proteobacteria;c__Gammaproteobacteria;o__Methylococcales;f__unidentified_Methylococcales;g__Methylobacter; | 0.00028172 | 0.00045779 | 0 | 0 | 0.398116 | 0.029845 | -0.000856 | 0.001419 |
| k__Bacteria;p__Acidobacteria;c__unidentified_Acidobacteria;o__unidentified_Acidobacteria;f__unidentified_Acidobacteria;g__unidentified_Acidobacteria; | 9.39E-05 | 0.00010758 | 0 | 0 | 0.269703 | 0.029845 | -0.000173 | 0.000361 |
| k__Bacteria;p__Proteobacteria;c__Gammaproteobacteria;o__Cardiobacteriales;f__unidentified_Cardiobacteriales;g__Ignatzschineria; | 0.000234767 | 0.00040663 | 0 | 0 | 0.42265 | 0.029845 | -0.000775 | 0.001245 |
| k__Bacteria;p__Bacteroidetes;c__Bacteroidia;o__Flavobacteriales;f__Flavobacteriaceae;g__Aureimarina; | 0.000293458 | 0.00034203 | 0 | 0 | 0.275591 | 0.029845 | -0.000556 | 0.001143 |
| k__Archaea;p__Thaumarchaeota;c__Nitrososphaeria;o__Nitrosopumilales;f__Nitrosopumilaceae;g__Candidatus_Nitrosoarchaeum; | 0.00021129 | 0.00036596 | 0 | 0 | 0.42265 | 0.029845 | -0.000698 | 0.00112 |
| k__Bacteria;p__Proteobacteria;c__Gammaproteobacteria;o__unidentified_Gammaproteobacteria;f__Burkholderiaceae;g__Massilia; | 0.000258243 | 0.00030767 | 2.35E-05 | 4.07E-05 | 0.316617 | 0.029845 | -0.000512 | 0.000981 |
| k__Bacteria;p__Proteobacteria;c__Gammaproteobacteria;o__unidentified_Gammaproteobacteria;f__Burkholderiaceae;g__Alcaligenes; | 0.000187813 | 0.0003253 | 0 | 0 | 0.42265 | 0.029845 | -0.00062 | 0.000996 |
| k__Bacteria;p__Actinobacteria;c__Coriobacteriia;o__Coriobacteriales;f__Eggerthellaceae;g__Senegalimassilia; | 0.000187813 | 0.0003253 | 0 | 0 | 0.42265 | 0.029845 | -0.00062 | 0.000996 |
| k__Bacteria;p__Firmicutes;c__Erysipelotrichia;o__Erysipelotrichales;f__Erysipelotrichaceae;g__Dubosiella; | 0.000316935 | 0.0002465 | 0 | 0 | 0.155838 | 0.029845 | -0.000295 | 0.000929 |
| k__Bacteria;p__Actinobacteria;c__unidentified_Actinobacteria;o__Corynebacteriales;f__Corynebacteriaceae;g__unidentified_Corynebacteriaceae; | 0.00021129 | 0.00027504 | 0 | 0 | 0.314752 | 0.029845 | -0.000472 | 0.000895 |
| k__Bacteria;p__Proteobacteria;c__Alphaproteobacteria;o__unidentified_Alphaproteobacteria;f__Acetobacteraceae;g__Gluconobacter; | 0.000176075 | 0.00030497 | 0 | 0 | 0.42265 | 0.029845 | -0.000582 | 0.000934 |
| k__Bacteria;p__Firmicutes;c__Bacilli;o__Bacillales;f__unidentified_Bacillales;g__Gemella; | 0.000399103 | 0.00019395 | 1.17E-05 | 2.03E-05 | 0.072815 | 0.029845 | -8.72E-05 | 0.000862 |
| k__Bacteria;p__Fusobacteria;c__Fusobacteriia;o__Fusobacteriales;f__Fusobacteriaceae;g__Fusobacterium; | 0.000246505 | 0.00025394 | 3.52E-05 | 6.10E-05 | 0.28408 | 0.029845 | -0.000377 | 0.0008 |
| k__Bacteria;p__Proteobacteria;c__Alphaproteobacteria;o__unidentified_Alphaproteobacteria;f__Acetobacteraceae;g__Acetobacter; | 0.000199552 | 0.00028681 | 0 | 0 | 0.35141 | 0.029845 | -0.000513 | 0.000912 |
| k__Bacteria;p__Bacteroidetes;c__Bacteroidia;o__Flavobacteriales;f__Flavobacteriaceae;g__Flavobacterium; | 0.000246505 | 0.00026587 | 1.17E-05 | 2.03E-05 | 0.265377 | 0.029845 | -0.00042 | 0.00089 |
| k__Archaea;p__Crenarchaeota;c__unidentified_Crenarchaeota;o__unidentified_Crenarchaeota;f__unidentified_Crenarchaeota;g__unidentified_Crenarchaeota; | 0.000164337 | 0.00028464 | 0 | 0 | 0.42265 | 0.029845 | -0.000543 | 0.000871 |
| k__Bacteria;p__unidentified_Bacteria;c__unidentified_Bacteria;o__Campylobacterales;f__unidentified_Campylobacterales;g__Sulfurimonas; | 1.17E-05 | 2.03E-05 | 0 | 0 | 0.42265 | 0.029845 | -3.88E-05 | 6.22E-05 |
| k__Bacteria;p__Gemmatimonadetes;c__unidentified_Gemmatimonadetes;o__Gemmatimonadales;f__Gemmatimonadaceae;g__Gemmatimonas; | 0.000164337 | 0.00028464 | 0 | 0 | 0.42265 | 0.029845 | -0.000543 | 0.000871 |
| k__Bacteria;p__Proteobacteria;c__Gammaproteobacteria;o__unidentified_Gammaproteobacteria;f__Burkholderiaceae;g__unidentified_Burkholderiaceae; | 8.22E-05 | 0.00014232 | 0 | 0 | 0.42265 | 0.029845 | -0.000271 | 0.000436 |
| k__Bacteria;p__Firmicutes;c__Negativicutes;o__Selenomonadales;f__Acidaminococcaceae;g__Acidaminococcus; | 0.000176075 | 0.0002465 | 0 | 0 | 0.341573 | 0.029845 | -0.000436 | 0.000788 |
| k__Bacteria;p__Fusobacteria;c__Fusobacteriia;o__Fusobacteriales;f__Fusobacteriaceae;g__Psychrilyobacter; | 0.000152598 | 0.00026431 | 0 | 0 | 0.42265 | 0.029845 | -0.000504 | 0.000809 |
| k__Bacteria;p__Firmicutes;c__Bacilli;o__Lactobacillales;f__Leuconostocaceae;g__Weissella; | 0.00021129 | 0.00018634 | 2.35E-05 | 4.07E-05 | 0.219259 | 0.029845 | -0.000249 | 0.000624 |
| k__Bacteria;p__Firmicutes;c__Bacilli;o__Bacillales;f__Staphylococcaceae;g__Jeotgalicoccus; | 0.000129122 | 0.00022365 | 0 | 0 | 0.42265 | 0.029845 | -0.000426 | 0.000685 |
| k__Bacteria;p__Bacteroidetes;c__Bacteroidia;o__Bacteroidales;f__Prevotellaceae;g__unidentified_Prevotellaceae; | 0.00028172 | 9.32E-05 | 0 | 0 | 0.034578 | 0.029845 | 5.03E-05 | 0.000513 |
| k__Bacteria;p__Proteobacteria;c__Gammaproteobacteria;o__Cellvibrionales;f__Halieaceae;g__Halioglobus; | 0.000316935 | 7.04E-05 | 0 | 0 | 0.016065 | 0.029845 | 0.000142 | 0.000492 |
| k__Bacteria;p__Proteobacteria;c__Gammaproteobacteria;o__Enterobacteriales;f__Enterobacteriaceae;g__Pantoea; | 0.000129122 | 0.00022365 | 1.17E-05 | 2.03E-05 | 0.459498 | 0.032012 | -0.000432 | 0.000667 |
| k__Bacteria;p__Fusobacteria;c__Fusobacteriia;o__Fusobacteriales;f__Fusobacteriaceae;g__Cetobacterium; | 0.000199552 | 0.00019395 | 0 | 0 | 0.216681 | 0.029845 | -0.000282 | 0.000681 |
| k__Bacteria;p__Firmicutes;c__Clostridia;o__Clostridiales;f__Ruminococcaceae;g__Ruminiclostridium; | 0.000129122 | 0.00022365 | 0 | 0 | 0.42265 | 0.029845 | -0.000426 | 0.000685 |
| k__Bacteria;p__Firmicutes;c__Clostridia;o__Clostridiales;f__Ruminococcaceae;g__Anaerotruncus; | 0.000117383 | 0.00020331 | 0 | 0 | 0.42265 | 0.029845 | -0.000388 | 0.000622 |
| k__Bacteria;p__Proteobacteria;c__Alphaproteobacteria;o__unidentified_Alphaproteobacteria;f__unidentified_Alphaproteobacteria;g__unidentified_Alphaproteobacteria; | 0.00014086 | 0.00012199 | 9.39E-05 | 0.000163 | 0.711111 | 0.047628 | -0.000289 | 0.000383 |
| k__Bacteria;p__Firmicutes;c__Bacilli;o__Lactobacillales;f__Streptococcaceae;g__Lactococcus; | 0.000199552 | 0.00018071 | 2.35E-05 | 2.03E-05 | 0.232459 | 0.029845 | -0.000265 | 0.000617 |
| k__Bacteria;p__Proteobacteria;c__Gammaproteobacteria;o__unidentified_Gammaproteobacteria;f__Burkholderiaceae;g__Lautropia; | 0.000105645 | 0.00018298 | 0 | 0 | 0.42265 | 0.029845 | -0.000349 | 0.00056 |
| k__Bacteria;p__Proteobacteria;c__Gammaproteobacteria;o__Enterobacteriales;f__Enterobacteriaceae;g__Citrobacter; | 0.000199552 | 0.00017371 | 0 | 0 | 0.184912 | 0.029845 | -0.000232 | 0.000631 |
| k__Bacteria;p__Proteobacteria;c__Gammaproteobacteria;o__Aeromonadales;f__Aeromonadaceae;g__Aeromonas; | 0.000164337 | 0.00015879 | 0 | 0 | 0.214919 | 0.029845 | -0.00023 | 0.000559 |
| k__Bacteria;p__Gracilibacteria;c__unidentified_Gracilibacteria;o__unidentified_Gracilibacteria;f__unidentified_Gracilibacteria;g__unidentified_Gracilibacteria; | 0.000129122 | 0.00016642 | 0 | 0 | 0.311153 | 0.029845 | -0.000284 | 0.000543 |
| k__Bacteria;p__Tenericutes;c__Mollicutes;o__Mycoplasmatales;f__Mycoplasmataceae;g__Mycoplasma; | 0.000105645 | 0.00018298 | 0 | 0 | 0.42265 | 0.029845 | -0.000349 | 0.00056 |
| k__Bacteria;p__Firmicutes;c__Bacilli;o__Lactobacillales;f__Lactobacillaceae;g__Pediococcus; | 0.000129122 | 0.00016642 | 0 | 0 | 0.311153 | 0.029845 | -0.000284 | 0.000543 |
| k__Bacteria;p__Proteobacteria;c__Deltaproteobacteria;o__unidentified_Deltaproteobacteria;f__unidentified_Deltaproteobacteria;g__unidentified_Deltaproteobacteria; | 7.04E-05 | 0.00012199 | 0 | 0 | 0.42265 | 0.029845 | -0.000233 | 0.000373 |
| k__Bacteria;p__Actinobacteria;c__unidentified_Actinobacteria;o__Actinomycetales;f__Actinomycetaceae;g__Actinomyces; | 0.000258243 | 7.33E-05 | 0 | 0 | 0.025824 | 0.029845 | 7.61E-05 | 0.00044 |
| k__Bacteria;p__Actinobacteria;c__unidentified_Actinobacteria;o__Propionibacteriales;f__Propionibacteriaceae;g__Cutibacterium; | 0.000152598 | 0.00012367 | 1.17E-05 | 2.03E-05 | 0.184395 | 0.029845 | -0.000156 | 0.000437 |
| k__Bacteria;p__Firmicutes;c__Bacilli;o__Bacillales;f__Bacillaceae;g__Pseudogracilibacillus; | 9.39E-05 | 0.00016265 | 0 | 0 | 0.42265 | 0.029845 | -0.00031 | 0.000498 |
| k__Bacteria;p__Proteobacteria;c__Alphaproteobacteria;o__Sphingomonadales;f__Sphingomonadaceae;g__Parablastomonas; | 9.39E-05 | 0.00016265 | 0 | 0 | 0.42265 | 0.029845 | -0.00031 | 0.000498 |
| k__Bacteria;p__Proteobacteria;c__Alphaproteobacteria;o__Rhodobacterales;f__Rhodobacteraceae;g__Paracoccus; | 9.39E-05 | 0.00016265 | 1.17E-05 | 2.03E-05 | 0.474396 | 0.032903 | -0.000313 | 0.000478 |
| k__Bacteria;p__Actinobacteria;c__unidentified_Actinobacteria;o__Micrococcales;f__Microbacteriaceae;g__Microbacterium; | 9.39E-05 | 0.00016265 | 0 | 0 | 0.42265 | 0.029845 | -0.00031 | 0.000498 |
| k__Bacteria;p__Bacteroidetes;c__Bacteroidia;o__Flavobacteriales;f__unidentified_Flavobacteriales;g__Cloacibacterium; | 0.000117383 | 0.00014661 | 0 | 0 | 0.29986 | 0.029845 | -0.000247 | 0.000482 |
| k__Bacteria;p__Proteobacteria;c__Alphaproteobacteria;o__Rhizobiales;f__unidentified_Rhizobiales;g__Devosia; | 0.000187813 | 0.00010758 | 0 | 0 | 0.094178 | 0.029845 | -7.94E-05 | 0.000455 |
| k__Bacteria;p__Fusobacteria;c__Fusobacteriia;o__Fusobacteriales;f__Leptotrichiaceae;g__Leptotrichia; | 7.04E-05 | 7.04E-05 | 0 | 0 | 0.225403 | 0.029845 | -0.000105 | 0.000245 |
| k__Bacteria;p__Proteobacteria;c__Gammaproteobacteria;o__Alteromonadales;f__Pseudoalteromonadaceae;g__Pseudoalteromonas; | 0.000164337 | 0.0001132 | 1.17E-05 | 2.03E-05 | 0.140657 | 0.029845 | -0.000117 | 0.000422 |
| k__Bacteria;p__Verrucomicrobia;c__Verrucomicrobiae;o__Chthoniobacterales;f__unidentified_Chthoniobacterales;g__Terrimicrobium; | 8.22E-05 | 0.00014232 | 0 | 0 | 0.42265 | 0.029845 | -0.000271 | 0.000436 |
| k__Bacteria;p__Acidobacteria;c__Acidobacteriia;o__Solibacterales;f__unidentified_Solibacterales;g__Bryobacter; | 0.000129122 | 0.00012367 | 0 | 0 | 0.212274 | 0.029845 | -0.000178 | 0.000436 |
| k__Bacteria;p__Actinobacteria;c__Coriobacteriia;o__Coriobacteriales;f__Eggerthellaceae;g__Enterorhabdus; | 0.00014086 | 0.00010564 | 0 | 0 | 0.147197 | 0.029845 | -0.000122 | 0.000403 |
| k__Bacteria;p__Proteobacteria;c__Gammaproteobacteria;o__Alteromonadales;f__Alteromonadaceae;g__Alteromonas; | 0.000129122 | 0.00012367 | 0 | 0 | 0.212274 | 0.029845 | -0.000178 | 0.000436 |
| k__Bacteria;p__Firmicutes;c__Bacilli;o__Lactobacillales;f__Carnobacteriaceae;g__Atopostipes; | 8.22E-05 | 0.00014232 | 1.17E-05 | 2.03E-05 | 0.482466 | 0.033315 | -0.000274 | 0.000414 |
| k__Archaea;p__Euryarchaeota;c__Methanomicrobia;o__Methanomicrobiales;f__Methanoregulaceae;g__Methanoregula; | 2.35E-05 | 4.07E-05 | 0 | 0 | 0.42265 | 0.029845 | -7.75E-05 | 0.000124 |
| k__Bacteria;p__Proteobacteria;c__Gammaproteobacteria;o__unidentified_Gammaproteobacteria;f__Burkholderiaceae;g__Ramlibacter; | 0.00014086 | 0.00012697 | 1.17E-05 | 2.03E-05 | 0.217979 | 0.029845 | -0.000176 | 0.000434 |
| k__Bacteria;p__Proteobacteria;c__Gammaproteobacteria;o__unidentified_Gammaproteobacteria;f__Burkholderiaceae;g__Ralstonia; | 7.04E-05 | 0.00012199 | 3.52E-05 | 6.10E-05 | 0.685598 | 0.046117 | -0.000218 | 0.000289 |
| k__Bacteria;p__Firmicutes;c__Bacilli;o__Bacillales;f__Bacillaceae;g__Geobacillus; | 7.04E-05 | 0.00012199 | 0 | 0 | 0.42265 | 0.029845 | -0.000233 | 0.000373 |
| k__Bacteria;p__Proteobacteria;c__Alphaproteobacteria;o__Rhizobiales;f__Rhizobiaceae;g__Aquamicrobium; | 8.22E-05 | 0.0001132 | 0 | 0 | 0.335589 | 0.029845 | -0.000199 | 0.000363 |
| k__Bacteria;p__unidentified_Bacteria;c__unidentified_Bacteria;o__Campylobacterales;f__Campylobacteraceae;g__Campylobacter; | 0.000129122 | 0.0001132 | 0 | 0 | 0.186857 | 0.029845 | -0.000152 | 0.00041 |
| k__Bacteria;p__Firmicutes;c__Clostridia;o__Clostridiales;f__unidentified_Clostridiales;g__Peptoniphilus; | 7.04E-05 | 0.00012199 | 0 | 0 | 0.42265 | 0.029845 | -0.000233 | 0.000373 |
| k__Bacteria;p__Firmicutes;c__Bacilli;o__Bacillales;f__Bacillaceae;g__Bacillus; | 0.000105645 | 0.00010564 | 0 | 0 | 0.225403 | 0.029845 | -0.000157 | 0.000368 |
| k__Bacteria;p__Firmicutes;c__Bacilli;o__Lactobacillales;f__Carnobacteriaceae;g__Jeotgalibaca; | 9.39E-05 | 0.00010758 | 0 | 0 | 0.269703 | 0.029845 | -0.000173 | 0.000361 |
| k__Bacteria;p__Firmicutes;c__Bacilli;o__Bacillales;f__Bacillaceae;g__Anoxybacillus; | 0.000152598 | 5.38E-05 | 1.17E-05 | 2.03E-05 | 0.032672 | 0.029845 | 2.41E-05 | 0.000258 |
| k__Bacteria;p__Actinobacteria;c__unidentified_Actinobacteria;o__Micrococcales;f__Microbacteriaceae;g__Rhodoluna; | 7.04E-05 | 0.00012199 | 0 | 0 | 0.42265 | 0.029845 | -0.000233 | 0.000373 |
| k__Bacteria;p__Proteobacteria;c__Alphaproteobacteria;o__Rhizobiales;f__Rhizobiaceae;g__unidentified_Rhizobiaceae; | 8.22E-05 | 0.0001132 | 0 | 0 | 0.335589 | 0.029845 | -0.000199 | 0.000363 |
| k__Bacteria;p__Firmicutes;c__Bacilli;o__Bacillales;f__Bacillaceae;g__Virgibacillus; | 7.04E-05 | 0.00012199 | 0 | 0 | 0.42265 | 0.029845 | -0.000233 | 0.000373 |
| k__Bacteria;p__Proteobacteria;c__Gammaproteobacteria;o__unidentified_Gammaproteobacteria;f__Francisellaceae;g__Francisella; | 7.04E-05 | 0.00012199 | 1.17E-05 | 2.03E-05 | 0.493525 | 0.033928 | -0.000234 | 0.000351 |
| k__Bacteria;p__Proteobacteria;c__Alphaproteobacteria;o__Rhizobiales;f__Rhizobiaceae;g__Pseudorhizobium; | 8.22E-05 | 8.86E-05 | 0 | 0 | 0.249521 | 0.029845 | -0.000138 | 0.000302 |
| k__Bacteria;p__Rokubacteria;c__unidentified_Rokubacteria;o__unidentified_Rokubacteria;f__unidentified_Rokubacteria;g__Candidatus_Methylomirabilis; | 5.87E-05 | 0.00010166 | 0 | 0 | 0.42265 | 0.029845 | -0.000194 | 0.000311 |
| k__Bacteria;p__Actinobacteria;c__unidentified_Actinobacteria;o__Propionibacteriales;f__Nocardioidaceae;g__Aeromicrobium; | 5.87E-05 | 7.33E-05 | 0 | 0 | 0.29986 | 0.029845 | -0.000123 | 0.000241 |
| k__Bacteria;p__Bacteroidetes;c__Bacteroidia;o__Bacteroidales;f__Barnesiellaceae;g__Barnesiella; | 4.70E-05 | 8.13E-05 | 0 | 0 | 0.42265 | 0.029845 | -0.000155 | 0.000249 |
| k__Bacteria;p__Firmicutes;c__Erysipelotrichia;o__Erysipelotrichales;f__Erysipelotrichaceae;g__Allobaculum; | 4.70E-05 | 8.13E-05 | 0 | 0 | 0.42265 | 0.029845 | -0.000155 | 0.000249 |
| k__Bacteria;p__Actinobacteria;c__Coriobacteriia;o__Coriobacteriales;f__Atopobiaceae;g__Atopobium; | 4.70E-05 | 8.13E-05 | 0 | 0 | 0.42265 | 0.029845 | -0.000155 | 0.000249 |
| k__Bacteria;p__Proteobacteria;c__Gammaproteobacteria;o__Pseudomonadales;f__Moraxellaceae;g__Enhydrobacter; | 8.22E-05 | 5.38E-05 | 0 | 0 | 0.118083 | 0.029845 | -5.15E-05 | 0.000216 |
| k__Bacteria;p__Proteobacteria;c__Deltaproteobacteria;o__Syntrophobacterales;f__Syntrophaceae;g__Syntrophus; | 4.70E-05 | 8.13E-05 | 0 | 0 | 0.42265 | 0.029845 | -0.000155 | 0.000249 |
| k__Bacteria;p__Bacteroidetes;c__Bacteroidia;o__Bacteroidales;f__Muribaculaceae;g__Muribaculum; | 9.39E-05 | 8.13E-05 | 0 | 0 | 0.183503 | 0.029845 | -0.000108 | 0.000296 |
| k__Bacteria;p__Firmicutes;c__Negativicutes;o__Selenomonadales;f__Acidaminococcaceae;g__Succiniclasticum; | 4.70E-05 | 8.13E-05 | 0 | 0 | 0.42265 | 0.029845 | -0.000155 | 0.000249 |
| k__Bacteria;p__Proteobacteria;c__Alphaproteobacteria;o__Rhizobiales;f__Xanthobacteraceae;g__Pseudolabrys; | 7.04E-05 | 7.04E-05 | 0 | 0 | 0.225403 | 0.029845 | -0.000105 | 0.000245 |
| k__Bacteria;p__Proteobacteria;c__Gammaproteobacteria;o__unidentified_Gammaproteobacteria;f__Burkholderiaceae;g__Rhodoferax; | 8.22E-05 | 7.33E-05 | 0 | 0 | 0.19171 | 0.029845 | -9.99E-05 | 0.000264 |
| k__Bacteria;p__Firmicutes;c__Clostridia;o__Clostridiales;f__unidentified_Clostridiales;g__Parvimonas; | 4.70E-05 | 8.13E-05 | 0 | 0 | 0.42265 | 0.029845 | -0.000155 | 0.000249 |
| k__Bacteria;p__Bacteroidetes;c__Bacteroidia;o__Cytophagales;f__Hymenobacteraceae;g__Pontibacter; | 9.39E-05 | 8.13E-05 | 0 | 0 | 0.183503 | 0.029845 | -0.000108 | 0.000296 |
| k__Bacteria;p__Firmicutes;c__Erysipelotrichia;o__Erysipelotrichales;f__Erysipelotrichaceae;g__Ileibacterium; | 3.52E-05 | 6.10E-05 | 1.17E-05 | 2.03E-05 | 0.581315 | 0.039442 | -0.000112 | 0.000159 |
| k__Bacteria;p__Bacteroidetes;c__Bacteroidia;o__Flavobacteriales;f__Flavobacteriaceae;g__Tenacibaculum; | 3.52E-05 | 6.10E-05 | 0 | 0 | 0.42265 | 0.029845 | -0.000116 | 0.000187 |
| k__Bacteria;p__Proteobacteria;c__Deltaproteobacteria;o__Desulfuromonadales;f__Geobacteraceae;g__Geobacter; | 3.52E-05 | 6.10E-05 | 0 | 0 | 0.42265 | 0.029845 | -0.000116 | 0.000187 |
| k__Bacteria;p__Proteobacteria;c__Alphaproteobacteria;o__Sphingomonadales;f__Sphingomonadaceae;g__Novosphingobium; | 8.22E-05 | 2.03E-05 | 0 | 0 | 0.019804 | 0.029845 | 3.17E-05 | 0.000133 |
| k__Bacteria;p__Proteobacteria;c__Gammaproteobacteria;o__Alteromonadales;f__unidentified_Alteromonadales;g__Marinobacter; | 2.35E-05 | 4.07E-05 | 1.17E-05 | 2.03E-05 | 0.685598 | 0.046117 | -7.27E-05 | 9.62E-05 |
| k__Bacteria;p__unidentified_Bacteria;c__unidentified_Bacteria;o__unidentified_Bacteria;f__unidentified_Bacteria;g__Coprothermobacter; | 3.52E-05 | 6.10E-05 | 0 | 0 | 0.42265 | 0.029845 | -0.000116 | 0.000187 |
| k__Bacteria;p__Proteobacteria;c__Gammaproteobacteria;o__Enterobacteriales;f__Enterobacteriaceae;g__Proteus; | 3.52E-05 | 6.10E-05 | 1.17E-05 | 2.03E-05 | 0.581315 | 0.039442 | -0.000112 | 0.000159 |
| k__Bacteria;p__Bacteroidetes;c__Bacteroidia;o__Flavobacteriales;f__Crocinitomicaceae;g__Fluviicola; | 5.87E-05 | 5.38E-05 | 0 | 0 | 0.199359 | 0.029845 | -7.49E-05 | 0.000192 |
| k__Bacteria;p__Proteobacteria;c__Gammaproteobacteria;o__Cellvibrionales;f__Cellvibrionaceae;g__Marinagarivorans; | 3.52E-05 | 6.10E-05 | 0 | 0 | 0.42265 | 0.029845 | -0.000116 | 0.000187 |
| k__Bacteria;p__Actinobacteria;c__Acidimicrobiia;o__unidentified_Acidimicrobiia;f__unidentified_Acidimicrobiia;g__unidentified_Acidimicrobiia; | 3.52E-05 | 6.10E-05 | 0 | 0 | 0.42265 | 0.029845 | -0.000116 | 0.000187 |
| k__Bacteria;p__Proteobacteria;c__Gammaproteobacteria;o__Oceanospirillales;f__Halomonadaceae;g__Halomonas; | 3.52E-05 | 6.10E-05 | 0 | 0 | 0.42265 | 0.029845 | -0.000116 | 0.000187 |
| k__Bacteria;p__Proteobacteria;c__Gammaproteobacteria;o__unidentified_Gammaproteobacteria;f__Rhodocyclaceae;g__Sulfuritalea; | 3.52E-05 | 6.10E-05 | 0 | 0 | 0.42265 | 0.029845 | -0.000116 | 0.000187 |
| k__Bacteria;p__Actinobacteria;c__unidentified_Actinobacteria;o__Propionibacteriales;f__Nocardioidaceae;g__Nocardioides; | 3.52E-05 | 6.10E-05 | 0 | 0 | 0.42265 | 0.029845 | -0.000116 | 0.000187 |
| k__Bacteria;p__Proteobacteria;c__Alphaproteobacteria;o__Rhizobiales;f__Beijerinckiaceae;g__Methylobacterium; | 4.70E-05 | 5.38E-05 | 0 | 0 | 0.269703 | 0.029845 | -8.67E-05 | 0.000181 |
| k__Bacteria;p__Firmicutes;c__Erysipelotrichia;o__Erysipelotrichales;f__Erysipelotrichaceae;g__Faecalibaculum; | 4.70E-05 | 5.38E-05 | 0 | 0 | 0.269703 | 0.029845 | -8.67E-05 | 0.000181 |
| k__Bacteria;p__Gemmatimonadetes;c__unidentified_Gemmatimonadetes;o__Gemmatimonadales;f__Gemmatimonadaceae;g__Gemmatirosa; | 2.35E-05 | 4.07E-05 | 0 | 0 | 0.42265 | 0.029845 | -7.75E-05 | 0.000124 |
| k__Bacteria;p__Bacteroidetes;c__Bacteroidia;o__Flavobacteriales;f__unidentified_Flavobacteriales;g__Chryseobacterium; | 2.35E-05 | 4.07E-05 | 0 | 0 | 0.42265 | 0.029845 | -7.75E-05 | 0.000124 |
| k__Bacteria;p__Bacteroidetes;c__Bacteroidia;o__Chitinophagales;f__Saprospiraceae;g__unidentified_Saprospiraceae; | 3.52E-05 | 3.52E-05 | 0 | 0 | 0.225403 | 0.029845 | -5.23E-05 | 0.000123 |
| k__Bacteria;p__Proteobacteria;c__Alphaproteobacteria;o__Rhizobiales;f__Xanthobacteraceae;g__Xanthobacter; | 2.35E-05 | 4.07E-05 | 0 | 0 | 0.42265 | 0.029845 | -7.75E-05 | 0.000124 |
| k__Bacteria;p__Proteobacteria;c__Gammaproteobacteria;o__Pasteurellales;f__Pasteurellaceae;g__Aggregatibacter; | 2.35E-05 | 4.07E-05 | 0 | 0 | 0.42265 | 0.029845 | -7.75E-05 | 0.000124 |
| k__Bacteria;p__Firmicutes;c__Bacilli;o__Bacillales;f__Bacillaceae;g__Cerasibacillus; | 0 | 0 | 2.35E-05 | 4.07E-05 | 0.42265 | 0.029845 | -0.000124 | 7.75E-05 |
| k__Bacteria;p__Firmicutes;c__Erysipelotrichia;o__Erysipelotrichales;f__Erysipelotrichaceae;g__Faecalitalea; | 2.35E-05 | 4.07E-05 | 0 | 0 | 0.42265 | 0.029845 | -7.75E-05 | 0.000124 |
| k__Bacteria;p__Verrucomicrobia;c__Verrucomicrobiae;o__Chthoniobacterales;f__Chthoniobacteraceae;g__Candidatus_Udaeobacter; | 2.35E-05 | 4.07E-05 | 0 | 0 | 0.42265 | 0.029845 | -7.75E-05 | 0.000124 |
| k__Bacteria;p__Bacteroidetes;c__Bacteroidia;o__Flavobacteriales;f__unidentified_Flavobacteriales;g__Elizabethkingia; | 2.35E-05 | 4.07E-05 | 0 | 0 | 0.42265 | 0.029845 | -7.75E-05 | 0.000124 |
| k__Bacteria;p__Bacteroidetes;c__Bacteroidia;o__Sphingobacteriales;f__Sphingobacteriaceae;g__Pedobacter; | 2.35E-05 | 4.07E-05 | 0 | 0 | 0.42265 | 0.029845 | -7.75E-05 | 0.000124 |
| k__Bacteria;p__Planctomycetes;c__Planctomycetacia;o__unidentified_Planctomycetacia;f__Gemmataceae;g__Gemmata; | 2.35E-05 | 4.07E-05 | 0 | 0 | 0.42265 | 0.029845 | -7.75E-05 | 0.000124 |
| k__Bacteria;p__Acidobacteria;c__Acidobacteriia;o__Solibacterales;f__unidentified_Solibacterales;g__Candidatus_Solibacter; | 2.35E-05 | 4.07E-05 | 0 | 0 | 0.42265 | 0.029845 | -7.75E-05 | 0.000124 |
| k__Bacteria;p__Proteobacteria;c__Deltaproteobacteria;o__Myxococcales;f__Haliangiaceae;g__Haliangium; | 0 | 0 | 1.17E-05 | 2.03E-05 | 0.42265 | 0.029845 | -6.22E-05 | 3.88E-05 |
| k__Bacteria;p__Verrucomicrobia;c__Verrucomicrobiae;o__Opitutales;f__Puniceicoccaceae;g__Cerasicoccus; | 0 | 0 | 1.17E-05 | 2.03E-05 | 0.42265 | 0.029845 | -6.22E-05 | 3.88E-05 |
| k__Bacteria;p__Proteobacteria;c__Gammaproteobacteria;o__unidentified_Gammaproteobacteria;f__Methylophilaceae;g__Methylophilus; | 0 | 0 | 1.17E-05 | 2.03E-05 | 0.42265 | 0.029845 | -6.22E-05 | 3.88E-05 |
| k__Bacteria;p__Firmicutes;c__Bacilli;o__Lactobacillales;f__Leuconostocaceae;g__Leuconostoc; | 2.35E-05 | 2.03E-05 | 1.17E-05 | 2.03E-05 | 0.518519 | 0.03549 | -3.44E-05 | 5.78E-05 |
| k__Bacteria;p__Acidobacteria;c__unidentified_Acidobacteria;o__unidentified_Acidobacteria;f__unidentified_Acidobacteria;g__Stenotrophobacter; | 1.17E-05 | 2.03E-05 | 0 | 0 | 0.42265 | 0.029845 | -3.88E-05 | 6.22E-05 |
| k__Bacteria;p__Proteobacteria;c__Gammaproteobacteria;o__Pseudomonadales;f__Moraxellaceae;g__Psychrobacter; | 1.17E-05 | 2.03E-05 | 0 | 0 | 0.42265 | 0.029845 | -3.88E-05 | 6.22E-05 |
| k__Bacteria;p__Proteobacteria;c__Alphaproteobacteria;o__Sphingomonadales;f__Sphingomonadaceae;g__Sphingobium; | 1.17E-05 | 2.03E-05 | 0 | 0 | 0.42265 | 0.029845 | -3.88E-05 | 6.22E-05 |
| k__Bacteria;p__Bacteroidetes;c__Bacteroidia;o__Bacteroidales;f__Rikenellaceae;g__Rikenella; | 1.17E-05 | 2.03E-05 | 0 | 0 | 0.42265 | 0.029845 | -3.88E-05 | 6.22E-05 |
| k__Bacteria;p__Bacteroidetes;c__Bacteroidia;o__Chitinophagales;f__Chitinophagaceae;g__Sediminibacterium; | 0 | 0 | 1.17E-05 | 2.03E-05 | 0.42265 | 0.029845 | -6.22E-05 | 3.88E-05 |
| k__Bacteria;p__Acidobacteria;c__Acidobacteriia;o__Solibacterales;f__unidentified_Solibacterales;g__Paludibaculum; | 1.17E-05 | 2.03E-05 | 0 | 0 | 0.42265 | 0.029845 | -3.88E-05 | 6.22E-05 |
| k__Bacteria;p__Proteobacteria;c__Gammaproteobacteria;o__unidentified_Gammaproteobacteria;f__Methylophilaceae;g__Candidatus_Methylopumilus; | 0 | 0 | 1.17E-05 | 2.03E-05 | 0.42265 | 0.029845 | -6.22E-05 | 3.88E-05 |
| k__Bacteria;p__Proteobacteria;c__Alphaproteobacteria;o__unidentified_Alphaproteobacteria;f__unidentified_Alphaproteobacteria;g__Dongia; | 0 | 0 | 1.17E-05 | 2.03E-05 | 0.42265 | 0.029845 | -6.22E-05 | 3.88E-05 |
| k__Bacteria;p__Actinobacteria;c__unidentified_Actinobacteria;o__Frankiales;f__Geodermatophilaceae;g__Blastococcus; | 1.17E-05 | 2.03E-05 | 0 | 0 | 0.42265 | 0.029845 | -3.88E-05 | 6.22E-05 |
| k__Bacteria;p__Actinobacteria;c__unidentified_Actinobacteria;o__Corynebacteriales;f__Corynebacteriaceae;g__Corynebacterium; | 2.35E-05 | 2.03E-05 | 0 | 0 | 0.183503 | 0.029845 | -2.70E-05 | 7.40E-05 |
| k__Bacteria;p__Firmicutes;c__Bacilli;o__Bacillales;f__Staphylococcaceae;g__Nosocomiicoccus; | 0 | 0 | 1.17E-05 | 2.03E-05 | 0.42265 | 0.029845 | -6.22E-05 | 3.88E-05 |
| k__Bacteria;p__Proteobacteria;c__Gammaproteobacteria;o__Enterobacteriales;f__Enterobacteriaceae;g__Buchnera; | 1.17E-05 | 2.03E-05 | 0 | 0 | 0.42265 | 0.029845 | -3.88E-05 | 6.22E-05 |
| Others | 0.160474698 | 0.05973807 | 0.006433 | 0.000652 | 0.046635 | 0.029845 | 0.005669 | 0.302415 |

**Supplementary Table 4. Significance test of functional abundance difference of intestinal flora of pearl gentian grouper fed by FM and SBM40 diets (n=3)**

| Taxa | avg(FM) | sd(FM) | avg(SBM40) | sd(SBM40) | p.value |
| --- | --- | --- | --- | --- | --- |
| aerobic_chemoheterotrophy | 0.32728 | 0.002954 | 0.101482 | 0.033449 | 0.006891 |
| animal_parasites_or_symbionts | 0.002441 | 0.002224 | 0.058943 | 0.022203 | 0.046547 |
| others | 0.002616 | 0.001163 | 0.152414 | 0.063675 | 0.055237 |
| chemoheterotrophy | 0.328523 | 0.003604 | 0.252637 | 0.041382 | 0.085368 |
| human_gut | 0.00023 | 3.00E-05 | 0.02548 | 0.013772 | 0.086485 |
| mammal_gut | 0.00023 | 3.00E-05 | 0.02548 | 0.013772 | 0.086485 |
| sulfate_respiration | 0 | 0 | 0.000284 | 0.000173 | 0.104776 |
| respiration_of_sulfur_compounds | 0 | 0 | 0.000284 | 0.000173 | 0.104776 |
| human_pathogens_septicemia | 2.73E-05 | 2.50E-05 | 0.000133 | 7.57E-05 | 0.125912 |
| nitrate_reduction | 0.00512 | 0.002899 | 0.046562 | 0.029352 | 0.132993 |
| fermentation | 0.325924 | 0.006 | 0.20517 | 0.088147 | 0.140371 |
| xylanolysis | 1.07E-05 | 9.24E-06 | 0.008637 | 0.006789 | 0.158746 |
| dark_oxidation_of_sulfur_compounds | 3.30E-05 | 3.30E-05 | 0.000233 | 0.000166 | 0.167092 |
| human_pathogens_gastroenteritis | 0 | 0 | 4.37E-05 | 3.80E-05 | 0.184667 |
| dark_sulfide_oxidation | 0 | 0 | 0.000174 | 0.000154 | 0.189202 |
| nitrite_respiration | 3.83E-05 | 3.43E-05 | 0.00299 | 0.002626 | 0.190979 |
| acetoclastic_methanogenesis | 0 | 0 | 0.000492 | 0.000464 | 0.208167 |
| methanogenesis | 0 | 0 | 0.000497 | 0.000471 | 0.209596 |
| cellulolysis | 0 | 0 | 0.000194 | 0.000185 | 0.210247 |
| aromatic_compound_degradation | 5.97E-05 | 3.78E-05 | 0.000387 | 0.000341 | 0.237364 |
| dark_hydrogen_oxidation | 2.20E-05 | 3.81E-05 | 0.000123 | 0.000116 | 0.267963 |
| hydrocarbon_degradation | 3.80E-05 | 3.81E-05 | 0.000307 | 0.000339 | 0.30239 |
| nitrate_respiration | 0.002108 | 0.002169 | 0.030312 | 0.035926 | 0.306753 |
| nitrogen_respiration | 0.002125 | 0.002195 | 0.03056 | 0.036296 | 0.307491 |
| human_pathogens_nosocomia | 2.73E-05 | 2.50E-05 | 0.000243 | 0.000284 | 0.318294 |
| plant_pathogen | 2.17E-05 | 1.88E-05 | 0.00134 | 0.001774 | 0.327058 |
| human_pathogens_all | 0.002173 | 0.002273 | 0.030233 | 0.038994 | 0.338718 |
| fumarate_respiration | 2.73E-05 | 2.50E-05 | 0.000453 | 0.000609 | 0.349143 |
| chitinolysis | 0 | 0 | 0.001202 | 0.001717 | 0.349172 |
| nitrate_ammonification | 0 | 0 | 0.001202 | 0.001717 | 0.349172 |
| nitrite_ammonification | 1.63E-05 | 2.83E-05 | 0.001384 | 0.001975 | 0.353203 |
| cyanobacteria | 0.000131 | 0.000123 | 0.003964 | 0.0056 | 0.35754 |
| oxygenic_photoautotrophy | 0.000131 | 0.000123 | 0.003964 | 0.0056 | 0.35754 |
| photoautotrophy | 0.000131 | 0.000123 | 0.003964 | 0.0056 | 0.35754 |
| phototrophy | 0.000131 | 0.000123 | 0.003964 | 0.0056 | 0.35754 |
| aerobic_nitrite_oxidation | 0 | 0 | 0.000596 | 0.000902 | 0.371023 |
| nitrification | 0 | 0 | 0.000611 | 0.000929 | 0.372357 |
| methylotrophy | 3.80E-05 | 4.13E-05 | 0.000235 | 0.000319 | 0.395834 |
| human_pathogens_diarrhea | 1.63E-05 | 2.83E-05 | 0.000841 | 0.001416 | 0.41958 |
| methanogenesis_by_CO2_reduction_with_H2 | 0 | 0 | 5.00E-06 | 8.66E-06 | 0.42265 |
| hydrogenotrophic_methanogenesis | 0 | 0 | 5.00E-06 | 8.66E-06 | 0.42265 |
| aerobic_ammonia_oxidation | 0 | 0 | 1.53E-05 | 2.66E-05 | 0.42265 |
| arsenite_oxidation_detoxification | 0 | 0 | 6.67E-05 | 0.000115 | 0.42265 |
| dissimilatory_arsenite_oxidation | 0 | 0 | 6.67E-05 | 0.000115 | 0.42265 |
| invertebrate_parasites | 0 | 0 | 1.03E-05 | 1.79E-05 | 0.42265 |
| predatory_or_exoparasitic | 5.33E-06 | 9.24E-06 | 0 | 0 | 0.42265 |
| methanotrophy | 1.10E-05 | 1.91E-05 | 0.000197 | 0.000328 | 0.429993 |
| ureolysis | 2.17E-05 | 2.50E-05 | 5.63E-05 | 6.21E-05 | 0.444409 |
| nitrite_denitrification | 2.20E-05 | 3.81E-05 | 0.000141 | 0.000225 | 0.456577 |
| nitrous_oxide_denitrification | 2.20E-05 | 3.81E-05 | 0.000141 | 0.000225 | 0.456577 |
| denitrification | 2.20E-05 | 3.81E-05 | 0.000141 | 0.000225 | 0.456577 |
| human_pathogens_pneumonia | 4.90E-05 | 4.31E-05 | 0.000261 | 0.000413 | 0.46792 |
| plastic_degradation | 3.27E-05 | 3.30E-05 | 0.000292 | 0.000506 | 0.46825 |
| manganese_oxidation | 2.17E-05 | 1.88E-05 | 0.00019 | 0.000329 | 0.469291 |
| nitrogen_fixation | 1.63E-05 | 2.83E-05 | 0.000128 | 0.000222 | 0.475561 |
| aliphatic_non_methane_hydrocarbon_degradation | 5.33E-06 | 9.24E-06 | 3.83E-05 | 6.64E-05 | 0.480766 |
| nitrate_denitrification | 2.20E-05 | 3.81E-05 | 7.43E-05 | 0.000109 | 0.501913 |
| dark_thiosulfate_oxidation | 3.30E-05 | 3.30E-05 | 5.90E-05 | 5.47E-05 | 0.527516 |
| aromatic_hydrocarbon_degradation | 1.63E-05 | 2.83E-05 | 5.63E-05 | 9.76E-05 | 0.55668 |
| methanol_oxidation | 2.70E-05 | 2.49E-05 | 3.83E-05 | 4.79E-05 | 0.740079 |
| iron_respiration | 5.33E-06 | 9.24E-06 | 5.00E-06 | 8.66E-06 | 0.965826 |

**Supplementary Table 5. The compositions of intestinal flora with significant changes in functional abundance in SBM40 group (n=3)**

| # aerobic_chemoheterotrophy (93 records): |
| --- |
| k__Bacteria;p__Acidobacteria;c__Acidobacteriia;o__Solibacterales;f__unidentified_Solibacterales;g__Bryobacter;s__ |
| k__Bacteria;p__Acidobacteria;c__Acidobacteriia;o__Solibacterales;f__unidentified_Solibacterales;g__Bryobacter;s__ |
| k__Bacteria;p__Acidobacteria;c__Acidobacteriia;o__Solibacterales;f__unidentified_Solibacterales;g__Bryobacter;s__Acidobacteriaceae_bacterium_LX51 |
| k__Bacteria;p__Actinobacteria;c__Thermoleophilia;o__Solirubrobacterales;f__Solirubrobacteraceae;g__Solirubrobacter |
| k__Bacteria;p__Actinobacteria;c__Thermoleophilia;o__Solirubrobacterales;f__Solirubrobacteraceae;g__Solirubrobacter;s__ |
| k__Bacteria;p__Actinobacteria;c__unidentified_Actinobacteria;o__Frankiales;f__Geodermatophilaceae;g__Blastococcus;s__ |
| k__Bacteria;p__Actinobacteria;c__unidentified_Actinobacteria;o__Micrococcales;f__Microbacteriaceae;g__Microbacterium |
| k__Bacteria;p__Actinobacteria;c__unidentified_Actinobacteria;o__Propionibacteriales;f__Nocardioidaceae;g__Aeromicrobium |
| k__Bacteria;p__Actinobacteria;c__unidentified_Actinobacteria;o__Propionibacteriales;f__Nocardioidaceae;g__Nocardioides |
| k__Bacteria;p__Bacteroidetes;c__Bacteroidia;o__Chitinophagales;f__Chitinophagaceae;g__Ferruginibacter;s__ |
| k__Bacteria;p__Bacteroidetes;c__Bacteroidia;o__Flavobacteriales;f__Flavobacteriaceae;g__Flavobacterium |
| k__Bacteria;p__Bacteroidetes;c__Bacteroidia;o__Flavobacteriales;f__Flavobacteriaceae;g__Flavobacterium |
| k__Bacteria;p__Bacteroidetes;c__Bacteroidia;o__Flavobacteriales;f__Flavobacteriaceae;g__Tenacibaculum |
| k__Bacteria;p__Bacteroidetes;c__Bacteroidia;o__Sphingobacteriales;f__Sphingobacteriaceae;g__Pedobacter |
| k__Bacteria;p__Firmicutes;c__Bacilli;o__Bacillales;f__Bacillaceae;g__Geobacillus;s__Geobacillus_stearothermophilus |
| k__Bacteria;p__Proteobacteria;c__Alphaproteobacteria;o__Caulobacterales;f__Caulobacteraceae;g__Caulobacter;s__Caulobacter_fusiformis |
| k__Bacteria;p__Proteobacteria;c__Alphaproteobacteria;o__Rhizobiales;f__Hyphomicrobiaceae;g__Hyphomicrobium;s__ |
| k__Bacteria;p__Proteobacteria;c__Alphaproteobacteria;o__Rhizobiales;f__Hyphomicrobiaceae;g__Hyphomicrobium;s__ |
| k__Bacteria;p__Proteobacteria;c__Alphaproteobacteria;o__Rhizobiales;f__Xanthobacteraceae;g__Xanthobacter;s__ |
| k__Bacteria;p__Proteobacteria;c__Alphaproteobacteria;o__Rhodobacterales;f__Rhodobacteraceae;g__Marivita;s__ |
| k__Bacteria;p__Proteobacteria;c__Alphaproteobacteria;o__Rhodobacterales;f__Rhodobacteraceae;g__Nautella;s__Nautella_italica |
| k__Bacteria;p__Proteobacteria;c__Alphaproteobacteria;o__Rhodobacterales;f__Rhodobacteraceae;g__Paracoccus |
| k__Bacteria;p__Proteobacteria;c__Alphaproteobacteria;o__Sphingomonadales;f__Sphingomonadaceae;g__;s__ |
| k__Bacteria;p__Proteobacteria;c__Alphaproteobacteria;o__Sphingomonadales;f__Sphingomonadaceae;g__Novosphingobium;s__Novosphingobium_subterraneum |
| k__Bacteria;p__Proteobacteria;c__Alphaproteobacteria;o__Sphingomonadales;f__Sphingomonadaceae;g__Parablastomonas;s__ |
| k__Bacteria;p__Proteobacteria;c__Alphaproteobacteria;o__Sphingomonadales;f__Sphingomonadaceae;g__Sphingobium;s__Sphingobium_xenophagum |
| k__Bacteria;p__Proteobacteria;c__Alphaproteobacteria;o__Sphingomonadales;f__Sphingomonadaceae;g__Sphingomonas |
| k__Bacteria;p__Proteobacteria;c__Alphaproteobacteria;o__Sphingomonadales;f__Sphingomonadaceae;g__Sphingomonas |
| k__Bacteria;p__Proteobacteria;c__Alphaproteobacteria;o__Sphingomonadales;f__Sphingomonadaceae;g__Sphingomonas;s__Sphingomonas_leidyi |
| k__Bacteria;p__Proteobacteria;c__Alphaproteobacteria;o__unidentified_Alphaproteobacteria;f__Acetobacteraceae;g__Acetobacter;s__Acetobacter_fabarum |
| k__Bacteria;p__Proteobacteria;c__Alphaproteobacteria;o__unidentified_Alphaproteobacteria;f__Acetobacteraceae;g__Gluconobacter;s__Gluconobacter_frateurii |
| k__Bacteria;p__Proteobacteria;c__Gammaproteobacteria;o__Alteromonadales;f__Alteromonadaceae;g__;s__ |
| k__Bacteria;p__Proteobacteria;c__Gammaproteobacteria;o__Alteromonadales;f__Alteromonadaceae;g__Aestuariibacter |
| k__Bacteria;p__Proteobacteria;c__Gammaproteobacteria;o__Alteromonadales;f__Alteromonadaceae;g__Alteromonas;s__Alteromonas_macleodii |
| k__Bacteria;p__Proteobacteria;c__Gammaproteobacteria;o__Alteromonadales;f__Alteromonadaceae;g__Glaciecola;s__ |
| k__Bacteria;p__Proteobacteria;c__Gammaproteobacteria;o__Alteromonadales;f__Pseudoalteromonadaceae;g__Pseudoalteromonas |
| k__Bacteria;p__Proteobacteria;c__Gammaproteobacteria;o__Alteromonadales;f__Pseudoalteromonadaceae;g__Pseudoalteromonas;s__Pseudoalteromonas_shioyasakiensis |
| k__Bacteria;p__Proteobacteria;c__Gammaproteobacteria;o__Alteromonadales;f__unidentified_Alteromonadales;g__Marinobacter;s__Marinobacter_alkaliphilus |
| k__Bacteria;p__Proteobacteria;c__Gammaproteobacteria;o__Enterobacteriales;f__Enterobacteriaceae;g__Citrobacter |
| k__Bacteria;p__Proteobacteria;c__Gammaproteobacteria;o__Enterobacteriales;f__Enterobacteriaceae;g__Klebsiella |
| k__Bacteria;p__Proteobacteria;c__Gammaproteobacteria;o__Enterobacteriales;f__Enterobacteriaceae;g__Klebsiella |
| k__Bacteria;p__Proteobacteria;c__Gammaproteobacteria;o__Oceanospirillales;f__Halomonadaceae;g__Halomonas |
| k__Bacteria;p__Proteobacteria;c__Gammaproteobacteria;o__Oceanospirillales;f__Halomonadaceae;g__Halomonas;s__Halomonas_titanicae |
| k__Bacteria;p__Proteobacteria;c__Gammaproteobacteria;o__Oceanospirillales;f__unidentified_Oceanospirillales;g__Marinobacterium;s__Marinobacterium_marisflavi |
| k__Bacteria;p__Proteobacteria;c__Gammaproteobacteria;o__Pasteurellales;f__Pasteurellaceae;g__Actinobacillus;s__[Haemophilus]_parasuis |
| k__Bacteria;p__Proteobacteria;c__Gammaproteobacteria;o__Pasteurellales;f__Pasteurellaceae;g__Actinobacillus;s__Haemophilus_parahaemolyticus |
| k__Bacteria;p__Proteobacteria;c__Gammaproteobacteria;o__Pasteurellales;f__Pasteurellaceae;g__Haemophilus |
| k__Bacteria;p__Proteobacteria;c__Gammaproteobacteria;o__Pasteurellales;f__Pasteurellaceae;g__Haemophilus;s__Haemophilus_parainfluenzae |
| k__Bacteria;p__Proteobacteria;c__Gammaproteobacteria;o__Pasteurellales;f__Pasteurellaceae;g__Haemophilus;s__Haemophilus_parainfluenzae |
| k__Bacteria;p__Proteobacteria;c__Gammaproteobacteria;o__Pseudomonadales;f__Moraxellaceae;g__Acinetobacter |
| k__Bacteria;p__Proteobacteria;c__Gammaproteobacteria;o__Pseudomonadales;f__Moraxellaceae;g__Acinetobacter |
| k__Bacteria;p__Proteobacteria;c__Gammaproteobacteria;o__Pseudomonadales;f__Moraxellaceae;g__Acinetobacter |
| k__Bacteria;p__Proteobacteria;c__Gammaproteobacteria;o__Pseudomonadales;f__Moraxellaceae;g__Acinetobacter;s__Acinetobacter_junii |
| k__Bacteria;p__Proteobacteria;c__Gammaproteobacteria;o__Pseudomonadales;f__Moraxellaceae;g__Acinetobacter;s__Acinetobacter_lwoffii |
| k__Bacteria;p__Proteobacteria;c__Gammaproteobacteria;o__Pseudomonadales;f__Moraxellaceae;g__Acinetobacter;s__Acinetobacter_ursingii |
| k__Bacteria;p__Proteobacteria;c__Gammaproteobacteria;o__Pseudomonadales;f__Moraxellaceae;g__Enhydrobacter;s__Moraxella_osloensis |
| k__Bacteria;p__Proteobacteria;c__Gammaproteobacteria;o__Pseudomonadales;f__Moraxellaceae;g__Psychrobacter |
| k__Bacteria;p__Proteobacteria;c__Gammaproteobacteria;o__Pseudomonadales;f__Pseudomonadaceae;g__Pseudomonas |
| k__Bacteria;p__Proteobacteria;c__Gammaproteobacteria;o__Pseudomonadales;f__Pseudomonadaceae;g__Pseudomonas;s__Pseudomonas_aeruginosa |
| k__Bacteria;p__Proteobacteria;c__Gammaproteobacteria;o__Pseudomonadales;f__Pseudomonadaceae;g__Pseudomonas;s__Pseudomonas_azotoformans |
| k__Bacteria;p__Proteobacteria;c__Gammaproteobacteria;o__Pseudomonadales;f__Pseudomonadaceae;g__Pseudomonas;s__Pseudomonas_xanthomarina |
| k__Bacteria;p__Proteobacteria;c__Gammaproteobacteria;o__unidentified_Gammaproteobacteria;f__Neisseriaceae;g__Neisseria |
| k__Bacteria;p__Proteobacteria;c__Gammaproteobacteria;o__unidentified_Gammaproteobacteria;f__Neisseriaceae;g__Neisseria;s__Neisseria_flava |
| k__Bacteria;p__Proteobacteria;c__Gammaproteobacteria;o__unidentified_Gammaproteobacteria;f__Rhodocyclaceae;g__Thauera |
| k__Bacteria;p__Proteobacteria;c__Gammaproteobacteria;o__Vibrionales;f__Vibrionaceae |
| k__Bacteria;p__Proteobacteria;c__Gammaproteobacteria;o__Vibrionales;f__Vibrionaceae;g__Photobacterium |
| k__Bacteria;p__Proteobacteria;c__Gammaproteobacteria;o__Vibrionales;f__Vibrionaceae;g__Photobacterium |
| k__Bacteria;p__Proteobacteria;c__Gammaproteobacteria;o__Vibrionales;f__Vibrionaceae;g__Photobacterium |
| k__Bacteria;p__Proteobacteria;c__Gammaproteobacteria;o__Vibrionales;f__Vibrionaceae;g__Photobacterium |
| k__Bacteria;p__Proteobacteria;c__Gammaproteobacteria;o__Vibrionales;f__Vibrionaceae;g__Photobacterium |
| k__Bacteria;p__Proteobacteria;c__Gammaproteobacteria;o__Vibrionales;f__Vibrionaceae;g__Photobacterium |
| k__Bacteria;p__Proteobacteria;c__Gammaproteobacteria;o__Vibrionales;f__Vibrionaceae;g__Photobacterium;s__Photobacterium_aphoticum |
| k__Bacteria;p__Proteobacteria;c__Gammaproteobacteria;o__Vibrionales;f__Vibrionaceae;g__Photobacterium;s__Photobacterium_aphoticum |
| k__Bacteria;p__Proteobacteria;c__Gammaproteobacteria;o__Vibrionales;f__Vibrionaceae;g__Photobacterium;s__Photobacterium_sp_PaH103b |
| k__Bacteria;p__Proteobacteria;c__Gammaproteobacteria;o__Vibrionales;f__Vibrionaceae;g__Photobacterium;s__Photobacterium_sp_PaH103b |
| k__Bacteria;p__Proteobacteria;c__Gammaproteobacteria;o__Vibrionales;f__Vibrionaceae;g__Photobacterium;s__Photobacterium_sp_PaH103b |
| k__Bacteria;p__Proteobacteria;c__Gammaproteobacteria;o__Vibrionales;f__Vibrionaceae;g__Photobacterium;s__Photobacterium_sp_PaH103b |
| k__Bacteria;p__Proteobacteria;c__Gammaproteobacteria;o__Vibrionales;f__Vibrionaceae;g__Vibrio |
| k__Bacteria;p__Proteobacteria;c__Gammaproteobacteria;o__Vibrionales;f__Vibrionaceae;g__Vibrio |
| k__Bacteria;p__Proteobacteria;c__Gammaproteobacteria;o__Vibrionales;f__Vibrionaceae;g__Vibrio |
| k__Bacteria;p__Proteobacteria;c__Gammaproteobacteria;o__Vibrionales;f__Vibrionaceae;g__Vibrio |
| k__Bacteria;p__Proteobacteria;c__Gammaproteobacteria;o__Vibrionales;f__Vibrionaceae;g__Vibrio;s__Vibrio_ponticus |
| k__Bacteria;p__Proteobacteria;c__Gammaproteobacteria;o__Xanthomonadales;f__Xanthomonadaceae;g__Arenimonas;s__ |
| k__Bacteria;p__Proteobacteria;c__Gammaproteobacteria;o__Xanthomonadales;f__Xanthomonadaceae;g__Arenimonas;s__ |
| k__Bacteria;p__Proteobacteria;c__Gammaproteobacteria;o__Xanthomonadales;f__Xanthomonadaceae;g__Arenimonas;s__ |
| k__Bacteria;p__Proteobacteria;c__Gammaproteobacteria;o__Xanthomonadales;f__Xanthomonadaceae;g__Stenotrophomonas |
| k__Bacteria;p__Proteobacteria;c__Gammaproteobacteria;o__Xanthomonadales;f__Xanthomonadaceae;g__Stenotrophomonas |
| k__Bacteria;p__Proteobacteria;c__Gammaproteobacteria;o__Xanthomonadales;f__Xanthomonadaceae;g__Stenotrophomonas;s__ |
| k__Bacteria;p__Tenericutes;c__Mollicutes;o__Mycoplasmatales;f__Mycoplasmataceae;g__Mycoplasma;s__Mycoplasma_hyorhinis |
| k__Bacteria;p__Verrucomicrobia;c__Verrucomicrobiae;o__Verrucomicrobiales;f__Akkermansiaceae;g__Akkermansia |
| k__Bacteria;p__Verrucomicrobia;c__Verrucomicrobiae;o__Verrucomicrobiales;f__Akkermansiaceae;g__Akkermansia;s__ |
| k__Bacteria;p__Verrucomicrobia;c__Verrucomicrobiae;o__Verrucomicrobiales;f__Rubritaleaceae;g__Rubritalea;s__ |
| k__Bacteria;p__Verrucomicrobia;c__Verrucomicrobiae;o__Verrucomicrobiales;f__Rubritaleaceae;g__Rubritalea;s__ |
| # animal_parasites_or_symbionts (63 records): |
| k__Bacteria;p__Actinobacteria;c__unidentified_Actinobacteria;o__Actinomycetales;f__Actinomycetaceae;g__Actinomyces;s__Actinomyces_odontolyticus |
| k__Bacteria;p__Actinobacteria;c__unidentified_Actinobacteria;o__Bifidobacteriales;f__Bifidobacteriaceae;g__Bifidobacterium;s__Bifidobacterium_animalis |
| k__Bacteria;p__Actinobacteria;c__unidentified_Actinobacteria;o__Bifidobacteriales;f__Bifidobacteriaceae;g__Bifidobacterium;s__Bifidobacterium_bifidum |
| k__Bacteria;p__Actinobacteria;c__unidentified_Actinobacteria;o__Bifidobacteriales;f__Bifidobacteriaceae;g__Bifidobacterium;s__Bifidobacterium_breve |
| k__Bacteria;p__Actinobacteria;c__unidentified_Actinobacteria;o__Bifidobacteriales;f__Bifidobacteriaceae;g__Bifidobacterium;s__Bifidobacterium_pseudocatenulatum |
| k__Bacteria;p__Bacteroidetes;c__Bacteroidia;o__Bacteroidales;f__Bacteroidaceae;g__Bacteroides;s__Bacteroides_caccae |
| k__Bacteria;p__Bacteroidetes;c__Bacteroidia;o__Bacteroidales;f__Bacteroidaceae;g__Bacteroides;s__Bacteroides_dorei |
| k__Bacteria;p__Bacteroidetes;c__Bacteroidia;o__Bacteroidales;f__Bacteroidaceae;g__Bacteroides;s__Bacteroides_dorei |
| k__Bacteria;p__Bacteroidetes;c__Bacteroidia;o__Bacteroidales;f__Bacteroidaceae;g__Bacteroides;s__Bacteroides_eggerthii |
| k__Bacteria;p__Bacteroidetes;c__Bacteroidia;o__Bacteroidales;f__Bacteroidaceae;g__Bacteroides;s__Bacteroides_fragilis |
| k__Bacteria;p__Bacteroidetes;c__Bacteroidia;o__Bacteroidales;f__Bacteroidaceae;g__Bacteroides;s__Bacteroides_stercoris |
| k__Bacteria;p__Bacteroidetes;c__Bacteroidia;o__Bacteroidales;f__Bacteroidaceae;g__Bacteroides;s__Bacteroides_thetaiotaomicron |
| k__Bacteria;p__Bacteroidetes;c__Bacteroidia;o__Bacteroidales;f__Bacteroidaceae;g__Bacteroides;s__Bacteroides_uniformis |
| k__Bacteria;p__Bacteroidetes;c__Bacteroidia;o__Bacteroidales;f__Bacteroidaceae;g__Bacteroides;s__Bacteroides_vulgatus |
| k__Bacteria;p__Bacteroidetes;c__Bacteroidia;o__Bacteroidales;f__Prevotellaceae;g__unidentified_Prevotellaceae;s__Prevotella_histicola |
| k__Bacteria;p__Bacteroidetes;c__Bacteroidia;o__Bacteroidales;f__Prevotellaceae;g__unidentified_Prevotellaceae;s__Prevotella_stercorea |
| k__Bacteria;p__Bacteroidetes;c__Bacteroidia;o__Bacteroidales;f__Rikenellaceae;g__Alistipes;s__Faecalibacterium_prausnitzii |
| k__Bacteria;p__Bacteroidetes;c__Bacteroidia;o__Bacteroidales;f__Tannerellaceae;g__Parabacteroides;s__Parabacteroides_distasonis |
| k__Bacteria;p__Bacteroidetes;c__Bacteroidia;o__Bacteroidales;f__Tannerellaceae;g__Parabacteroides;s__Parabacteroides_merdae |
| k__Bacteria;p__Firmicutes;c__Bacilli;o__Bacillales;f__Bacillaceae;g__Bacillus;s__Bacillus_anthracis |
| k__Bacteria;p__Firmicutes;c__Bacilli;o__Lactobacillales;f__Lactobacillaceae;g__Lactobacillus;s__Lactobacillus_reuteri |
| k__Bacteria;p__Firmicutes;c__Clostridia;o__Clostridiales;f__Lachnospiraceae;g__Dorea;s__Dorea_formicigenerans |
| k__Bacteria;p__Firmicutes;c__Clostridia;o__Clostridiales;f__Lachnospiraceae;g__Roseburia |
| k__Bacteria;p__Firmicutes;c__Clostridia;o__Clostridiales;f__Lachnospiraceae;g__Roseburia;s__ |
| k__Bacteria;p__Firmicutes;c__Clostridia;o__Clostridiales;f__Lachnospiraceae;g__Roseburia;s__Roseburia_inulinivorans |
| k__Bacteria;p__Firmicutes;c__Clostridia;o__Clostridiales;f__Lachnospiraceae;g__unidentified_Lachnospiraceae;s__[Eubacterium]_hallii |
| k__Bacteria;p__Firmicutes;c__Clostridia;o__Clostridiales;f__Lachnospiraceae;g__unidentified_Lachnospiraceae;s__[Ruminococcus]_torques |
| k__Bacteria;p__Firmicutes;c__Clostridia;o__Clostridiales;f__Lachnospiraceae;g__unidentified_Lachnospiraceae;s__Clostridium_sp_L2-50 |
| k__Bacteria;p__Firmicutes;c__Clostridia;o__Clostridiales;f__Lachnospiraceae;g__unidentified_Lachnospiraceae;s__Coprococcus_catus |
| k__Bacteria;p__Firmicutes;c__Clostridia;o__Clostridiales;f__Ruminococcaceae;g__unidentified_Ruminococcaceae;s__Ruminococcus_bromii |
| k__Bacteria;p__Firmicutes;c__Clostridia;o__Clostridiales;f__unidentified_Clostridiales;g__unidentified_Clostridiales;s__Clostridium_butyricum |
| k__Bacteria;p__Firmicutes;c__Clostridia;o__Clostridiales;f__unidentified_Clostridiales;g__unidentified_Clostridiales;s__Clostridium_perfringens |
| k__Bacteria;p__Firmicutes;c__Erysipelotrichia;o__Erysipelotrichales;f__Erysipelotrichaceae;g__unidentified_Erysipelotrichaceae;s__[Clostridium]_innocuum |
| k__Bacteria;p__Firmicutes;c__Negativicutes;o__Selenomonadales;f__Acidaminococcaceae;g__Acidaminococcus;s__Acidaminococcus_intestini |
| k__Bacteria;p__Proteobacteria;c__Gammaproteobacteria;o__Enterobacteriales;f__Enterobacteriaceae;g__Citrobacter |
| k__Bacteria;p__Proteobacteria;c__Gammaproteobacteria;o__Enterobacteriales;f__Enterobacteriaceae;g__Enterobacter;s__Escherichia_vulneris |
| k__Bacteria;p__Proteobacteria;c__Gammaproteobacteria;o__Enterobacteriales;f__Enterobacteriaceae;g__Proteus;s__Proteus_mirabilis |
| k__Bacteria;p__Proteobacteria;c__Gammaproteobacteria;o__Enterobacteriales;f__Enterobacteriaceae;g__Serratia;s__Serratia_marcescens |
| k__Bacteria;p__Proteobacteria;c__Gammaproteobacteria;o__Pasteurellales;f__Pasteurellaceae;g__Actinobacillus;s__[Haemophilus]_parasuis |
| k__Bacteria;p__Proteobacteria;c__Gammaproteobacteria;o__Pasteurellales;f__Pasteurellaceae;g__Actinobacillus;s__Haemophilus_parahaemolyticus |
| k__Bacteria;p__Proteobacteria;c__Gammaproteobacteria;o__Pasteurellales;f__Pasteurellaceae;g__Aggregatibacter;s__Aggregatibacter_segnis |
| k__Bacteria;p__Proteobacteria;c__Gammaproteobacteria;o__Pasteurellales;f__Pasteurellaceae;g__Haemophilus |
| k__Bacteria;p__Proteobacteria;c__Gammaproteobacteria;o__Pasteurellales;f__Pasteurellaceae;g__Haemophilus;s__Haemophilus_parainfluenzae |
| k__Bacteria;p__Proteobacteria;c__Gammaproteobacteria;o__Pasteurellales;f__Pasteurellaceae;g__Haemophilus;s__Haemophilus_parainfluenzae |
| k__Bacteria;p__Proteobacteria;c__Gammaproteobacteria;o__Pseudomonadales;f__Moraxellaceae;g__Acinetobacter |
| k__Bacteria;p__Proteobacteria;c__Gammaproteobacteria;o__Pseudomonadales;f__Moraxellaceae;g__Acinetobacter |
| k__Bacteria;p__Proteobacteria;c__Gammaproteobacteria;o__Pseudomonadales;f__Moraxellaceae;g__Acinetobacter |
| k__Bacteria;p__Proteobacteria;c__Gammaproteobacteria;o__Pseudomonadales;f__Moraxellaceae;g__Acinetobacter;s__Acinetobacter_junii |
| k__Bacteria;p__Proteobacteria;c__Gammaproteobacteria;o__Pseudomonadales;f__Moraxellaceae;g__Acinetobacter;s__Acinetobacter_lwoffii |
| k__Bacteria;p__Proteobacteria;c__Gammaproteobacteria;o__Pseudomonadales;f__Moraxellaceae;g__Acinetobacter;s__Acinetobacter_ursingii |
| k__Bacteria;p__Proteobacteria;c__Gammaproteobacteria;o__Pseudomonadales;f__Moraxellaceae;g__Enhydrobacter;s__Moraxella_osloensis |
| k__Bacteria;p__Proteobacteria;c__Gammaproteobacteria;o__Pseudomonadales;f__Moraxellaceae;g__Moraxella |
| k__Bacteria;p__Proteobacteria;c__Gammaproteobacteria;o__Pseudomonadales;f__Pseudomonadaceae;g__Pseudomonas;s__Pseudomonas_aeruginosa |
| k__Bacteria;p__Proteobacteria;c__Gammaproteobacteria;o__unidentified_Gammaproteobacteria;f__Burkholderiaceae;g__Alcaligenes;s__Alcaligenes_faecalis |
| k__Bacteria;p__Proteobacteria;c__Gammaproteobacteria;o__unidentified_Gammaproteobacteria;f__Burkholderiaceae;g__unidentified_Burkholderiaceae;s__Burkholderiales_bacterium_X4 |
| k__Bacteria;p__Proteobacteria;c__Gammaproteobacteria;o__unidentified_Gammaproteobacteria;f__Francisellaceae;g__Francisella |
| k__Bacteria;p__Proteobacteria;c__Gammaproteobacteria;o__Xanthomonadales;f__Xanthomonadaceae;g__Stenotrophomonas |
| k__Bacteria;p__Proteobacteria;c__Gammaproteobacteria;o__Xanthomonadales;f__Xanthomonadaceae;g__Stenotrophomonas |
| k__Bacteria;p__Proteobacteria;c__Gammaproteobacteria;o__Xanthomonadales;f__Xanthomonadaceae;g__Stenotrophomonas;s__ |
| k__Bacteria;p__unidentified_Bacteria;c__unidentified_Bacteria;o__Campylobacterales;f__Campylobacteraceae;g__Campylobacter;s__Campylobacter_concisus |
| k__Bacteria;p__unidentified_Bacteria;c__unidentified_Bacteria;o__Campylobacterales;f__Campylobacteraceae;g__Campylobacter;s__Campylobacter_showae |
| k__Bacteria;p__unidentified_Bacteria;c__unidentified_Bacteria;o__Campylobacterales;f__Helicobacteraceae;g__Helicobacter;s__Helicobacter_pylori |
| k__Bacteria;p__unidentified_Bacteria;c__unidentified_Bacteria;o__Campylobacterales;f__Helicobacteraceae;g__Helicobacter;s__Helicobacter_rodentium |

| **Supplementary Table 6. The KEGG pathway enrichment of Profile A** | | | | | | | |
| --- | --- | --- | --- | --- | --- | --- | --- |
| KEGG_A_class | KEGG_B_class | Pathway | Profile6 (352) | All (23075) | Pvalue | Qvalue | Pathway ID |
| Human Diseases | Immune diseases | Asthma | 28 | 271 | 1.69E-15 | 4.50E-13 | ko05310 |
| Human Diseases | Infectious diseases | Measles | 35 | 699 | 8.23E-10 | 1.09E-07 | ko05162 |
| Environmental Information Processing | Signal transduction | NF-kappa B signaling pathway | 26 | 450 | 7.69E-09 | 6.82E-07 | ko04064 |
| Human Diseases | Infectious diseases | Hepatitis C | 26 | 590 | 1.52E-06 | 9.73E-05 | ko05160 |
| Human Diseases | Immune diseases | Primary immunodeficiency | 14 | 197 | 2.19E-06 | 9.73E-05 | ko05340 |
| Human Diseases | Infectious diseases | Influenza A | 30 | 759 | 2.19E-06 | 9.73E-05 | ko05164 |
| Organismal Systems | Development | Osteoclast differentiation | 23 | 502 | 3.25E-06 | 1.10E-04 | ko04380 |
| Environmental Information Processing | Signal transduction | TNF signaling pathway | 19 | 361 | 3.30E-06 | 1.10E-04 | ko04668 |
| Human Diseases | Endocrine and metabolic diseases | Type II diabetes mellitus | 13 | 181 | 4.49E-06 | 1.33E-04 | ko04930 |
| Human Diseases | Immune diseases | Systemic lupus erythematosus | 17 | 306 | 5.34E-06 | 1.42E-04 | ko05322 |
| Human Diseases | Infectious diseases | Herpes simplex infection | 32 | 889 | 6.99E-06 | 1.69E-04 | ko05168 |
| Human Diseases | Immune diseases | Autoimmune thyroid disease | 18 | 360 | 1.20E-05 | 2.56E-04 | ko05320 |
| Human Diseases | Immune diseases | Allograft rejection | 18 | 361 | 1.25E-05 | 2.56E-04 | ko05330 |
| Human Diseases | Infectious diseases | Staphylococcus aureus infection | 14 | 237 | 1.83E-05 | 3.35E-04 | ko05150 |
| Organismal Systems | Endocrine system | Prolactin signaling pathway | 16 | 303 | 1.89E-05 | 3.35E-04 | ko04917 |
| Organismal Systems | Immune system | Intestinal immune network for IgA production | 13 | 239 | 8.44E-05 | 1.40E-03 | ko04672 |
| Human Diseases | Infectious diseases | Epstein-Barr virus infection | 32 | 1040 | 0.000142 | 2.23E-03 | ko05169 |
| Metabolism | Biosynthesis of other secondary metabolites | Streptomycin biosynthesis | 5 | 42 | 0.00043 | 5.88E-03 | ko00521 |
| Organismal Systems | Immune system | RIG-I-like receptor signaling pathway | 12 | 247 | 0.000441 | 5.88E-03 | ko04622 |
| Human Diseases | Infectious diseases | Leishmaniasis | 15 | 359 | 0.000448 | 5.88E-03 | ko05140 |
| Human Diseases | Cancers | Transcriptional misregulation in cancers | 22 | 651 | 0.000464 | 5.88E-03 | ko05202 |
| Organismal Systems | Immune system | NOD-like receptor signaling pathway | 21 | 619 | 0.000591 | 7.15E-03 | ko04621 |
| Human Diseases | Cardiovascular diseases | Viral myocarditis | 18 | 501 | 0.000752 | 8.70E-03 | ko05416 |
| Human Diseases | Immune diseases | Inflammatiory bowel disease (IBD) | 10 | 194 | 0.00083 | 9.20E-03 | ko05321 |
| Organismal Systems | Endocrine system | Adipocytokine signaling pathway | 15 | 390 | 0.001041 | 1.09E-02 | ko04920 |
| Metabolism | Biosynthesis of other secondary metabolites | Neomycin, kanamycin and gentamicin biosynthesis | 4 | 30 | 0.001066 | 1.09E-02 | ko00524 |
| Organismal Systems | Immune system | Th1 and Th2 cell differentiation | 14 | 374 | 0.001946 | 1.92E-02 | ko04658 |
| Organismal Systems | Immune system | Hematopoietic cell lineage | 17 | 507 | 0.002173 | 2.06E-02 | ko04640 |
| Human Diseases | Infectious diseases | African trypanosomiasis | 9 | 193 | 0.002948 | 2.67E-02 | ko05143 |
| Genetic Information Processing | Folding, sorting and degradation | Ubiquitin mediated proteolysis | 16 | 479 | 0.003011 | 2.67E-02 | ko04120 |
| Organismal Systems | Endocrine system | Insulin signaling pathway | 19 | 621 | 0.003394 | 2.91E-02 | ko04910 |
| Cellular Processes | Transport and catabolism | Phagosome | 27 | 1009 | 0.003513 | 2.92E-02 | ko04145 |
| Environmental Information Processing | Signal transduction | HIF-1 signaling pathway | 15 | 449 | 0.004007 | 3.23E-02 | ko04066 |
| Organismal Systems | Immune system | Toll-like receptor signaling pathway | 11 | 286 | 0.004696 | 3.67E-02 | ko04620 |
| Organismal Systems | Immune system | Th17 cell differentiation | 16 | 509 | 0.005374 | 4.08E-02 | ko04659 |
| Human Diseases | Endocrine and metabolic diseases | Non-alcoholic fatty liver disease (NAFLD) | 16 | 533 | 0.008198 | 6.06E-02 | ko04932 |
| Human Diseases | Immune diseases | Rheumatoid arthritis | 12 | 358 | 0.009309 | 6.67E-02 | ko05323 |
| Environmental Information Processing | Signaling molecules and interaction | Cell adhesion molecules (CAMs) | 16 | 542 | 0.009523 | 6.67E-02 | ko04514 |
| Environmental Information Processing | Signal transduction | Calcium signaling pathway | 19 | 689 | 0.009923 | 6.77E-02 | ko04020 |
| Organismal Systems | Immune system | Cytosolic DNA-sensing pathway | 6 | 121 | 0.010722 | 7.13E-02 | ko04623 |
| Metabolism | Carbohydrate metabolism | Amino sugar and nucleotide sugar metabolism | 12 | 369 | 0.011625 | 7.54E-02 | ko00520 |
| Metabolism | Carbohydrate metabolism | Fructose and mannose metabolism | 7 | 165 | 0.01357 | 8.43E-02 | ko00051 |
| Human Diseases | Immune diseases | Graft-versus-host disease | 9 | 246 | 0.013635 | 8.43E-02 | ko05332 |
| Metabolism | Metabolism of cofactors and vitamins | Nicotinate and nicotinamide metabolism | 7 | 174 | 0.017674 | 1.07E-01 | ko00760 |
| Human Diseases | Infectious diseases | Toxoplasmosis | 14 | 488 | 0.018404 | 1.09E-01 | ko05145 |
| Human Diseases | Endocrine and metabolic diseases | Type I diabetes mellitus | 9 | 262 | 0.019706 | 1.14E-01 | ko04940 |
| Environmental Information Processing | Signal transduction | Jak-STAT signaling pathway | 14 | 497 | 0.021144 | 1.18E-01 | ko04630 |
| Human Diseases | Endocrine and metabolic diseases | Insulin resistance | 15 | 546 | 0.021226 | 1.18E-01 | ko04931 |
| Human Diseases | Cancers | Viral carcinogenesis | 22 | 908 | 0.022731 | 1.23E-01 | ko05203 |
| Metabolism | Nucleotide metabolism | Pyrimidine metabolism | 9 | 273 | 0.024881 | 1.32E-01 | ko00240 |
| Organismal Systems | Immune system | Fc epsilon RI signaling pathway | 11 | 371 | 0.027924 | 1.46E-01 | ko04664 |
| Organismal Systems | Immune system | Antigen processing and presentation | 13 | 475 | 0.031435 | 1.61E-01 | ko04612 |
| Metabolism | Metabolism of terpenoids and polyketides | Terpenoid backbone biosynthesis | 4 | 79 | 0.032745 | 1.64E-01 | ko00900 |
| Metabolism | Carbohydrate metabolism | Galactose metabolism | 7 | 203 | 0.036692 | 1.81E-01 | ko00052 |
| Metabolism | Lipid metabolism | alpha-Linolenic acid metabolism | 5 | 123 | 0.040451 | 1.96E-01 | ko00592 |
| Metabolism | Amino acid metabolism | Valine, leucine and isoleucine biosynthesis | 1 | 3 | 0.045071 | 2.14E-01 | ko00290 |
| Genetic Information Processing | Replication and repair | Homologous recombination | 3 | 53 | 0.047114 | 2.20E-01 | ko03440 |
| Human Diseases | Infectious diseases | Tuberculosis | 21 | 946 | 0.056046 | 2.57E-01 | ko05152 |
| Organismal Systems | Immune system | Natural killer cell mediated cytotoxicity | 15 | 628 | 0.059313 | 2.67E-01 | ko04650 |
| Organismal Systems | Immune system | IL-17 signaling pathway | 8 | 276 | 0.06202 | 2.71E-01 | ko04657 |
| Human Diseases | Cancers | Central carbon metabolism in cancer | 9 | 325 | 0.062523 | 2.71E-01 | ko05230 |
| Human Diseases | Infectious diseases | Malaria | 4 | 98 | 0.063163 | 2.71E-01 | ko05144 |
| Human Diseases | Cancers | Small cell lung cancer | 11 | 432 | 0.068578 | 2.90E-01 | ko05222 |
| Human Diseases | Endocrine and metabolic diseases | AGE-RAGE signaling pathway in diabetic complications | 13 | 539 | 0.071167 | 2.96E-01 | ko04933 |
| Cellular Processes | Cell growth and death | Apoptosis | 16 | 704 | 0.074606 | 2.99E-01 | ko04210 |
| Cellular Processes | Cell growth and death | Apoptosis - multiple species | 4 | 104 | 0.07504 | 2.99E-01 | ko04215 |
| Human Diseases | Infectious diseases | HTLV-I infection | 21 | 981 | 0.075437 | 2.99E-01 | ko05166 |
| Organismal Systems | Immune system | B cell receptor signaling pathway | 11 | 441 | 0.076752 | 3.00E-01 | ko04662 |
| Environmental Information Processing | Signaling molecules and interaction | Cytokine-cytokine receptor interaction | 10 | 405 | 0.093231 | 3.59E-01 | ko04060 |
| Metabolism | Metabolism of other amino acids | Phosphonate and phosphinate metabolism | 2 | 34 | 0.094643 | 3.60E-01 | ko00440 |
| Human Diseases | Cardiovascular diseases | Dilated cardiomyopathy (DCM) | 9 | 370 | 0.114911 | 4.31E-01 | ko05414 |
| Metabolism | Carbohydrate metabolism | Starch and sucrose metabolism | 5 | 169 | 0.117216 | 4.33E-01 | ko00500 |
| Genetic Information Processing | Folding, sorting and degradation | Protein processing in endoplasmic reticulum | 16 | 765 | 0.127242 | 4.62E-01 | ko04141 |
| Metabolism | Metabolism of terpenoids and polyketides | Biosynthesis of ansamycins | 1 | 9 | 0.129227 | 4.62E-01 | ko01051 |
| Organismal Systems | Immune system | Toll and Imd signaling pathway | 5 | 175 | 0.130337 | 4.62E-01 | ko04624 |
| Cellular Processes | Cellular community - prokaryotes | Quorum sensing | 3 | 84 | 0.137047 | 4.74E-01 | ko02024 |
| Metabolism | Lipid metabolism | Primary bile acid biosynthesis | 4 | 130 | 0.13791 | 4.74E-01 | ko00120 |
| Human Diseases | Drug resistance | Platinum drug resistance | 7 | 281 | 0.139787 | 4.74E-01 | ko01524 |
| Metabolism | Carbohydrate metabolism | Pentose phosphate pathway | 4 | 131 | 0.14066 | 4.74E-01 | ko00030 |
| Metabolism | Xenobiotics biodegradation and metabolism | Bisphenol degradation | 1 | 10 | 0.142515 | 4.74E-01 | ko00363 |
| Metabolism | Energy metabolism | Nitrogen metabolism | 2 | 45 | 0.150154 | 4.86E-01 | ko00910 |
| Human Diseases | Infectious diseases | Hepatitis B | 13 | 616 | 0.150409 | 4.86E-01 | ko05161 |
| Organismal Systems | Immune system | Fc gamma R-mediated phagocytosis | 12 | 562 | 0.153374 | 4.86E-01 | ko04666 |
| Human Diseases | Infectious diseases | Pertussis | 5 | 185 | 0.153594 | 4.86E-01 | ko05133 |
| Metabolism | Glycan biosynthesis and metabolism | Glycosphingolipid biosynthesis - globo and isoglobo series | 5 | 186 | 0.15601 | 4.88E-01 | ko00603 |
| Human Diseases | Infectious diseases | Amoebiasis | 12 | 568 | 0.161474 | 4.99E-01 | ko05146 |
| Metabolism | Nucleotide metabolism | Purine metabolism | 11 | 516 | 0.167638 | 5.09E-01 | ko00230 |
| Cellular Processes | Cell growth and death | p53 signaling pathway | 5 | 191 | 0.16832 | 5.09E-01 | ko04115 |
| Metabolism | Glycan biosynthesis and metabolism | Glycosaminoglycan biosynthesis - heparan sulfate / heparin | 2 | 50 | 0.177119 | 5.29E-01 | ko00534 |
| Environmental Information Processing | Signal transduction | Hippo signaling pathway - multiple species | 3 | 100 | 0.196367 | 5.77E-01 | ko04392 |
| Environmental Information Processing | Signal transduction | Two-component system | 2 | 54 | 0.199187 | 5.77E-01 | ko02020 |
| Organismal Systems | Endocrine system | PPAR signaling pathway | 9 | 423 | 0.199721 | 5.77E-01 | ko03320 |
| Metabolism | Lipid metabolism | Linoleic acid metabolism | 4 | 155 | 0.212405 | 6.08E-01 | ko00591 |
| Environmental Information Processing | Membrane transport | ABC transporters | 8 | 375 | 0.21583 | 6.11E-01 | ko02010 |
| Human Diseases | Infectious diseases | Chagas disease (American trypanosomiasis) | 8 | 379 | 0.223917 | 6.21E-01 | ko05142 |
| Metabolism | Lipid metabolism | Fatty acid biosynthesis | 3 | 107 | 0.224027 | 6.21E-01 | ko00061 |
| Organismal Systems | Digestive system | Carbohydrate digestion and absorption | 5 | 213 | 0.226514 | 6.21E-01 | ko04973 |
| Organismal Systems | Immune system | Complement and coagulation cascades | 3 | 112 | 0.244226 | 6.63E-01 | ko04610 |
| Environmental Information Processing | Signal transduction | Phospholipase D signaling pathway | 13 | 690 | 0.257133 | 6.91E-01 | ko04072 |
| Metabolism | Glycan biosynthesis and metabolism | Glycosphingolipid biosynthesis - ganglio series | 3 | 116 | 0.260588 | 6.93E-01 | ko00604 |
| Human Diseases | Infectious diseases | Salmonella infection | 7 | 342 | 0.268359 | 7.07E-01 | ko05132 |
| Organismal Systems | Nervous system | Retrograde endocannabinoid signaling | 6 | 289 | 0.280109 | 7.30E-01 | ko04723 |
| Metabolism | Glycan biosynthesis and metabolism | Glycosaminoglycan biosynthesis - keratan sulfate | 2 | 70 | 0.289388 | 7.47E-01 | ko00533 |
| Genetic Information Processing | Folding, sorting and degradation | RNA degradation | 5 | 237 | 0.295524 | 7.55E-01 | ko03018 |
| Metabolism | Metabolism of cofactors and vitamins | Ubiquinone and other terpenoid-quinone biosynthesis | 1 | 23 | 0.297935 | 7.55E-01 | ko00130 |
| Metabolism | Global and overview maps | Carbon metabolism | 10 | 538 | 0.307355 | 7.65E-01 | ko01200 |
| Cellular Processes | Transport and catabolism | Peroxisome | 8 | 420 | 0.312194 | 7.65E-01 | ko04146 |
| Environmental Information Processing | Signal transduction | MAPK signaling pathway | 14 | 785 | 0.313043 | 7.65E-01 | ko04010 |
| Environmental Information Processing | Signal transduction | PI3K-Akt signaling pathway | 22 | 1283 | 0.316103 | 7.65E-01 | ko04151 |
| Environmental Information Processing | Signal transduction | MAPK signaling pathway - yeast | 3 | 130 | 0.318683 | 7.65E-01 | ko04011 |
| Genetic Information Processing | Replication and repair | Non-homologous end-joining | 1 | 25 | 0.319213 | 7.65E-01 | ko03450 |
| Cellular Processes | Cell growth and death | Cell cycle - Caulobacter | 1 | 27 | 0.339847 | 8.07E-01 | ko04112 |
| Metabolism | Xenobiotics biodegradation and metabolism | Aminobenzoate degradation | 1 | 28 | 0.349929 | 8.24E-01 | ko00627 |
| Human Diseases | Cancers | Pathways in cancer | 25 | 1512 | 0.367353 | 8.45E-01 | ko05200 |
| Metabolism | Lipid metabolism | Ether lipid metabolism | 4 | 201 | 0.36775 | 8.45E-01 | ko00565 |
| Metabolism | Glycan biosynthesis and metabolism | Mucin type O-glycan biosynthesis | 3 | 142 | 0.368663 | 8.45E-01 | ko00512 |
| Organismal Systems | Digestive system | Bile secretion | 9 | 513 | 0.382629 | 8.70E-01 | ko04976 |
| Metabolism | Lipid metabolism | Synthesis and degradation of ketone bodies | 1 | 32 | 0.388746 | 8.76E-01 | ko00072 |
| Metabolism | Amino acid metabolism | Alanine, aspartate and glutamate metabolism | 4 | 208 | 0.391908 | 8.76E-01 | ko00250 |
| Metabolism | Global and overview maps | Fatty acid metabolism | 5 | 275 | 0.409703 | 9.08E-01 | ko01212 |
| Metabolism | Biosynthesis of other secondary metabolites | Caffeine metabolism | 1 | 35 | 0.416333 | 9.15E-01 | ko00232 |
| Environmental Information Processing | Signal transduction | Hippo signaling pathway -fly | 5 | 285 | 0.439652 | 9.51E-01 | ko04391 |
| Organismal Systems | Digestive system | Mineral absorption | 5 | 285 | 0.439652 | 9.51E-01 | ko04978 |
| Metabolism | Lipid metabolism | Glycerolipid metabolism | 5 | 290 | 0.454501 | 9.75E-01 | ko00561 |
| Organismal Systems | Excretory system | Aldosterone-regulated sodium reabsorption | 3 | 164 | 0.458074 | 9.75E-01 | ko04960 |
| Metabolism | Lipid metabolism | Glycerophospholipid metabolism | 8 | 487 | 0.466073 | 9.80E-01 | ko00564 |
| Metabolism | Metabolism of cofactors and vitamins | Thiamine metabolism | 1 | 41 | 0.467839 | 9.80E-01 | ko00730 |
| Metabolism | Lipid metabolism | Arachidonic acid metabolism | 4 | 232 | 0.473234 | 9.83E-01 | ko00590 |
| Metabolism | Global and overview maps | Biosynthesis of amino acids | 5 | 303 | 0.492563 | 9.99E-01 | ko01230 |
| Metabolism | Lipid metabolism | Fatty acid degradation | 5 | 304 | 0.495452 | 9.99E-01 | ko00071 |
| Metabolism | Glycan biosynthesis and metabolism | Glycosylphosphatidylinositol(GPI)-anchor biosynthesis | 1 | 46 | 0.50728 | 9.99E-01 | ko00563 |
| Organismal Systems | Immune system | T cell receptor signaling pathway | 7 | 440 | 0.508452 | 9.99E-01 | ko04660 |
| Cellular Processes | Cell growth and death | Cell cycle | 5 | 312 | 0.518339 | 9.99E-01 | ko04110 |
| Metabolism | Carbohydrate metabolism | Glycolysis / Gluconeogenesis | 5 | 320 | 0.54078 | 9.99E-01 | ko00010 |
| Human Diseases | Substance dependence | Morphine addiction | 3 | 189 | 0.552537 | 9.99E-01 | ko05032 |
| Organismal Systems | Environmental adaptation | Circadian rhythm | 2 | 121 | 0.55319 | 9.99E-01 | ko04710 |
| Human Diseases | Cancers | Pancreatic cancer | 5 | 326 | 0.557289 | 9.99E-01 | ko05212 |
| Human Diseases | Infectious diseases | Legionellosis | 3 | 191 | 0.559669 | 9.99E-01 | ko05134 |
| Human Diseases | Infectious diseases | Shigellosis | 5 | 328 | 0.562727 | 9.99E-01 | ko05131 |
| Environmental Information Processing | Signal transduction | FoxO signaling pathway | 8 | 538 | 0.578724 | 9.99E-01 | ko04068 |
| Metabolism | Lipid metabolism | Steroid hormone biosynthesis | 2 | 131 | 0.596391 | 9.99E-01 | ko00140 |
| Metabolism | Amino acid metabolism | Glycine, serine and threonine metabolism | 2 | 131 | 0.596391 | 9.99E-01 | ko00260 |
| Human Diseases | Cardiovascular diseases | Fluid shear stress and atherosclerosis | 8 | 547 | 0.597489 | 9.99E-01 | ko05418 |
| Human Diseases | Neurodegenerative diseases | Alzheimer disease | 9 | 617 | 0.601018 | 9.99E-01 | ko05010 |
| Environmental Information Processing | Signal transduction | Ras signaling pathway | 11 | 754 | 0.602616 | 9.99E-01 | ko04014 |
| Organismal Systems | Endocrine system | Regulation of lipolysis in adipocyte | 3 | 208 | 0.617438 | 9.99E-01 | ko04923 |
| Organismal Systems | Immune system | Chemokine signaling pathway | 9 | 627 | 0.62019 | 9.99E-01 | ko04062 |
| Metabolism | Lipid metabolism | Steroid biosynthesis | 1 | 63 | 0.620826 | 9.99E-01 | ko00100 |
| Human Diseases | Cancers | MicroRNAs in cancer | 10 | 712 | 0.649087 | 9.99E-01 | ko05206 |
| Organismal Systems | Digestive system | Vitamin digestion and absorption | 5 | 366 | 0.659072 | 9.99E-01 | ko04977 |
| Human Diseases | Cancers | Breast cancer | 5 | 367 | 0.661415 | 9.99E-01 | ko05224 |
| Metabolism | Metabolism of other amino acids | Glutathione metabolism | 4 | 296 | 0.663881 | 9.99E-01 | ko00480 |
| Metabolism | Metabolism of cofactors and vitamins | One carbon pool by folate | 1 | 71 | 0.664822 | 9.99E-01 | ko00670 |
| Organismal Systems | Environmental adaptation | Plant-pathogen interaction | 1 | 71 | 0.664822 | 9.99E-01 | ko04626 |
| Metabolism | Lipid metabolism | Biosynthesis of unsaturated fatty acids | 2 | 151 | 0.673223 | 9.99E-01 | ko01040 |
| Cellular Processes | Cell growth and death | Apoptosis - fly | 3 | 227 | 0.675682 | 9.99E-01 | ko04214 |
| Organismal Systems | Endocrine system | Renin-angiotensin system | 4 | 304 | 0.68412 | 9.99E-01 | ko04614 |
| Genetic Information Processing | Translation | mRNA surveillance pathway | 4 | 306 | 0.689045 | 9.99E-01 | ko03015 |
| Metabolism | Glycan biosynthesis and metabolism | Other types of O-glycan biosynthesis | 1 | 76 | 0.689696 | 9.99E-01 | ko00514 |
| Metabolism | Amino acid metabolism | Cysteine and methionine metabolism | 2 | 158 | 0.697166 | 9.99E-01 | ko00270 |
| Organismal Systems | Development | Dorso-ventral axis formation | 1 | 78 | 0.699121 | 9.99E-01 | ko04320 |
| Metabolism | Carbohydrate metabolism | Inositol phosphate metabolism | 5 | 385 | 0.70179 | 9.99E-01 | ko00562 |
| Organismal Systems | Circulatory system | Cardiac muscle contraction | 2 | 162 | 0.710185 | 9.99E-01 | ko04260 |
| Organismal Systems | Endocrine system | Thyroid hormone signaling pathway | 8 | 608 | 0.712546 | 9.99E-01 | ko04919 |
| Human Diseases | Cancers | Chronic myeloid leukemia | 4 | 317 | 0.715156 | 9.99E-01 | ko05220 |
| Human Diseases | Cancers | Acute myeloid leukemia | 3 | 243 | 0.719457 | 9.99E-01 | ko05221 |
| Organismal Systems | Aging | Longevity regulating pathway - multiple species | 3 | 249 | 0.734647 | 9.99E-01 | ko04213 |
| Human Diseases | Neurodegenerative diseases | Parkinson disease | 4 | 327 | 0.73747 | 9.99E-01 | ko05012 |
| Human Diseases | Infectious diseases | Bacterial invasion of epithelial cells | 6 | 482 | 0.746976 | 9.99E-01 | ko05100 |
| Metabolism | Glycan biosynthesis and metabolism | Glycosaminoglycan degradation | 1 | 94 | 0.764942 | 9.99E-01 | ko00531 |
| Metabolism | Carbohydrate metabolism | Butanoate metabolism | 1 | 94 | 0.764942 | 9.99E-01 | ko00650 |
| Organismal Systems | Endocrine system | Progesterone-mediated oocyte maturation | 3 | 262 | 0.765328 | 9.99E-01 | ko04914 |
| Metabolism | Glycan biosynthesis and metabolism | Various types of N-glycan biosynthesis | 2 | 181 | 0.765774 | 9.99E-01 | ko00513 |
| Environmental Information Processing | Signal transduction | Rap1 signaling pathway | 11 | 866 | 0.772978 | 9.99E-01 | ko04015 |
| Metabolism | Metabolism of cofactors and vitamins | Porphyrin and chlorophyll metabolism | 1 | 99 | 0.782403 | 9.99E-01 | ko00860 |
| Environmental Information Processing | Signal transduction | AMPK signaling pathway | 7 | 581 | 0.786007 | 9.99E-01 | ko04152 |
| Environmental Information Processing | Signal transduction | MAPK signaling pathway - fly | 4 | 356 | 0.794695 | 9.99E-01 | ko04013 |
| Metabolism | Carbohydrate metabolism | Pentose and glucuronate interconversions | 1 | 103 | 0.795435 | 9.99E-01 | ko00040 |
| Metabolism | Glycan biosynthesis and metabolism | Other glycan degradation | 1 | 103 | 0.795435 | 9.99E-01 | ko00511 |
| Metabolism | Amino acid metabolism | Arginine biosynthesis | 1 | 104 | 0.798569 | 9.99E-01 | ko00220 |
| Metabolism | Carbohydrate metabolism | Glyoxylate and dicarboxylate metabolism | 1 | 106 | 0.804696 | 9.99E-01 | ko00630 |
| Organismal Systems | Nervous system | Long-term depression | 2 | 199 | 0.809669 | 9.99E-01 | ko04730 |
| Organismal Systems | Immune system | Leukocyte transendothelial migration | 7 | 602 | 0.81508 | 9.99E-01 | ko04670 |
| Cellular Processes | Cellular community - eukaryotes | Focal adhesion | 13 | 1050 | 0.816184 | 9.99E-01 | ko04510 |
| Organismal Systems | Development | Axon guidance | 7 | 604 | 0.817683 | 9.99E-01 | ko04360 |
| Environmental Information Processing | Signal transduction | ErbB signaling pathway | 4 | 370 | 0.818515 | 9.99E-01 | ko04012 |
| Genetic Information Processing | Folding, sorting and degradation | SNARE interactions in vesicular transport | 1 | 113 | 0.82471 | 9.99E-01 | ko04130 |
| Environmental Information Processing | Signal transduction | Phosphatidylinositol signaling system | 6 | 534 | 0.827034 | 9.99E-01 | ko04070 |
| Genetic Information Processing | Folding, sorting and degradation | Proteasome | 1 | 116 | 0.832649 | 9.99E-01 | ko03050 |
| Metabolism | Energy metabolism | Carbon fixation in photosynthetic organisms | 1 | 118 | 0.837741 | 9.99E-01 | ko00710 |
| Human Diseases | Infectious diseases | Epithelial cell signaling in Helicobacter pylori infection | 3 | 301 | 0.840396 | 9.99E-01 | ko05120 |
| Metabolism | Xenobiotics biodegradation and metabolism | Drug metabolism - other enzymes | 2 | 222 | 0.855024 | 9.99E-01 | ko00983 |
| Environmental Information Processing | Signaling molecules and interaction | Neuroactive ligand-receptor interaction | 3 | 313 | 0.858895 | 9.99E-01 | ko04080 |
| Organismal Systems | Aging | Longevity regulating pathway - mammal | 4 | 401 | 0.863229 | 9.99E-01 | ko04211 |
| Organismal Systems | Aging | Longevity regulating pathway - worm | 4 | 402 | 0.8645 | 9.99E-01 | ko04212 |
| Environmental Information Processing | Signal transduction | Hippo signaling pathway | 5 | 489 | 0.869651 | 9.99E-01 | ko04390 |
| Organismal Systems | Excretory system | Proximal tubule bicarbonate reclamation | 1 | 134 | 0.873293 | 9.99E-01 | ko04964 |
| Metabolism | Energy metabolism | Oxidative phosphorylation | 3 | 329 | 0.880629 | 9.99E-01 | ko00190 |
| Metabolism | Energy metabolism | Methane metabolism | 1 | 138 | 0.880893 | 9.99E-01 | ko00680 |
| Metabolism | Amino acid metabolism | Histidine metabolism | 1 | 139 | 0.88272 | 9.99E-01 | ko00340 |
| Human Diseases | Cancers | Colorectal cancer | 2 | 241 | 0.884817 | 9.99E-01 | ko05210 |
| Organismal Systems | Nervous system | Neurotrophin signaling pathway | 5 | 504 | 0.885645 | 9.99E-01 | ko04722 |
| Human Diseases | Cancers | Choline metabolism in cancer | 5 | 504 | 0.885645 | 9.99E-01 | ko05231 |
| Human Diseases | Drug resistance | Endocrine resistance | 3 | 334 | 0.886785 | 9.99E-01 | ko01522 |
| Metabolism | Carbohydrate metabolism | Propanoate metabolism | 1 | 142 | 0.888038 | 9.99E-01 | ko00640 |
| Metabolism | Lipid metabolism | Fatty acid elongation | 1 | 145 | 0.893115 | 9.99E-01 | ko00062 |
| Environmental Information Processing | Signal transduction | cAMP signaling pathway | 7 | 694 | 0.90786 | 9.99E-01 | ko04024 |
| Human Diseases | Cancers | Renal cell carcinoma | 3 | 355 | 0.909655 | 9.99E-01 | ko05211 |
| Metabolism | Amino acid metabolism | Tryptophan metabolism | 2 | 262 | 0.911102 | 9.99E-01 | ko00380 |
| Environmental Information Processing | Signal transduction | Hedgehog signaling pathway | 1 | 161 | 0.916558 | 9.99E-01 | ko04340 |
| Organismal Systems | Endocrine system | Ovarian Steroidogenesis | 1 | 161 | 0.916558 | 9.99E-01 | ko04913 |
| Metabolism | Amino acid metabolism | Valine, leucine and isoleucine degradation | 2 | 270 | 0.919551 | 9.99E-01 | ko00280 |
| Human Diseases | Cancers | Glioma | 2 | 279 | 0.928154 | 9.99E-01 | ko05214 |
| Metabolism | Glycan biosynthesis and metabolism | N-Glycan biosynthesis | 1 | 171 | 0.928528 | 9.99E-01 | ko00510 |
| Genetic Information Processing | Translation | RNA transport | 4 | 468 | 0.92873 | 9.99E-01 | ko03013 |
| Cellular Processes | Cellular community - eukaryotes | Adherens junction | 4 | 470 | 0.930157 | 9.99E-01 | ko04520 |
| Human Diseases | Cardiovascular diseases | Hypertrophic cardiomyopathy (HCM) | 2 | 282 | 0.930823 | 9.99E-01 | ko05410 |
| Environmental Information Processing | Signaling molecules and interaction | ECM-receptor interaction | 3 | 380 | 0.931383 | 9.99E-01 | ko04512 |
| Metabolism | Metabolism of other amino acids | beta-Alanine metabolism | 1 | 174 | 0.931773 | 9.99E-01 | ko00410 |
| Human Diseases | Neurodegenerative diseases | Huntington disease | 5 | 573 | 0.939313 | 9.99E-01 | ko05016 |
| Human Diseases | Cancers | Prostate cancer | 3 | 393 | 0.94068 | 9.99E-01 | ko05215 |
| Cellular Processes | Transport and catabolism | Endocytosis | 13 | 1238 | 0.942771 | 9.99E-01 | ko04144 |
| Organismal Systems | Nervous system | Glutamatergic synapse | 2 | 299 | 0.944265 | 9.99E-01 | ko04724 |
| Human Diseases | Infectious diseases | Pathogenic Escherichia coli infection | 2 | 300 | 0.944973 | 9.99E-01 | ko05130 |
| Metabolism | Lipid metabolism | Sphingolipid metabolism | 2 | 302 | 0.946364 | 9.99E-01 | ko00600 |
| Organismal Systems | Endocrine system | Estrogen signaling pathway | 3 | 402 | 0.94642 | 9.99E-01 | ko04915 |
| Environmental Information Processing | Signal transduction | Sphingolipid signaling pathway | 5 | 587 | 0.946941 | 9.99E-01 | ko04071 |
| Human Diseases | Substance dependence | Alcoholism | 2 | 305 | 0.948387 | 9.99E-01 | ko05034 |
| Human Diseases | Infectious diseases | Vibrio cholerae infection | 2 | 307 | 0.949696 | 9.99E-01 | ko05110 |
| Metabolism | Amino acid metabolism | Lysine degradation | 2 | 314 | 0.95403 | 9.99E-01 | ko00310 |
| Environmental Information Processing | Signal transduction | Wnt signaling pathway | 3 | 428 | 0.960234 | 9.99E-01 | ko04310 |
| Human Diseases | Cancers | Melanoma | 1 | 209 | 0.960342 | 9.99E-01 | ko05218 |
| Metabolism | Amino acid metabolism | Arginine and proline metabolism | 1 | 213 | 0.962728 | 9.99E-01 | ko00330 |
| Cellular Processes | Transport and catabolism | Autophagy - animal | 5 | 639 | 0.96827 | 9.99E-01 | ko04140 |
| Organismal Systems | Digestive system | Pancreatic secretion | 3 | 449 | 0.968877 | 9.99E-01 | ko04972 |
| Organismal Systems | Nervous system | GABAergic synapse | 1 | 226 | 0.969538 | 9.99E-01 | ko04727 |
| Organismal Systems | Nervous system | Serotonergic synapse | 2 | 351 | 0.971619 | 9.99E-01 | ko04726 |
| Cellular Processes | Cellular community - eukaryotes | Tight junction | 7 | 836 | 0.973058 | 9.99E-01 | ko04530 |
| Organismal Systems | Endocrine system | Insulin secretion | 1 | 234 | 0.973097 | 9.99E-01 | ko04911 |
| Human Diseases | Cancers | Endometrial cancer | 1 | 237 | 0.974322 | 9.99E-01 | ko05213 |
| Organismal Systems | Digestive system | Protein digestion and absorption | 5 | 668 | 0.976409 | 9.99E-01 | ko04974 |
| Cellular Processes | Transport and catabolism | Autophagy - yeast | 1 | 247 | 0.978017 | 9.99E-01 | ko04138 |
| Organismal Systems | Environmental adaptation | Circadian entrainment | 1 | 252 | 0.97966 | 9.99E-01 | ko04713 |
| Organismal Systems | Endocrine system | Aldosterone synthesis and secretion | 1 | 254 | 0.980283 | 9.99E-01 | ko04925 |
| Human Diseases | Cardiovascular diseases | Arrhythmogenic right ventricular cardiomyopathy (ARVC) | 1 | 257 | 0.981181 | 9.99E-01 | ko05412 |
| Environmental Information Processing | Signal transduction | VEGF signaling pathway | 1 | 259 | 0.981757 | 9.99E-01 | ko04370 |
| Human Diseases | Drug resistance | EGFR tyrosine kinase inhibitor resistance | 2 | 387 | 0.9824 | 9.99E-01 | ko01521 |
| Human Diseases | Cancers | Non-small cell lung cancer | 1 | 272 | 0.985097 | 9.99E-01 | ko05223 |
| Organismal Systems | Digestive system | Fat digestion and absorption | 2 | 400 | 0.985216 | 9.99E-01 | ko04975 |
| Organismal Systems | Sensory system | Inflammatory mediator regulation of TRP channels | 2 | 410 | 0.98708 | 9.99E-01 | ko04750 |
| Environmental Information Processing | Signal transduction | mTOR signaling pathway | 3 | 523 | 0.987218 | 9.99E-01 | ko04150 |
| Organismal Systems | Endocrine system | Glucagon signaling pathway | 2 | 418 | 0.988405 | 9.99E-01 | ko04922 |
| Organismal Systems | Endocrine system | Melanogenesis | 1 | 289 | 0.988561 | 9.99E-01 | ko04916 |
| Organismal Systems | Endocrine system | GnRH signaling pathway | 1 | 305 | 0.991084 | 9.99E-01 | ko04912 |
| Genetic Information Processing | Transcription | Spliceosome | 2 | 441 | 0.991519 | 9.99E-01 | ko03040 |
| Organismal Systems | Endocrine system | Oxytocin signaling pathway | 3 | 563 | 0.992216 | 9.99E-01 | ko04921 |
| Environmental Information Processing | Signal transduction | Apelin signaling pathway | 2 | 454 | 0.9929 | 9.99E-01 | ko04371 |
| Cellular Processes | Cell growth and death | Oocyte meiosis | 1 | 328 | 0.99377 | 9.99E-01 | ko04114 |
| Organismal Systems | Nervous system | Cholinergic synapse | 1 | 375 | 0.997009 | 9.99E-01 | ko04725 |
| Organismal Systems | Nervous system | Dopaminergic synapse | 1 | 375 | 0.997009 | 9.99E-01 | ko04728 |
| Cellular Processes | Cellular community - eukaryotes | Signaling pathways regulating pluripotency of stem cells | 1 | 403 | 0.99807 | 9.99E-01 | ko04550 |
| Human Diseases | Cancers | Proteoglycans in cancer | 5 | 948 | 0.998979 | 9.99E-01 | ko05205 |
| Cellular Processes | Transport and catabolism | Lysosome | 4 | 842 | 0.999035 | 9.99E-01 | ko04142 |
| Cellular Processes | Cell motility | Regulation of actin cytoskeleton | 5 | 967 | 0.999187 | 9.99E-01 | ko04810 |
| Environmental Information Processing | Signal transduction | cGMP - PKG signaling pathway | 2 | 616 | 0.999267 | 9.99E-01 | ko04022 |
| Organismal Systems | Immune system | Platelet activation | 2 | 641 | 0.999488 | 9.99E-01 | ko04611 |

| **Supplementary Table 7. The KEGG pathway enrichment of Profile B** | | | | | | | |
| --- | --- | --- | --- | --- | --- | --- | --- |
| KEGG_A_class | KEGG_B_class | Pathway | profile1 (314) | All (23075) | Pvalue | Qvalue | Pathway ID |
| Organismal Systems | Digestive system | Fat digestion and absorption | 32 | 400 | 9.00E-16 | 2.43E-13 | ko04975 |
| Metabolism | Carbohydrate metabolism | Amino sugar and nucleotide sugar metabolism | 24 | 369 | 3.03E-10 | 4.09E-08 | ko00520 |
| Organismal Systems | Digestive system | Vitamin digestion and absorption | 23 | 366 | 1.40E-09 | 1.26E-07 | ko04977 |
| Organismal Systems | Digestive system | Protein digestion and absorption | 31 | 668 | 3.14E-09 | 2.12E-07 | ko04974 |
| Metabolism | Amino acid metabolism | Tryptophan metabolism | 17 | 262 | 1.30E-07 | 7.01E-06 | ko00380 |
| Organismal Systems | Excretory system | Proximal tubule bicarbonate reclamation | 11 | 134 | 2.31E-06 | 1.04E-04 | ko04964 |
| Metabolism | Amino acid metabolism | Glycine, serine and threonine metabolism | 10 | 131 | 1.27E-05 | 4.91E-04 | ko00260 |
| Cellular Processes | Cellular community - prokaryotes | Quorum sensing | 8 | 84 | 1.91E-05 | 6.45E-04 | ko02024 |
| Organismal Systems | Endocrine system | PPAR signaling pathway | 18 | 423 | 2.25E-05 | 6.74E-04 | ko03320 |
| Metabolism | Lipid metabolism | Fatty acid biosynthesis | 8 | 107 | 0.000109 | 2.95E-03 | ko00061 |
| Organismal Systems | Digestive system | Pancreatic secretion | 17 | 449 | 0.000157 | 3.85E-03 | ko04972 |
| Organismal Systems | Digestive system | Bile secretion | 18 | 513 | 0.000258 | 5.79E-03 | ko04976 |
| Organismal Systems | Endocrine system | Renin-angiotensin system | 13 | 304 | 0.000294 | 6.10E-03 | ko04614 |
| Metabolism | Metabolism of cofactors and vitamins | One carbon pool by folate | 6 | 71 | 0.000413 | 7.49E-03 | ko00670 |
| Metabolism | Global and overview maps | Fatty acid metabolism | 12 | 275 | 0.000416 | 7.49E-03 | ko01212 |
| Metabolism | Xenobiotics biodegradation and metabolism | Aminobenzoate degradation | 4 | 28 | 0.000532 | 8.98E-03 | ko00627 |
| Metabolism | Carbohydrate metabolism | Glyoxylate and dicarboxylate metabolism | 7 | 106 | 0.000624 | 9.91E-03 | ko00630 |
| Organismal Systems | Endocrine system | Adipocytokine signaling pathway | 14 | 390 | 0.000987 | 1.48E-02 | ko04920 |
| Metabolism | Global and overview maps | Carbon metabolism | 17 | 538 | 0.001211 | 1.72E-02 | ko01200 |
| Metabolism | Carbohydrate metabolism | Citrate cycle (TCA cycle) | 8 | 156 | 0.00137 | 1.85E-02 | ko00020 |
| Metabolism | Lipid metabolism | Steroid hormone biosynthesis | 7 | 131 | 0.002143 | 2.76E-02 | ko00140 |
| Metabolism | Carbohydrate metabolism | Starch and sucrose metabolism | 8 | 169 | 0.002267 | 2.78E-02 | ko00500 |
| Metabolism | Metabolism of other amino acids | beta-Alanine metabolism | 8 | 174 | 0.002715 | 3.19E-02 | ko00410 |
| Metabolism | Lipid metabolism | Fatty acid degradation | 11 | 304 | 0.003156 | 3.55E-02 | ko00071 |
| Organismal Systems | Endocrine system | Renin secretion | 10 | 278 | 0.004985 | 5.38E-02 | ko04924 |
| Environmental Information Processing | Signal transduction | AMPK signaling pathway | 16 | 581 | 0.006323 | 6.57E-02 | ko04152 |
| Metabolism | Metabolism of other amino acids | Cyanoamino acid metabolism | 3 | 30 | 0.007721 | 7.72E-02 | ko00460 |
| Organismal Systems | Digestive system | Carbohydrate digestion and absorption | 8 | 213 | 0.008995 | 8.67E-02 | ko04973 |
| Human Diseases | Cancers | Chemical carcinogenesis | 7 | 177 | 0.010886 | 1.01E-01 | ko05204 |
| Metabolism | Metabolism of cofactors and vitamins | Riboflavin metabolism | 3 | 36 | 0.01279 | 1.11E-01 | ko00740 |
| Metabolism | Metabolism of cofactors and vitamins | Folate biosynthesis | 3 | 36 | 0.01279 | 1.11E-01 | ko00790 |
| Metabolism | Xenobiotics biodegradation and metabolism | Drug metabolism - cytochrome P450 | 8 | 230 | 0.013814 | 1.17E-01 | ko00982 |
| Metabolism | Metabolism of cofactors and vitamins | Thiamine metabolism | 3 | 41 | 0.018172 | 1.49E-01 | ko00730 |
| Metabolism | Carbohydrate metabolism | Galactose metabolism | 7 | 203 | 0.021512 | 1.71E-01 | ko00052 |
| Metabolism | Lipid metabolism | Sphingolipid metabolism | 9 | 302 | 0.023106 | 1.78E-01 | ko00600 |
| Metabolism | Amino acid metabolism | Arginine and proline metabolism | 7 | 213 | 0.02705 | 2.03E-01 | ko00330 |
| Cellular Processes | Transport and catabolism | Peroxisome | 11 | 420 | 0.029668 | 2.16E-01 | ko04146 |
| Metabolism | Lipid metabolism | Primary bile acid biosynthesis | 5 | 130 | 0.032717 | 2.32E-01 | ko00120 |
| Environmental Information Processing | Signal transduction | Two-component system | 3 | 54 | 0.037199 | 2.58E-01 | ko02020 |
| Metabolism | Lipid metabolism | Arachidonic acid metabolism | 7 | 232 | 0.04009 | 2.71E-01 | ko00590 |
| Metabolism | Carbohydrate metabolism | Propanoate metabolism | 5 | 142 | 0.04503 | 2.97E-01 | ko00640 |
| Metabolism | Metabolism of cofactors and vitamins | Porphyrin and chlorophyll metabolism | 4 | 99 | 0.046374 | 2.98E-01 | ko00860 |
| Human Diseases | Infectious diseases | African trypanosomiasis | 6 | 193 | 0.049028 | 3.08E-01 | ko05143 |
| Metabolism | Xenobiotics biodegradation and metabolism | Metabolism of xenobiotics by cytochrome P450 | 5 | 153 | 0.05845 | 3.59E-01 | ko00980 |
| Organismal Systems | Endocrine system | Glucagon signaling pathway | 10 | 418 | 0.061058 | 3.59E-01 | ko04922 |
| Metabolism | Lipid metabolism | Linoleic acid metabolism | 5 | 155 | 0.061111 | 3.59E-01 | ko00591 |
| Metabolism | Biosynthesis of other secondary metabolites | Indole alkaloid biosynthesis | 1 | 5 | 0.066218 | 3.72E-01 | ko00901 |
| Metabolism | Biosynthesis of other secondary metabolites | Betalain biosynthesis | 1 | 5 | 0.066218 | 3.72E-01 | ko00965 |
| Metabolism | Amino acid metabolism | Valine, leucine and isoleucine degradation | 7 | 270 | 0.076979 | 4.24E-01 | ko00280 |
| Metabolism | Carbohydrate metabolism | Ascorbate and aldarate metabolism | 4 | 122 | 0.085467 | 4.52E-01 | ko00053 |
| Metabolism | Amino acid metabolism | Tyrosine metabolism | 4 | 122 | 0.085467 | 4.52E-01 | ko00350 |
| Metabolism | Biosynthesis of other secondary metabolites | Isoquinoline alkaloid biosynthesis | 2 | 43 | 0.115963 | 5.88E-01 | ko00950 |
| Metabolism | Lipid metabolism | Cutin, suberine and wax biosynthesis | 1 | 9 | 0.11603 | 5.88E-01 | ko00073 |
| Metabolism | Energy metabolism | Methane metabolism | 4 | 138 | 0.119661 | 5.88E-01 | ko00680 |
| Metabolism | Metabolism of cofactors and vitamins | Retinol metabolism | 5 | 191 | 0.120255 | 5.88E-01 | ko00830 |
| Metabolism | Amino acid metabolism | Histidine metabolism | 4 | 139 | 0.12197 | 5.88E-01 | ko00340 |
| Metabolism | Xenobiotics biodegradation and metabolism | Bisphenol degradation | 1 | 10 | 0.128064 | 6.07E-01 | ko00363 |
| Environmental Information Processing | Signaling molecules and interaction | Neuroactive ligand-receptor interaction | 7 | 313 | 0.136585 | 6.15E-01 | ko04080 |
| Metabolism | Carbohydrate metabolism | Butanoate metabolism | 3 | 94 | 0.136635 | 6.15E-01 | ko00650 |
| Metabolism | Amino acid metabolism | Lysine degradation | 7 | 314 | 0.138185 | 6.15E-01 | ko00310 |
| Metabolism | Lipid metabolism | Ether lipid metabolism | 5 | 201 | 0.140204 | 6.15E-01 | ko00565 |
| Environmental Information Processing | Membrane transport | ABC transporters | 8 | 375 | 0.141218 | 6.15E-01 | ko02010 |
| Metabolism | Amino acid metabolism | Phenylalanine metabolism | 2 | 49 | 0.143426 | 6.15E-01 | ko00360 |
| Metabolism | Carbohydrate metabolism | Glycolysis / Gluconeogenesis | 7 | 320 | 0.147981 | 6.24E-01 | ko00010 |
| Metabolism | Amino acid metabolism | Alanine, aspartate and glutamate metabolism | 5 | 208 | 0.154974 | 6.44E-01 | ko00250 |
| Metabolism | Carbohydrate metabolism | Pentose and glucuronate interconversions | 3 | 103 | 0.165558 | 6.77E-01 | ko00040 |
| Metabolism | Metabolism of cofactors and vitamins | Pantothenate and CoA biosynthesis | 2 | 56 | 0.176933 | 7.03E-01 | ko00770 |
| Human Diseases | Endocrine and metabolic diseases | Maturity onset diabetes of the young | 2 | 56 | 0.176933 | 7.03E-01 | ko04950 |
| Human Diseases | Cardiovascular diseases | Hypertrophic cardiomyopathy (HCM) | 6 | 282 | 0.187716 | 7.25E-01 | ko05410 |
| Metabolism | Carbohydrate metabolism | Fructose and mannose metabolism | 4 | 165 | 0.18803 | 7.25E-01 | ko00051 |
| Metabolism | Metabolism of other amino acids | Selenocompound metabolism | 2 | 59 | 0.191644 | 7.29E-01 | ko00450 |
| Metabolism | Lipid metabolism | Steroid biosynthesis | 2 | 63 | 0.211491 | 7.78E-01 | ko00100 |
| Human Diseases | Endocrine and metabolic diseases | Insulin resistance | 10 | 546 | 0.212243 | 7.78E-01 | ko04931 |
| Metabolism | Metabolism of cofactors and vitamins | Nicotinate and nicotinamide metabolism | 4 | 174 | 0.21315 | 7.78E-01 | ko00760 |
| Metabolism | Metabolism of other amino acids | Glutathione metabolism | 6 | 296 | 0.217182 | 7.82E-01 | ko00480 |
| Organismal Systems | Digestive system | Salivary secretion | 6 | 299 | 0.223694 | 7.95E-01 | ko04970 |
| Metabolism | Lipid metabolism | alpha-Linolenic acid metabolism | 3 | 123 | 0.235032 | 8.24E-01 | ko00592 |
| Metabolism | Glycan biosynthesis and metabolism | Glycosphingolipid biosynthesis - globo and isoglobo series | 4 | 186 | 0.24796 | 8.58E-01 | ko00603 |
| Organismal Systems | Immune system | Hematopoietic cell lineage | 9 | 507 | 0.255275 | 8.66E-01 | ko04640 |
| Human Diseases | Infectious diseases | Chagas disease (American trypanosomiasis) | 7 | 379 | 0.259215 | 8.66E-01 | ko05142 |
| Human Diseases | Drug resistance | Antifolate resistance | 4 | 190 | 0.259832 | 8.66E-01 | ko01523 |
| Human Diseases | Immune diseases | Primary immunodeficiency | 4 | 197 | 0.280861 | 9.25E-01 | ko05340 |
| Organismal Systems | Aging | Longevity regulating pathway - worm | 7 | 402 | 0.307977 | 1.00E+00 | ko04212 |
| Organismal Systems | Endocrine system | Regulation of lipolysis in adipocyte | 4 | 208 | 0.314386 | 1.00E+00 | ko04923 |
| Human Diseases | Neurodegenerative diseases | Alzheimer disease | 10 | 617 | 0.331927 | 1.00E+00 | ko05010 |
| Metabolism | Xenobiotics biodegradation and metabolism | Chloroalkane and chloroalkene degradation | 2 | 88 | 0.337078 | 1.00E+00 | ko00625 |
| Metabolism | Metabolism of other amino acids | D-Glutamine and D-glutamate metabolism | 1 | 30 | 0.33721 | 1.00E+00 | ko00471 |
| Metabolism | Lipid metabolism | Biosynthesis of unsaturated fatty acids | 3 | 151 | 0.33828 | 1.00E+00 | ko01040 |
| Metabolism | Lipid metabolism | Glycerophospholipid metabolism | 8 | 487 | 0.344895 | 1.00E+00 | ko00564 |
| Metabolism | Metabolism of other amino acids | Taurine and hypotaurine metabolism | 1 | 32 | 0.355149 | 1.00E+00 | ko00430 |
| Metabolism | Xenobiotics biodegradation and metabolism | Drug metabolism - other enzymes | 4 | 222 | 0.357493 | 1.00E+00 | ko00983 |
| Metabolism | Amino acid metabolism | Cysteine and methionine metabolism | 3 | 158 | 0.36426 | 1.00E+00 | ko00270 |
| Organismal Systems | Nervous system | GABAergic synapse | 4 | 226 | 0.369827 | 1.00E+00 | ko04727 |
| Metabolism | Metabolism of other amino acids | Phosphonate and phosphinate metabolism | 1 | 34 | 0.372604 | 1.00E+00 | ko00440 |
| Organismal Systems | Endocrine system | Ovarian Steroidogenesis | 3 | 161 | 0.375349 | 1.00E+00 | ko04913 |
| Organismal Systems | Environmental adaptation | Circadian rhythm - fly | 1 | 35 | 0.381154 | 1.00E+00 | ko04711 |
| Metabolism | Global and overview maps | Biosynthesis of amino acids | 5 | 303 | 0.395512 | 1.00E+00 | ko01230 |
| Metabolism | Xenobiotics biodegradation and metabolism | Naphthalene degradation | 1 | 37 | 0.397908 | 1.00E+00 | ko00626 |
| Metabolism | Amino acid metabolism | Arginine biosynthesis | 2 | 104 | 0.414698 | 1.00E+00 | ko00220 |
| Metabolism | Global and overview maps | Degradation of aromatic compounds | 1 | 40 | 0.422194 | 1.00E+00 | ko01220 |
| Metabolism | Biosynthesis of other secondary metabolites | Streptomycin biosynthesis | 1 | 42 | 0.437839 | 1.00E+00 | ko00521 |
| Human Diseases | Substance dependence | Cocaine addiction | 2 | 109 | 0.438044 | 1.00E+00 | ko05030 |
| Environmental Information Processing | Signal transduction | FoxO signaling pathway | 8 | 538 | 0.449862 | 1.00E+00 | ko04068 |
| Metabolism | Carbohydrate metabolism | Pyruvate metabolism | 3 | 187 | 0.469091 | 1.00E+00 | ko00620 |
| Metabolism | Glycan biosynthesis and metabolism | Glycosphingolipid biosynthesis - ganglio series | 2 | 116 | 0.469855 | 1.00E+00 | ko00604 |
| Metabolism | Metabolism of terpenoids and polyketides | Limonene and pinene degradation | 1 | 47 | 0.47513 | 1.00E+00 | ko00903 |
| Metabolism | Metabolism of terpenoids and polyketides | Insect hormone biosynthesis | 1 | 47 | 0.47513 | 1.00E+00 | ko00981 |
| Metabolism | Energy metabolism | Carbon fixation in photosynthetic organisms | 2 | 118 | 0.478746 | 1.00E+00 | ko00710 |
| Metabolism | Metabolism of cofactors and vitamins | Vitamin B6 metabolism | 1 | 48 | 0.482287 | 1.00E+00 | ko00750 |
| Organismal Systems | Environmental adaptation | Circadian rhythm | 2 | 121 | 0.491909 | 1.00E+00 | ko04710 |
| Organismal Systems | Nervous system | Serotonergic synapse | 5 | 351 | 0.521474 | 1.00E+00 | ko04726 |
| Human Diseases | Cancers | Choline metabolism in cancer | 7 | 504 | 0.531295 | 1.00E+00 | ko05231 |
| Human Diseases | Drug resistance | Platinum drug resistance | 4 | 281 | 0.533538 | 1.00E+00 | ko01524 |
| Organismal Systems | Digestive system | Mineral absorption | 4 | 285 | 0.544684 | 1.00E+00 | ko04978 |
| Metabolism | Lipid metabolism | Glycerolipid metabolism | 4 | 290 | 0.558422 | 1.00E+00 | ko00561 |
| Environmental Information Processing | Signal transduction | MAPK signaling pathway - plant | 1 | 61 | 0.566937 | 1.00E+00 | ko04016 |
| Metabolism | Glycan biosynthesis and metabolism | Mucin type O-glycan biosynthesis | 2 | 142 | 0.577948 | 1.00E+00 | ko00512 |
| Organismal Systems | Endocrine system | Thyroid hormone signaling pathway | 8 | 608 | 0.58798 | 1.00E+00 | ko04919 |
| Metabolism | Lipid metabolism | Fatty acid elongation | 2 | 145 | 0.589337 | 1.00E+00 | ko00062 |
| Metabolism | Carbohydrate metabolism | Inositol phosphate metabolism | 5 | 385 | 0.603542 | 1.00E+00 | ko00562 |
| Metabolism | Glycan biosynthesis and metabolism | Glycosaminoglycan biosynthesis - keratan sulfate | 1 | 70 | 0.617311 | 1.00E+00 | ko00533 |
| Organismal Systems | Environmental adaptation | Plant-pathogen interaction | 1 | 71 | 0.622535 | 1.00E+00 | ko04626 |
| Organismal Systems | Aging | Longevity regulating pathway - multiple species | 3 | 249 | 0.661303 | 1.00E+00 | ko04213 |
| Human Diseases | Substance dependence | Amphetamine addiction | 2 | 168 | 0.669064 | 1.00E+00 | ko05031 |
| Environmental Information Processing | Signal transduction | Calcium signaling pathway | 8 | 689 | 0.723841 | 1.00E+00 | ko04020 |
| Metabolism | Glycan biosynthesis and metabolism | Glycosaminoglycan degradation | 1 | 94 | 0.724877 | 1.00E+00 | ko00531 |
| Metabolism | Global and overview maps | 2-Oxocarboxylic acid metabolism | 1 | 95 | 0.728636 | 1.00E+00 | ko01210 |
| Human Diseases | Infectious diseases | Legionellosis | 2 | 191 | 0.735831 | 1.00E+00 | ko05134 |
| Organismal Systems | Endocrine system | Insulin signaling pathway | 7 | 621 | 0.743926 | 1.00E+00 | ko04910 |
| Metabolism | Glycan biosynthesis and metabolism | Other glycan degradation | 1 | 103 | 0.756923 | 1.00E+00 | ko00511 |
| Environmental Information Processing | Signal transduction | TGF-beta signaling pathway | 2 | 218 | 0.799262 | 1.00E+00 | ko04350 |
| Organismal Systems | Immune system | Cytosolic DNA-sensing pathway | 1 | 121 | 0.81028 | 1.00E+00 | ko04623 |
| Environmental Information Processing | Signal transduction | Sphingolipid signaling pathway | 6 | 587 | 0.813046 | 1.00E+00 | ko04071 |
| Human Diseases | Infectious diseases | Influenza A | 8 | 759 | 0.814266 | 1.00E+00 | ko05164 |
| Organismal Systems | Endocrine system | Insulin secretion | 2 | 234 | 0.830155 | 1.00E+00 | ko04911 |
| Metabolism | Nucleotide metabolism | Purine metabolism | 5 | 516 | 0.833906 | 1.00E+00 | ko00230 |
| Metabolism | Carbohydrate metabolism | Pentose phosphate pathway | 1 | 131 | 0.834697 | 1.00E+00 | ko00030 |
| Human Diseases | Drug resistance | Endocrine resistance | 3 | 334 | 0.83535 | 1.00E+00 | ko01522 |
| Human Diseases | Infectious diseases | Staphylococcus aureus infection | 2 | 237 | 0.835448 | 1.00E+00 | ko05150 |
| Environmental Information Processing | Signal transduction | PI3K-Akt signaling pathway | 14 | 1283 | 0.836965 | 1.00E+00 | ko04151 |
| Organismal Systems | Immune system | Intestinal immune network for IgA production | 2 | 239 | 0.838894 | 1.00E+00 | ko04672 |
| Human Diseases | Cancers | Acute myeloid leukemia | 2 | 243 | 0.845591 | 1.00E+00 | ko05221 |
| Environmental Information Processing | Signal transduction | Phosphatidylinositol signaling system | 5 | 534 | 0.854855 | 1.00E+00 | ko04070 |
| Cellular Processes | Cell growth and death | Meiosis - yeast | 1 | 143 | 0.859896 | 1.00E+00 | ko04113 |
| Environmental Information Processing | Signal transduction | VEGF signaling pathway | 2 | 259 | 0.869926 | 1.00E+00 | ko04370 |
| Human Diseases | Immune diseases | Autoimmune thyroid disease | 3 | 360 | 0.870352 | 1.00E+00 | ko05320 |
| Human Diseases | Immune diseases | Allograft rejection | 3 | 361 | 0.871557 | 1.00E+00 | ko05330 |
| Organismal Systems | Endocrine system | Progesterone-mediated oocyte maturation | 2 | 262 | 0.874078 | 1.00E+00 | ko04914 |
| Organismal Systems | Immune system | Fc gamma R-mediated phagocytosis | 5 | 562 | 0.883051 | 1.00E+00 | ko04666 |
| Organismal Systems | Immune system | Fc epsilon RI signaling pathway | 3 | 371 | 0.883075 | 1.00E+00 | ko04664 |
| Human Diseases | Immune diseases | Asthma | 2 | 271 | 0.885813 | 1.00E+00 | ko05310 |
| Organismal Systems | Nervous system | Dopaminergic synapse | 3 | 375 | 0.887419 | 1.00E+00 | ko04728 |
| Metabolism | Nucleotide metabolism | Pyrimidine metabolism | 2 | 273 | 0.88828 | 1.00E+00 | ko00240 |
| Organismal Systems | Immune system | Antigen processing and presentation | 4 | 475 | 0.889784 | 1.00E+00 | ko04612 |
| Organismal Systems | Excretory system | Aldosterone-regulated sodium reabsorption | 1 | 164 | 0.89513 | 1.00E+00 | ko04960 |
| Genetic Information Processing | Folding, sorting and degradation | Protein processing in endoplasmic reticulum | 7 | 765 | 0.899164 | 1.00E+00 | ko04141 |
| Genetic Information Processing | Translation | Ribosome biogenesis in eukaryotes | 1 | 167 | 0.899383 | 1.00E+00 | ko03008 |
| Metabolism | Glycan biosynthesis and metabolism | N-Glycan biosynthesis | 1 | 171 | 0.904788 | 1.00E+00 | ko00510 |
| Human Diseases | Cancers | Prostate cancer | 3 | 393 | 0.905236 | 1.00E+00 | ko05215 |
| Human Diseases | Cardiovascular diseases | Viral myocarditis | 4 | 501 | 0.9121 | 1.00E+00 | ko05416 |
| Organismal Systems | Aging | Longevity regulating pathway - mammal | 3 | 401 | 0.912306 | 1.00E+00 | ko04211 |
| Organismal Systems | Endocrine system | Estrogen signaling pathway | 3 | 402 | 0.913156 | 1.00E+00 | ko04915 |
| Organismal Systems | Nervous system | Glutamatergic synapse | 2 | 299 | 0.916157 | 1.00E+00 | ko04724 |
| Human Diseases | Infectious diseases | Measles | 6 | 699 | 0.916788 | 1.00E+00 | ko05162 |
| Metabolism | Glycan biosynthesis and metabolism | Various types of N-glycan biosynthesis | 1 | 181 | 0.917066 | 1.00E+00 | ko00513 |
| Human Diseases | Endocrine and metabolic diseases | Type II diabetes mellitus | 1 | 181 | 0.917066 | 1.00E+00 | ko04930 |
| Organismal Systems | Sensory system | Inflammatory mediator regulation of TRP channels | 3 | 410 | 0.919687 | 1.00E+00 | ko04750 |
| Human Diseases | Substance dependence | Alcoholism | 2 | 305 | 0.921593 | 1.00E+00 | ko05034 |
| Human Diseases | Immune diseases | Systemic lupus erythematosus | 2 | 306 | 0.922466 | 1.00E+00 | ko05322 |
| Organismal Systems | Circulatory system | Adrenergic signaling in cardiomyocytes | 3 | 416 | 0.924288 | 1.00E+00 | ko04261 |
| Human Diseases | Substance dependence | Morphine addiction | 1 | 189 | 0.925742 | 1.00E+00 | ko05032 |
| Cellular Processes | Cell growth and death | Cell cycle | 2 | 312 | 0.927515 | 1.00E+00 | ko04110 |
| Organismal Systems | Excretory system | Endocrine and other factor-regulated calcium reabsorption | 1 | 197 | 0.933513 | 1.00E+00 | ko04961 |
| Environmental Information Processing | Signal transduction | Notch signaling pathway | 1 | 199 | 0.935326 | 1.00E+00 | ko04330 |
| Organismal Systems | Nervous system | Synaptic vesicle cycle | 1 | 199 | 0.935326 | 1.00E+00 | ko04721 |
| Human Diseases | Cancers | Central carbon metabolism in cancer | 2 | 325 | 0.937413 | 1.00E+00 | ko05230 |
| Genetic Information Processing | Transcription | Spliceosome | 3 | 441 | 0.940979 | 1.00E+00 | ko03040 |
| Organismal Systems | Immune system | B cell receptor signaling pathway | 3 | 441 | 0.940979 | 1.00E+00 | ko04662 |
| Cellular Processes | Transport and catabolism | Lysosome | 7 | 842 | 0.942853 | 1.00E+00 | ko04142 |
| Human Diseases | Cancers | Melanoma | 1 | 209 | 0.943676 | 1.00E+00 | ko05218 |
| Environmental Information Processing | Signal transduction | HIF-1 signaling pathway | 3 | 449 | 0.945557 | 1.00E+00 | ko04066 |
| Environmental Information Processing | Signal transduction | Ras signaling pathway | 6 | 754 | 0.94615 | 1.00E+00 | ko04014 |
| Environmental Information Processing | Signal transduction | Apelin signaling pathway | 3 | 454 | 0.948249 | 1.00E+00 | ko04371 |
| Human Diseases | Infectious diseases | Amoebiasis | 4 | 568 | 0.952213 | 1.00E+00 | ko05146 |
| Human Diseases | Cancers | Renal cell carcinoma | 2 | 355 | 0.955593 | 1.00E+00 | ko05211 |
| Cellular Processes | Cell growth and death | Apoptosis - fly | 1 | 227 | 0.95609 | 1.00E+00 | ko04214 |
| Human Diseases | Immune diseases | Rheumatoid arthritis | 2 | 358 | 0.957104 | 1.00E+00 | ko05323 |
| Human Diseases | Infectious diseases | Leishmaniasis | 2 | 359 | 0.957597 | 1.00E+00 | ko05140 |
| Organismal Systems | Endocrine system | Thyroid hormone synthesis | 1 | 233 | 0.959589 | 1.00E+00 | ko04918 |
| Environmental Information Processing | Signal transduction | Phospholipase D signaling pathway | 5 | 690 | 0.959975 | 1.00E+00 | ko04072 |
| Human Diseases | Infectious diseases | Hepatitis C | 4 | 590 | 0.961171 | 1.00E+00 | ko05160 |
| Human Diseases | Cancers | Breast cancer | 2 | 367 | 0.961349 | 1.00E+00 | ko05224 |
| Environmental Information Processing | Signal transduction | cAMP signaling pathway | 5 | 694 | 0.96137 | 1.00E+00 | ko04024 |
| Genetic Information Processing | Folding, sorting and degradation | RNA degradation | 1 | 237 | 0.961766 | 1.00E+00 | ko03018 |
| Human Diseases | Cancers | Endometrial cancer | 1 | 237 | 0.961766 | 1.00E+00 | ko05213 |
| Environmental Information Processing | Signal transduction | ErbB signaling pathway | 2 | 370 | 0.962673 | 1.00E+00 | ko04012 |
| Human Diseases | Cardiovascular diseases | Dilated cardiomyopathy (DCM) | 2 | 370 | 0.962673 | 1.00E+00 | ko05414 |
| Human Diseases | Cancers | Colorectal cancer | 1 | 241 | 0.963826 | 1.00E+00 | ko05210 |
| Human Diseases | Immune diseases | Graft-versus-host disease | 1 | 246 | 0.966246 | 1.00E+00 | ko05332 |
| Cellular Processes | Transport and catabolism | Autophagy - yeast | 1 | 247 | 0.96671 | 1.00E+00 | ko04138 |
| Organismal Systems | Immune system | RIG-I-like receptor signaling pathway | 1 | 247 | 0.96671 | 1.00E+00 | ko04622 |
| Environmental Information Processing | Signal transduction | Jak-STAT signaling pathway | 3 | 497 | 0.966785 | 1.00E+00 | ko04630 |
| Organismal Systems | Environmental adaptation | Circadian entrainment | 1 | 252 | 0.968938 | 1.00E+00 | ko04713 |
| Human Diseases | Drug resistance | EGFR tyrosine kinase inhibitor resistance | 2 | 387 | 0.96939 | 1.00E+00 | ko01521 |
| Organismal Systems | Endocrine system | Aldosterone synthesis and secretion | 1 | 254 | 0.969787 | 1.00E+00 | ko04925 |
| Organismal Systems | Immune system | NOD-like receptor signaling pathway | 4 | 619 | 0.970617 | 1.00E+00 | ko04621 |
| Human Diseases | Endocrine and metabolic diseases | Type I diabetes mellitus | 1 | 262 | 0.972957 | 1.00E+00 | ko04940 |
| Organismal Systems | Immune system | Natural killer cell mediated cytotoxicity | 4 | 628 | 0.973082 | 1.00E+00 | ko04650 |
| Organismal Systems | Nervous system | Long-term potentiation | 1 | 263 | 0.973329 | 1.00E+00 | ko04720 |
| Human Diseases | Infectious diseases | Epstein-Barr virus infection | 8 | 1040 | 0.974237 | 1.00E+00 | ko05169 |
| Human Diseases | Infectious diseases | Tuberculosis | 7 | 946 | 0.975103 | 1.00E+00 | ko05152 |
| Cellular Processes | Transport and catabolism | Autophagy - animal | 4 | 639 | 0.975832 | 1.00E+00 | ko04140 |
| Human Diseases | Cancers | Non-small cell lung cancer | 1 | 272 | 0.976458 | 1.00E+00 | ko05223 |
| Human Diseases | Endocrine and metabolic diseases | Non-alcoholic fatty liver disease (NAFLD) | 3 | 533 | 0.977299 | 1.00E+00 | ko04932 |
| Organismal Systems | Immune system | IL-17 signaling pathway | 1 | 276 | 0.977728 | 1.00E+00 | ko04657 |
| Human Diseases | Cancers | Glioma | 1 | 279 | 0.978635 | 1.00E+00 | ko05214 |
| Human Diseases | Endocrine and metabolic diseases | AGE-RAGE signaling pathway in diabetic complications | 3 | 539 | 0.97871 | 1.00E+00 | ko04933 |
| Human Diseases | Cardiovascular diseases | Fluid shear stress and atherosclerosis | 3 | 547 | 0.980463 | 1.00E+00 | ko05418 |
| Organismal Systems | Immune system | Toll-like receptor signaling pathway | 1 | 286 | 0.980612 | 1.00E+00 | ko04620 |
| Organismal Systems | Nervous system | Retrograde endocannabinoid signaling | 1 | 289 | 0.981403 | 1.00E+00 | ko04723 |
| Organismal Systems | Endocrine system | Melanogenesis | 1 | 289 | 0.981403 | 1.00E+00 | ko04916 |
| Human Diseases | Infectious diseases | Epithelial cell signaling in Helicobacter pylori infection | 1 | 301 | 0.984256 | 1.00E+00 | ko05120 |
| Organismal Systems | Endocrine system | Prolactin signaling pathway | 1 | 303 | 0.984687 | 1.00E+00 | ko04917 |
| Human Diseases | Neurodegenerative diseases | Huntington disease | 3 | 573 | 0.985258 | 1.00E+00 | ko05016 |
| Genetic Information Processing | Translation | mRNA surveillance pathway | 1 | 306 | 0.985312 | 1.00E+00 | ko03015 |
| Cellular Processes | Transport and catabolism | Phagosome | 7 | 1009 | 0.985414 | 1.00E+00 | ko04145 |
| Environmental Information Processing | Signal transduction | NF-kappa B signaling pathway | 2 | 450 | 0.985511 | 1.00E+00 | ko04064 |
| Human Diseases | Infectious diseases | Vibrio cholerae infection | 1 | 307 | 0.985515 | 1.00E+00 | ko05110 |
| Human Diseases | Cancers | Chronic myeloid leukemia | 1 | 317 | 0.987393 | 1.00E+00 | ko05220 |
| Organismal Systems | Circulatory system | Vascular smooth muscle contraction | 2 | 466 | 0.988052 | 1.00E+00 | ko04270 |
| Human Diseases | Cancers | MicroRNAs in cancer | 4 | 712 | 0.988393 | 1.00E+00 | ko05206 |
| Human Diseases | Cancers | Pancreatic cancer | 1 | 326 | 0.988875 | 1.00E+00 | ko05212 |
| Cellular Processes | Cell growth and death | Oocyte meiosis | 1 | 328 | 0.98918 | 1.00E+00 | ko04114 |
| Metabolism | Energy metabolism | Oxidative phosphorylation | 1 | 329 | 0.98933 | 1.00E+00 | ko00190 |
| Human Diseases | Infectious diseases | Bacterial invasion of epithelial cells | 2 | 482 | 0.990158 | 1.00E+00 | ko05100 |
| Environmental Information Processing | Signal transduction | cGMP - PKG signaling pathway | 3 | 616 | 0.990818 | 1.00E+00 | ko04022 |
| Human Diseases | Infectious diseases | Hepatitis B | 3 | 616 | 0.990818 | 1.00E+00 | ko05161 |
| Environmental Information Processing | Signal transduction | TNF signaling pathway | 1 | 361 | 0.993163 | 1.00E+00 | ko04668 |
| Human Diseases | Cancers | Transcriptional misregulation in cancers | 3 | 651 | 0.993795 | 1.00E+00 | ko05202 |
| Human Diseases | Infectious diseases | Herpes simplex infection | 5 | 889 | 0.993906 | 1.00E+00 | ko05168 |
| Environmental Information Processing | Signal transduction | mTOR signaling pathway | 2 | 523 | 0.994037 | 1.00E+00 | ko04150 |
| Organismal Systems | Nervous system | Cholinergic synapse | 1 | 375 | 0.994374 | 1.00E+00 | ko04725 |
| Environmental Information Processing | Signaling molecules and interaction | ECM-receptor interaction | 1 | 380 | 0.994753 | 1.00E+00 | ko04512 |
| Environmental Information Processing | Signaling molecules and interaction | Cell adhesion molecules (CAMs) | 2 | 542 | 0.995283 | 1.00E+00 | ko04514 |
| Cellular Processes | Cellular community - eukaryotes | Signaling pathways regulating pluripotency of stem cells | 1 | 403 | 0.996192 | 1.00E+00 | ko04550 |
| Environmental Information Processing | Signal transduction | Wnt signaling pathway | 1 | 428 | 0.997313 | 1.00E+00 | ko04310 |
| Human Diseases | Cancers | Small cell lung cancer | 1 | 432 | 0.997459 | 1.00E+00 | ko05222 |
| Organismal Systems | Immune system | T cell receptor signaling pathway | 1 | 440 | 0.997728 | 1.00E+00 | ko04660 |
| Organismal Systems | Immune system | Leukocyte transendothelial migration | 2 | 602 | 0.997766 | 1.00E+00 | ko04670 |
| Organismal Systems | Development | Axon guidance | 2 | 604 | 0.997821 | 1.00E+00 | ko04360 |
| Cellular Processes | Cellular community - eukaryotes | Adherens junction | 1 | 470 | 0.998506 | 1.00E+00 | ko04520 |
| Human Diseases | Cancers | Viral carcinogenesis | 4 | 908 | 0.998575 | 1.00E+00 | ko05203 |
| Human Diseases | Infectious diseases | Toxoplasmosis | 1 | 488 | 0.998839 | 1.00E+00 | ko05145 |
| Environmental Information Processing | Signal transduction | Hippo signaling pathway | 1 | 489 | 0.998855 | 1.00E+00 | ko04390 |
| Organismal Systems | Development | Osteoclast differentiation | 1 | 502 | 0.999046 | 1.00E+00 | ko04380 |
| Organismal Systems | Nervous system | Neurotrophin signaling pathway | 1 | 504 | 0.999072 | 1.00E+00 | ko04722 |
| Organismal Systems | Immune system | Th17 cell differentiation | 1 | 509 | 0.999135 | 1.00E+00 | ko04659 |
| Cellular Processes | Cell growth and death | Apoptosis | 2 | 704 | 0.999387 | 1.00E+00 | ko04210 |
| Cellular Processes | Transport and catabolism | Endocytosis | 6 | 1238 | 0.999442 | 1.00E+00 | ko04144 |
| Environmental Information Processing | Signal transduction | Rap1 signaling pathway | 3 | 866 | 0.999496 | 1.00E+00 | ko04015 |
| Organismal Systems | Endocrine system | Oxytocin signaling pathway | 1 | 563 | 0.999594 | 1.00E+00 | ko04921 |
| Human Diseases | Cancers | Proteoglycans in cancer | 3 | 948 | 0.999814 | 1.00E+00 | ko05205 |
| Organismal Systems | Immune system | Chemokine signaling pathway | 1 | 627 | 0.999835 | 1.00E+00 | ko04062 |
| Organismal Systems | Immune system | Platelet activation | 1 | 641 | 0.999865 | 1.00E+00 | ko04611 |
| Human Diseases | Infectious diseases | HTLV-I infection | 3 | 981 | 0.999876 | 1.00E+00 | ko05166 |
| Cellular Processes | Cellular community - eukaryotes | Tight junction | 2 | 836 | 0.999889 | 1.00E+00 | ko04530 |
| Cellular Processes | Cell motility | Regulation of actin cytoskeleton | 2 | 967 | 0.99998 | 1.00E+00 | ko04810 |
| Environmental Information Processing | Signal transduction | MAPK signaling pathway | 1 | 785 | 0.999982 | 1.00E+00 | ko04010 |
| Cellular Processes | Cellular community - eukaryotes | Focal adhesion | 2 | 1050 | 0.999994 | 1.00E+00 | ko04510 |
| Human Diseases | Cancers | Pathways in cancer | 3 | 1512 | 1 | 1.00E+00 | ko05200 |

**Supplementary Table 8. The co-contained differential metabolites in diets and DI in SBM20_N group contents in positive mode（n=12）**

| Metabolite | FC1 | VIP1 | FC2 | VIP2 | Log2FC1 | Log2FC2 | Change range |
| --- | --- | --- | --- | --- | --- | --- | --- |
| (4S)-4-[(2E,4Z)-2,4-Decadienoyloxy]  -4-(trimethylammonio)butanoate | 0.296647791 | 1.886319868 | 3.935150382 | 3.341759 | -1.753177055 | 1.976418769 | >2, contrary |
| 1-palmitoyl-2-stearoyl-sn-glycero-3-phosphoserine | 2.496076412 | 1.530846271 | 0.261719323 | 3.169006 | 1.3196621 | -1.933907651 | >2, contrary |
| 2,4-dihydroxyheptadec-16-enyl acetate | 2.507765949 | 1.246147333 | 0.339385352 | 2.63689 | 1.326402707 | -1.559003796 | >2, contrary |
| psychotrine | 0.473727148 | 1.126703706 | 3.499273684 | 3.076478 | -1.077871744 | 1.807055505 | >2, contrary |
| Tetroxoprim | 0.42804365 | 1.453279332 | 2.128583898 | 1.863073 | -1.224170171 | 1.089893955 | >2, contrary |
| Ethoxyquin | 0.234623162 | 2.317764869 | 0.311606648 | 4.051256 | -2.091582652 | -1.682202082 | <2, removed |
| Acetylmethadol | 0.477342233 | 1.161647354 | 0.371266861 | 2.421194 | -1.06690411 | -1.429471548 | <2, removed |
| Dihydrokavain | 0.463250258 | 1.222746971 | 0.487285559 | 1.720994 | -1.110136315 | -1.037160627 | <2, removed |
| Cinaciguat | 0.290780882 | 1.970268535 | 0.315635899 | 2.851593 | -1.781995676 | -1.663666795 | <2, removed |

Note: FC, fold change; VIP, Variable Importance in the Projection. FC1, VIP1 and Log2FC1 were measured from DI content samples (SBM20_N/FM group); FC2, VIP2 and Log2FC2 were measured from diet samples (SBM20/FM group). The metabolites with contrary trends were considered to be significantly affected by fish; the co-contained metabolites with same trend and differential times of Log2FC < 2 were considered to be no-significantly changed and removed in the whole differential metabolites of DI contents.

**Supplementary Table 9. The co-contained differential metabolites in diets and DI in SBM40_N group contents in positive mode（n=12）**

| Metabolite | FC1 | VIP1 | FC2 | VIP2 | Log2FC1 | log2FC2 | Change range |
| --- | --- | --- | --- | --- | --- | --- | --- |
| bencyclane | 0.1198536 | 2.166734453 | 0.49370846 | 1.495603 | -3.06066 | -1.01827 | >2,retained |
| Malonylglycitin | 16.702464 | 3.058492432 | 2.0380951 | 1.433911 | 4.061989 | 1.027221 | >2,retained |
| (+/-)-Methoprene | 0.3929308 | 1.033613743 | 3.46799881 | 2.512726 | -1.34765 | 1.794103 | >2,congrary |
| methyl 3-hydroxypalmitate | 0.3370042 | 1.121855757 | 2.77214886 | 2.076308 | -1.56916 | 1.471005 | >2,congrary |
| metixene | 5.4192847 | 1.798547309 | 0.48073459 | 1.444988 | 2.438102 | -1.05669 | >2,congrary |
| Daidzin | 22.359416 | 3.174317493 | 2.14316516 | 1.535149 | 4.482811 | 1.099743 | >2,retained |
| 1-Phenyl-1,3-octadecanedione | 4.1000429 | 1.496971527 | 0.36611751 | 1.99693 | 2.035639 | -1.44962 | >2,congrary |
| Ulipristal | 0.1992586 | 1.615151136 | 2.93755999 | 2.250349 | -2.32729 | 1.554618 | >2,congrary |
| pnb | 0.3123625 | 1.238470037 | 4.48228373 | 2.706957 | -1.67871 | 2.164234 | >2,congrary |
| Genistin | 32.419098 | 3.592423962 | 2.18708279 | 1.786578 | 5.018772 | 1.129008 | >2,retained |
| Fingolimod | 0.0879678 | 2.57335712 | 0.39457886 | 1.828527 | -3.50688 | -1.34161 | >2,retained |
| N-Acetylcytidine | 0.2982501 | 1.194427484 | 2.60189056 | 1.925562 | -1.74541 | 1.37956 | >2,congrary |
| Succinic anhydride | 0.2971948 | 1.53070976 | 2.27729519 | 2.195135 | -1.75052 | 1.187321 | >2,congrary |
| Mesalazine | 0.1101504 | 1.971653467 | 2.28045085 | 1.670129 | -3.18245 | 1.189319 | >2,congrary |
| n-Ribosylhistidine | 0.2662619 | 1.437341515 | 0.14818708 | 3.871106 | -1.90908 | -2.75451 | <2, removed |
| (Ac)2-L-Lys-D-Ala | 0.3846634 | 1.026170197 | 0.42395338 | 1.73885 | -1.37833 | -1.23802 | <2, removed |
| Bevenopran | 0.2474363 | 1.541803973 | 0.48337089 | 1.477512 | -2.01487 | -1.0488 | <2, removed |
| trans-2-Tetradecenoylcarnitine | 0.2276856 | 1.573701038 | 0.38144297 | 1.933312 | -2.13488 | -1.39046 | <2, removed |
| 1-[(Carboxyacetyl)amino]cyclopropanecarboxylic acid | 4.9413635 | 1.714296165 | 2.42572515 | 1.775605 | 2.304909 | 1.278416 | <2, removed |
| 1-palmitoyl-2-stearoyl-sn-glycero-3-phosphoserine | 4.3371736 | 1.787300141 | 2.76389641 | 2.087589 | 2.116755 | 1.466704 | <2, removed |
| 5-Methoxy-3-indoleaceate | 0.2386146 | 1.539652243 | 0.35711086 | 2.073622 | -2.06725 | -1.48556 | <2, removed |
| Araloside A | 3.044832 | 1.325854014 | 2.14549057 | 1.539071 | 1.606363 | 1.101308 | <2, removed |
| Dinoseb | 0.3391567 | 1.376663858 | 0.4977522 | 1.432918 | -1.55998 | -1.0065 | <2, removed |
| Ethoxyquin | 0.1033717 | 1.458732136 | 0.32986194 | 2.781499 | -3.27409 | -1.60007 | <2, removed |
| Ethylmorphine | 0.2062063 | 1.662648189 | 0.37757714 | 2.047894 | -2.27784 | -1.40516 | <2, removed |
| saccharopine | 0.2525802 | 1.442675351 | 0.4796944 | 1.457436 | -1.98519 | -1.05981 | <2, removed |

Note: FC, fold change; VIP, Variable Importance in the Projection. FC1, VIP1 and Log2FC1 were measured from DI content samples (SBM40_N/FM group); FC2, VIP2 and Log2FC2 were measured from diet samples (SBM40/FM group). The metabolites with contrary trends were considered to be significantly affected by fish; the co-contained metabolites with same trend and differential times of Log2FC < 2 were considered to be no-significantly changed and removed in the whole differential metabolites of DI contents.

**Supplementary Table 10. The co-contained differential metabolites in diets and DI contents in SBM20_N group in negative mode （n=12）**

| Metabolite | FC1 | VIP1 | FC2 | VIP2 | Log2FC1 | Log2FC2 | Change range |
| --- | --- | --- | --- | --- | --- | --- | --- |
| PGF2a ethanolamide | 0.460558024 | 1.187939422 | 2.527383305 | 2.276005 | -1.118545167 | 1.337644481 | >2, contrary |
| Enterostatin | 0.131215783 | 2.974249847 | 0.426591814 | 2.114052 | -2.929986833 | -1.229071813 | <2, removed |
| 2-Sulfosuccinic acid | 0.386506382 | 1.242214842 | 0.280196339 | 3.108779 | -1.371435859 | -1.835489989 | <2, removed |
| 2-Acetamido-2-deoxy-D-glucono-1,5-lactone | 4.398847324 | 1.909976418 | 2.649145257 | 2.360593 | 2.137125529 | 1.405526951 | <2, removed |

Note: same as Supplementary Table 8.

**Supplementary Table 11. The co-contained differential metabolites in diets and DI in SBM40_N group contents in negative mode（n=12）**

| Metabolite | FC1 | VIP1 | FC2 | VIP2 | Log2FC1 | Log2FC2 | Change range |
| --- | --- | --- | --- | --- | --- | --- | --- |
| 3-O-beta-D-Galactopyranosyl-D-arabinose | 0.395276273 | 1.13253784 | 0.045258054 | 4.364641 | -1.33907 | -4.46568 | >2,ratained |
| FMNH2 | 14.31066566 | 3.073585751 | 2.956322114 | 1.902759 | 3.839019 | 1.563803 | >2,ratained |
| Ethyl Linoleate | 7.685815837 | 1.19100449 | 0.461904532 | 1.406313 | 2.942198 | -1.11433 | >2,contrary |
| Glycitin | 8.97217627 | 2.231287951 | 2.229747907 | 1.563662 | 3.165458 | 1.156881 | >2,ratained |
| Ginsenoside Ro | 12.65914221 | 2.898653817 | 2.41895986 | 1.60457 | 3.662108 | 1.274387 | >2,ratained |
| LTF4 | 5.368538167 | 1.823357388 | 0.480544411 | 1.270291 | 2.424529 | -1.05726 | >2,contrary |
| disobutamide | 0.232987344 | 1.400971065 | 2.444461756 | 1.572212 | -2.10168 | 1.289517 | >2,contrary |
| Lysophosphatidylinositol | 0.173474469 | 1.475887125 | 2.211611631 | 1.393307 | -2.5272 | 1.145098 | >2,contrary |
| N-Phenylacetylglutamic acid | 3.497820928 | 1.638671698 | 2.224346462 | 1.41123 | 1.806456 | 1.153382 | <2, removed |
| N-acetylleukotriene E4 | 0.219633377 | 1.325684502 | 0.490240361 | 1.313981 | -2.18683 | -1.02844 | <2, removed |
| Gluconic acid | 5.795326624 | 2.371642254 | 2.578243183 | 1.679654 | 2.53489 | 1.366388 | <2, removed |
| Escin IB | 5.271123172 | 1.898326296 | 2.531324151 | 1.675957 | 2.39811 | 1.339892 | <2, removed |
| Malonic acid | 3.712668503 | 1.521856193 | 2.932100581 | 2.020436 | 1.892457 | 1.551935 | <2, removed |
| Alosetron | 0.221943894 | 1.373179413 | 0.457979748 | 1.36532 | -2.17173 | -1.12664 | <2, removed |
| soyasapogenol B 3-O-beta-glucuronide | 4.64583496 | 1.945553988 | 3.172291001 | 2.044757 | 2.215938 | 1.665525 | <2, removed |

Note: same as Supplementary Table 9.

| **Supplementary Table 12. The significnat differential metabolites in DI contents of SBM20 vs. FM in positive mode (n = 12)** | | | | | | | | |
| --- | --- | --- | --- | --- | --- | --- | --- | --- |
|
| ID | Name_des | Formula | Molecular Weight | log2FC | Pvalue | ROC | VIP | Up.Down |
| Com_3378_pos | 5-methylbenzimidazole | C8 H8 N2 | 132.0683 | 5.129712976 | 7.53E-08 | 1 | 5.315462186 | up |
| Com_4140_pos | Propoxur | C11 H15 N O3 | 209.10486 | 5.074534854 | 2.54E-05 | 0.909722222 | 4.818601519 | up |
| Com_5549_pos | 2,2'-([(2R,5R)-2-Benzyl-3,6-dihydroxy-5-(hydroxymethyl)-2,5-dihydropyrazine-2,5-diyl]bis  {sulfanediyl[(1Z,2R)-2-amino-1-hydroxy-3-propyl-1-ylidene](Z)azanylylidene})diacetic acid (non-preferred name) | C22 H30 N6 O9 S2 | 586.15095 | 4.761567006 | 7.03E-08 | 1 | 4.965089168 | up |
| Com_4153_pos | Lurasidone | C28 H36 N4 O2 S | 492.25583 | 4.492042547 | 1.02E-06 | 0.993055556 | 4.602868599 | up |
| Com_4806_pos | (5,10,10-Trihydroxy-2,6-diiminooctahydro-1H,8H-pyrrolo[1,2-c]purin-4-yl)methyl hydroxycarbamate | C10 H17 N7 O6 | 331.12366 | 4.415901593 | 2.19E-09 | 1 | 4.929432852 | up |
| Com_3269_pos | Glycitin | C22 H22 O10 | 446.12096 | 4.38287613 | 4.57E-08 | 1 | 4.537198885 | up |
| Com_3739_pos | Byakangelicol | C17 H16 O6 | 316.09427 | 4.292366261 | 5.93E-09 | 1 | 4.68168822 | up |
| Com_1979_pos | Genistin | C21 H20 O10 | 432.10525 | 3.955143065 | 5.68E-13 | 1 | 4.408600168 | up |
| Com_2283_pos | Testosterone undecanoate | C30 H48 O3 | 456.35985 | 3.92573209 | 3.99E-14 | 1 | 4.34480232 | up |
| Com_2449_pos | Malonylglycitin | C25 H24 O13 | 532.12162 | 3.909536595 | 5.76E-09 | 1 | 4.164903052 | up |
| Com_655_pos | Testosterone propionate | C22 H32 O3 | 344.23204 | 3.835451496 | 1.26E-06 | 1 | 3.623713083 | up |
| Com_357_pos | Genistein 4'-O-glucuronide | C21 H18 O11 | 446.08458 | 3.79367654 | 1.86E-10 | 1 | 4.486938629 | up |
| Com_2639_pos | Glycitein | C16 H12 O5 | 284.068 | 3.777010085 | 1.49E-09 | 1 | 4.172300022 | up |
| Com_4358_pos | 3,4-dihydroxyphenylacetic acid | C8 H8 O4 | 168.04218 | 3.617849766 | 5.25E-13 | 1 | 4.050711024 | up |
| Com_1306_pos | Carpipramine | C28 H38 N4 O | 446.30044 | 3.585255814 | 0.000382445 | 0.986111111 | 2.931800268 | up |
| Com_811_pos | Daidzein | C15 H10 O4 | 254.0576 | 3.583147386 | 1.11E-08 | 1 | 4.390419608 | up |
| Com_2025_pos | Furfural | C5 H4 O2 | 96.02115 | 3.505535182 | 2.57E-13 | 1 | 3.90123414 | up |
| Com_3474_pos | Daidzin | C21 H20 O9 | 416.11026 | 3.4418192 | 8.91E-14 | 1 | 3.793023978 | up |
| Com_1031_pos | Genistein | C15 H10 O5 | 270.05249 | 3.432281392 | 4.10E-10 | 1 | 4.071330232 | up |
| Com_3706_pos | 2-O-ETHYL ASCORBIC ACID | C8 H12 O6 | 204.06328 | 3.084439425 | 7.13E-15 | 1 | 3.46116681 | up |
| Com_1133_pos | 3',4'-(Dioctyloxy) acetophenone | C24 H40 O3 | 376.29723 | 2.90048172 | 0.000645496 | 0.9375 | 2.652571234 | up |
| Com_12686_pos | 1-{[7-(2-Amino-2-oxoethyl)-10-(3-amino-3-oxopropyl)-13-sec-butyl-16-(4-hydroxybenzyl)  -6,9,12,15,18-pentaoxo-1,2-dithia-5,8,11,14,17-pentaazacycloicosan-4-yl]carbonyl}prolylleucylglycinamide | C43 H65 N11 O12 S2 | 991.42184 | 2.897042883 | 0.000919155 | 0.895833333 | 2.559222489 | up |
| Com_2013_pos | Progesterone | C21 H30 O2 | 314.224 | 2.700639681 | 0.012699316 | 0.763888889 | 2.019670344 | up |
| Com_13735_pos | 2-Acetamido-4-O-[2-(carboxyamino)-2-deoxy-beta-D-glucopyranosyl]-2-deoxy-beta-D-glucopyranose | C15 H26 N2 O12 | 426.15 | 2.640392378 | 5.42E-05 | 0.951388889 | 2.614239619 | up |
| Com_433_pos | L-Cystine | C6 H12 N2 O4 S2 | 240.02357 | 2.636745903 | 0.012753326 | 0.840277778 | 2.565427486 | up |
| Com_13650_pos | 3-(4,9-Dihydrothieno[2,3-c][2]benzothiepin-4-yl)-N,N-dimethyl-1-propanamine | C17 H21 N S2 | 303.11014 | 2.578048836 | 7.87E-07 | 1 | 2.697607775 | up |
| Com_15057_pos | 6-(1-Hydroxyethyl)-3-(hydroxymethyl)-2,7-dioxabicyclo[4.1.0]hept-3-en-5-one | C8 H10 O5 | 186.05268 | 2.55252743 | 6.73E-14 | 1 | 2.86539586 | up |
| Com_3402_pos | (1Z,2E,4E,6E)-N-(2-Hydroxy-5-oxo-1-cyclopenten-1-yl)-2,4,6-octatrienimidic acid | C13 H15 N O3 | 233.10499 | 2.550357435 | 4.09E-06 | 0.958333333 | 2.731573883 | up |
| Com_6396_pos | demethylphylloquinone | C30 H44 O2 | 436.33388 | 2.526294834 | 1.03E-10 | 1 | 2.776094904 | up |
| Com_11426_pos | Secogalioside | C17 H24 O12 | 420.12628 | 2.518789829 | 6.27E-05 | 0.944444444 | 2.466354015 | up |
| Com_654_pos | 7,8-Bis(hydroxymethyl)-1,4a-dimethyl-3,4,4a,5,6,7-hexahydro-2H-benzo[7]annulen-2-one | C15 H22 O3 | 250.15409 | 2.447859322 | 0.000581063 | 0.958333333 | 2.101682642 | up |
| Com_3608_pos | 4-{(Z)-[(4E,7Z,16Z,19Z)-1-Hydroxy-4,7,10,13,16,19-docosahexaen-1-ylidene]amino}butanoic acid | C26 H39 N O3 | 413.29216 | 2.424752214 | 0.018123836 | 0.756944444 | 1.778499389 | up |
| Com_12505_pos | (2R,6S,12Z,13aR,14aR,16aS)-6-{[(Cyclopentyloxy)carbonyl]amino}-2-({2-[2-(isopropylamino)  -1,3-thiazol-4-yl]-7-methoxy-4-quinolinyl}oxy)-5,16-dioxo-1,2,3,6,7,8,9,10,11,13a,14,15,16,16a-  tetradecahydrocyclopropa[e]pyrrolo[1,2-a][1,4]diazacyclopentadecine-14a(5H)-carboxylic acid | C40 H50 N6 O8 S | 774.34189 | 2.406678701 | 2.46E-05 | 0.930555556 | 2.617330114 | up |
| Com_887_pos | 3-Methyl-5-(5,5,8a-trimethyl-2-methylene-7-oxodecahydro-1-naphthalenyl)pentyl acetate | C22 H36 O3 | 348.2654 | 2.35811992 | 0.004658358 | 0.861111111 | 1.829366214 | up |
| Com_313_pos | Ethinylestradiol sulfonate | C23 H30 O4 S | 402.18612 | 2.34709543 | 0.037550931 | 0.6875 | 1.464193047 | up |
| Com_927_pos | (2E,4E,6E,8E,10E,12E,14E)-15-(4-Hydroxy-2,6,6-trimethyl-1-cyclohexen-1-yl)-4,9,13  -trimethyl-2,4,6,8,10,12,14-pentadecaheptaenal | C27 H36 O2 | 392.27175 | 2.33029356 | 0.001646108 | 0.840277778 | 1.999320333 | up |
| Com_5441_pos | 3-Oxoolean-12-en-29-oic acid | C30 H46 O3 | 454.34413 | 2.25918078 | 2.21E-11 | 1 | 2.502552236 | up |
| Com_321_pos | Eicosapentaenoic acid ethyl ester | C22 H34 O2 | 330.25505 | 2.2147183 | 0.006946445 | 0.798611111 | 1.864207855 | up |
| Com_4346_pos | Medroxyprogesterone | C22 H32 O3 | 344.23462 | 2.126192093 | 0.000494712 | 0.881944444 | 1.954736926 | up |
| Com_616_pos | Valylproline | C10 H18 N2 O3 | 214.13167 | 2.100669404 | 0.000159459 | 0.944444444 | 2.132337145 | up |
| Com_11802_pos | benthiavalicarb-isopropyl | C18 H24 F N3 O3 S | 381.1508 | 2.061794651 | 2.93E-06 | 0.986111111 | 2.13381561 | up |
| Com_5284_pos | Isoniazid | C6 H7 N3 O | 137.05891 | 2.011130871 | 0.014136031 | 0.798611111 | 1.38545665 | up |
| Com_740_pos | Promegestone | C22 H30 O2 | 326.2238 | 2.008708449 | 0.001552005 | 0.868055556 | 1.938512981 | up |
| Com_5533_pos | methyl alpha-D-mannoside | C7 H14 O6 | 194.07892 | 1.982622001 | 4.08E-05 | 0.986111111 | 1.99663738 | up |
| Com_1365_pos | 2-Acrylamido-2-methyl-1-propane sulfonic acid | C7 H13 N O4 S | 207.0565 | 1.974450748 | 0.032285594 | 0.6875 | 1.445985182 | up |
| Com_111_pos | Agmatine | C5 H14 N4 | 130.12181 | 1.9712111 | 0.038969342 | 0.756944444 | 1.255184361 | up |
| Com_7237_pos | 3b-Hydroxy-5-cholenoic acid | C24 H38 O3 | 374.28132 | 1.969409298 | 0.010425412 | 0.756944444 | 1.499650389 | up |
| Com_3776_pos | 1-[(Carboxyacetyl)amino]cyclopropanecarboxylic acid | C7 H9 N O5 | 187.04799 | 1.956429452 | 1.41E-07 | 1 | 2.112451822 | up |
| Com_14567_pos | Antioside | C29 H44 O10 | 552.29083 | 1.949146672 | 0.025641801 | 0.652777778 | 1.480132228 | up |
| Com_1139_pos | (2S)-4-(Methylsulfanyl)-2-({[(3S,4S,5R)-2,3,4-trihydroxy-5-(hydroxymethyl)tetrahydro-2-furanyl]methyl}amino)butanoic acid | C11 H21 N O7 S | 311.10333 | 1.897728286 | 0.008047991 | 0.798611111 | 2.116486475 | up |
| Com_8911_pos | Fortimicin FU-10 | C12 H24 N2 O9 | 340.14901 | 1.809655505 | 1.50E-08 | 0.993055556 | 1.99791788 | up |
| Com_13944_pos | metixene | C20 H23 N S | 309.15621 | 1.793352718 | 9.16E-08 | 0.986111111 | 1.988563719 | up |
| Com_20457_pos | Imatinib | C29 H31 N7 O | 493.26128 | 1.785907108 | 0.005464074 | 0.756944444 | 2.054222601 | up |
| Com_1486_pos | Pyrimidine | C4 H4 N2 | 80.03743 | 1.778808932 | 0.000888357 | 0.833333333 | 1.806494784 | up |
| Com_11439_pos | taurolithocholic acid sulfate | C26 H45 N O8 S2 | 563.26123 | 1.724030649 | 0.001253763 | 0.895833333 | 1.553985956 | up |
| Com_5726_pos | 3-hydroxytetradecanoylcarnitine | C21 H41 N O5 | 387.29778 | 1.720993077 | 0.004606517 | 0.805555556 | 1.428723758 | up |
| Com_217_pos | oxiracetam | C6 H10 N2 O3 | 158.06909 | 1.717769632 | 0.001911916 | 0.826388889 | 1.629734799 | up |
| Com_12944_pos | (1R,3S,9R,10S,13R,15E,17E,19E,21E,23R,25S,26R,27S)-23-[(3-Amino-3,6-dideoxy-beta-  D-mannopyranosyl)oxy]-10-ethyl-1,3,9,27-tetrahydroxy-7,11-dioxo-13-propyl-12,29-dioxabicyclo  [23.3.1]nonacosa-15,17,19,21-tetraene-26-carboxylic acid | C39 H61 N O14 | 767.40554 | 1.706730354 | 1.59E-05 | 0.958333333 | 1.787092342 | up |
| Com_12230_pos | Edetol | C14 H32 N2 O4 | 292.2363 | 1.700401055 | 0.000216952 | 0.854166667 | 2.107285947 | up |
| Com_329_pos | Tetrahydrocortisone | C21 H32 O5 | 346.21156 | 1.699697214 | 0.011055991 | 0.805555556 | 1.710342383 | up |
| Com_349_pos | L-(+)-Citrulline | C6 H13 N3 O3 | 175.09565 | 1.669958377 | 0.002743221 | 0.8125 | 1.618239323 | up |
| Com_10596_pos | 4-Pyridoxate | C8 H9 N O4 | 183.05306 | 1.64723116 | 7.17E-05 | 0.923611111 | 1.843602534 | up |
| Com_6321_pos | (3aR,4aS,5R,7aS,8S,9aR)-5-Hydroxy-4a,8-dimethyl-3-methyleneoctahydroazuleno[6,5-b]furan-2,6(3H,4H)-dione | C15 H20 O4 | 264.13577 | 1.612579317 | 0.000498297 | 0.881944444 | 1.707842464 | up |
| Com_8646_pos | phellopterin | C17 H16 O5 | 300.09903 | 1.581003327 | 9.71E-05 | 0.9375 | 1.644088607 | up |
| Com_2823_pos | Leukotriene E3 | C23 H39 N O5 S | 441.25658 | 1.577020591 | 0.009908177 | 0.770833333 | 1.407089081 | up |
| Com_9858_pos | fluprednisolone | C21 H27 F O5 | 378.1824 | 1.568983392 | 0.005290865 | 0.791666667 | 1.378862795 | up |
| Com_5640_pos | Heptylbenzene | C13 H20 | 176.15639 | 1.564416283 | 8.14E-07 | 0.972222222 | 1.745631176 | up |
| Com_17616_pos | trans-Zeatin-7-glucoside | C16 H23 N5 O6 | 381.16302 | 1.558262361 | 0.004770799 | 0.770833333 | 1.50055856 | up |
| Com_1194_pos | 3-[(2-Isopropyl-5-methylcyclohexyl)oxy]-1,2-propanediol | C13 H26 O3 | 230.18787 | 1.530895234 | 0.009838603 | 0.75 | 1.217940281 | up |
| Com_1624_pos | 22-Oxodocosanoic acid | C22 H42 O3 | 354.31285 | 1.513001253 | 0.00375842 | 0.840277778 | 1.354927654 | up |
| Com_1073_pos | 2,6-di-tert-butyl-4-ethylphenol | C16 H26 O | 234.19812 | 1.496840298 | 0.032187196 | 0.722222222 | 1.264283273 | up |
| Com_3934_pos | trihexyphenidyl | C20 H31 N O | 301.23995 | 1.491000802 | 0.038083066 | 0.708333333 | 1.248299851 | up |
| Com_11328_pos | Cyclo(leucylprolyl) | C11 H18 N2 O2 | 210.1368 | 1.486753301 | 0.006699806 | 0.875 | 1.511037329 | up |
| Com_4080_pos | Anandamide | C22 H37 N O2 | 347.28185 | 1.469298816 | 0.037516158 | 0.708333333 | 1.141507317 | up |
| Com_21_pos | L-Norleucine | C6 H13 N O2 | 131.09454 | 1.466681265 | 0.000210945 | 0.902777778 | 1.551316108 | up |
| Com_5235_pos | 4-methylquinolin-2-ol | C10 H9 N O | 159.06902 | 1.440324105 | 0.000131041 | 0.909722222 | 1.792720132 | up |
| Com_4616_pos | Norethisterone enanthate | C27 H38 O3 | 410.28085 | 1.438575362 | 0.021897872 | 0.722222222 | 1.19040436 | up |
| Com_494_pos | Prolylleucine | C11 H20 N2 O3 | 228.14723 | 1.43681264 | 0.006891653 | 0.826388889 | 1.386769896 | up |
| Com_15243_pos | Colcemid | C21 H25 N O5 | 371.17422 | 1.428647569 | 0.00013967 | 0.909722222 | 1.462097732 | up |
| Com_2076_pos | Retinal 2 | C20 H26 O | 282.1978 | 1.421413704 | 0.000157152 | 0.958333333 | 1.367630811 | up |
| Com_1452_pos | frescolat ML | C13 H24 O3 | 228.17218 | 1.413597385 | 0.00695432 | 0.791666667 | 1.158727917 | up |
| Com_15607_pos | 3-Hydroxyflavone | C15 H10 O3 | 238.0626 | 1.400165688 | 0.000915425 | 0.888888889 | 1.445704082 | up |
| Com_1075_pos | L-Theanine | C7 H14 N2 O3 | 174.10043 | 1.382254274 | 0.000359116 | 0.888888889 | 1.511683066 | up |
| Com_7844_pos | Dehydrosoyasaponin I | C48 H76 O18 | 940.50123 | 1.379884101 | 8.56E-06 | 0.930555556 | 1.530680779 | up |
| Com_478_pos | Methionine sulfoxide | C5 H11 N O3 S | 165.04593 | 1.376632358 | 0.001353338 | 0.875 | 1.863353696 | up |
| Com_16983_pos | PIVOPRIL | C16 H27 N O4 S | 329.16491 | 1.342525737 | 0.008158066 | 0.798611111 | 1.255926551 | up |
| Com_757_pos | Bexarotene | C24 H28 O2 | 348.20598 | 1.334921153 | 0.015502845 | 0.770833333 | 1.377505195 | up |
| Com_3563_pos | 3-Desoxy-3,4-methylenedioxy pyrovalerone | C17 H23 N O2 | 273.16855 | 1.33233147 | 0.002407684 | 0.861111111 | 1.437922228 | up |
| Com_6363_pos | 2,4-dihydroxyheptadec-16-enyl acetate | C19 H36 O4 | 328.26074 | 1.326402707 | 0.011458765 | 0.791666667 | 1.246147333 | up |
| Com_5152_pos | 1-palmitoyl-2-stearoyl-sn-glycero-3-phosphoserine | C40 H78 N O10 P | 763.53717 | 1.3196621 | 0.016845506 | 0.798611111 | 1.530846271 | up |
| Com_18781_pos | lycopsamine | C15 H25 N O5 | 299.17277 | 1.315780299 | 0.000434865 | 0.895833333 | 1.540987729 | up |
| Com_438_pos | EDDA | C6 H12 N2 O4 | 176.07964 | 1.310821181 | 0.000598162 | 0.833333333 | 1.279172609 | up |
| Com_3275_pos | Amdoxovir | C9 H12 N6 O3 | 252.09684 | 1.292915733 | 0.00318546 | 0.9375 | 2.097347891 | up |
| Com_20683_pos | 3-O-(3-Methylbutanoyl)-beta-D-fructofuranosyl 3-O-decanoyl-4-O-(3-methylbutanoyl)-alpha-D-glucopyranoside | C32 H56 O14 | 664.36461 | 1.287042836 | 0.018293563 | 0.75 | 1.098926833 | up |
| Com_8093_pos | Dihydrouridine | C9 H14 N2 O6 | 246.08486 | 1.276675819 | 0.000725536 | 0.861111111 | 1.206138798 | up |
| Com_257_pos | Threonine | C4 H9 N O3 | 119.05815 | 1.275679124 | 0.001024586 | 0.881944444 | 1.592931205 | up |
| Com_16739_pos | Linagliptin | C25 H28 N8 O2 | 472.23128 | 1.2717223 | 0.0001051 | 0.888888889 | 1.295472282 | up |
| Com_15288_pos | gibberellin A1 | C19 H24 O6 | 348.15825 | 1.257289187 | 0.025735716 | 0.708333333 | 1.082207448 | up |
| Com_12784_pos | (Z)-3-butylidenephthalide | C12 H12 O2 | 188.08366 | 1.253171933 | 1.67E-05 | 0.979166667 | 1.366052545 | up |
| Com_175_pos | Guanine | C5 H5 N5 O | 151.04929 | 1.243072463 | 0.000119937 | 0.909722222 | 1.488260354 | up |
| Com_9985_pos | Onapristone | C29 H39 N O3 | 449.29085 | 1.241286267 | 0.017065119 | 0.770833333 | 1.189043776 | up |
| Com_7073_pos | 1,3-Di-tert-butylbenzene | C14 H22 | 190.17201 | 1.238130265 | 1.31E-08 | 1 | 1.366495838 | up |
| Com_4018_pos | gamma-Mangostin | C23 H24 O6 | 396.15838 | 1.223359696 | 0.012184296 | 0.770833333 | 1.277834807 | up |
| Com_423_pos | Ricinoleic Acid | C18 H34 O3 | 298.25075 | 1.218918233 | 0.019542742 | 0.784722222 | 1.64020616 | up |
| Com_4189_pos | 6-Hydroxy-1,2-hexanediyl dioctanoate | C22 H42 O5 | 386.30231 | 1.215128792 | 0.014062608 | 0.75 | 1.427285576 | up |
| Com_1016_pos | asp-leu | C10 H18 N2 O5 | 246.12128 | 1.209087294 | 0.000900181 | 0.875 | 1.296412346 | up |
| Com_12730_pos | N-(2,6,10,14-Tetramethylpentadecanoyl)glycine | C21 H41 N O3 | 355.308 | 1.203356298 | 0.007665252 | 0.819444444 | 1.209166944 | up |
| Com_9962_pos | (3alpha,5beta,7alpha,8xi,9xi,12alpha,14xi,25R)-7-(beta-D-Galactopyranosyloxy)-3,12-dihydroxycholestan-27-yl acetate | C35 H60 O10 | 640.41557 | 1.187225936 | 0.020493405 | 0.756944444 | 1.606318045 | up |
| Com_9846_pos | Ethyl methoxycinnamate | C12 H14 O3 | 206.0942 | 1.179770873 | 7.94E-05 | 0.930555556 | 1.318435551 | up |
| Com_11969_pos | Soyasaponin I | C48 H78 O18 | 942.52092 | 1.171823835 | 2.64E-05 | 0.965277778 | 1.226604487 | up |
| Com_2455_pos | acebutolol | C18 H28 N2 O4 | 336.20583 | 1.167561316 | 0.016209025 | 0.777777778 | 1.285185703 | up |
| Com_371_pos | 13-KODE | C18 H30 O3 | 294.21895 | 1.158012397 | 0.008754074 | 0.798611111 | 1.333101454 | up |
| Com_20960_pos | isonixin | C14 H14 N2 O2 | 242.10665 | 1.155549669 | 0.010435868 | 0.770833333 | 1.18229478 | up |
| Com_5890_pos | Androstanolone | C19 H30 O2 | 290.2242 | 1.148975332 | 0.004148282 | 0.916666667 | 1.019973173 | up |
| Com_8659_pos | Calenduloside E | C36 H56 O9 | 632.38963 | 1.145164085 | 0.003382062 | 0.847222222 | 1.240511245 | up |
| Com_9106_pos | Araloside A | C47 H74 O18 | 926.48578 | 1.134166305 | 4.11E-06 | 0.9375 | 1.338590602 | up |
| Com_20881_pos | 9-(alpha-D-glucosyl)kinetin | C16 H19 N5 O6 | 377.13207 | 1.132556676 | 0.005571053 | 0.777777778 | 1.110551277 | up |
| Com_2675_pos | Nitrendipine | C18 H20 N2 O6 | 360.13163 | 1.12571915 | 0.007679102 | 0.791666667 | 1.394410436 | up |
| Com_174_pos | (1S,4aS,5R)-5-[2-(3-Furyl)ethyl]-1,4a-dimethyl-6-methylenedecahydro-1-naphthalenecarboxylic acid | C20 H28 O3 | 316.20081 | 1.112547378 | 7.85E-05 | 0.909722222 | 1.225630596 | up |
| Com_3590_pos | Methyldienolone | C19 H26 O2 | 286.19274 | 1.110021997 | 0.028630704 | 0.722222222 | 1.030522288 | up |
| Com_535_pos | Thymine | C5 H6 N2 O2 | 126.04283 | 1.106998354 | 6.09E-05 | 0.909722222 | 1.268520656 | up |
| Com_13865_pos | tert-Butyl 3-amino-1-methyl-2,3-dioxopropylcarbamate | C9 H16 N2 O4 | 216.11083 | 1.101726271 | 0.004928185 | 0.833333333 | 1.045764332 | up |
| Com_766_pos | 1-Piperideine | C5 H9 N | 83.07352 | 1.100991302 | 0.000131338 | 0.951388889 | 1.102699125 | up |
| Com_39_pos | Piperidine | C5 H11 N | 85.08912 | 1.096893684 | 0.002492232 | 0.868055556 | 1.164303032 | up |
| Com_1525_pos | Tetraacetylethylenediamine | C10 H16 N2 O4 | 228.11077 | 1.096323675 | 0.010070719 | 0.847222222 | 1.285180556 | up |
| Com_17323_pos | (1S,4R,4'S,5'S,6'R,9S,10E,12E,14S,15S,16E,19R,21R)-6'-[(2S)-2-Butanyl]-4',9,15-trihydroxy-5',6,10,14,16  -pentamethyl-3',4',5',6'-tetrahydro-3H,7H-spiro[2,20-dioxatricyclo[17.3.1.0~4,9~]tricosa-5,10,12,16-tetraene-21,2'-pyran]-3,7-dione | C34 H50 O8 | 586.35227 | 1.090539233 | 0.015020004 | 0.791666667 | 1.066841555 | up |
| Com_2138_pos | Propargite | C19 H26 O4 S | 350.15388 | 1.090368339 | 0.004411849 | 0.805555556 | 1.144344513 | up |
| Com_286_pos | Perindoprilat | C17 H28 N2 O5 | 340.20084 | 1.087714087 | 0.000114406 | 0.902777778 | 1.145352981 | up |
| Com_21152_pos | (1alpha,3alpha,6alpha,14alpha,15alpha,16beta)-8-Acetoxy-3,6,10,13,15-pentahydroxy-1,16  -dimethoxy-4-(methoxymethyl)-20-methylaconitan-14-yl benzoate | C32 H43 N O12 | 633.28002 | 1.081782311 | 7.16E-06 | 0.972222222 | 1.134182914 | up |
| Com_121_pos | 9-Oxo-10(E),12(E)-octadecadienoic acid | C18 H30 O3 | 294.21889 | 1.079928665 | 0.000154598 | 0.888888889 | 1.176170386 | up |
| Com_3271_pos | Leu-Gly-Pro | C13 H23 N3 O4 | 285.16848 | 1.064888076 | 0.003130091 | 0.833333333 | 1.03523079 | up |
| Com_5024_pos | Allopregnanolone | C21 H34 O2 | 318.25545 | 1.063388969 | 0.018740225 | 0.770833333 | 1.258105228 | up |
| Com_2_pos | Cinnamic acid | C9 H8 O2 | 148.05233 | 1.055953466 | 0.001008006 | 0.861111111 | 1.128072561 | up |
| Com_3_pos | L-Phenylalanine | C9 H11 N O2 | 165.0789 | 1.054659206 | 0.000980809 | 0.861111111 | 1.127183889 | up |
| Com_18105_pos | EUGENYL GLUCOSIDE | C16 H22 O7 | 326.13677 | 1.052546351 | 0.017030559 | 0.756944444 | 1.042170166 | up |
| Com_4820_pos | lamtidine | C18 H28 N6 O | 344.23264 | 1.050970417 | 0.002286107 | 0.861111111 | 1.071016574 | up |
| Com_2876_pos | Oxaceprol | C7 H11 N O4 | 173.06879 | 1.048679531 | 0.018296139 | 0.770833333 | 1.284658866 | up |
| Com_243_pos | all-cis-4,7,10,13,16-Docosapentaenoic acid | C22 H34 O2 | 330.25524 | 1.048123945 | 0.008363527 | 0.819444444 | 1.269461766 | up |
| Com_283_pos | Pyrrolidine | C4 H9 N | 71.07352 | 1.045681321 | 0.029351452 | 0.791666667 | 1.126737877 | up |
| Com_2469_pos | Methyl alpha-aspartylphenylalaninate | C14 H18 N2 O5 | 294.12113 | 1.0398092 | 5.79E-05 | 0.916666667 | 1.159553073 | up |
| Com_177_pos | Tris(2-butoxyethyl) phosphate | C18 H39 O7 P | 398.24262 | 1.038097524 | 0.045414132 | 0.743055556 | 1.217910313 | up |
| Com_149_pos | heptanethiol | C7 H16 S | 132.09775 | 1.03386637 | 0.003507081 | 0.847222222 | 1.103664346 | up |
| Com_16560_pos | Apiin | C26 H28 O14 | 564.1477 | 1.030421786 | 0.005296384 | 0.798611111 | 1.269791773 | up |
| Com_7963_pos | Pregnanetriol | C21 H36 O3 | 336.2654 | 1.02914064 | 0.016510079 | 0.763888889 | 1.078513665 | up |
| Com_4415_pos | Sulfanilic acid | C6 H7 N O3 S | 173.01469 | 1.026220671 | 0.035311188 | 0.736111111 | 1.097147168 | up |
| Com_13047_pos | 1,2-Dimethoxy-12,13-dimethyl-12,13-dihydro[1,3]benzodioxolo[5,6-c]phenanthridine | C22 H21 N O4 | 363.14635 | 1.022718876 | 0.003434703 | 0.819444444 | 1.073439057 | up |
| Com_183_pos | DL-Lysine | C6 H14 N2 O2 | 146.10546 | 1.020317603 | 0.001095767 | 0.861111111 | 1.025143061 | up |
| Com_197_pos | Pipecolic acid | C6 H11 N O2 | 129.07892 | 1.017684358 | 0.001082053 | 0.861111111 | 1.039002507 | up |
| Com_10463_pos | (2-Hydroxy-2-oxido-1,3,2-dioxaphospholan-4-yl)methyl (9Z,12Z)-9,12-octadecadienoate | C21 H37 O6 P | 416.23406 | 1.011523233 | 0.009901294 | 0.777777778 | 1.066554864 | up |
| Com_8827_pos | Bifeprunox | C24 H23 N3 O2 | 385.17836 | 1.010908383 | 0.011631527 | 0.8125 | 1.21838222 | up |
| Com_20347_pos | (4E,6E,8E)-13-[(4E,6E,8E,12E,14E,16E,19Z)-10,18-Dihydroxy-12,16,19-trimethyl-11,22-  dioxooxacyclodocosa-4,6,8,12,14,16,19-heptaen-2-yl]-3,12-dihydroxy-11-methyl-10-oxo-  4,6,8-tetradecatrien-2-yl 6-deoxy-2,4-di-O-methyl-beta-D-galactopyranoside | C47 H66 O13 | 838.44681 | 1.005943688 | 0.000880026 | 0.895833333 | 1.055375375 | up |
| Com_5543_pos | Dehydrofalcarinone | C17 H20 O | 240.15099 | -1.004299999 | 0.000243193 | 0.895833333 | 1.183904953 | down |
| Com_12167_pos | HC Blue No. 1 | C11 H17 N3 O4 | 255.1216 | -1.008010594 | 0.025404076 | 0.736111111 | 1.117791271 | down |
| Com_4425_pos | quindoxin | C8 H6 N2 O2 | 162.04286 | -1.009921206 | 1.57E-05 | 0.965277778 | 1.083833436 | down |
| Com_8782_pos | 4,4'-Oxydianiline | C12 H12 N2 O | 200.09496 | -1.009951956 | 4.89E-06 | 0.986111111 | 1.0722076 | down |
| Com_21847_pos | (1S,4R,5'S,6'R,9S,10E,12E,14S,15S,16E,19R,21S)-9,15-Dihydroxy-6'-isopropyl-5',6,10,14,16  -pentamethyl-5',6'-dihydro-3H,7H-spiro[2,20-dioxatricyclo[17.3.1.0~4,9~]tricosa-5,10,12,16-tetraene-21,2'-pyran]-3,7-dione | C33 H46 O7 | 554.32176 | -1.01438774 | 4.22E-05 | 0.909722222 | 1.1071338 | down |
| Com_13256_pos | Kyotorphin | C15 H23 N5 O4 | 337.17442 | -1.015919008 | 0.000387846 | 0.930555556 | 1.202463576 | down |
| Com_18733_pos | Valorphin | C44 H60 N8 O12 | 892.42904 | -1.016621408 | 0.006380709 | 0.819444444 | 1.321101539 | down |
| Com_1172_pos | DL-Stachydrine | C7 H13 N O2 | 143.09462 | -1.01734513 | 1.08E-05 | 1 | 1.194999355 | down |
| Com_12713_pos | Loperamide | C29 H33 Cl N2 O2 | 476.22225 | -1.017425317 | 0.002835075 | 0.840277778 | 1.227372557 | down |
| Com_14608_pos | Noramidopyrine | C12 H15 N3 O | 217.12142 | -1.018134769 | 0.008703595 | 0.798611111 | 1.176169508 | down |
| Com_17656_pos | Olopatadine | C21 H23 N O3 | 337.16893 | -1.019005724 | 0.000255243 | 0.923611111 | 1.20100426 | down |
| Com_12231_pos | N(6),N(6)-Dimethyladenine | C7 H9 N5 | 163.08603 | -1.019681105 | 8.96E-07 | 0.979166667 | 1.097813732 | down |
| Com_16990_pos | Lupenone | C30 H48 O | 424.36987 | -1.024197124 | 2.43E-05 | 0.923611111 | 1.095845133 | down |
| Com_15129_pos | Silafluofen | C25 H29 F O2 Si | 408.19164 | -1.026182154 | 0.010483937 | 0.784722222 | 1.27921328 | down |
| Com_13637_pos | piketoprofen | C22 H20 N2 O2 | 344.15123 | -1.028708009 | 0.002725202 | 0.847222222 | 1.165781043 | down |
| Com_8181_pos | g-Butyrobetaine | C7 H15 N O2 | 145.11023 | -1.037110263 | 0.001113889 | 0.881944444 | 1.109184208 | down |
| Com_12591_pos | 4-[(2R)-2-(Aminomethyl)-2-(hydroxymethyl)-5-oxo-1-pyrrolidinyl]-3-(3-pentanylamino)benzoic acid | C18 H27 N3 O4 | 349.19851 | -1.037619261 | 0.006518038 | 0.798611111 | 1.348486853 | down |
| Com_19309_pos | Norgestimate | C23 H31 N O3 | 369.2299 | -1.038914253 | 1.26E-05 | 0.951388889 | 1.104740674 | down |
| Com_13849_pos | 2172 | C23 H29 N5 O | 391.23888 | -1.040472714 | 0.000458184 | 0.888888889 | 1.241176239 | down |
| Com_13208_pos | 5-Hydroxyindoleacetate | C10 H9 N O3 | 191.05816 | -1.054570085 | 1.85E-05 | 0.958333333 | 1.241074501 | down |
| Com_17141_pos | Levallorphan | C19 H25 N O | 283.19324 | -1.054792238 | 7.55E-05 | 0.923611111 | 1.187832335 | down |
| Com_17008_pos | Zolpidem | C19 H21 N3 O | 307.16791 | -1.056672777 | 0.001951009 | 0.881944444 | 1.437807229 | down |
| Com_15520_pos | 1-stearyl estercitric acid | C24 H44 O7 | 444.30836 | -1.057782322 | 0.002253216 | 0.840277778 | 1.097372142 | down |
| Com_1088_pos | Hydantoin-5-propionic acid | C6 H8 N2 O4 | 172.04841 | -1.060256571 | 0.001071087 | 0.840277778 | 1.270426526 | down |
| Com_7629_pos | Butabarbital | C10 H16 N2 O3 | 212.11607 | -1.061174919 | 8.58E-07 | 1 | 1.135709989 | down |
| Com_13000_pos | Camobucol | C33 H50 O4 S2 | 574.31778 | -1.062031091 | 1.13E-06 | 0.993055556 | 1.138624619 | down |
| Com_7863_pos | Allyxycarb | C16 H22 N2 O2 | 274.1677 | -1.062122396 | 0.001054682 | 0.902777778 | 1.030665143 | down |
| Com_3961_pos | 1-Methylguanine | C6 H7 N5 O | 165.06507 | -1.063614355 | 5.14E-05 | 0.923611111 | 1.175182385 | down |
| Com_8580_pos | norhaman | C11 H8 N2 | 168.06875 | -1.064535996 | 6.89E-06 | 0.986111111 | 1.117807811 | down |
| Com_1565_pos | Indole-3-carbidol | C9 H9 N O | 147.0682 | -1.065201632 | 3.19E-06 | 0.972222222 | 1.162314433 | down |
| Com_3497_pos | (3S,6S)-3-(4-Hydroxybenzyl)-6-(hydroxymethyl)-2,5-piperazinedione | C12 H14 N2 O4 | 250.09508 | -1.066798935 | 9.75E-05 | 0.923611111 | 1.104621179 | down |
| Com_13311_pos | lawsone | C10 H6 O3 | 174.03166 | -1.069363365 | 3.47E-05 | 0.944444444 | 1.284569977 | down |
| Com_16620_pos | 5-Methoxybenzimidazole | C8 H8 N2 O | 148.06351 | -1.071701229 | 0.00323745 | 0.833333333 | 1.298625671 | down |
| Com_14381_pos | N-Acetyl-L-phenylalanine | C11 H13 N O3 | 207.08939 | -1.072752559 | 6.19E-08 | 1 | 1.17915649 | down |
| Com_16151_pos | Fusarin C | C23 H29 N O7 | 431.1935 | -1.07635725 | 0.008689557 | 0.791666667 | 1.279925005 | down |
| Com_5290_pos | psychotrine | C28 H36 N2 O4 | 464.26717 | -1.077871745 | 4.41E-05 | 0.909722222 | 1.126703706 | down |
| Com_18275_pos | 6-Methoxy-17-methyl-6,7,8,14-tetradehydro-4,5-epoxymorphinan-3-ol | C18 H19 N O3 | 297.13759 | -1.078402177 | 0.018097717 | 0.770833333 | 1.146294765 | down |
| Com_9209_pos | Tandutinib | C31 H42 N6 O4 | 562.32834 | -1.078967364 | 0.001737172 | 0.854166667 | 1.155287554 | down |
| Com_1475_pos | D-Raffinose | C18 H32 O16 | 504.16898 | -1.079725976 | 6.74E-05 | 0.930555556 | 1.179483956 | down |
| Com_8116_pos | 1-Myristoyl-sn-glycerol 3-phosphate | C17 H35 O7 P | 382.21126 | -1.079787985 | 1.98E-06 | 0.972222222 | 1.181582737 | down |
| Com_9291_pos | (10E)-9,12,13-Trihydroxy-10-octadecenoic acid | C18 H34 O5 | 330.24008 | -1.080126358 | 0.001718347 | 0.861111111 | 1.054653288 | down |
| Com_5249_pos | Benzoic acid | C7 H6 O2 | 122.03676 | -1.081239439 | 0.000361033 | 0.916666667 | 1.046971103 | down |
| Com_4197_pos | Nootkatone | C15 H22 O | 218.16688 | -1.081437255 | 2.02E-06 | 1 | 1.144249825 | down |
| Com_9880_pos | Rolipram | C16 H21 N O3 | 275.15175 | -1.083226392 | 0.000101782 | 0.923611111 | 1.220595563 | down |
| Com_469_pos | Acetylcholine | C7 H15 N O2 | 145.11023 | -1.087456457 | 1.63E-05 | 0.944444444 | 1.164895192 | down |
| Com_12575_pos | Imidafenacin | C20 H21 N3 O | 319.16866 | -1.087914202 | 0.035753638 | 0.743055556 | 1.289867308 | down |
| Com_13204_pos | Mitragynine | C23 H30 N2 O4 | 398.22 | -1.088079559 | 0.000167049 | 0.909722222 | 1.302715292 | down |
| Com_10577_pos | (10S)-Juvenile hormone III diol | C16 H28 O4 | 284.19797 | -1.08877488 | 1.58E-05 | 0.923611111 | 1.252779443 | down |
| Com_20341_pos | guaiapate | C18 H29 N O4 | 323.20812 | -1.09305877 | 0.009579112 | 0.791666667 | 1.118646347 | down |
| Com_1917_pos | Hexadecanedioic acid mono-L-carnitine ester | C23 H43 N O6 | 429.30855 | -1.093128281 | 0.001301987 | 0.881944444 | 1.358199221 | down |
| Com_4122_pos | Valclavam | C14 H23 N3 O6 | 329.15825 | -1.096042679 | 0.015017776 | 0.756944444 | 1.403397119 | down |
| Com_17052_pos | Difeterol | C25 H29 N O2 | 375.21924 | -1.096810421 | 0.000726927 | 0.868055556 | 1.263395307 | down |
| Com_421_pos | Succinic acid | C4 H6 O4 | 118.0266 | -1.103196159 | 0.001287522 | 0.888888889 | 1.278324546 | down |
| Com_13989_pos | Imafen | C11 H13 N3 | 187.11087 | -1.11546889 | 0.007251961 | 0.777777778 | 1.351127126 | down |
| Com_9564_pos | S-[(2E,6E)-farnesyl]-L-cysteine | C18 H31 N O2 S | 325.20623 | -1.115752238 | 2.05E-06 | 0.979166667 | 1.226168999 | down |
| Com_7695_pos | QV1MVO1R | C10 H11 N O4 | 209.06872 | -1.11849548 | 1.52E-05 | 0.979166667 | 1.333434736 | down |
| Com_10081_pos | DL-Cerulenin | C12 H17 N O3 | 223.12043 | -1.120280183 | 2.21E-06 | 1 | 1.304097469 | down |
| Com_8155_pos | sampangine | C15 H8 N2 O | 232.06291 | -1.121671963 | 0.001572584 | 0.909722222 | 1.33453415 | down |
| Com_19678_pos | Eicosapentanoic acid | C20 H30 O2 | 302.22405 | -1.122646431 | 5.84E-05 | 0.951388889 | 1.292356156 | down |
| Com_1678_pos | 3-[(12-Hydroxyoctadecanoyl)oxy]-4-(trimethylammonio)butanoate | C25 H49 N O5 | 443.36046 | -1.130035432 | 0.001574521 | 0.895833333 | 1.467911504 | down |
| Com_16070_pos | prilocaine | C13 H20 N2 O | 220.15738 | -1.131200339 | 2.83E-06 | 0.986111111 | 1.29557623 | down |
| Com_2958_pos | Palmitoylcarnitine | C23 H45 N O4 | 399.33421 | -1.134911371 | 0.000927766 | 0.902777778 | 1.535924794 | down |
| Com_1698_pos | O-oleoylcarnitine | C25 H47 N O4 | 425.34995 | -1.13855067 | 0.001967635 | 0.895833333 | 1.501049663 | down |
| Com_12552_pos | clobutinol | C14 H22 Cl N O | 255.13807 | -1.139643643 | 3.67E-05 | 0.944444444 | 1.247911821 | down |
| Com_12899_pos | 2-Isopropenyl-4,6,8-trimethoxy-9-methyl-2,3-dihydrofuro[2,3-b]quinolin-9-ium | C18 H22 N O4 | 316.15302 | -1.140068597 | 0.001620618 | 0.826388889 | 1.327386848 | down |
| Com_6923_pos | (-)-Prostaglandin E1 | C20 H34 O5 | 354.24038 | -1.142282653 | 1.38E-05 | 0.9375 | 1.250310791 | down |
| Com_1122_pos | Glycylglycylglycine | C6 H11 N3 O4 | 189.07494 | -1.150952363 | 0.000292153 | 0.888888889 | 1.327518062 | down |
| Com_18561_pos | ENADENINE | C10 H13 N5 | 203.11764 | -1.155258031 | 5.81E-06 | 0.951388889 | 1.273991008 | down |
| Com_11135_pos | Ethopabate | C12 H15 N O4 | 237.0998 | -1.165569129 | 0.000201427 | 0.888888889 | 1.289671351 | down |
| Com_1809_pos | L-(+)-Leucine | C6 H13 N O2 | 131.09461 | -1.171381939 | 4.37E-07 | 1 | 1.333546016 | down |
| Com_21987_pos | 2-Methyl-1,2-bis(3-pyridyl)-1-propanol | C14 H16 N2 O | 228.12622 | -1.177748283 | 3.38E-05 | 0.965277778 | 1.44809569 | down |
| Com_5079_pos | trp-lys | C17 H24 N4 O3 | 332.18438 | -1.178086621 | 0.003035249 | 0.805555556 | 1.459289815 | down |
| Com_453_pos | Acetyl-L-carnitine | C9 H17 N O4 | 203.11572 | -1.178497179 | 3.41E-05 | 0.958333333 | 1.426829224 | down |
| Com_4925_pos | Ethylmorphine | C19 H23 N O3 | 313.1732 | -1.183849392 | 0.002431479 | 0.826388889 | 1.294287274 | down |
| Com_11251_pos | oxybutynin | C22 H31 N O3 | 357.22968 | -1.185240907 | 0.000582403 | 0.902777778 | 1.417738312 | down |
| Com_788_pos | Tomelukast | C16 H22 N4 O3 | 318.16866 | -1.194930836 | 0.001653476 | 0.819444444 | 1.533288801 | down |
| Com_9894_pos | Isocarboxazid | C12 H13 N3 O2 | 231.10059 | -1.195083646 | 0.014555308 | 0.756944444 | 1.344941789 | down |
| Com_11109_pos | Nefopam | C17 H19 N O | 253.14639 | -1.196184798 | 1.42E-07 | 0.993055556 | 1.319685346 | down |
| Com_2162_pos | N-Palmitoyl-L-tyrosine | C25 H41 N O4 | 419.30296 | -1.19789022 | 3.06E-05 | 0.930555556 | 1.3497645 | down |
| Com_7391_pos | N-Undecanoylglycine | C13 H25 N O3 | 243.1832 | -1.200115801 | 6.01E-05 | 0.9375 | 1.36821707 | down |
| Com_16470_pos | (2R)-2-Hydroxy-3-(phosphonooxy)propyl (11Z)-11-icosenoate | C23 H45 O7 P | 464.2882 | -1.200121276 | 0.000131267 | 0.923611111 | 1.274113187 | down |
| Com_1573_pos | (4S)-4-[(11-Carboxyundecanoyl)oxy]-4-(trimethylammonio)butanoate | C19 H35 N O6 | 373.24589 | -1.206759133 | 0.000117545 | 0.923611111 | 1.320244743 | down |
| Com_11210_pos | 4-[(3S,4R)-4-(4-Fluorophenyl)-3-hexanyl]phenol | C18 H21 F O | 272.15885 | -1.211267984 | 1.24E-05 | 0.944444444 | 1.289814632 | down |
| Com_8660_pos | Sarpogrelate | C24 H31 N O6 | 429.21676 | -1.214576893 | 0.003459527 | 0.8125 | 1.247098256 | down |
| Com_15506_pos | Embelin | C17 H26 O4 | 294.18229 | -1.21521012 | 2.29E-06 | 0.993055556 | 1.36575536 | down |
| Com_1340_pos | N-Arachidonoyl-L-serine | C23 H37 N O4 | 391.27168 | -1.217018094 | 0.000295485 | 0.881944444 | 1.44771807 | down |
| Com_19443_pos | 6-HYDROXYMETHYL-7,8-DIHYDROPTERIN | C7 H9 N5 O2 | 195.07587 | -1.218822633 | 2.24E-07 | 1 | 1.355225067 | down |
| Com_12968_pos | Tetroxoprim | C16 H22 N4 O4 | 334.1638 | -1.224170172 | 0.0013369 | 0.826388889 | 1.453279332 | down |
| Com_11827_pos | Indecainide | C20 H24 N2 O | 308.18829 | -1.224495103 | 9.65E-06 | 0.965277778 | 1.35872569 | down |
| Com_7848_pos | Atropine | C17 H23 N O3 | 289.16737 | -1.225945754 | 0.000149657 | 0.909722222 | 1.47858338 | down |
| Com_13474_pos | Orphenadrine | C18 H23 N O | 269.1776 | -1.229896223 | 2.63E-06 | 0.993055556 | 1.300544677 | down |
| Com_4577_pos | trans-geranic acid | C10 H16 O2 | 168.1146 | -1.235329586 | 0.006758115 | 0.861111111 | 1.168489222 | down |
| Com_13055_pos | phenacetin | C10 H13 N O2 | 179.09449 | -1.248022871 | 1.69E-07 | 1 | 1.337718064 | down |
| Com_12389_pos | HEPES | C8 H18 N2 O4 S | 238.09895 | -1.24893137 | 0.01162245 | 0.791666667 | 1.436177317 | down |
| Com_12516_pos | 3-[2-(Hydroxymethyl)-4-methoxyphenyl]-6-methoxy-4-oxo-3,4-dihydro-1(2H)-quinazolinecarbaldehyde | C18 H18 N2 O5 | 342.12102 | -1.258825969 | 0.006393191 | 0.791666667 | 1.199751059 | down |
| Com_1082_pos | N-Sulfanilyl-3,4-xylamide | C15 H16 N2 O3 S | 304.08762 | -1.263628858 | 0.00048542 | 0.902777778 | 1.451510149 | down |
| Com_14601_pos | PD123319 | C31 H32 N4 O3 | 508.24886 | -1.26791346 | 0.008137706 | 0.8125 | 1.325221342 | down |
| Com_19849_pos | (3S,8aS)-3-(4-Hydroxybenzyl)hexahydropyrrolo[1,2-a]pyrazine-1,4-dione | C14 H16 N2 O3 | 260.11635 | -1.271395465 | 1.27E-06 | 0.972222222 | 1.43579767 | down |
| Com_20061_pos | (2Z,4E,6E,8E,10E,12E,14Z)-15-(6-Hydroxy-4,4,7a-trimethyl-2,4,5,6,7,7a-hexahydro-  1-benzofuran-2-yl)-2,6,11-trimethyl-2,4,6,8,10,12,14-hexadecaheptaenal | C30 H40 O3 | 448.2993 | -1.274718448 | 0.000408398 | 0.881944444 | 1.416439067 | down |
| Com_21909_pos | 8-Hydroxyamoxapine | C17 H16 Cl N3 O2 | 329.09413 | -1.276558077 | 0.00022317 | 0.958333333 | 1.23095035 | down |
| Com_8011_pos | amquinate | C18 H24 N2 O3 | 316.17801 | -1.277211236 | 0.000299389 | 0.875 | 1.475316625 | down |
| Com_17588_pos | Norgestrienone | C20 H22 O2 | 294.16278 | -1.279927102 | 0.000170491 | 0.923611111 | 1.455794473 | down |
| Com_2610_pos | O-octanoyl-L-carnitine | C15 H29 N O4 | 287.20921 | -1.281660345 | 4.96E-08 | 1 | 1.399499015 | down |
| Com_20110_pos | Aurachin D | C25 H33 N O | 363.25511 | -1.285818302 | 0.000127607 | 0.923611111 | 1.284953407 | down |
| Com_2750_pos | saccharopine | C11 H20 N2 O6 | 276.13163 | -1.285859631 | 0.00010771 | 0.895833333 | 1.363895771 | down |
| Com_5687_pos | Cerivastatin | C26 H34 F N O5 | 459.24387 | -1.286215268 | 6.63E-05 | 0.923611111 | 1.425977189 | down |
| Com_14346_pos | libenzapril | C18 H25 N3 O5 | 363.17791 | -1.288767175 | 0.019907825 | 0.770833333 | 1.107975464 | down |
| Com_2657_pos | Myriocin | C21 H39 N O6 | 401.27715 | -1.289215715 | 4.27E-08 | 1 | 1.413077512 | down |
| Com_12432_pos | Diphenylpyraline | C19 H23 N O | 281.17749 | -1.293082457 | 4.83E-06 | 0.979166667 | 1.422158227 | down |
| Com_9054_pos | 2-(1-Hydroxy-2,4,6-trimethyl-3-oxo-2,3-dihydro-1H-inden-5-yl)ethyl acetate | C16 H20 O4 | 276.13598 | -1.300382224 | 8.28E-07 | 1 | 1.501447009 | down |
| Com_7095_pos | levorphanol | C17 H23 N O | 257.17761 | -1.307779019 | 7.49E-05 | 0.909722222 | 1.423485043 | down |
| Com_17335_pos | (1S,4aS)-1,4a-Dimethyl-1,2,3,4,4a,5,6,8a-octahydronaphthalene | C12 H20 | 164.15654 | -1.312873103 | 5.92E-05 | 0.9375 | 1.415267131 | down |
| Com_5428_pos | Salmeterol | C25 H37 N O4 | 415.27169 | -1.31645114 | 0.00107905 | 0.895833333 | 1.757294241 | down |
| Com_5124_pos | JWH 213 | C27 H29 N O | 383.23025 | -1.318411411 | 2.26E-05 | 0.944444444 | 1.383690926 | down |
| Com_808_pos | 2,3-Bis(octanoyloxy)propanimidic acid | C19 H35 N O5 | 357.25101 | -1.319695353 | 3.54E-08 | 1 | 1.483283964 | down |
| Com_18691_pos | Glutathionylaminopropylcadaverine | C18 H36 N6 O5 S | 448.24704 | -1.32777915 | 2.20E-06 | 0.972222222 | 1.504613129 | down |
| Com_1240_pos | Gly-Lys | C8 H17 N3 O3 | 203.1269 | -1.328563175 | 8.67E-06 | 0.951388889 | 1.478386166 | down |
| Com_3238_pos | MYRISTOYLLEVOCARNITINE | C21 H41 N O4 | 371.30292 | -1.332150467 | 5.31E-07 | 0.993055556 | 1.505085114 | down |
| Com_2813_pos | N-Benzyl-4-piperidone | C12 H15 N O | 189.11522 | -1.332892226 | 0.00056814 | 0.854166667 | 1.845039703 | down |
| Com_3329_pos | JWH-147 | C27 H27 N O | 381.21462 | -1.333878921 | 6.34E-06 | 0.965277778 | 1.407361674 | down |
| Com_3404_pos | picoprazole | C17 H17 N3 O3 S | 343.09842 | -1.339885488 | 0.000245486 | 0.902777778 | 1.550210611 | down |
| Com_1828_pos | Ethyl violet | C31 H41 N3 | 455.32434 | -1.348604016 | 3.22E-06 | 0.979166667 | 1.539399553 | down |
| Com_17362_pos | EPTAZOCINE | C15 H21 N O | 231.16205 | -1.358191987 | 6.48E-06 | 0.979166667 | 1.421164849 | down |
| Com_18787_pos | Balfourodinine | C17 H22 N O4 | 304.15325 | -1.362576696 | 0.000305807 | 0.930555556 | 1.396421905 | down |
| Com_14557_pos | Sirolimus | C51 H79 N O13 | 913.55965 | -1.365866334 | 0.000464161 | 0.868055556 | 2.007331849 | down |
| Com_3740_pos | Leukotriene B4 | C20 H32 O4 | 336.22942 | -1.372211285 | 0.008642062 | 0.840277778 | 1.494510622 | down |
| Com_13798_pos | 2E-Crotamiton | C13 H17 N O | 203.13096 | -1.374001432 | 0.000341808 | 0.868055556 | 1.489050549 | down |
| Com_15749_pos | Alprenolol | C15 H23 N O2 | 249.17253 | -1.381529517 | 4.32E-07 | 0.993055556 | 1.556631664 | down |
| Com_18726_pos | 4-Amino-1-{(2xi)-5-O-[hydroxy({hydroxy[(2R)-2-[(9Z)-9-octadecenoyloxy]-3-(stearoyloxy)  propoxy]phosphoryl}oxy)phosphoryl]-beta-D-threo-pentofuranosyl}-2(1H)-pyrimidinone | C48 H87 N3 O15 P2 | 1007.56437 | -1.382150172 | 0.000435693 | 0.902777778 | 1.32945082 | down |
| Com_15792_pos | 3-Methylcyclohexanethiol | C7 H14 S | 130.081 | -1.383681606 | 9.90E-10 | 1 | 1.50337578 | down |
| Com_14833_pos | 6-(2-Amino-2-carboxyethyl)-7,8-dioxo-1,2,3,4,7,8-hexahydro-2,4-quinolinedicarboxylic acid | C14 H14 N2 O8 | 338.07517 | -1.384482078 | 1.80E-06 | 0.979166667 | 1.516498561 | down |
| Com_16347_pos | Isopropyl methoxy pyrazine | C8 H12 N2 O | 152.09426 | -1.392597507 | 5.85E-05 | 0.9375 | 1.409744601 | down |
| Com_20977_pos | 6-[8-Hydroxy-1-(hydroxymethyl)octahydro-2H-quinolizin-3-yl]-2-piperidinone | C15 H26 N2 O3 | 282.1937 | -1.392960258 | 0.00063922 | 0.902777778 | 1.283877167 | down |
| Com_17912_pos | 4-Amino-1-{(2xi)-5-O-[({[(2R)-2-[(10Z,13Z,16Z)-10,13,16-docosatrienoyloxy]-3-(stearoyloxy)  propoxy](hydroxy)phosphoryl}oxy)(hydroxy)phosphoryl]-beta-D-threo-pentofuranosyl}-2(1H)-pyrimidinone | C52 H91 N3 O15 P2 | 1059.59543 | -1.398428603 | 0.004612684 | 0.930555556 | 1.126118211 | down |
| Com_18291_pos | demethoxycurcumin | C20 H18 O5 | 338.11386 | -1.399068424 | 4.08E-05 | 0.930555556 | 1.542480373 | down |
| Com_6389_pos | indeloxazine | C14 H17 N O2 | 231.12563 | -1.406370109 | 1.47E-05 | 0.958333333 | 1.582541563 | down |
| Com_19614_pos | FLUTRIMAZOLE | C22 H16 F2 N2 | 346.12718 | -1.409864393 | 2.49E-07 | 1 | 1.564244614 | down |
| Com_20066_pos | Echimidine | C20 H31 N O7 | 397.21001 | -1.411105451 | 3.43E-06 | 0.965277778 | 1.561595857 | down |
| Com_10468_pos | 1-Glyceryl stearate | C25 H46 O6 | 442.32947 | -1.420206307 | 0.00172469 | 0.847222222 | 1.387992549 | down |
| Com_7914_pos | 6-hydroxypseudooxynicotine | C10 H14 N2 O2 | 194.10554 | -1.428301405 | 1.19E-07 | 1 | 1.564981552 | down |
| Com_19897_pos | 3,3',3'',3''',3''''-[8,13-Bis(carboxymethyl)-18-methyl-2,3,7,12,17-porphyrinpentayl]pentapropanoic acid | C40 H40 N4 O14 | 800.25017 | -1.429534663 | 1.07E-06 | 0.965277778 | 1.640526857 | down |
| Com_13088_pos | (6R,7S)-6,7-Dihydroxy-8-methyl-8-azabicyclo[3.2.1]oct-3-yl (2E)-2-methyl-2-butenoate | C13 H21 N O4 | 255.14669 | -1.436686033 | 0.000139195 | 0.9375 | 1.468955414 | down |
| Com_3621_pos | XLR11 N-(2-fluoropentyl) isomer | C21 H28 F N O | 329.21977 | -1.446599573 | 1.43E-06 | 0.965277778 | 1.601777608 | down |
| Com_1519_pos | Fluoxetine | C17 H18 F3 N O | 309.13201 | -1.448567581 | 5.56E-06 | 0.972222222 | 1.633488938 | down |
| Com_18226_pos | (2R,3R)-N-{4-[(Diaminomethylene)amino]butyl}-5-[(1E)-3-({4-[(diaminomethylene)amino]  butyl}amino)-3-oxo-1-propen-1-yl]-2-(4-hydroxyphenyl)-2,3-dihydro-1-benzofuran-3-carboxamide | C28 H38 N8 O4 | 550.30106 | -1.453048495 | 6.13E-06 | 0.958333333 | 1.563845809 | down |
| Com_12976_pos | PGF2a ethanolamide | C22 H39 N O5 | 397.28203 | -1.456191664 | 1.13E-05 | 0.965277778 | 1.502034966 | down |
| Com_17476_pos | 3,7-Dimethyl-1,6-octadien-3-yl 2-aminobenzoate | C17 H23 N O2 | 273.17247 | -1.456986028 | 0.001506186 | 0.868055556 | 1.493037233 | down |
| Com_13529_pos | 4-Acetyl-1,3-benzoxazol-2(3H)-one | C9 H7 N O3 | 177.04255 | -1.458131927 | 4.00E-06 | 0.965277778 | 1.667208394 | down |
| Com_13370_pos | Geranyl formate | C11 H18 O2 | 182.13062 | -1.460576015 | 0.000367177 | 0.958333333 | 1.38220394 | down |
| Com_16000_pos | Imiprothrin | C17 H22 N2 O4 | 318.15775 | -1.468693294 | 1.42E-06 | 0.993055556 | 1.697991016 | down |
| Com_15770_pos | Sequifenadine | C22 H27 N O | 321.20872 | -1.469068929 | 2.88E-05 | 0.958333333 | 1.830963092 | down |
| Com_1273_pos | Cadralazine | C12 H21 N5 O3 | 283.16398 | -1.470311898 | 4.38E-07 | 0.979166667 | 1.657147071 | down |
| Com_21849_pos | (8E)-9-(1,3-Benzodioxol-5-yl)-1-(1-piperidinyl)-8-nonen-1-one | C21 H29 N O3 | 343.21562 | -1.472130074 | 2.70E-06 | 0.958333333 | 1.606631545 | down |
| Com_8276_pos | Geranyllinalool | C20 H34 O | 290.26043 | -1.488069957 | 0.000727164 | 0.895833333 | 1.432942077 | down |
| Com_4928_pos | glutethimide | C13 H15 N O2 | 217.11007 | -1.489673503 | 0.000153312 | 0.895833333 | 1.712382616 | down |
| Com_4468_pos | N-Palmitoyl taurine | C18 H37 N O4 S | 363.24038 | -1.490972709 | 1.81E-05 | 0.972222222 | 1.527602639 | down |
| Com_13570_pos | Tavaborole | C7 H6 B F O2 | 152.04374 | -1.499017246 | 0.000887821 | 0.881944444 | 1.868418491 | down |
| Com_14587_pos | MPTP N-OXIDE | C12 H15 N O | 189.11516 | -1.501968108 | 3.51E-06 | 0.986111111 | 1.6013084 | down |
| Com_4342_pos | Methyl-2-aminobenzoate | C8 H9 N O2 | 151.06318 | -1.505069916 | 2.31E-06 | 0.993055556 | 1.711136953 | down |
| Com_1700_pos | Dihomo-gamma-linolenic acid | C20 H34 O2 | 306.25531 | -1.507376083 | 0.007496667 | 0.777777778 | 1.525918899 | down |
| Com_1687_pos | (2E)-hexadecenoylcarnitine | C23 H43 N O4 | 397.31858 | -1.52542684 | 1.35E-07 | 0.979166667 | 1.706125105 | down |
| Com_13765_pos | PYRIMIDIFEN | C20 H28 Cl N3 O2 | 377.18651 | -1.542266837 | 0.000235975 | 0.916666667 | 1.91824583 | down |
| Com_783_pos | Cholecalciferol | C27 H44 O | 384.33869 | -1.543598827 | 0.004217745 | 0.881944444 | 2.340770328 | down |
| Com_7614_pos | 3-Hydroxy-cis-5-tetradecenoylcarnitine | C21 H39 N O5 | 385.28215 | -1.549873868 | 4.93E-08 | 1 | 1.733021324 | down |
| Com_10300_pos | Cyclazocine | C18 H25 N O | 271.19326 | -1.563662145 | 2.46E-07 | 0.993055556 | 1.695931637 | down |
| Com_17176_pos | saxagliptin | C18 H25 N3 O2 | 315.19411 | -1.568666816 | 0.001129539 | 0.847222222 | 1.784437858 | down |
| Com_1554_pos | N-Acetylanthranilic acid | C9 H9 N O3 | 179.05817 | -1.571449955 | 2.77E-06 | 1 | 1.808251075 | down |
| Com_1623_pos | Tetrahydrodeoxycorticosterone | C21 H34 O3 | 334.24928 | -1.572142857 | 0.012974237 | 0.8125 | 1.951750979 | down |
| Com_3295_pos | trans-2-Tetradecenoylcarnitine | C21 H39 N O4 | 369.2874 | -1.606349632 | 1.02E-08 | 1 | 1.786495761 | down |
| Com_1796_pos | Decanoylcarnitine | C17 H33 N O4 | 315.24036 | -1.628429395 | 2.12E-08 | 1 | 1.721162036 | down |
| Com_1544_pos | bis(3-aminopropyl)amine | C6 H17 N3 | 131.14221 | -1.63420728 | 0.00019727 | 0.923611111 | 1.763996111 | down |
| Com_5721_pos | 4-tert-Octylphenol monoethoxylate | C16 H26 O2 | 250.19427 | -1.637475363 | 0.000608196 | 0.868055556 | 1.712360391 | down |
| Com_5004_pos | zindotrine | C11 H15 N5 | 217.13329 | -1.642275817 | 6.96E-08 | 0.986111111 | 1.826396081 | down |
| Com_17539_pos | trifloxystrobin | C20 H19 F3 N2 O4 | 408.13166 | -1.658189553 | 6.63E-07 | 0.993055556 | 1.939223033 | down |
| Com_2197_pos | 8-hydroxy-7-methylguanine | C6 H7 N5 O2 | 181.06024 | -1.660011373 | 0.000821649 | 0.895833333 | 2.002003912 | down |
| Com_20707_pos | Bitertanol | C20 H23 N3 O2 | 337.17859 | -1.672854783 | 2.94E-05 | 0.986111111 | 1.634085916 | down |
| Com_17986_pos | 2,3-dinor-8-epi-prostaglandin F2alpha | C18 H30 O5 | 326.20999 | -1.694555564 | 1.35E-06 | 0.993055556 | 1.748473922 | down |
| Com_4828_pos | 3-[(3-Hydroxytridecanoyl)oxy]-4-(trimethylammonio)butanoate | C20 H39 N O5 | 373.28223 | -1.725848106 | 1.13E-11 | 1 | 1.876488707 | down |
| Com_17855_pos | pratosartan | C25 H26 N6 O | 426.21792 | -1.72809694 | 1.21E-08 | 1 | 1.945788197 | down |
| Com_7418_pos | PYROQUILON | C11 H11 N O | 173.084 | -1.731573257 | 7.46E-06 | 0.986111111 | 1.767994231 | down |
| Com_3020_pos | CONDELPHINE | C25 H39 N O6 | 449.27732 | -1.734638172 | 8.77E-07 | 0.972222222 | 1.895945236 | down |
| Com_1412_pos | 3-[(6-Oxodecanoyl)oxy]-4-(trimethylammonio)butanoate | C17 H31 N O5 | 329.21971 | -1.749563271 | 1.38E-09 | 1 | 1.883173851 | down |
| Com_1420_pos | (4S)-4-[(2E,4Z)-2,4-Decadienoyloxy]-4-(trimethylammonio)butanoate | C17 H29 N O4 | 311.20912 | -1.753177057 | 1.54E-09 | 1 | 1.886319868 | down |
| Com_1321_pos | Omeprazole | C17 H19 N3 O3 S | 345.11413 | -1.765281739 | 3.41E-05 | 0.951388889 | 2.050177734 | down |
| Com_1304_pos | 9-Methoxy-2-(methylsulfanyl)-4,9-dihydro[1,3]thiazino[6,5-b]indole | C12 H12 N2 O S2 | 264.03878 | -1.768694448 | 0.00173104 | 0.895833333 | 2.99528726 | down |
| Com_7309_pos | Xanthotoxol | C11 H6 O4 | 202.02661 | -1.778776155 | 0.038500502 | 0.777777778 | 1.166080177 | down |
| Com_17488_pos | 3113 | C17 H26 N2 O4 S | 354.16068 | -1.787631094 | 3.29E-06 | 0.972222222 | 2.016037745 | down |
| Com_4772_pos | 3-[(3-Hydroxydecanoyl)oxy]-4-(trimethylammonio)butanoate | C17 H33 N O5 | 331.23533 | -1.795376211 | 2.48E-08 | 1 | 1.978005191 | down |
| Com_15725_pos | CILAZAPRILAT | C20 H27 N3 O5 | 389.19468 | -1.795569449 | 8.16E-07 | 0.986111111 | 1.931162551 | down |
| Com_19679_pos | (4S)-4-{[2-O-(beta-L-Arabinofuranosyl)-beta-L-arabinofuranosyl]oxy}proline | C15 H25 N O11 | 395.1425 | -1.797917408 | 2.59E-06 | 0.979166667 | 1.989370841 | down |
| Com_4147_pos | UNII:OUT5YHB7BO | C18 H35 N O2 | 297.26627 | -1.803129506 | 1.23E-06 | 0.951388889 | 2.033243886 | down |
| Com_2041_pos | 3-(indol-3-yl)-2-oxobutyric acid | C12 H11 N O3 | 217.07363 | -1.810361596 | 3.15E-07 | 1 | 1.97689637 | down |
| Com_7492_pos | Xylitol | C5 H12 O5 | 152.06837 | -1.814508294 | 5.19E-08 | 1 | 1.891647875 | down |
| Com_11324_pos | sedamine | C14 H21 N O | 219.16216 | -1.829607587 | 3.02E-07 | 1 | 1.974955731 | down |
| Com_15543_pos | Ankorine | C19 H29 N O4 | 335.20914 | -1.840150293 | 3.70E-09 | 1 | 2.067906917 | down |
| Com_1843_pos | Valproic acid | C8 H16 O2 | 144.11475 | -1.840652467 | 9.42E-11 | 1 | 1.983538508 | down |
| Com_19319_pos | Naltrexone | C20 H23 N O4 | 341.16357 | -1.848567951 | 1.33E-06 | 0.972222222 | 2.050919912 | down |
| Com_17796_pos | Fananserin | C23 H24 F N3 O2 S | 425.1584 | -1.88201221 | 7.27E-07 | 0.993055556 | 2.210116753 | down |
| Com_2383_pos | 3-Methoxyflavone | C16 H12 O3 | 274.05944 | -1.902745271 | 7.41E-05 | 0.930555556 | 2.38817398 | down |
| Com_4300_pos | 2-Amino-1,3,4-octadecanetriol | C18 H39 N O3 | 317.29232 | -1.909189637 | 3.44E-05 | 0.909722222 | 2.044454888 | down |
| Com_6829_pos | Linoleamide | C18 H33 N O | 279.25565 | -1.914123283 | 1.90E-06 | 0.965277778 | 2.107646976 | down |
| Com_898_pos | 9-Decenoylcarnitine | C17 H31 N O4 | 313.22478 | -1.916636932 | 2.22E-10 | 1 | 2.062348954 | down |
| Com_2180_pos | 2978 | C14 H16 O2 | 216.11487 | -1.921696665 | 1.85E-05 | 0.972222222 | 2.267811276 | down |
| Com_3932_pos | (-)-Lupinine | C10 H19 N O | 169.1475 | -1.925910921 | 0.000137259 | 0.916666667 | 2.311649523 | down |
| Com_7364_pos | 3-hydroxyoctanoylcarnitine | C15 H29 N O5 | 303.2041 | -1.931061024 | 1.25E-09 | 1 | 2.065588134 | down |
| Com_2718_pos | (2E)-3-(Carbamimidoylsulfanyl)acrylic acid | C4 H6 N2 O2 S | 146.01463 | -1.936860874 | 0.017741831 | 0.763888889 | 2.263587773 | down |
| Com_5448_pos | O-nonanoylcarnitine | C16 H31 N O4 | 301.22475 | -1.94802007 | 4.04E-10 | 1 | 2.146561736 | down |
| Com_2207_pos | Metolachlor morpholinone | C14 H19 N O2 | 233.14136 | -1.959946594 | 2.17E-05 | 0.972222222 | 2.326178655 | down |
| Com_9264_pos | (2E,4E,6E,8E,10E)-1-[5-Hydroxy-2-(2-hydroxyethyl)-2H-pyrrol-4-yl]-10-(hydroxymethyl)  -2,6,8-trimethyl-2,4,6,8,10-dodecapentaen-1-one | C22 H29 N O4 | 371.21138 | -1.976983769 | 0.000313109 | 0.895833333 | 2.398268754 | down |
| Com_12874_pos | Chromocarb | C10 H6 O4 | 190.02659 | -1.979674515 | 5.79E-09 | 1 | 2.190024342 | down |
| Com_905_pos | Octylamine | C8 H19 N | 129.15173 | -1.983441792 | 3.43E-06 | 0.972222222 | 2.095234002 | down |
| Com_10009_pos | bisphenol A | C15 H16 O2 | 228.11459 | -1.998281891 | 0.001591858 | 0.909722222 | 1.730186555 | down |
| Com_1651_pos | (8E)-2-Amino-8-octadecene-1,3,4-triol | C18 H37 N O3 | 315.27672 | -2.050386476 | 5.79E-05 | 0.895833333 | 2.481308723 | down |
| Com_15025_pos | Dimepiperate | C15 H21 N O S | 263.13411 | -2.051916765 | 1.32E-07 | 1 | 2.144602706 | down |
| Com_15145_pos | Artemotil | C17 H28 O5 | 312.1946 | -2.05890446 | 1.63E-09 | 1 | 2.295062213 | down |
| Com_3052_pos | PD-128042 | C23 H39 N O4 | 393.28723 | -2.060515982 | 3.87E-10 | 1 | 2.265712036 | down |
| Com_5200_pos | piroximone | C11 H11 N3 O2 | 217.08503 | -2.06463582 | 1.73E-07 | 1 | 2.250744283 | down |
| Com_4558_pos | Methylphenidate | C14 H19 N O2 | 233.14132 | -2.082678409 | 3.47E-08 | 1 | 2.247649896 | down |
| Com_2715_pos | Palmitic Acid | C16 H32 O2 | 256.23971 | -2.103225423 | 0.00019602 | 0.986111111 | 1.947332848 | down |
| Com_2002_pos | Oleamide | C18 H35 N O | 281.27133 | -2.118417234 | 3.10E-06 | 0.965277778 | 2.579580163 | down |
| Com_1588_pos | THREO-SPHINGOSINE, (-)- | C18 H37 N O2 | 299.28195 | -2.136109011 | 4.03E-06 | 0.965277778 | 2.637130867 | down |
| Com_2537_pos | (4S)-4-[(2E)-2-Octenoyloxy]-4-(trimethylammonio)butanoate | C15 H27 N O4 | 285.19369 | -2.161632065 | 5.02E-11 | 1 | 2.391940374 | down |
| Com_6200_pos | Oleoylethanolamide | C20 H39 N O2 | 325.29743 | -2.17597125 | 0.00022833 | 0.965277778 | 2.019760206 | down |
| Com_10462_pos | Ethyl levulinate | C7 H12 O3 | 144.07866 | -2.179568455 | 5.22E-10 | 1 | 2.367915174 | down |
| Com_7846_pos | 6721 | C16 H20 N6 | 296.17382 | -2.229953931 | 5.58E-05 | 0.958333333 | 2.136112976 | down |
| Com_1989_pos | Debromohymenialdisine | C11 H11 N5 O2 | 245.08964 | -2.340566795 | 2.26E-07 | 1 | 2.452581599 | down |
| Com_6881_pos | Civetone | C17 H30 O | 250.22931 | -2.349248028 | 0.000778406 | 0.902777778 | 2.059244791 | down |
| Com_1170_pos | Hydroxylysine | C6 H14 N2 O3 | 162.10045 | -2.366192559 | 0.02790605 | 0.673611111 | 1.780708451 | down |
| Com_4034_pos | 3beta-Fluoro-5beta-pregnan-20-one | C21 H33 F O | 320.25136 | -2.374197321 | 0.000179572 | 1 | 2.184527574 | down |
| Com_2404_pos | androstenol | C19 H30 O | 274.22917 | -2.411512427 | 4.53E-07 | 0.958333333 | 2.74642009 | down |
| Com_7272_pos | tretoquinol | C19 H23 N O5 | 345.1568 | -2.43259523 | 5.36E-06 | 0.993055556 | 2.439217892 | down |
| Com_10949_pos | Mesalazine | C7 H7 N O3 | 153.04249 | -2.44110502 | 6.63E-05 | 0.979166667 | 2.326498276 | down |
| Com_14162_pos | Pirenzepine | C19 H21 N5 O2 | 351.16763 | -2.46967573 | 1.12E-05 | 0.951388889 | 2.518636445 | down |
| Com_1277_pos | Dicyclomine | C19 H35 N O2 | 309.26621 | -2.49223587 | 3.00E-07 | 0.965277778 | 2.84541216 | down |
| Com_15386_pos | Epristeride | C25 H37 N O3 | 399.27677 | -2.495477382 | 1.34E-05 | 0.965277778 | 2.419940194 | down |
| Com_5063_pos | METIPRANOLOL | C17 H27 N O4 | 309.19365 | -2.52914652 | 8.08E-09 | 1 | 2.777403326 | down |
| Com_3048_pos | Ethyl malate | C8 H14 O5 | 190.08408 | -2.548815333 | 2.13E-10 | 1 | 2.830409273 | down |
| Com_9547_pos | Trp-Phe | C20 H21 N3 O3 | 351.15769 | -2.595428673 | 2.44E-08 | 1 | 2.717165792 | down |
| Com_14904_pos | primidone | C12 H14 N2 O2 | 218.10556 | -2.601965223 | 0.047266965 | 0.75 | 1.47758249 | down |
| Com_16389_pos | Cucurbitacin E | C32 H44 O8 | 556.30333 | -2.626760055 | 0.020014741 | 0.868055556 | 1.398125846 | down |
| Com_15682_pos | nicotine imine | C10 H13 N2 | 161.10688 | -2.722786693 | 0.001219237 | 0.951388889 | 2.05880869 | down |
| Com_5058_pos | Dipivefrin | C19 H29 N O5 | 351.20398 | -2.980427302 | 4.89E-07 | 1 | 3.028975537 | down |
| Com_10677_pos | jesaconitine | C35 H49 N O12 | 675.32236 | -3.341803711 | 6.08E-06 | 0.979166667 | 3.127670529 | down |
| Com_2293_pos | amfonelic acid | C18 H16 N2 O3 | 308.11558 | -3.748451505 | 1.32E-10 | 1 | 4.220201888 | down |

| **Supplementary Table 13. The significnat differential metabolites in DI contents of SBM40 vs. FM in positive mode (n = 12)** | | | | | | | | | |
| --- | --- | --- | --- | --- | --- | --- | --- | --- | --- |
|
| ID | Name_des | Formula | Molecular Weight | FC | log2FC | Pvalue | AUC | VIP | Up.Down |
| Com_10039_pos | O-{Hydroxy[(2R)-2-(palmitoyloxy)-3-(tetradecanoyloxy)propoxy]phosphoryl}-L-serine | C36 H70 N O10 P | 707.47515 | 4.284942 | 2.099276 | 0.001422 | 0.819444 | 1.190382 | up |
| Com_1031_pos | Genistein | C15 H10 O5 | 270.05249 | 15.6432 | 3.967464 | 1.46E-10 | 1 | 3.282617 | up |
| Com_10348_pos | TG(18:2(9Z,12Z)/18:2(9Z,12Z)/20:0)[iso3] | C59 H106 O6 | 910.79534 | 3.125066 | 1.643887 | 0.002445 | 0.854167 | 1.115005 | up |
| Com_10412_pos | Gibberellin A4 | C19 H24 O5 | 332.16361 | 2.988216 | 1.579285 | 5.40E-06 | 0.986111 | 1.258379 | up |
| Com_10596_pos | 4-Pyridoxate | C8 H9 N O4 | 183.05306 | 2.497861 | 1.320693 | 0.002267 | 0.847222 | 1.212032 | up |
| Com_1073_pos | 2,6-di-tert-butyl-4-ethylphenol | C16 H26 O | 234.19812 | 3.074059 | 1.620145 | 1.76E-05 | 0.9375 | 1.194857 | up |
| Com_10829_pos | (E)-Chalcone | C15 H12 O | 208.08951 | 3.532073 | 1.820515 | 0.000472 | 0.923611 | 1.499536 | up |
| Com_11328_pos | Cyclo(leucylprolyl) | C11 H18 N2 O2 | 210.1368 | 3.223235 | 1.688509 | 1.30E-05 | 0.951389 | 1.42968 | up |
| Com_1133_pos | 3',4'-(Dioctyloxy) acetophenone | C24 H40 O3 | 376.29723 | 18.67365 | 4.222932 | 5.82E-08 | 0.986111 | 3.053376 | up |
| Com_11426_pos | Secogalioside | C17 H24 O12 | 420.12628 | 3.082329 | 1.624021 | 0.001944 | 0.833333 | 1.245997 | up |
| Com_11439_pos | taurolithocholic acid sulfate | C26 H45 N O8 S2 | 563.26123 | 4.234516 | 2.082197 | 5.28E-05 | 0.9375 | 1.45604 | up |
| Com_11802_pos | benthiavalicarb-isopropyl | C18 H24 F N3 O3 S | 381.1508 | 3.964804 | 1.98725 | 5.74E-07 | 1 | 1.511023 | up |
| Com_1194_pos | 3-[(2-Isopropyl-5-methylcyclohexyl)oxy]-1,2-propanediol | C13 H26 O3 | 230.18787 | 7.556046 | 2.917632 | 5.61E-05 | 0.965278 | 1.749284 | up |
| Com_11969_pos | Soyasaponin I | C48 H78 O18 | 942.52092 | 3.75926 | 1.910449 | 3.15E-07 | 1 | 1.415212 | up |
| Com_12054_pos | 13-(3,4-Dimethyl-5-pentyl-2-furyl)tridecanoic acid | C24 H42 O3 | 378.31208 | 2.425939 | 1.278543 | 0.000373 | 0.916667 | 1.125236 | up |
| Com_12063_pos | CE(15:1) | C42 H72 O2 | 608.55552 | 5.150734 | 2.364778 | 2.47E-06 | 1 | 1.562759 | up |
| Com_12170_pos | Docosatetraenoylethanolamide | C24 H41 N O2 | 375.31337 | 3.556609 | 1.830502 | 0.000485 | 0.895833 | 1.206955 | up |
| Com_121_pos | 9-Oxo-10(E),12(E)-octadecadienoic acid | C18 H30 O3 | 294.21889 | 2.752405 | 1.460693 | 1.01E-06 | 0.972222 | 1.065292 | up |
| Com_12230_pos | Edetol | C14 H32 N2 O4 | 292.2363 | 2.518608 | 1.332626 | 0.003644 | 0.826389 | 1.030835 | up |
| Com_12292_pos | Cholest-5-en-3-yl beta-D-glucopyranosiduronic acid | C33 H54 O7 | 562.38718 | 3.568946 | 1.835498 | 4.69E-05 | 0.979167 | 1.314826 | up |
| Com_12505_pos | (2R,6S,12Z,13aR,14aR,16aS)-6-{[(Cyclopentyloxy)carbonyl]amino}  -2-({2-[2-(isopropylamino)-1,3-thiazol-4-yl]-7-methoxy-4-quinolinyl}  oxy)-5,16-dioxo-1,2,3,6,7,8,9,10,11,13a,14,15,16,16a-  tetradecahydrocyclopropa[e]pyrrolo[1,2-a][1,4]diazacyclopentadecine  -14a(5H)-carboxylic acid | C40 H50 N6 O8 S | 774.34189 | 3.744335 | 1.90471 | 0.004019 | 0.819444 | 1.688414 | up |
| Com_12686_pos | 1-{[7-(2-Amino-2-oxoethyl)-10-(3-amino-3-oxopropyl)-13-sec-butyl  -16-(4-hydroxybenzyl)-6,9,12,15,18-pentaoxo-1,2-dithia-5,8,11,14,17  -pentaazacycloicosan-4-yl]carbonyl}prolylleucylglycinamide | C43 H65 N11 O12 S2 | 991.42184 | 2.590538 | 1.373252 | 0.030596 | 0.666667 | 1.033996 | up |
| Com_12730_pos | N-(2,6,10,14-Tetramethylpentadecanoyl)glycine | C21 H41 N O3 | 355.308 | 2.835217 | 1.503459 | 1.37E-05 | 0.944444 | 1.144145 | up |
| Com_12765_pos | (+)-Riboflavin | C17 H20 N4 O6 | 376.13942 | 3.356615 | 1.747007 | 0.00039 | 0.881944 | 1.525228 | up |
| Com_12809_pos | phe-asn | C13 H17 N3 O4 | 279.12124 | 2.869624 | 1.520862 | 0.000645 | 0.888889 | 1.272599 | up |
| Com_12941_pos | Enpromate | C22 H23 N O2 | 333.17247 | 3.074044 | 1.620138 | 0.001795 | 0.819444 | 1.012384 | up |
| Com_12944_pos | (1R,3S,9R,10S,13R,15E,17E,19E,21E,23R,25S,26R,27S)-23-  [(3-Amino-3,6-dideoxy-beta-D-mannopyranosyl)oxy]-10-ethyl  -1,3,9,27-tetrahydroxy-7,11-dioxo-13-propyl-12,29-dioxabicyclo  [23.3.1]nonacosa-15,17,19,21-tetraene-26-carboxylic acid | C39 H61 N O14 | 767.40554 | 4.034767 | 2.012485 | 1.12E-07 | 1 | 1.568016 | up |
| Com_1306_pos | Carpipramine | C28 H38 N4 O | 446.30044 | 54.74612 | 5.774685 | 5.82E-08 | 1 | 3.831617 | up |
| Com_1317_pos | STS-135 | C24 H31 F N2 O | 382.24774 | 4.003434 | 2.001238 | 0.003509 | 0.840278 | 1.369906 | up |
| Com_13650_pos | 3-(4,9-Dihydrothieno[2,3-c][2]benzothiepin-4-yl)-N,N-dimethyl-  1-propanamine | C17 H21 N S2 | 303.11014 | 5.237219 | 2.388801 | 1.52E-05 | 0.993056 | 1.832695 | up |
| Com_1365_pos | 2-Acrylamido-2-methyl-1-propane sulfonic acid | C7 H13 N O4 S | 207.0565 | 9.57237 | 3.258876 | 8.96E-06 | 0.923611 | 2.259105 | up |
| Com_13735_pos | 2-Acetamido-4-O-[2-(carboxyamino)-2-deoxy-beta-D-  glucopyranosyl]-2-deoxy-beta-D-glucopyranose | C15 H26 N2 O12 | 426.15 | 2.636528 | 1.398639 | 0.012391 | 0.743056 | 1.145912 | up |
| Com_13795_pos | bucromarone | C29 H37 N O4 | 463.27211 | 2.503957 | 1.32421 | 8.55E-07 | 1 | 1.015841 | up |
| Com_13944_pos | metixene | C20 H23 N S | 309.15621 | 5.419285 | 2.438102 | 1.36E-08 | 1 | 1.798547 | up |
| Com_1452_pos | frescolat ML | C13 H24 O3 | 228.17218 | 6.442374 | 2.687593 | 4.20E-05 | 0.993056 | 1.640994 | up |
| Com_14577_pos | indaconitine | C34 H47 N O10 | 629.32176 | 2.338781 | 1.225757 | 0.001044 | 0.923611 | 1.217125 | up |
| Com_14645_pos | (4S)-4-{[(11Z)-3-Hydroxy-11-octadecenoyl]oxy}-4-  (trimethylammonio)butanoate | C25 H47 N O5 | 441.34462 | 3.702588 | 1.888534 | 0.000913 | 0.854167 | 1.111471 | up |
| Com_14920_pos | 4-{(Z)-[(5E,8Z,11Z,14Z)-1-Hydroxy-5,8,11,14-  icosatetraen-1-ylidene]amino}butanoic acid | C24 H39 N O3 | 389.29195 | 3.971185 | 1.989569 | 8.30E-07 | 0.979167 | 1.376816 | up |
| Com_15057_pos | 6-(1-Hydroxyethyl)-3-(hydroxymethyl)-2,7-  dioxabicyclo[4.1.0]hept-3-en-5-one | C8 H10 O5 | 186.05268 | 7.502742 | 2.907418 | 1.38E-15 | 1 | 2.290702 | up |
| Com_15243_pos | Colcemid | C21 H25 N O5 | 371.17422 | 4.807184 | 2.265192 | 0.000611 | 0.895833 | 1.386113 | up |
| Com_15288_pos | gibberellin A1 | C19 H24 O6 | 348.15825 | 4.724628 | 2.240201 | 3.32E-06 | 0.972222 | 1.550795 | up |
| Com_15370_pos | Salvinorin A | C23 H28 O8 | 432.17814 | 2.645014 | 1.403275 | 1.88E-05 | 0.944444 | 1.140897 | up |
| Com_15607_pos | 3-Hydroxyflavone | C15 H10 O3 | 238.0626 | 2.438814 | 1.28618 | 0.00092 | 0.916667 | 1.076557 | up |
| Com_1624_pos | 22-Oxodocosanoic acid | C22 H42 O3 | 354.31285 | 6.957852 | 2.798642 | 2.95E-07 | 0.986111 | 1.898458 | up |
| Com_16664_pos | Ergosterol | C28 H44 O | 396.33842 | 2.534169 | 1.341513 | 4.58E-07 | 1 | 1.021613 | up |
| Com_16682_pos | 17beta-(Acetylthio)estra-1,3,5(10)-trien-3-ol acetate | C22 H28 O3 S | 372.17494 | 2.512217 | 1.328961 | 8.96E-06 | 0.951389 | 1.014065 | up |
| Com_174_pos | (1S,4aS,5R)-5-[2-(3-Furyl)ethyl]-1,4a-dimethyl-6-  methylenedecahydro-1-naphthalenecarboxylic acid | C20 H28 O3 | 316.20081 | 2.792857 | 1.481742 | 9.28E-07 | 0.979167 | 1.085124 | up |
| Com_177_pos | Tris(2-butoxyethyl) phosphate | C18 H39 O7 P | 398.24262 | 2.752749 | 1.460873 | 0.000116 | 0.916667 | 1.273461 | up |
| Com_1806_pos | 1-Phenyl-1,3-octadecanedione | C24 H38 O2 | 358.28667 | 4.100043 | 2.035639 | 0.000144 | 0.909722 | 1.496972 | up |
| Com_18576_pos | 1-hexadecyl-sn-glycerol 3-phosphate | C19 H41 O6 P | 396.26409 | 2.697115 | 1.431417 | 0.000159 | 0.902778 | 1.026211 | up |
| Com_1857_pos | 2-oxo-8-methylthiooctanoic acid | C9 H16 O3 S | 204.08237 | 3.292534 | 1.719198 | 4.48E-05 | 0.944444 | 1.161667 | up |
| Com_18582_pos | 6-O-[Bis(diisopropylamino)acetyl]hexonic acid | C20 H40 N2 O8 | 436.27944 | 4.290809 | 2.10125 | 2.30E-05 | 0.958333 | 1.405441 | up |
| Com_185_pos | Uric acid | C5 H4 N4 O3 | 168.02831 | 2.522188 | 1.334676 | 0.001341 | 0.875 | 1.447995 | up |
| Com_196_pos | N-Acetyl-D-galactosamine | C8 H15 N O6 | 221.08983 | 5.92686 | 2.567268 | 4.35E-07 | 0.979167 | 1.919264 | up |
| Com_1979_pos | Genistin | C21 H20 O10 | 432.10525 | 32.4191 | 5.018772 | 7.39E-10 | 1 | 3.592424 | up |
| Com_2013_pos | Progesterone | C21 H30 O2 | 314.224 | 11.59666 | 3.535638 | 1.02E-05 | 0.9375 | 2.37004 | up |
| Com_20246_pos | 9-ribosylzeatin | C15 H21 N5 O5 | 351.15383 | 3.178815 | 1.668489 | 7.04E-05 | 0.951389 | 1.144888 | up |
| Com_2025_pos | Furfural | C5 H4 O2 | 96.02115 | 9.369826 | 3.228022 | 2.54E-13 | 1 | 2.55637 | up |
| Com_20347_pos | (4E,6E,8E)-13-[(4E,6E,8E,12E,14E,16E,19Z)-10,18-  Dihydroxy-12,16,19-trimethyl-11,22-dioxooxacyclodocosa-  4,6,8,12,14,16,19-heptaen-2-yl]-3,12-dihydroxy-11-methyl  -10-oxo-4,6,8-tetradecatrien-2-yl 6-deoxy-2,4-di-O-methyl-  beta-D-galactopyranoside | C47 H66 O13 | 838.44681 | 2.423317 | 1.276983 | 9.28E-06 | 0.972222 | 1.027928 | up |
| Com_2076_pos | Retinal 2 | C20 H26 O | 282.1978 | 8.125598 | 3.022474 | 2.13E-05 | 1 | 1.854933 | up |
| Com_21152_pos | (1alpha,3alpha,6alpha,14alpha,15alpha,16beta)-8-Acetoxy  -3,6,10,13,15-pentahydroxy-1,16-dimethoxy-4-  (methoxymethyl)-20-methylaconitan-14-yl benzoate | C32 H43 N O12 | 633.28002 | 4.576958 | 2.194389 | 1.87E-13 | 1 | 1.692271 | up |
| Com_2115_pos | 2-(14,15-Epoxyeicosatrienoyl) glycerol | C23 H38 O5 | 394.27135 | 3.329293 | 1.735216 | 0.001229 | 0.868056 | 1.246286 | up |
| Com_2138_pos | Propargite | C19 H26 O4 S | 350.15388 | 3.542617 | 1.824815 | 8.28E-06 | 0.951389 | 1.289092 | up |
| Com_223_pos | chitobiose, di-N-acetyl | C16 H28 N2 O11 | 424.1689 | 23.50172 | 4.554695 | 0.000136 | 0.958333 | 2.640969 | up |
| Com_2283_pos | Testosterone undecanoate | C30 H48 O3 | 456.35985 | 16.91489 | 4.080222 | 4.88E-14 | 1 | 3.185884 | up |
| Com_243_pos | all-cis-4,7,10,13,16-Docosapentaenoic acid | C22 H34 O2 | 330.25524 | 3.205789 | 1.68068 | 1.26E-05 | 0.958333 | 1.376011 | up |
| Com_2449_pos | Malonylglycitin | C25 H24 O13 | 532.12162 | 16.70246 | 4.061989 | 1.74E-08 | 1 | 3.058492 | up |
| Com_2455_pos | acebutolol | C18 H28 N2 O4 | 336.20583 | 4.256702 | 2.089736 | 2.34E-06 | 0.958333 | 1.628147 | up |
| Com_2639_pos | Glycitein | C16 H12 O5 | 284.068 | 18.12198 | 4.179668 | 1.79E-07 | 1 | 3.232877 | up |
| Com_280_pos | 1,2,3,4-Tetramethyl-1,3-cyclopentadiene | C9 H14 | 122.10933 | 2.699545 | 1.432716 | 3.83E-05 | 0.930556 | 1.152636 | up |
| Com_2823_pos | Leukotriene E3 | C23 H39 N O5 S | 441.25658 | 3.016259 | 1.59276 | 6.03E-05 | 0.902778 | 1.151043 | up |
| Com_296_pos | N-Acetyllactosamine | C14 H25 N O11 | 383.14233 | 12.64314 | 3.660283 | 0.000851 | 0.875 | 1.992766 | up |
| Com_3100_pos | 7a-Hydroxytestosterone | C19 H28 O3 | 304.2035 | 4.98918 | 2.318803 | 0.019969 | 0.715278 | 1.143491 | up |
| Com_313_pos | Ethinylestradiol sulfonate | C23 H30 O4 S | 402.18612 | 18.3288 | 4.19604 | 0.000502 | 0.854167 | 2.334089 | up |
| Com_321_pos | Eicosapentaenoic acid ethyl ester | C22 H34 O2 | 330.25505 | 14.89587 | 3.89684 | 3.79E-05 | 0.930556 | 2.422588 | up |
| Com_3269_pos | Glycitin | C22 H22 O10 | 446.12096 | 31.27045 | 4.966728 | 1.48E-10 | 1 | 3.668025 | up |
| Com_329_pos | Tetrahydrocortisone | C21 H32 O5 | 346.21156 | 4.10186 | 2.036278 | 1.23E-05 | 0.930556 | 1.655614 | up |
| Com_3331_pos | Isoxaben | C18 H24 N2 O4 | 332.17222 | 3.574113 | 1.837585 | 2.97E-05 | 0.951389 | 1.41789 | up |
| Com_3378_pos | 5-methylbenzimidazole | C8 H8 N2 | 132.0683 | 21.95108 | 4.45622 | 0.000102 | 0.923611 | 3.128086 | up |
| Com_3402_pos | (1Z,2E,4E,6E)-N-(2-Hydroxy-5-oxo-1-cyclopenten-  1-yl)-2,4,6-octatrienimidic acid | C13 H15 N O3 | 233.10499 | 4.457282 | 2.156164 | 5.38E-06 | 0.9375 | 1.840239 | up |
| Com_3474_pos | Daidzin | C21 H20 O9 | 416.11026 | 22.35942 | 4.482811 | 2.05E-09 | 1 | 3.174317 | up |
| Com_3483_pos | 5-hydroxyisouric acid | C5 H4 N4 O4 | 184.02325 | 3.746879 | 1.905689 | 4.06E-05 | 0.923611 | 1.714141 | up |
| Com_357_pos | Genistein 4'-O-glucuronide | C21 H18 O11 | 446.08458 | 16.28285 | 4.025282 | 2.84E-10 | 1 | 3.385268 | up |
| Com_3590_pos | Methyldienolone | C19 H26 O2 | 286.19274 | 2.806634 | 1.488841 | 9.96E-05 | 0.902778 | 1.081395 | up |
| Com_3706_pos | 2-O-ETHYL ASCORBIC ACID | C8 H12 O6 | 204.06328 | 12.8729 | 3.686266 | 2.02E-16 | 1 | 2.868129 | up |
| Com_371_pos | 13-KODE | C18 H30 O3 | 294.21895 | 2.943521 | 1.557543 | 0.000102 | 0.9375 | 1.286319 | up |
| Com_3739_pos | Byakangelicol | C17 H16 O6 | 316.09427 | 14.37443 | 3.845432 | 1.77E-07 | 0.993056 | 3.068754 | up |
| Com_3776_pos | 1-[(Carboxyacetyl)amino]cyclopropanecarboxylic acid | C7 H9 N O5 | 187.04799 | 4.941363 | 2.304909 | 3.46E-06 | 0.972222 | 1.714296 | up |
| Com_3934_pos | trihexyphenidyl | C20 H31 N O | 301.23995 | 4.025974 | 2.009338 | 0.000305 | 0.868056 | 1.357052 | up |
| Com_4018_pos | gamma-Mangostin | C23 H24 O6 | 396.15838 | 3.04615 | 1.606987 | 0.000181 | 0.916667 | 1.206493 | up |
| Com_402_pos | Eicosapentaenoic acid methyl ester | C21 H32 O2 | 316.23958 | 2.42943 | 1.280618 | 0.000164 | 0.923611 | 1.099045 | up |
| Com_4080_pos | Anandamide | C22 H37 N O2 | 347.28185 | 8.84787 | 3.14533 | 0.000481 | 0.930556 | 1.743446 | up |
| Com_4140_pos | Propoxur | C11 H15 N O3 | 209.10486 | 27.6474 | 4.789072 | 7.13E-12 | 1 | 3.762591 | up |
| Com_4153_pos | Lurasidone | C28 H36 N4 O2 S | 492.25583 | 11.18534 | 3.483537 | 2.92E-05 | 0.986111 | 2.585076 | up |
| Com_4189_pos | 6-Hydroxy-1,2-hexanediyl dioctanoate | C22 H42 O5 | 386.30231 | 2.565468 | 1.359222 | 0.000365 | 0.854167 | 1.205287 | up |
| Com_423_pos | Ricinoleic Acid | C18 H34 O3 | 298.25075 | 3.210228 | 1.682676 | 0.000549 | 0.930556 | 1.552611 | up |
| Com_433_pos | L-Cystine | C6 H12 N2 O4 S2 | 240.02357 | 11.00658 | 3.460294 | 1.08E-05 | 0.944444 | 2.67807 | up |
| Com_4346_pos | Medroxyprogesterone | C22 H32 O3 | 344.23462 | 12.06003 | 3.592161 | 0.000492 | 0.881944 | 2.054702 | up |
| Com_4358_pos | 3,4-dihydroxyphenylacetic acid | C8 H8 O4 | 168.04218 | 10.61704 | 3.408309 | 1.63E-12 | 1 | 2.721336 | up |
| Com_4616_pos | Norethisterone enanthate | C27 H38 O3 | 410.28085 | 3.114309 | 1.638912 | 1.41E-07 | 0.979167 | 1.195073 | up |
| Com_4627_pos | 2-Acetamido-4-(5-amino-2,2-dimethyl-4-oxo-3,4  -dihydro-2H-chromen-6-yl)-4-oxobutyl (10E,12E)  -10,12-octadecadienoate | C35 H52 N2 O6 | 596.38085 | 2.597483 | 1.377114 | 0.000937 | 0.895833 | 1.365599 | up |
| Com_4712_pos | Testosterone phenylpropionate | C28 H36 O3 | 420.26538 | 10.78509 | 3.430966 | 0.008658 | 0.715278 | 1.637079 | up |
| Com_478_pos | Methionine sulfoxide | C5 H11 N O3 S | 165.04593 | 2.081216 | 1.057427 | 0.005446 | 0.8125 | 1.099256 | up |
| Com_4806_pos | (5,10,10-Trihydroxy-2,6-diiminooctahydro-  1H,8H-pyrrolo[1,2-c]purin-4-yl)methyl  hydroxycarbamate | C10 H17 N7 O6 | 331.12366 | 15.1986 | 3.925866 | 9.32E-08 | 0.993056 | 3.247219 | up |
| Com_5024_pos | Allopregnanolone | C21 H34 O2 | 318.25545 | 3.103271 | 1.63379 | 2.79E-05 | 0.979167 | 1.374167 | up |
| Com_5060_pos | Biotin sulfone | C10 H16 N2 O5 S | 276.07761 | 3.415089 | 1.771923 | 0.02242 | 0.770833 | 1.188831 | up |
| Com_5152_pos | 1-palmitoyl-2-stearoyl-sn-glycero-3-phosphoserine | C40 H78 N O10 P | 763.53717 | 4.337174 | 2.116755 | 3.40E-06 | 0.944444 | 1.7873 | up |
| Com_5264_pos | Furomine | C20 H32 N2 O4 | 364.23729 | 3.093526 | 1.629252 | 1.95E-05 | 0.965278 | 1.375119 | up |
| Com_5284_pos | Isoniazid | C6 H7 N3 O | 137.05891 | 8.644559 | 3.111792 | 0.0004 | 0.875 | 1.763564 | up |
| Com_5441_pos | 3-Oxoolean-12-en-29-oic acid | C30 H46 O3 | 454.34413 | 5.649285 | 2.498068 | 1.32E-13 | 1 | 1.951166 | up |
| Com_5533_pos | methyl alpha-D-mannoside | C7 H14 O6 | 194.07892 | 2.882486 | 1.527314 | 0.002866 | 0.881944 | 1.210735 | up |
| Com_5549_pos | 2,2'-([(2R,5R)-2-Benzyl-3,6-dihydroxy-5-(hydroxymethyl)  -2,5-dihydropyrazine-2,5-diyl]bis{sulfanediyl[(1Z,2R)-2  -amino-1-hydroxy-3-propyl-1-ylidene](Z)azanylylidene})  diacetic acid (non-preferred name) | C22 H30 N6 O9 S2 | 586.15095 | 15.14803 | 3.921058 | 6.59E-05 | 0.909722 | 2.939332 | up |
| Com_5560_pos | Medrogestone | C23 H32 O2 | 340.23964 | 4.240404 | 2.084202 | 0.000522 | 0.902778 | 1.270395 | up |
| Com_5640_pos | Heptylbenzene | C13 H20 | 176.15639 | 3.742322 | 1.903934 | 5.42E-12 | 1 | 1.546223 | up |
| Com_5726_pos | 3-hydroxytetradecanoylcarnitine | C21 H41 N O5 | 387.29778 | 5.86202 | 2.551398 | 9.95E-06 | 0.979167 | 1.676497 | up |
| Com_5779_pos | 1-arachidonoyl-sn-glycero-3-phosphocholine | C28 H50 N O7 P | 543.32995 | 2.924576 | 1.548227 | 5.68E-05 | 0.916667 | 1.150671 | up |
| Com_578_pos | Neolinustatin | C17 H29 N O11 | 423.17375 | 28.02237 | 4.808507 | 0.000179 | 0.902778 | 2.810836 | up |
| Com_5824_pos | N-pentylpantothenamide | C14 H28 N2 O4 | 288.2059 | 3.979513 | 1.992592 | 6.42E-07 | 0.979167 | 1.520546 | up |
| Com_5890_pos | Androstanolone | C19 H30 O2 | 290.2242 | 4.894134 | 2.291053 | 0.001051 | 0.840278 | 1.339605 | up |
| Com_610_pos | a-Linolenoyl ethanolamide | C20 H35 N O2 | 321.26621 | 3.469892 | 1.794891 | 0.000872 | 0.868056 | 1.171522 | up |
| Com_6396_pos | demethylphylloquinone | C30 H44 O2 | 436.33388 | 5.924825 | 2.566772 | 8.37E-14 | 1 | 1.951749 | up |
| Com_645_pos | N-[(1S)-4-Carbamimidamido-1-carboxybutyl]asparaginylaspartic acid | C14 H24 N6 O8 | 404.16512 | 12.43188 | 3.635973 | 0.004738 | 0.847222 | 1.981632 | up |
| Com_654_pos | 7,8-Bis(hydroxymethyl)-1,4a-dimethyl-3,4,4a,5,6,7-  hexahydro-2H-benzo[7]annulen-2-one | C15 H22 O3 | 250.15409 | 14.71964 | 3.879671 | 9.31E-07 | 1 | 2.506132 | up |
| Com_655_pos | Testosterone propionate | C22 H32 O3 | 344.23204 | 51.82598 | 5.695604 | 5.27E-08 | 1 | 3.753188 | up |
| Com_6936_pos | (2R,2'R,4a'S,6'R,8a'S)-4,6'-Dihydroxy-2',5',5',8a'  -tetramethyl-3',4',4a',5',6',7,7',8,8',8a'-decahydro-  2'H-spiro[furo[2,3-E]isoindole-2,1'-naphthalen]-6(3H)-one | C23 H31 N O4 | 385.23049 | 3.444753 | 1.784401 | 0.002576 | 0.840278 | 1.188386 | up |
| Com_6948_pos | Diosgenin | C27 H42 O3 | 414.3126 | 4.534806 | 2.181041 | 0.044747 | 0.722222 | 1.178111 | up |
| Com_7066_pos | (8E,15Z)-1,8,15-Heptadecatriene-11,13-diyne | C17 H22 | 226.17179 | 9.212866 | 3.20365 | 0.002141 | 0.798611 | 1.629306 | up |
| Com_7237_pos | 3b-Hydroxy-5-cholenoic acid | C24 H38 O3 | 374.28132 | 9.467015 | 3.24291 | 7.81E-06 | 0.986111 | 2.075285 | up |
| Com_736_pos | Eicosapentaenoic acid | C20 H30 O2 | 302.2239 | 6.406544 | 2.679546 | 6.16E-05 | 0.9375 | 1.671599 | up |
| Com_740_pos | Promegestone | C22 H30 O2 | 326.2238 | 8.398349 | 3.070106 | 3.15E-05 | 0.909722 | 2.036824 | up |
| Com_757_pos | Bexarotene | C24 H28 O2 | 348.20598 | 3.385805 | 1.759499 | 5.17E-05 | 0.923611 | 1.395348 | up |
| Com_7670_pos | 1-Naphthaleneacetic acid | C12 H10 O2 | 186.06873 | 3.151105 | 1.655858 | 0.001143 | 0.875 | 1.271552 | up |
| Com_768_pos | Arachidonic acid | C20 H32 O2 | 304.23968 | 3.381961 | 1.75786 | 0.009833 | 0.770833 | 1.062625 | up |
| Com_7844_pos | Dehydrosoyasaponin I | C48 H76 O18 | 940.50123 | 5.459686 | 2.448818 | 4.34E-11 | 1 | 1.937605 | up |
| Com_7935_pos | N-Stearoylglycine | C20 H39 N O3 | 341.29231 | 3.009532 | 1.589539 | 6.22E-06 | 0.958333 | 1.141751 | up |
| Com_7963_pos | Pregnanetriol | C21 H36 O3 | 336.2654 | 2.793033 | 1.481832 | 7.27E-05 | 0.909722 | 1.135736 | up |
| Com_8093_pos | Dihydrouridine | C9 H14 N2 O6 | 246.08486 | 3.801349 | 1.926512 | 5.99E-05 | 0.9375 | 1.295329 | up |
| Com_811_pos | Daidzein | C15 H10 O4 | 254.0576 | 13.30441 | 3.733833 | 9.76E-09 | 1 | 3.261106 | up |
| Com_8199_pos | Docosatetraenoic acid | C22 H36 O2 | 332.27096 | 4.184551 | 2.065073 | 0.000111 | 0.9375 | 1.288034 | up |
| Com_8646_pos | phellopterin | C17 H16 O5 | 300.09903 | 3.085369 | 1.625443 | 0.001805 | 0.861111 | 1.279641 | up |
| Com_8659_pos | Calenduloside E | C36 H56 O9 | 632.38963 | 2.414332 | 1.271624 | 0.000463 | 0.895833 | 1.062978 | up |
| Com_8827_pos | Bifeprunox | C24 H23 N3 O2 | 385.17836 | 3.147581 | 1.654243 | 1.36E-05 | 0.951389 | 1.347094 | up |
| Com_887_pos | 3-Methyl-5-(5,5,8a-trimethyl-2-methylene-7-  oxodecahydro-1-naphthalenyl)pentyl acetate | C22 H36 O3 | 348.2654 | 12.20083 | 3.608907 | 9.50E-06 | 0.986111 | 2.259877 | up |
| Com_88_pos | (10Z)-1,1,1-Trifluoro-19-hydroxy-10-nonadecen-3-one | C19 H33 F3 O2 | 350.24216 | 2.31643 | 1.211903 | 0.046462 | 0.972222 | 1.642895 | up |
| Com_8911_pos | Fortimicin FU-10 | C12 H24 N2 O9 | 340.14901 | 6.461902 | 2.691959 | 0.00023 | 0.875 | 1.97723 | up |
| Com_9106_pos | Araloside A | C47 H74 O18 | 926.48578 | 3.044832 | 1.606363 | 3.59E-08 | 0.972222 | 1.325854 | up |
| Com_9246_pos | Boldione | C19 H24 O2 | 284.17699 | 4.307493 | 2.106848 | 0.000205 | 0.958333 | 1.277786 | up |
| Com_927_pos | (2E,4E,6E,8E,10E,12E,14E)-15-(4-Hydroxy-2,6,6  -trimethyl-1-cyclohexen-1-yl)-4,9,13-trimethyl-2,  4,6,8,10,12,14-pentadecaheptaenal | C27 H36 O2 | 392.27175 | 5.913171 | 2.563932 | 1.08E-05 | 0.958333 | 1.697975 | up |
| Com_96_pos | FARNESYL ACETONE | C18 H30 O | 262.22922 | 2.641304 | 1.401251 | 4.81E-05 | 0.909722 | 1.083371 | up |
| Com_9869_pos | Itopride | C20 H26 N2 O4 | 358.18764 | 3.422679 | 1.775126 | 1.01E-05 | 0.944444 | 1.299421 | up |
| Com_9962_pos | (3alpha,5beta,7alpha,8xi,9xi,12alpha,14xi,25R)-  7-(beta-D-Galactopyranosyloxy)-3,12-  dihydroxycholestan-27-yl acetate | C35 H60 O10 | 640.41557 | 3.09809 | 1.631379 | 0.000205 | 0.951389 | 1.601107 | up |
| Com_9985_pos | Onapristone | C29 H39 N O3 | 449.29085 | 3.21995 | 1.687038 | 0.001728 | 0.854167 | 1.135434 | up |
| Com_998_pos | Linolenic acid ethyl ester | C20 H34 O2 | 306.25532 | 4.706864 | 2.234766 | 0.000445 | 0.868056 | 1.337348 | up |
| Com_10009_pos | bisphenol A | C15 H16 O2 | 228.11459 | 0.198253 | -2.33458 | 0.000409 | 1 | 1.350793 | down |
| Com_10097_pos | Tyramine | C8 H11 N O | 137.08402 | 0.351633 | -1.50786 | 6.08E-08 | 1 | 1.114374 | down |
| Com_10195_pos | 3-(Methylsulfinyl)-L-alanine | C4 H9 N O3 S | 151.03026 | 0.347264 | -1.52589 | 4.49E-06 | 0.972222 | 1.162271 | down |
| Com_10300_pos | Cyclazocine | C18 H25 N O | 271.19326 | 0.177524 | -2.49392 | 4.93E-10 | 1 | 1.85089 | down |
| Com_10332_pos | (3beta,6beta,9xi,13xi)-15-(Dimethylamino)-  3-hydroxy-6,18:14,16-diepoxypimar-7-en-18-one | C22 H33 N O4 | 375.24269 | 0.255607 | -1.968 | 4.91E-06 | 0.986111 | 1.501685 | down |
| Com_1036_pos | 1,12-Dihydroxy-1,6,12,17-tetraazacyclodocosane-  2,5,13,16-tetrone | C18 H32 N4 O6 | 400.23203 | 0.285646 | -1.8077 | 7.39E-07 | 0.979167 | 1.328832 | down |
| Com_10462_pos | Ethyl levulinate | C7 H12 O3 | 144.07866 | 0.242962 | -2.04119 | 9.13E-10 | 1 | 1.467936 | down |
| Com_10468_pos | 1-Glyceryl stearate | C25 H46 O6 | 442.32947 | 0.309442 | -1.69226 | 0.000318 | 0.888889 | 1.094464 | down |
| Com_10469_pos | (2E)-2-{[(1E,4S)-4-Ammonio-4-carboxy-1-buten-   1. yl]imino}-5-[(1R)-1-(1H-indol-3-yl)ethyl]-4- 2. oxo-1,3-oxazolidin-5-ide | C18 H20 N4 O4 | 356.1501 | 0.23786 | -2.07182 | 5.05E-06 | 0.965278 | 1.540963 | down |
| Com_10677_pos | jesaconitine | C35 H49 N O12 | 675.32236 | 0.109267 | -3.19407 | 9.44E-06 | 0.972222 | 1.981908 | down |
| Com_10780_pos | Trimethoprim | C14 H18 N4 O3 | 290.13794 | 0.330616 | -1.59677 | 2.90E-05 | 0.9375 | 1.239125 | down |
| Com_1082_pos | N-Sulfanilyl-3,4-xylamide | C15 H16 N2 O3 S | 304.08762 | 0.148607 | -2.75043 | 2.55E-08 | 1 | 2.054424 | down |
| Com_10839_pos | (1R,3R,5R)-2-[(2S)-2-Amino-2-(3,5-dihydroxyadamantan  -1-yl)acetyl]-2-azabicyclo[3.1.0]hexane-3-carbonitrile | C18 H25 N3 O3 | 331.18911 | 0.235652 | -2.08527 | 8.07E-06 | 0.972222 | 1.574253 | down |
| Com_1088_pos | Hydantoin-5-propionic acid | C6 H8 N2 O4 | 172.04841 | 0.282145 | -1.82549 | 5.52E-06 | 0.986111 | 1.40434 | down |
| Com_10949_pos | Mesalazine | C7 H7 N O3 | 153.04249 | 0.11015 | -3.18245 | 5.64E-06 | 1 | 1.971653 | down |
| Com_11109_pos | Nefopam | C17 H19 N O | 253.14639 | 0.226754 | -2.1408 | 1.49E-12 | 1 | 1.570062 | down |
| Com_11135_pos | Ethopabate | C12 H15 N O4 | 237.0998 | 0.207643 | -2.26782 | 6.75E-09 | 1 | 1.609919 | down |
| Com_11164_pos | indolidan | C14 H15 N3 O2 | 257.11623 | 0.350497 | -1.51253 | 0.000947 | 0.861111 | 1.121081 | down |
| Com_11169_pos | PUROMYCIN | C22 H29 N7 O5 | 471.2223 | 0.397655 | -1.33041 | 0.000546 | 0.895833 | 1.049135 | down |
| Com_11210_pos | 4-[(3S,4R)-4-(4-Fluorophenyl)-3-hexanyl]phenol | C18 H21 F O | 272.15885 | 0.36025 | -1.47293 | 0.000275 | 0.895833 | 1.516816 | down |
| Com_1122_pos | Glycylglycylglycine | C6 H11 N3 O4 | 189.07494 | 0.274276 | -1.8663 | 2.89E-06 | 0.986111 | 1.420245 | down |
| Com_11251_pos | oxybutynin | C22 H31 N O3 | 357.22968 | 0.249391 | -2.00352 | 3.93E-08 | 1 | 1.478859 | down |
| Com_11322_pos | (+)-cassaine | C24 H39 N O4 | 405.28713 | 0.293556 | -1.76829 | 3.71E-10 | 1 | 1.272305 | down |
| Com_11324_pos | sedamine | C14 H21 N O | 219.16216 | 0.308259 | -1.69779 | 1.14E-05 | 0.930556 | 1.453952 | down |
| Com_11550_pos | N-benzoyl-D-arginine-4-nitroanilide | C19 H22 N6 O4 | 398.16854 | 0.365313 | -1.45279 | 0.000814 | 0.888889 | 1.179932 | down |
| Com_11575_pos | gamma-Glu-Gly | C7 H12 N2 O5 | 204.0745 | 0.312062 | -1.68009 | 6.72E-05 | 0.9375 | 1.233557 | down |
| Com_1172_pos | DL-Stachydrine | C7 H13 N O2 | 143.09462 | 0.319635 | -1.6455 | 2.27E-08 | 1 | 1.254411 | down |
| Com_11815_pos | Clavamycin F | C15 H24 N4 O7 | 372.16405 | 0.349705 | -1.51579 | 0.00107 | 0.895833 | 1.111083 | down |
| Com_11827_pos | Indecainide | C20 H24 N2 O | 308.18829 | 0.237434 | -2.0744 | 2.28E-08 | 1 | 1.531723 | down |
| Com_1187_pos | (3-Hydroxy-2-oxo-2,3-dihydro-1H-indol-3-yl)acetonitrile | C10 H8 N2 O2 | 188.05768 | 0.353484 | -1.50028 | 5.22E-05 | 0.944444 | 1.112909 | down |
| Com_1205_pos | CINPERENE | C25 H28 N2 O2 | 388.21453 | 0.10485 | -3.25361 | 9.41E-10 | 1 | 2.319425 | down |
| Com_12167_pos | HC Blue No. 1 | C11 H17 N3 O4 | 255.1216 | 0.377272 | -1.40632 | 0.00202 | 0.861111 | 1.017095 | down |
| Com_12171_pos | Hydrocortisone succinate | C25 H34 O8 | 462.22737 | 0.246427 | -2.02077 | 3.01E-07 | 0.986111 | 1.495456 | down |
| Com_12231_pos | N(6),N(6)-Dimethyladenine | C7 H9 N5 | 163.08603 | 0.323632 | -1.62758 | 4.92E-10 | 1 | 1.177637 | down |
| Com_12389_pos | HEPES | C8 H18 N2 O4 S | 238.09895 | 0.175208 | -2.51286 | 3.00E-05 | 0.993056 | 1.529774 | down |
| Com_12432_pos | Diphenylpyraline | C19 H23 N O | 281.17749 | 0.2486 | -2.0081 | 6.89E-09 | 1 | 1.478931 | down |
| Com_12516_pos | 3-[2-(Hydroxymethyl)-4-methoxyphenyl]-6-  methoxy-4-oxo-3,4-dihydro-1(2H)-quinazolinecarbaldehyde | C18 H18 N2 O5 | 342.12102 | 0.460272 | -1.11944 | 0.002657 | 0.847222 | 1.505713 | down |
| Com_12558_pos | 1-octadecanoyl-2-(7Z,10Z,13Z,16Z)-docosatetraenoyl  -sn-glycero-3-phosphoethanolamine | C45 H82 N O8 P | 795.57469 | 0.187664 | -2.41378 | 0.000134 | 0.993056 | 1.454301 | down |
| Com_12575_pos | Imidafenacin | C20 H21 N3 O | 319.16866 | 0.201687 | -2.30981 | 6.60E-05 | 0.923611 | 1.513831 | down |
| Com_12591_pos | 4-[(2R)-2-(Aminomethyl)-2-(hydroxymethyl)-5-oxo-1-  pyrrolidinyl]-3-(3-pentanylamino)benzoic acid | C18 H27 N3 O4 | 349.19851 | 0.215316 | -2.21547 | 8.11E-07 | 0.972222 | 1.648641 | down |
| Com_12642_pos | Pirfenidone | C12 H11 N O | 185.084 | 0.309381 | -1.69254 | 5.11E-06 | 0.958333 | 1.248356 | down |
| Com_12713_pos | Loperamide | C29 H33 Cl N2 O2 | 476.22225 | 0.268156 | -1.89886 | 8.39E-06 | 1 | 1.269103 | down |
| Com_1273_pos | Cadralazine | C12 H21 N5 O3 | 283.16398 | 0.193794 | -2.3674 | 5.02E-10 | 1 | 1.774669 | down |
| Com_12746_pos | Frovatriptan | C14 H17 N3 O | 243.13705 | 0.273756 | -1.86904 | 4.30E-06 | 0.958333 | 1.361092 | down |
| Com_1277_pos | Dicyclomine | C19 H35 N O2 | 309.26621 | 0.156375 | -2.67692 | 2.79E-06 | 0.979167 | 2.139407 | down |
| Com_12874_pos | Chromocarb | C10 H6 O4 | 190.02659 | 0.306838 | -1.70445 | 3.15E-05 | 1 | 1.41298 | down |
| Com_1288_pos | Propionylcarnitine | C10 H19 N O4 | 217.13135 | 0.278508 | -1.84421 | 2.42E-08 | 1 | 1.323227 | down |
| Com_12899_pos | 1. Isopropenyl-4,6,8-trimethoxy-9-methyl-2,3- 2. dihydrofuro[2,3-b]quinolin-9-ium | C18 H22 N O4 | 316.15302 | 0.20848 | -2.26202 | 2.80E-07 | 0.993056 | 1.669407 | down |
| Com_12968_pos | Tetroxoprim | C16 H22 N4 O4 | 334.1638 | 0.183853 | -2.44338 | 2.03E-07 | 1 | 1.812947 | down |
| Com_12976_pos | PGF2a ethanolamide | C22 H39 N O5 | 397.28203 | 0.24197 | -2.0471 | 3.63E-06 | 1 | 1.509643 | down |
| Com_13000_pos | Camobucol | C33 H50 O4 S2 | 574.31778 | 0.305399 | -1.71123 | 1.79E-08 | 1 | 1.270983 | down |
| Com_1304_pos | 9-Methoxy-2-(methylsulfanyl)-4,9-dihydro[1,3]thiazino[6,5-b]indole | C12 H12 N2 O S2 | 264.03878 | 0.018634 | -5.74588 | 1.71E-09 | 1 | 4.555247 | down |
| Com_13055_pos | phenacetin | C10 H13 N O2 | 179.09449 | 0.355018 | -1.49403 | 1.14E-08 | 1 | 1.057222 | down |
| Com_13088_pos | (6R,7S)-6,7-Dihydroxy-8-methyl-8-azabicyclo  [3.2.1]oct-3-yl (2E)-2-methyl-2-butenoate | C13 H21 N O4 | 255.14669 | 0.235699 | -2.08498 | 1.22E-06 | 1 | 1.391068 | down |
| Com_13204_pos | Mitragynine | C23 H30 N2 O4 | 398.22 | 0.270067 | -1.88861 | 3.84E-07 | 0.972222 | 1.47216 | down |
| Com_1321_pos | Omeprazole | C17 H19 N3 O3 S | 345.11413 | 0.071755 | -3.80079 | 4.63E-11 | 1 | 2.760781 | down |
| Com_13256_pos | Kyotorphin | C15 H23 N5 O4 | 337.17442 | 0.255184 | -1.97039 | 3.98E-08 | 1 | 1.48371 | down |
| Com_13278_pos | Eterobarb | C16 H20 N2 O5 | 320.13667 | 0.221405 | -2.17524 | 1.73E-05 | 0.9375 | 1.610172 | down |
| Com_13370_pos | Geranyl formate | C11 H18 O2 | 182.13062 | 0.324989 | -1.62154 | 0.000124 | 0.965278 | 1.091856 | down |
| Com_1340_pos | N-Arachidonoyl-L-serine | C23 H37 N O4 | 391.27168 | 0.471839 | -1.08363 | 0.002104 | 0.861111 | 1.098027 | down |
| Com_13447_pos | Dolichotheline | C10 H17 N3 O | 195.13719 | 0.289031 | -1.7907 | 3.00E-06 | 0.972222 | 1.317125 | down |
| Com_1344_pos | Fingolimod | C19 H33 N O2 | 307.25061 | 0.087968 | -3.50688 | 4.42E-10 | 1 | 2.573357 | down |
| Com_13457_pos | 3,8,13,17-tetramethyl-12-vinyl-2,7,18-Porphinetripropionic acid | C35 H36 N4 O6 | 608.26402 | 0.346027 | -1.53105 | 0.001239 | 0.875 | 1.34178 | down |
| Com_13470_pos | ajugarin I | C24 H34 O7 | 434.22891 | 0.325325 | -1.62005 | 3.23E-05 | 0.972222 | 1.062624 | down |
| Com_13474_pos | Orphenadrine | C18 H23 N O | 269.1776 | 0.298721 | -1.74313 | 1.27E-08 | 1 | 1.237977 | down |
| Com_1351_pos | Testosterone isocaproate | C25 H38 O3 | 386.28133 | 0.382245 | -1.38743 | 0.000459 | 0.888889 | 1.157293 | down |
| Com_1352_pos | Actinoquinol | C11 H11 N O4 S | 253.0406 | 0.255663 | -1.96769 | 1.44E-07 | 0.993056 | 1.462471 | down |
| Com_13541_pos | Amlodipine | C20 H25 Cl N2 O5 | 408.14391 | 0.338969 | -1.56077 | 2.28E-06 | 1 | 1.192122 | down |
| Com_13548_pos | (-)-Physostigmine | C15 H21 N3 O2 | 275.16298 | 0.146452 | -2.7715 | 8.76E-08 | 1 | 1.989413 | down |
| Com_13570_pos | Tavaborole | C7 H6 B F O2 | 152.04374 | 0.097845 | -3.35336 | 3.61E-09 | 1 | 2.351616 | down |
| Com_13637_pos | piketoprofen | C22 H20 N2 O2 | 344.15123 | 0.21258 | -2.23392 | 2.39E-07 | 0.986111 | 1.628036 | down |
| Com_13678_pos | 4-{[(1R,2R)-2-{[4-(1,2-Benzothiazol-3-yl)-1-  piperazinyl]methyl}cyclohexyl]methyl}-10-  hydroxy-4-azatricyclo[5.2.1.0~2,6~]decane-3,5-dione | C28 H36 N4 O3 S | 508.25196 | 0.305743 | -1.70961 | 0.000273 | 0.923611 | 1.091936 | down |
| Com_13734_pos | osalmid | C13 H11 N O3 | 229.07365 | 0.340659 | -1.5536 | 0.000165 | 0.902778 | 1.461602 | down |
| Com_13798_pos | 2E-Crotamiton | C13 H17 N O | 203.13096 | 0.17266 | -2.53399 | 2.13E-07 | 1 | 1.711015 | down |
| Com_13849_pos | 2172 | C23 H29 N5 O | 391.23888 | 0.184667 | -2.437 | 5.14E-10 | 1 | 1.787755 | down |
| Com_13951_pos | (3-Amino-3-carboxypropyl){[5-(6-amino-9H-purin-  9-yl)-3,4-dihydroxytetrahydro-2-furanyl]methyl}  methylsulfonium | C15 H23 N6 O5 S | 399.1458 | 0.351751 | -1.50737 | 0.000269 | 0.930556 | 1.1019 | down |
| Com_13989_pos | Imafen | C11 H13 N3 | 187.11087 | 0.110427 | -3.17884 | 1.42E-08 | 1 | 2.164313 | down |
| Com_1412_pos | 3-[(6-Oxodecanoyl)oxy]-4-(trimethylammonio)butanoate | C17 H31 N O5 | 329.21971 | 0.322683 | -1.63181 | 3.86E-06 | 0.993056 | 1.25794 | down |
| Com_14162_pos | Pirenzepine | C19 H21 N5 O2 | 351.16763 | 0.110129 | -3.18273 | 7.29E-07 | 0.979167 | 2.104173 | down |
| Com_14205_pos | Bevenopran | C20 H26 N4 O4 | 386.19527 | 0.247436 | -2.01487 | 6.60E-06 | 0.958333 | 1.541804 | down |
| Com_1420_pos | (4S)-4-[(2E,4Z)-2,4-Decadienoyloxy]-4-(trimethy  lammonio)butanoate | C17 H29 N O4 | 311.20912 | 0.320828 | -1.64013 | 4.74E-06 | 0.993056 | 1.26759 | down |
| Com_14346_pos | libenzapril | C18 H25 N3 O5 | 363.17791 | 0.250234 | -1.99865 | 0.000446 | 0.916667 | 1.247196 | down |
| Com_14371_pos | amiloxate | C15 H20 O3 | 248.1408 | 0.435563 | -1.19905 | 0.000348 | 0.909722 | 1.048096 | down |
| Com_14381_pos | N-Acetyl-L-phenylalanine | C11 H13 N O3 | 207.08939 | 0.369056 | -1.43809 | 3.57E-10 | 1 | 1.052718 | down |
| Com_1441_pos | Eugenol | C10 H12 O2 | 164.08371 | 0.256628 | -1.96225 | 0.000357 | 0.902778 | 1.510093 | down |
| Com_14444_pos | butaperazine | C24 H31 N3 O S | 409.2207 | 0.294737 | -1.7625 | 1.09E-05 | 0.958333 | 1.257321 | down |
| Com_14483_pos | 5-Methoxytryptamine | C11 H14 N2 O | 190.11052 | 0.352782 | -1.50315 | 0.000538 | 0.868056 | 1.071394 | down |
| Com_14537_pos | Furmecyclox | C14 H21 N O3 | 251.1518 | 0.384002 | -1.38081 | 5.04E-06 | 0.958333 | 1.049505 | down |
| Com_14555_pos | Alpinine | C23 H29 N O6 | 415.19982 | 0.428396 | -1.22298 | 0.000529 | 0.909722 | 1.07815 | down |
| Com_14557_pos | Sirolimus | C51 H79 N O13 | 913.55965 | 0.22186 | -2.17228 | 9.77E-07 | 0.986111 | 1.842617 | down |
| Com_14560_pos | Tebufenpyrad | C18 H24 Cl N3 O | 333.161 | 0.239979 | -2.05902 | 3.80E-07 | 0.986111 | 1.545436 | down |
| Com_14587_pos | MPTP N-OXIDE | C12 H15 N O | 189.11516 | 0.241059 | -2.05254 | 2.34E-08 | 1 | 1.466716 | down |
| Com_14601_pos | PD123319 | C31 H32 N4 O3 | 508.24886 | 0.214101 | -2.22364 | 7.13E-05 | 0.958333 | 1.390093 | down |
| Com_14608_pos | Noramidopyrine | C12 H15 N3 O | 217.12142 | 0.257702 | -1.95622 | 5.21E-06 | 0.958333 | 1.394824 | down |
| Com_14625_pos | triamcinolone hexacetonide | C30 H41 F O7 | 532.28624 | 0.313973 | -1.67129 | 0.002019 | 0.916667 | 1.083361 | down |
| Com_14731_pos | Zicronapine | C22 H27 Cl N2 | 354.18529 | 0.203096 | -2.29977 | 1.05E-06 | 0.972222 | 1.724296 | down |
| Com_14833_pos | 6-(2-Amino-2-carboxyethyl)-7,8-dioxo-1,2,3,4,7,8-hexahydro  -2,4-quinolinedicarboxylic acid | C14 H14 N2 O8 | 338.07517 | 0.209442 | -2.25538 | 4.21E-10 | 1 | 1.601957 | down |
| Com_1489_pos | n-Ribosylhistidine | C11 H17 N3 O6 | 287.11127 | 0.266262 | -1.90908 | 2.12E-05 | 0.9375 | 1.437342 | down |
| Com_14904_pos | primidone | C12 H14 N2 O2 | 218.10556 | 0.136425 | -2.87382 | 0.021514 | 0.881944 | 1.377969 | down |
| Com_15025_pos | Dimepiperate | C15 H21 N O S | 263.13411 | 0.229189 | -2.12539 | 6.92E-08 | 0.993056 | 1.52285 | down |
| Com_1510_pos | 19-Nortestosterone | C18 H26 O2 | 274.19276 | 0.352434 | -1.50458 | 8.24E-05 | 0.9375 | 1.098517 | down |
| Com_15129_pos | Silafluofen | C25 H29 F O2 Si | 408.19164 | 0.161539 | -2.63005 | 9.20E-08 | 0.993056 | 1.880577 | down |
| Com_15145_pos | Artemotil | C17 H28 O5 | 312.1946 | 0.141504 | -2.82109 | 5.96E-14 | 1 | 2.088766 | down |
| Com_15171_pos | Propafenone | C21 H27 N O3 | 341.19868 | 0.252659 | -1.98474 | 3.71E-06 | 0.972222 | 1.609756 | down |
| Com_1519_pos | Fluoxetine | C17 H18 F3 N O | 309.13201 | 0.38039 | -1.39445 | 4.77E-06 | 0.944444 | 1.036584 | down |
| Com_1526_pos | oxamniquine | C14 H21 N3 O3 | 279.15795 | 0.288205 | -1.79483 | 0.000266 | 0.909722 | 1.326349 | down |
| Com_15317_pos | 4-Acetamido-2-amino-6-nitrotoluene | C9 H11 N3 O3 | 209.07993 | 0.270466 | -1.88648 | 2.22E-05 | 0.958333 | 1.353516 | down |
| Com_15339_pos | trans-2-Dodecenoylcarnitine | C19 H35 N O4 | 341.25594 | 0.38204 | -1.38821 | 1.71E-08 | 1 | 1.012403 | down |
| Com_15386_pos | Epristeride | C25 H37 N O3 | 399.27677 | 0.198703 | -2.33131 | 2.16E-05 | 0.930556 | 1.637271 | down |
| Com_1543_pos | 5-[8,10-Dihydroxy-11-(hydroxymethyl)-4,7-dimethyltridecyl]  -6-ethyl-4-hydroxy-4,5-dimethyl-2-cyclohexen-1-one | C26 H48 O5 | 440.3509 | 0.222887 | -2.16562 | 2.10E-06 | 0.9375 | 1.57191 | down |
| Com_1544_pos | bis(3-aminopropyl)amine | C6 H17 N3 | 131.14221 | 0.117404 | -3.09045 | 3.62E-08 | 1 | 2.271611 | down |
| Com_15506_pos | Embelin | C17 H26 O4 | 294.18229 | 0.353223 | -1.50135 | 7.89E-09 | 1 | 1.090956 | down |
| Com_15543_pos | Ankorine | C19 H29 N O4 | 335.20914 | 0.237837 | -2.07196 | 3.14E-09 | 1 | 1.568235 | down |
| Com_1554_pos | N-Acetylanthranilic acid | C9 H9 N O3 | 179.05817 | 0.356218 | -1.48917 | 3.28E-07 | 1 | 1.12869 | down |
| Com_15582_pos | Tryptophol | C10 H11 N O | 161.08416 | 0.250845 | -1.99513 | 1.80E-07 | 1 | 1.396905 | down |
| Com_15644_pos | NALMEFENE | C21 H25 N O3 | 339.18416 | 0.297492 | -1.74908 | 0.000368 | 0.944444 | 1.193874 | down |
| Com_1565_pos | Indole-3-carbidol | C9 H9 N O | 147.0682 | 0.241372 | -2.05067 | 9.57E-11 | 1 | 1.505988 | down |
| Com_15682_pos | nicotine imine | C10 H13 N2 | 161.10688 | 0.146048 | -2.77548 | 0.000912 | 0.951389 | 1.42537 | down |
| Com_15725_pos | CILAZAPRILAT | C20 H27 N3 O5 | 389.19468 | 0.19136 | -2.38564 | 1.29E-08 | 1 | 1.655774 | down |
| Com_1573_pos | (4S)-4-[(11-Carboxyundecanoyl)oxy]-4-  (trimethylammonio)butanoate | C19 H35 N O6 | 373.24589 | 0.369838 | -1.43503 | 6.90E-05 | 0.916667 | 1.146894 | down |
| Com_15749_pos | Alprenolol | C15 H23 N O2 | 249.17253 | 0.25075 | -1.99568 | 8.06E-09 | 1 | 1.496319 | down |
| Com_15770_pos | Sequifenadine | C22 H27 N O | 321.20872 | 0.17345 | -2.52741 | 1.93E-09 | 1 | 1.918723 | down |
| Com_15792_pos | 3-Methylcyclohexanethiol | C7 H14 S | 130.081 | 0.263847 | -1.92223 | 2.38E-10 | 1 | 1.420563 | down |
| Com_15799_pos | pentobarbital | C11 H18 N2 O3 | 226.13144 | 0.305663 | -1.70998 | 1.14E-09 | 1 | 1.258516 | down |
| Com_15863_pos | diaziquone | C16 H20 N4 O6 | 364.13771 | 0.2375 | -2.074 | 2.31E-06 | 0.979167 | 1.503631 | down |
| Com_15868_pos | buphenine | C19 H25 N O2 | 299.18972 | 0.383202 | -1.38382 | 2.45E-05 | 0.944444 | 1.174948 | down |
| Com_1588_pos | THREO-SPHINGOSINE, (-)- | C18 H37 N O2 | 299.28195 | 0.165407 | -2.59591 | 6.13E-07 | 1 | 2.097552 | down |
| Com_16000_pos | Imiprothrin | C17 H22 N2 O4 | 318.15775 | 0.274808 | -1.86351 | 5.47E-10 | 1 | 1.365373 | down |
| Com_16004_pos | Sweroside | C16 H22 O9 | 358.12713 | 0.290601 | -1.78289 | 3.01E-06 | 0.965278 | 1.275617 | down |
| Com_16070_pos | prilocaine | C13 H20 N2 O | 220.15738 | 0.225264 | -2.15031 | 3.40E-11 | 1 | 1.589405 | down |
| Com_16108_pos | Heptaethylene Glycol | C14 H30 O8 | 326.1946 | 0.258757 | -1.95033 | 2.16E-06 | 0.972222 | 1.379782 | down |
| Com_16151_pos | Fusarin C | C23 H29 N O7 | 431.1935 | 0.211525 | -2.2411 | 2.86E-06 | 0.979167 | 1.498935 | down |
| Com_16175_pos | Carvedilol | C24 H26 N2 O4 | 406.18816 | 0.369977 | -1.43449 | 1.92E-07 | 0.972222 | 1.061582 | down |
| Com_16234_pos | Tetracaine | C15 H24 N2 O2 | 264.18318 | 0.270568 | -1.88594 | 2.19E-07 | 1 | 1.369212 | down |
| Com_1623_pos | Tetrahydrodeoxycorticosterone | C21 H34 O3 | 334.24928 | 0.149138 | -2.74528 | 4.24E-05 | 0.951389 | 1.816624 | down |
| Com_16347_pos | Isopropyl methoxy pyrazine | C8 H12 N2 O | 152.09426 | 0.296918 | -1.75186 | 3.04E-06 | 0.958333 | 1.22958 | down |
| Com_16360_pos | 17,21-Epoxy-9-fluoro-11beta-hydroxyprogesterone | C21 H27 F O4 | 362.18771 | 0.320823 | -1.64015 | 1.27E-05 | 0.972222 | 1.209923 | down |
| Com_16363_pos | TOLMETIN | C15 H15 N O3 | 257.10473 | 0.33977 | -1.55737 | 1.03E-06 | 0.986111 | 1.189352 | down |
| Com_16389_pos | Cucurbitacin E | C32 H44 O8 | 556.30333 | 0.094061 | -3.41026 | 0.000659 | 0.958333 | 1.542932 | down |
| Com_16470_pos | (2R)-2-Hydroxy-3-(phosphonooxy)propyl (11Z)-11-icosenoate | C23 H45 O7 P | 464.2882 | 0.297532 | -1.74888 | 2.61E-06 | 0.951389 | 1.291309 | down |
| Com_1651_pos | (8E)-2-Amino-8-octadecene-1,3,4-triol | C18 H37 N O3 | 315.27672 | 0.112951 | -3.14623 | 8.64E-08 | 0.972222 | 2.410248 | down |
| Com_16562_pos | METHYL 2-(4-ISOPROPYL-4-METHYL-5-  OXO-2-IMIDAZOLIN-2-YL)-P- TOLUATE | C16 H20 N2 O3 | 288.14683 | 0.329901 | -1.5999 | 4.80E-06 | 0.986111 | 1.114157 | down |
| Com_16600_pos | (2R,3S)-3-Hydroxy-8-methyl-8-azabicyclo  [3.2.1]octane-2-carboxylic acid | C9 H15 N O3 | 185.10503 | 0.266609 | -1.9072 | 8.05E-06 | 0.979167 | 1.291096 | down |
| Com_16620_pos | 5-Methoxybenzimidazole | C8 H8 N2 O | 148.06351 | 0.187206 | -2.4173 | 1.26E-07 | 1 | 1.637161 | down |
| Com_16713_pos | 1-(2,6-Dihydroxy-4-methoxy-3,5-dimethylphenyl)  -3-phenyl-1-propanone | C18 H20 O4 | 300.13645 | 0.328363 | -1.60664 | 3.93E-05 | 0.986111 | 1.336362 | down |
| Com_16812_pos | cyclopeptine | C17 H16 N2 O2 | 280.12071 | 0.459574 | -1.12163 | 0.005266 | 0.8125 | 1.002876 | down |
| Com_16828_pos | Fursultiamine | C17 H26 N4 O3 S2 | 398.1434 | 0.337059 | -1.56893 | 0.003789 | 0.819444 | 1.094198 | down |
| Com_16881_pos | simeconazole | C14 H20 F N3 O Si | 293.1371 | 0.141376 | -2.82239 | 0.000921 | 0.909722 | 1.450748 | down |
| Com_16886_pos | 7a-Hydroxy-o-carbamoyl-deacetylcephalosporin C | C15 H20 N4 O9 S | 432.09577 | 0.294281 | -1.76473 | 0.001176 | 0.861111 | 1.216306 | down |
| Com_16961_pos | 4-(FLUOROPHENYL)-1-CYCLOPROPYLMETHYL-  5-(2-AMINO-4-PYRIMIDINYL)IMIDAZOLE | C17 H16 F N5 | 309.14013 | 0.128715 | -2.95775 | 0.009528 | 0.868056 | 1.468114 | down |
| Com_16976_pos | 3386 | C25 H30 F N O6 | 459.20525 | 0.466818 | -1.09907 | 0.002423 | 0.840278 | 1.024617 | down |
| Com_16995_pos | (3S,3aR,5S,5aS,7aS,9R,11aR,11bS,12aR)-3a-Hydroxy-  3-(hydroxymethyl)-2,5,9,11b-tetramethyl-3,3a,5b,7a,  8,9,10,11,11a,11b-decahydro-5H-benzo[4',5']indeno  [2',1':3,4]furo[2,3-c]pyrrole-1,12(2H,5aH)-dione | C22 H31 N O5 | 389.22035 | 0.341691 | -1.54924 | 0.000119 | 0.902778 | 1.293886 | down |
| Com_1700_pos | Dihomo-gamma-linolenic acid | C20 H34 O2 | 306.25531 | 0.266505 | -1.90777 | 0.000299 | 0.888889 | 1.53062 | down |
| Com_17014_pos | Reproterol | C18 H23 N5 O5 | 389.1682 | 0.327407 | -1.61084 | 0.002507 | 0.847222 | 1.069882 | down |
| Com_17052_pos | Difeterol | C25 H29 N O2 | 375.21924 | 0.309255 | -1.69313 | 1.34E-06 | 0.979167 | 1.357068 | down |
| Com_17127_pos | 4-METHYL MEIQX | C12 H13 N5 | 227.11627 | 0.330025 | -1.59935 | 1.10E-05 | 0.958333 | 1.114722 | down |
| Com_17141_pos | Levallorphan | C19 H25 N O | 283.19324 | 0.26813 | -1.899 | 4.82E-08 | 1 | 1.408933 | down |
| Com_17176_pos | saxagliptin | C18 H25 N3 O2 | 315.19411 | 0.168146 | -2.57222 | 2.65E-06 | 0.986111 | 1.711875 | down |
| Com_17223_pos | Alanyltryptophan | C14 H17 N3 O3 | 275.12687 | 0.37551 | -1.41308 | 0.000337 | 0.902778 | 1.067537 | down |
| Com_1729_pos | HC BLUE NO. 2 | C12 H19 N3 O5 | 285.13205 | 0.20121 | -2.31322 | 2.01E-05 | 0.944444 | 1.786223 | down |
| Com_17335_pos | (1S,4aS)-1,4a-Dimethyl-1,2,3,4,4a,5,6,8a-  octahydronaphthalene | C12 H20 | 164.15654 | 0.33751 | -1.567 | 1.21E-05 | 1 | 1.080662 | down |
| Com_17362_pos | EPTAZOCINE | C15 H21 N O | 231.16205 | 0.258266 | -1.95307 | 2.70E-08 | 1 | 1.446536 | down |
| Com_17476_pos | 3,7-Dimethyl-1,6-octadien-3-yl 2-aminobenzoate | C17 H23 N O2 | 273.17247 | 0.227307 | -2.13728 | 4.56E-05 | 0.979167 | 1.352114 | down |
| Com_17488_pos | 3113 | C17 H26 N2 O4 S | 354.16068 | 0.191919 | -2.38143 | 1.47E-08 | 1 | 1.741922 | down |
| Com_17539_pos | trifloxystrobin | C20 H19 F3 N2 O4 | 408.13166 | 0.192648 | -2.37596 | 2.36E-12 | 1 | 1.749513 | down |
| Com_17553_pos | lincomycin | C18 H34 N2 O6 S | 406.21366 | 0.319353 | -1.64678 | 2.61E-08 | 1 | 1.196601 | down |
| Com_17588_pos | Norgestrienone | C20 H22 O2 | 294.16278 | 0.231167 | -2.11299 | 7.34E-08 | 0.986111 | 1.475127 | down |
| Com_17656_pos | Olopatadine | C21 H23 N O3 | 337.16893 | 0.379331 | -1.39847 | 3.28E-05 | 0.944444 | 1.138656 | down |
| Com_17796_pos | Fananserin | C23 H24 F N3 O2 S | 425.1584 | 0.142018 | -2.81586 | 6.66E-13 | 1 | 2.071027 | down |
| Com_17842_pos | Loganin | C17 H26 O10 | 390.15358 | 0.278304 | -1.84527 | 0.000259 | 0.909722 | 1.286162 | down |
| Com_17848_pos | Androstenedione | C19 H26 O2 | 286.19417 | 0.326986 | -1.6127 | 7.06E-06 | 0.965278 | 1.170979 | down |
| Com_17855_pos | pratosartan | C25 H26 N6 O | 426.21792 | 0.215593 | -2.21362 | 8.75E-13 | 1 | 1.603242 | down |
| Com_1796_pos | Decanoylcarnitine | C17 H33 N O4 | 315.24036 | 0.347433 | -1.52519 | 3.65E-07 | 0.993056 | 1.112759 | down |
| Com_17986_pos | 2,3-dinor-8-epi-prostaglandin F2alpha | C18 H30 O5 | 326.20999 | 0.246755 | -2.01885 | 2.39E-07 | 0.986111 | 1.465789 | down |
| Com_18009_pos | 7-Isopropyl-4a-methyl-1-methylene-  1,2,3,4,4a,9,10,10a-octahydrophenanthrene | C19 H26 | 254.20297 | 0.211223 | -2.24316 | 1.94E-07 | 1 | 1.529264 | down |
| Com_18013_pos | Dodecanedioic acid | C12 H22 O4 | 230.15281 | 0.313113 | -1.67525 | 2.36E-06 | 0.944444 | 1.284841 | down |
| Com_18086_pos | Nigakilactone E | C24 H34 O8 | 450.2239 | 0.352551 | -1.5041 | 1.26E-05 | 0.930556 | 1.121798 | down |
| Com_1809_pos | L-(+)-Leucine | C6 H13 N O2 | 131.09461 | 0.2683 | -1.89808 | 1.30E-09 | 1 | 1.435528 | down |
| Com_18136_pos | N-Acetylleucylleucyltyrosine | C23 H35 N3 O6 | 449.25245 | 0.359276 | -1.47683 | 4.05E-06 | 0.958333 | 1.077888 | down |
| Com_18226_pos | (2R,3R)-N-{4-[(Diaminomethylene)amino]butyl}-  5-[(1E)-3-({4-[(diaminomethylene)amino]butyl}amino)  -3-oxo-1-propen-1-yl]-2-(4-hydroxyphenyl)-2,3-  dihydro-1-benzofuran-3-carboxamide | C28 H38 N8 O4 | 550.30106 | 0.258349 | -1.9526 | 4.14E-07 | 0.986111 | 1.455692 | down |
| Com_18275_pos | 1. Methoxy-17-methyl-6,7,8,14-tetradehydro-4,5- 2. epoxymorphinan-3-ol | C18 H19 N O3 | 297.13759 | 0.275842 | -1.85808 | 0.000223 | 0.881944 | 1.418787 | down |
| Com_1827_pos | Dimethoxymethyl-benzene | C9 H12 O2 | 152.08364 | 0.393924 | -1.34401 | 5.92E-06 | 1 | 1.073226 | down |
| Com_1828_pos | Ethyl violet | C31 H41 N3 | 455.32434 | 0.242386 | -2.04462 | 2.14E-08 | 1 | 1.534934 | down |
| Com_18291_pos | demethoxycurcumin | C20 H18 O5 | 338.11386 | 0.272405 | -1.87618 | 1.11E-06 | 0.986111 | 1.394363 | down |
| Com_18354_pos | Cinchonan-9-ol | C19 H22 N2 O | 294.17274 | 0.344181 | -1.53876 | 3.89E-06 | 0.993056 | 1.16605 | down |
| Com_18392_pos | asn-tyr | C13 H17 N3 O5 | 295.11642 | 0.192853 | -2.37443 | 0.004203 | 0.979167 | 1.140099 | down |
| Com_1843_pos | Valproic acid | C8 H16 O2 | 144.11475 | 0.21007 | -2.25106 | 5.85E-10 | 1 | 1.669357 | down |
| Com_18561_pos | ENADENINE | C10 H13 N5 | 203.11764 | 0.276506 | -1.85462 | 1.65E-07 | 1 | 1.410158 | down |
| Com_18691_pos | Glutathionylaminopropylcadaverine | C18 H36 N6 O5 S | 448.24704 | 0.322717 | -1.63166 | 1.20E-08 | 0.993056 | 1.202898 | down |
| Com_18726_pos | 4-Amino-1-{(2xi)-5-O-[hydroxy({hydroxy[(2R)-2-  [(9Z)-9-octadecenoyloxy]-3-(stearoyloxy)propoxy]  phosphoryl}oxy)phosphoryl]-beta-D-threo-  pentofuranosyl}-2(1H)-pyrimidinone | C48 H87 N3 O15 P2 | 1007.56437 | 0.323701 | -1.62727 | 9.76E-05 | 0.986111 | 1.02336 | down |
| Com_18766_pos | 2H-Pyrazino(1',2':1,5)pyrrolo(2,3-b)indole-  1,4(3H,5aH)-dione, 10b-(1,1-dimethyl-2-propenyl)-  6,10b,11,11a-tetrahydro-3-(1H-imidazol-4-ylmethylene)- | C22 H23 N5 O2 | 389.18648 | 0.375503 | -1.4131 | 1.40E-05 | 0.944444 | 1.142427 | down |
| Com_18776_pos | ALLN | C20 H37 N3 O4 | 383.27825 | 0.295539 | -1.75858 | 4.37E-06 | 0.986111 | 1.359669 | down |
| Com_18787_pos | Balfourodinine | C17 H22 N O4 | 304.15325 | 0.228708 | -2.12842 | 6.74E-07 | 1 | 1.421198 | down |
| Com_19059_pos | Tenofovir alafenamide | C21 H29 N6 O5 P | 476.19412 | 0.329305 | -1.6025 | 0.000764 | 0.881944 | 1.06384 | down |
| Com_19174_pos | 3,4,5-Trihydroxy-6-oxo-1-(3,5,7-trihydroxy-3,4  -dihydro-2H-chromen-2-yl)-6H-benzo[7]  annulene-8-carboxylic acid | C21 H16 O10 | 428.07488 | 0.237112 | -2.07636 | 2.80E-07 | 0.986111 | 1.464103 | down |
| Com_1917_pos | Hexadecanedioic acid mono-L-carnitine ester | C23 H43 N O6 | 429.30855 | 0.203102 | -2.29973 | 2.39E-07 | 0.986111 | 1.734693 | down |
| Com_1919_pos | 1-O-[(3alpha,5beta,7alpha,12beta)-3,7,12-  Trihydroxy-24-oxocholan-24-yl]-beta-D-galactopyranose | C30 H50 O10 | 570.34018 | 0.15353 | -2.70341 | 0.000145 | 0.909722 | 2.220571 | down |
| Com_19287_pos | turofexorate isopropyl | C25 H24 F2 N2 O3 | 438.17505 | 0.377633 | -1.40494 | 3.67E-06 | 0.958333 | 1.009373 | down |
| Com_19305_pos | Oseltamivir | C16 H28 N2 O4 | 312.20452 | 0.281858 | -1.82696 | 0.000192 | 0.895833 | 1.271807 | down |
| Com_19314_pos | 2-Isobutoxynaphthalene | C14 H16 O | 200.11988 | 0.298015 | -1.74654 | 2.55E-09 | 1 | 1.28235 | down |
| Com_19319_pos | Naltrexone | C20 H23 N O4 | 341.16357 | 0.175338 | -2.51179 | 2.55E-09 | 1 | 1.779878 | down |
| Com_19473_pos | Nemonapride (JAN) | C21 H26 Cl N3 O2 | 387.17092 | 0.382834 | -1.38521 | 0.000142 | 0.923611 | 1.112074 | down |
| Com_19614_pos | FLUTRIMAZOLE | C22 H16 F2 N2 | 346.12718 | 0.236152 | -2.08221 | 1.07E-10 | 1 | 1.531921 | down |
| Com_19679_pos | (4S)-4-{[2-O-(beta-L-Arabinofuranosyl)-beta-  L-arabinofuranosyl]oxy}proline | C15 H25 N O11 | 395.1425 | 0.247729 | -2.01317 | 2.08E-07 | 0.979167 | 1.459332 | down |
| Com_19731_pos | N-{(2S,3R,4E)-3-Hydroxy-1-[(3-O-sulfo-beta-  D-threo-hexopyranosyl)oxy]-4-octadecen-2-yl}tetradecanamide | C38 H73 N O11 S | 751.48819 | 0.269916 | -1.88942 | 7.09E-06 | 0.972222 | 1.27552 | down |
| Com_19849_pos | (3S,8aS)-3-(4-Hydroxybenzyl)hexahydropyrrolo  [1,2-a]pyrazine-1,4-dione | C14 H16 N2 O3 | 260.11635 | 0.366289 | -1.44895 | 1.77E-06 | 0.979167 | 1.118998 | down |
| Com_19857_pos | (1S,2E,16Z,18E,20S,21S,22R,23R,24R,25S,27R,28R,29R)-  6,8,21,23,27,28-Hexahydroxy-3,7,16,20,22,24,29-heptamethyl-  26-oxa-14-azatetracyclo[23.2.2.1~9,13~.0~5,10~]  triaconta-2,5,7,9,12,16,18-heptaene-4,11,15,30-tetrone | C35 H43 N O11 | 653.28586 | 0.32163 | -1.63653 | 5.00E-05 | 0.958333 | 1.103826 | down |
| Com_19897_pos | 3,3',3'',3''',3''''-[8,13-Bis(carboxymethyl)-18-methyl-  2,3,7,12,17-porphyrinpentayl]pentapropanoic acid | C40 H40 N4 O14 | 800.25017 | 0.230208 | -2.11899 | 1.07E-11 | 1 | 1.53376 | down |
| Com_1989_pos | Debromohymenialdisine | C11 H11 N5 O2 | 245.08964 | 0.156122 | -2.67925 | 8.60E-07 | 0.972222 | 2.134376 | down |
| Com_19905_pos | Leonurine | C14 H21 N3 O5 | 311.14771 | 0.332592 | -1.58817 | 0.000119 | 0.944444 | 1.017996 | down |
| Com_2002_pos | Oleamide | C18 H35 N O | 281.27133 | 0.166707 | -2.58461 | 3.61E-07 | 1 | 2.056501 | down |
| Com_20039_pos | 3,8,15-Trihydroxy-12,13-epoxytrichothec-9-en-4-yl acetate | C17 H24 O7 | 340.15264 | 0.198639 | -2.33178 | 1.52E-08 | 1 | 1.686282 | down |
| Com_20066_pos | Echimidine | C20 H31 N O7 | 397.21001 | 0.230173 | -2.11921 | 4.93E-09 | 1 | 1.539298 | down |
| Com_20214_pos | Galunisertib | C22 H19 N5 O | 369.16039 | 0.400922 | -1.31861 | 0.00036 | 0.895833 | 1.136412 | down |
| Com_20341_pos | guaiapate | C18 H29 N O4 | 323.20812 | 0.272241 | -1.87704 | 4.49E-05 | 0.972222 | 1.203363 | down |
| Com_2041_pos | 3-(indol-3-yl)-2-oxobutyric acid | C12 H11 N O3 | 217.07363 | 0.247197 | -2.01627 | 1.55E-07 | 1 | 1.505516 | down |
| Com_20504_pos | tipepidine | C15 H17 N S2 | 275.08106 | 0.250032 | -1.99981 | 0.001827 | 0.916667 | 1.175951 | down |
| Com_20707_pos | Bitertanol | C20 H23 N3 O2 | 337.17859 | 0.293698 | -1.76759 | 1.56E-05 | 0.986111 | 1.151822 | down |
| Com_20841_pos | Motesanib | C22 H23 N5 O | 373.18849 | 0.269272 | -1.89286 | 3.47E-08 | 1 | 1.334941 | down |
| Com_21218_pos | Fluvoxamine Acid | C14 H17 F3 N2 O3 | 318.11843 | 0.289747 | -1.78714 | 2.51E-05 | 0.944444 | 1.428827 | down |
| Com_2141_pos | 4-Aminopyridine | C5 H6 N2 | 94.05311 | 0.20305 | -2.30009 | 0.000375 | 0.888889 | 1.481287 | down |
| Com_2154_pos | 6-Acetamido-2-oxohexanoic acid | C8 H13 N O4 | 187.08444 | 0.292636 | -1.77282 | 0.00015 | 0.909722 | 1.455275 | down |
| Com_21557_pos | Trazodone | C19 H22 Cl N5 O | 371.15111 | 0.367337 | -1.44482 | 4.78E-05 | 0.944444 | 1.006434 | down |
| Com_21561_pos | Parbendazole | C13 H17 N3 O2 | 247.13164 | 0.338804 | -1.56148 | 8.77E-08 | 0.986111 | 1.152387 | down |
| Com_21671_pos | fenfluramine | C12 H16 F3 N | 231.12414 | 0.359355 | -1.47652 | 3.56E-06 | 0.972222 | 1.044019 | down |
| Com_2173_pos | Pentoxifylline | C13 H18 N4 O3 | 278.13755 | 0.206879 | -2.27314 | 1.29E-06 | 0.972222 | 1.742645 | down |
| Com_21767_pos | Molindone | C16 H24 N2 O2 | 276.18315 | 0.339471 | -1.55864 | 6.96E-06 | 0.965278 | 1.104795 | down |
| Com_2180_pos | 2978 | C14 H16 O2 | 216.11487 | 0.163875 | -2.60933 | 7.35E-07 | 1 | 2.005785 | down |
| Com_21849_pos | (8E)-9-(1,3-Benzodioxol-5-yl)-1-(1-piperidinyl)-8-nonen-1-one | C21 H29 N O3 | 343.21562 | 0.333008 | -1.58637 | 4.30E-06 | 0.986111 | 1.17854 | down |
| Com_21932_pos | Mocetinostat | C23 H20 N6 O | 396.17095 | 0.317498 | -1.65518 | 1.99E-05 | 0.951389 | 1.406085 | down |
| Com_2197_pos | 8-hydroxy-7-methylguanine | C6 H7 N5 O2 | 181.06024 | 0.156399 | -2.6767 | 2.08E-05 | 0.986111 | 2.135529 | down |
| Com_21987_pos | 2-Methyl-1,2-bis(3-pyridyl)-1-propanol | C14 H16 N2 O | 228.12622 | 0.323052 | -1.63016 | 1.40E-05 | 0.9375 | 1.402476 | down |
| Com_2207_pos | Metolachlor morpholinone | C14 H19 N O2 | 233.14136 | 0.155094 | -2.68879 | 1.76E-06 | 1 | 2.103229 | down |
| Com_22240_pos | spiperone | C23 H26 F N3 O2 | 395.20262 | 0.280979 | -1.83147 | 1.19E-05 | 0.951389 | 1.316321 | down |
| Com_2293_pos | amfonelic acid | C18 H16 N2 O3 | 308.11558 | 0.021861 | -5.51547 | 1.03E-14 | 1 | 4.051252 | down |
| Com_2381_pos | N-Acetyl-DL-tryptophan | C13 H14 N2 O3 | 246.10014 | 0.362312 | -1.4647 | 5.04E-06 | 0.979167 | 1.101758 | down |
| Com_2383_pos | 3-Methoxyflavone | C16 H12 O3 | 274.05944 | 0.043027 | -4.53861 | 1.39E-08 | 1 | 3.728456 | down |
| Com_2387_pos | (-)-nabilone | C24 H36 O3 | 372.26565 | 0.186117 | -2.42572 | 0.00028 | 0.888889 | 2.328466 | down |
| Com_2404_pos | androstenol | C19 H30 O | 274.22917 | 0.176647 | -2.50106 | 3.45E-06 | 0.979167 | 1.940369 | down |
| Com_2425_pos | Cafestol | C20 H28 O3 | 316.20321 | 0.347092 | -1.52661 | 3.48E-06 | 0.972222 | 1.088859 | down |
| Com_2441_pos | Sedanolide | C12 H18 O2 | 194.1306 | 0.307382 | -1.70189 | 1.96E-05 | 0.9375 | 1.207135 | down |
| Com_2526_pos | spermidine | C7 H19 N3 | 145.15779 | 0.239509 | -2.06185 | 1.56E-07 | 1 | 1.460287 | down |
| Com_2537_pos | (4S)-4-[(2E)-2-Octenoyloxy]-4-(trimethylammonio)butanoate | C15 H27 N O4 | 285.19369 | 0.239224 | -2.06357 | 1.00E-07 | 1 | 1.582696 | down |
| Com_2570_pos | Adrafinil | C15 H15 N O3 S | 289.07675 | 0.249014 | -2.0057 | 9.22E-05 | 0.986111 | 1.830827 | down |
| Com_2611_pos | (17beta)-17-Hydroxyestra-1(10),2,4-trien-3-yl  D-glucopyranosiduronic acid | C24 H32 O8 | 448.21182 | 0.302854 | -1.7233 | 6.70E-08 | 0.979167 | 1.297826 | down |
| Com_2657_pos | Myriocin | C21 H39 N O6 | 401.27715 | 0.325776 | -1.61805 | 9.12E-09 | 0.993056 | 1.209746 | down |
| Com_2715_pos | Palmitic Acid | C16 H32 O2 | 256.23971 | 0.262884 | -1.9275 | 0.000431 | 0.965278 | 1.238177 | down |
| Com_2718_pos | (2E)-3-(Carbamimidoylsulfanyl)acrylic acid | C4 H6 N2 O2 S | 146.01463 | 0.069463 | -3.8476 | 6.55E-06 | 0.944444 | 3.047345 | down |
| Com_2750_pos | saccharopine | C11 H20 N2 O6 | 276.13163 | 0.25258 | -1.98519 | 6.06E-07 | 0.993056 | 1.442675 | down |
| Com_2808_pos | 5-Chloro THJ 018 | C23 H21 Cl N2 O | 376.13779 | 0.31208 | -1.68001 | 3.57E-05 | 0.944444 | 1.13267 | down |
| Com_2813_pos | N-Benzyl-4-piperidone | C12 H15 N O | 189.11522 | 0.136406 | -2.87402 | 1.68E-10 | 1 | 2.045264 | down |
| Com_2868_pos | Hexanoylcarnitine | C13 H25 N O4 | 259.17805 | 0.382084 | -1.38804 | 1.18E-05 | 0.979167 | 1.060463 | down |
| Com_2958_pos | Palmitoylcarnitine | C23 H45 N O4 | 399.33421 | 0.491501 | -1.02473 | 0.001716 | 0.875 | 1.02158 | down |
| Com_2961_pos | Imagabalin | C9 H19 N O2 | 173.14153 | 0.223991 | -2.15849 | 2.10E-09 | 1 | 1.576101 | down |
| Com_3020_pos | CONDELPHINE | C25 H39 N O6 | 449.27732 | 0.228951 | -2.12689 | 5.68E-08 | 0.993056 | 1.562396 | down |
| Com_3048_pos | Ethyl malate | C8 H14 O5 | 190.08408 | 0.216615 | -2.20679 | 1.79E-09 | 1 | 1.625142 | down |
| Com_3052_pos | PD-128042 | C23 H39 N O4 | 393.28723 | 0.173541 | -2.52665 | 8.11E-10 | 1 | 1.870701 | down |
| Com_3059_pos | N-OLEOYL-4-AMINOBUTYRIC ACID | C22 H41 N O3 | 367.30779 | 0.419875 | -1.25197 | 0.000808 | 0.868056 | 1.198239 | down |
| Com_3060_pos | Ethyl eicosapentaenoic acid | C22 H34 O2 | 330.25511 | 0.381537 | -1.3901 | 0.0003 | 0.868056 | 1.044059 | down |
| Com_3081_pos | Acipimox | C6 H6 N2 O3 | 154.0378 | 0.272902 | -1.87354 | 6.22E-07 | 0.986111 | 1.351339 | down |
| Com_30_pos | Docosahexaenoic acid ethyl ester | C24 H36 O2 | 356.27087 | 0.213517 | -2.22758 | 2.23E-06 | 0.979167 | 1.694727 | down |
| Com_3131_pos | Dinoseb | C10 H12 N2 O5 | 240.07437 | 0.339157 | -1.55998 | 0.018341 | 0.763889 | 1.376664 | down |
| Com_3136_pos | Dihexyl phthalate | C20 H30 O4 | 334.21377 | 0.282614 | -1.8231 | 3.89E-07 | 1 | 1.265527 | down |
| Com_315_pos | 4-hyroxy-5-methyl-3-furanone | C5 H6 O3 | 114.03169 | 0.367493 | -1.44421 | 0.000847 | 0.881944 | 1.265795 | down |
| Com_3169_pos | Athamantin | C24 H30 O7 | 430.20124 | 0.388023 | -1.36579 | 0.000143 | 0.888889 | 1.12814 | down |
| Com_3238_pos | MYRISTOYLLEVOCARNITINE | C21 H41 N O4 | 371.30292 | 0.369903 | -1.43478 | 2.39E-06 | 0.986111 | 1.109142 | down |
| Com_327_pos | n-Hexanamide | C6 H13 N O | 115.09968 | 0.351749 | -1.50738 | 0.000252 | 0.951389 | 1.29188 | down |
| Com_3295_pos | trans-2-Tetradecenoylcarnitine | C21 H39 N O4 | 369.2874 | 0.227686 | -2.13488 | 1.64E-10 | 1 | 1.573701 | down |
| Com_3298_pos | Ethyldiphenylphosphine oxide | C14 H15 O P | 230.08746 | 0.269356 | -1.89241 | 6.85E-07 | 1 | 1.432513 | down |
| Com_3329_pos | JWH-147 | C27 H27 N O | 381.21462 | 0.278858 | -1.8424 | 8.01E-07 | 0.979167 | 1.384046 | down |
| Com_3342_pos | LY-294,002 | C19 H17 N O3 | 307.11968 | 0.274017 | -1.86766 | 4.24E-05 | 0.951389 | 1.304837 | down |
| Com_3376_pos | (R)-3-hydroxybutyrylcarnitine | C11 H21 N O5 | 247.14165 | 0.302561 | -1.7247 | 2.48E-06 | 0.979167 | 1.266194 | down |
| Com_3404_pos | picoprazole | C17 H17 N3 O3 S | 343.09842 | 0.123375 | -3.01888 | 7.42E-10 | 1 | 2.195704 | down |
| Com_348_pos | Glutaric acid | C5 H8 O4 | 132.0422 | 0.335437 | -1.57589 | 0.000417 | 0.881944 | 1.371903 | down |
| Com_3495_pos | His-pro | C11 H16 N4 O3 | 252.12193 | 0.338575 | -1.56245 | 2.66E-07 | 0.958333 | 1.103089 | down |
| Com_3497_pos | (3S,6S)-3-(4-Hydroxybenzyl)-6-(hydroxymethyl)  -2,5-piperazinedione | C12 H14 N2 O4 | 250.09508 | 0.328408 | -1.60644 | 5.91E-07 | 0.986111 | 1.150236 | down |
| Com_3506_pos | 2-(Methylsulfanyl)-3H-phenoxazin-3-one | C13 H9 N O2 S | 243.03491 | 0.324789 | -1.62242 | 0.00024 | 0.965278 | 1.492214 | down |
| Com_3523_pos | Cannabinol monomethyl ether | C22 H28 O2 | 324.20821 | 0.30567 | -1.70995 | 9.79E-08 | 1 | 1.202526 | down |
| Com_3621_pos | XLR11 N-(2-fluoropentyl) isomer | C21 H28 F N O | 329.21977 | 0.244247 | -2.03359 | 3.63E-07 | 0.993056 | 1.551229 | down |
| Com_3674_pos | Juvenile hormone III | C16 H26 O3 | 266.18775 | 0.451083 | -1.14854 | 0.002809 | 0.861111 | 1.124951 | down |
| Com_3740_pos | Leukotriene B4 | C20 H32 O4 | 336.22942 | 0.168372 | -2.57027 | 6.82E-06 | 0.993056 | 1.697951 | down |
| Com_374_pos | DL-Carnitine | C7 H15 N O3 | 161.10513 | 0.347098 | -1.52659 | 3.67E-07 | 0.993056 | 1.141745 | down |
| Com_37_pos | Creatinine | C4 H7 N3 O | 113.05888 | 0.481678 | -1.05386 | 0.045005 | 0.756944 | 1.016912 | down |
| Com_3859_pos | methyl 3-hydroxypalmitate | C17 H34 O3 | 286.25019 | 0.337004 | -1.56916 | 5.05E-05 | 0.895833 | 1.121856 | down |
| Com_3932_pos | (-)-Lupinine | C10 H19 N O | 169.1475 | 0.129476 | -2.94924 | 2.29E-07 | 0.972222 | 2.234952 | down |
| Com_4014_pos | 4-Phenyl-3-buten-2-one | C10 H10 O | 146.07312 | 0.280485 | -1.83401 | 0.000229 | 0.916667 | 1.429653 | down |
| Com_4034_pos | 3beta-Fluoro-5beta-pregnan-20-one | C21 H33 F O | 320.25136 | 0.207081 | -2.27173 | 0.000259 | 0.979167 | 1.469494 | down |
| Com_4059_pos | Phenazone | C11 H12 N2 O | 188.09489 | 0.323807 | -1.62679 | 0.000447 | 0.909722 | 1.161453 | down |
| Com_4122_pos | Valclavam | C14 H23 N3 O6 | 329.15825 | 0.16191 | -2.62674 | 9.00E-07 | 0.979167 | 1.923057 | down |
| Com_4131_pos | Pardoprunox | C12 H15 N3 O2 | 233.11621 | 0.125417 | -2.99519 | 1.73E-06 | 0.979167 | 2.306411 | down |
| Com_4147_pos | UNII:OUT5YHB7BO | C18 H35 N O2 | 297.26627 | 0.297178 | -1.7506 | 1.18E-05 | 0.951389 | 1.356628 | down |
| Com_4188_pos | (3alpha,4beta,8alpha)-4,15-Diacetoxy-3-hydroxy  -12,13-epoxytrichothec-9-en-8-yl hexanoate | C25 H36 O9 | 480.23802 | 0.299826 | -1.7378 | 2.18E-07 | 0.979167 | 1.281996 | down |
| Com_4192_pos | DL-Mevalonic acid | C6 H12 O4 | 148.07385 | 0.346274 | -1.53001 | 0.000371 | 0.909722 | 1.033664 | down |
| Com_421_pos | Succinic acid | C4 H6 O4 | 118.0266 | 0.318014 | -1.65284 | 0.000792 | 0.916667 | 1.637339 | down |
| Com_4225_pos | Sinapyl alcohol | C11 H14 O4 | 210.08915 | 0.255198 | -1.97031 | 5.75E-05 | 0.930556 | 1.532107 | down |
| Com_4297_pos | pnb | C12 H9 N O2 | 199.06314 | 0.312363 | -1.67871 | 0.000272 | 0.9375 | 1.23847 | down |
| Com_4300_pos | 2-Amino-1,3,4-octadecanetriol | C18 H39 N O3 | 317.29232 | 0.154195 | -2.69717 | 1.99E-07 | 1 | 1.886661 | down |
| Com_4331_pos | 2-Oxosuberate | C8 H12 O5 | 188.0683 | 0.347121 | -1.52649 | 0.000439 | 0.9375 | 1.122381 | down |
| Com_4342_pos | Methyl-2-aminobenzoate | C8 H9 N O2 | 151.06318 | 0.333183 | -1.58561 | 8.92E-08 | 1 | 1.195023 | down |
| Com_4390_pos | 2-{4-[(1-Oxido-2-thienyl)carbonyl]phenyl}propanoic acid | C14 H12 O4 S | 276.04505 | 0.499664 | -1.00097 | 0.003613 | 0.875 | 1.006318 | down |
| Com_4396_pos | obacunone | C26 H30 O7 | 454.20017 | 0.301014 | -1.7321 | 0.000562 | 0.895833 | 1.481782 | down |
| Com_4425_pos | quindoxin | C8 H6 N2 O2 | 162.04286 | 0.308368 | -1.69728 | 2.45E-08 | 0.986111 | 1.257715 | down |
| Com_4433_pos | Aflatoxin B2 | C17 H14 O6 | 336.05964 | 0.42783 | -1.22489 | 0.000548 | 0.895833 | 1.202874 | down |
| Com_4443_pos | 1,2,3,4-tetrahydro-beta-carboline-3-carboxylic acid | C12 H12 N2 O2 | 216.08979 | 0.297139 | -1.75079 | 0.000707 | 0.909722 | 1.337238 | down |
| Com_4468_pos | N-Palmitoyl taurine | C18 H37 N O4 S | 363.24038 | 0.234962 | -2.0895 | 2.38E-07 | 1 | 1.503425 | down |
| Com_4496_pos | Ethyl benzoylacetate | C11 H12 O3 | 192.07862 | 0.265961 | -1.91071 | 8.30E-05 | 0.930556 | 1.487968 | down |
| Com_453_pos | Acetyl-L-carnitine | C9 H17 N O4 | 203.11572 | 0.282905 | -1.82161 | 3.57E-08 | 1 | 1.382103 | down |
| Com_4558_pos | Methylphenidate | C14 H19 N O2 | 233.14132 | 0.161614 | -2.62938 | 4.02E-10 | 1 | 1.866135 | down |
| Com_4577_pos | trans-geranic acid | C10 H16 O2 | 168.1146 | 0.31201 | -1.68034 | 0.000194 | 0.930556 | 1.42008 | down |
| Com_4631_pos | 2-[(Dimethylamino)methylidene]indan-1-one | C12 H13 N O | 187.09968 | 0.281146 | -1.83061 | 7.30E-07 | 0.972222 | 1.357094 | down |
| Com_4632_pos | N1-[4-(1,3-Oxazol-5-yl)phenyl]cyclopropane-1-carboxamide | C13 H12 N2 O2 | 228.08968 | 0.292472 | -1.77363 | 5.81E-06 | 0.958333 | 1.337157 | down |
| Com_469_pos | Acetylcholine | C7 H15 N O2 | 145.11023 | 0.33749 | -1.56708 | 2.40E-07 | 0.993056 | 1.152779 | down |
| Com_4739_pos | Epirizole | C11 H14 N4 O2 | 234.11131 | 0.369781 | -1.43526 | 1.85E-05 | 0.965278 | 1.015271 | down |
| Com_4772_pos | 3-[(3-Hydroxydecanoyl)oxy]-4-(trimethylammonio)butanoate | C17 H33 N O5 | 331.23533 | 0.198365 | -2.33377 | 7.72E-09 | 1 | 1.729757 | down |
| Com_4828_pos | 3-[(3-Hydroxytridecanoyl)oxy]-4-(trimethylammonio)butanoate | C20 H39 N O5 | 373.28223 | 0.229963 | -2.12053 | 2.03E-12 | 1 | 1.571394 | down |
| Com_4925_pos | Ethylmorphine | C19 H23 N O3 | 313.1732 | 0.206206 | -2.27784 | 9.61E-07 | 0.965278 | 1.662648 | down |
| Com_4928_pos | glutethimide | C13 H15 N O2 | 217.11007 | 0.137658 | -2.86084 | 3.11E-09 | 1 | 2.016301 | down |
| Com_5004_pos | zindotrine | C11 H15 N5 | 217.13329 | 0.314387 | -1.66939 | 6.89E-08 | 1 | 1.22122 | down |
| Com_5026_pos | Pregabalin | C8 H17 N O2 | 159.12589 | 0.389551 | -1.36012 | 8.97E-06 | 0.958333 | 1.000113 | down |
| Com_5058_pos | Dipivefrin | C19 H29 N O5 | 351.20398 | 0.147976 | -2.75656 | 2.16E-06 | 1 | 1.911331 | down |
| Com_5063_pos | METIPRANOLOL | C17 H27 N O4 | 309.19365 | 0.140132 | -2.83514 | 3.48E-10 | 1 | 2.058905 | down |
| Com_5079_pos | trp-lys | C17 H24 N4 O3 | 332.18438 | 0.14762 | -2.76004 | 9.38E-08 | 1 | 2.062443 | down |
| Com_5117_pos | flumetasone | C22 H28 F2 O5 | 410.1901 | 0.367948 | -1.44243 | 3.28E-05 | 0.944444 | 1.083957 | down |
| Com_5124_pos | JWH 213 | C27 H29 N O | 383.23025 | 0.322153 | -1.63418 | 9.84E-06 | 0.958333 | 1.254437 | down |
| Com_5154_pos | Rupatadine | C26 H26 Cl N3 | 415.18217 | 0.20947 | -2.25519 | 2.78E-05 | 0.930556 | 1.796096 | down |
| Com_5200_pos | piroximone | C11 H11 N3 O2 | 217.08503 | 0.107759 | -3.21412 | 1.34E-10 | 1 | 2.348182 | down |
| Com_5290_pos | psychotrine | C28 H36 N2 O4 | 464.26717 | 0.222853 | -2.16583 | 1.33E-09 | 1 | 1.584596 | down |
| Com_5314_pos | 2-Methoxy-3-methylpyrazine | C6 H8 N2 O | 124.06376 | 0.405876 | -1.30089 | 2.64E-05 | 0.9375 | 1.022171 | down |
| Com_5368_pos | Androst-4-en-3-one | C19 H28 O | 272.21352 | 0.12781 | -2.96793 | 1.39E-09 | 1 | 2.101615 | down |
| Com_5428_pos | Salmeterol | C25 H37 N O4 | 415.27169 | 0.194191 | -2.36445 | 8.25E-09 | 0.993056 | 1.722883 | down |
| Com_5448_pos | O-nonanoylcarnitine | C16 H31 N O4 | 301.22475 | 0.161746 | -2.6282 | 2.29E-06 | 1 | 2.107611 | down |
| Com_544_pos | 2-Arachidonoyl glycerol | C23 H38 O4 | 378.27641 | 0.346056 | -1.53092 | 0.00024 | 0.923611 | 1.194983 | down |
| Com_551_pos | Succinic anhydride | C4 H4 O3 | 100.01605 | 0.297195 | -1.75052 | 0.001508 | 0.840278 | 1.53071 | down |
| Com_5566_pos | bencyclane | C19 H31 N O | 289.24016 | 0.119854 | -3.06066 | 1.44E-09 | 1 | 2.166734 | down |
| Com_556_pos | 1-(beta-D-ribofuranosyl)thymine | C10 H14 N2 O6 | 258.08493 | 0.358805 | -1.47873 | 0.016648 | 0.784722 | 1.371242 | down |
| Com_5592_pos | DEMETHYLCHLORPROMAZINE | C16 H17 Cl N2 S | 304.08153 | 0.390736 | -1.35574 | 0.000435 | 0.909722 | 1.148301 | down |
| Com_5614_pos | Indane | C9 H10 | 118.07822 | 0.345032 | -1.5352 | 8.85E-05 | 0.930556 | 1.175487 | down |
| Com_5618_pos | Ulipristal | C28 H35 N O3 | 433.26096 | 0.199259 | -2.32729 | 0.00312 | 0.8125 | 1.615151 | down |
| Com_5664_pos | 2-Propynyl N-[6-(phenylsulfanyl)-5-  (trifluoromethyl)-3-pyridinyl]carbamate | C16 H11 F3 N2 O2 S | 352.04883 | 0.235887 | -2.08383 | 2.31E-06 | 0.986111 | 1.674981 | down |
| Com_5687_pos | Cerivastatin | C26 H34 F N O5 | 459.24387 | 0.170377 | -2.5532 | 2.83E-08 | 0.986111 | 1.965288 | down |
| Com_5717_pos | Schaftoside | C26 H28 O14 | 564.14764 | 0.229335 | -2.12447 | 4.52E-06 | 0.958333 | 1.65235 | down |
| Com_5721_pos | 4-tert-Octylphenol monoethoxylate | C16 H26 O2 | 250.19427 | 0.251595 | -1.99082 | 4.43E-05 | 0.909722 | 1.383041 | down |
| Com_5766_pos | Oxycarboxin | C12 H13 N O4 S | 267.05617 | 0.284993 | -1.811 | 1.26E-06 | 0.972222 | 1.309278 | down |
| Com_5811_pos | aspergillic acid | C12 H20 N2 O2 | 224.15222 | 0.240563 | -2.05552 | 9.37E-07 | 1 | 1.441118 | down |
| Com_591_pos | (+/-)-Methoprene | C19 H34 O3 | 310.25019 | 0.392931 | -1.34765 | 0.002299 | 0.861111 | 1.033614 | down |
| Com_606_pos | pentoxyl | C6 H8 N2 O3 | 156.05349 | 0.31845 | -1.65086 | 7.22E-05 | 0.930556 | 1.196274 | down |
| Com_6111_pos | (M)-murrastifoline-F | C28 H24 N2 O2 | 420.18266 | 0.27961 | -1.83851 | 1.40E-05 | 0.958333 | 1.632313 | down |
| Com_6200_pos | Oleoylethanolamide | C20 H39 N O2 | 325.29743 | 0.237898 | -2.07158 | 0.000343 | 0.951389 | 1.37163 | down |
| Com_624_pos | lys-leu | C12 H25 N3 O3 | 259.18931 | 0.37817 | -1.40289 | 5.62E-07 | 0.993056 | 1.07539 | down |
| Com_629_pos | spinacine | C7 H9 N3 O2 | 167.06948 | 0.169933 | -2.55696 | 9.50E-05 | 0.9375 | 1.972088 | down |
| Com_6389_pos | indeloxazine | C14 H17 N O2 | 231.12563 | 0.200729 | -2.31668 | 8.70E-09 | 1 | 1.722371 | down |
| Com_6393_pos | Carbamazepine | C15 H12 N2 O | 258.07495 | 0.252421 | -1.9861 | 3.85E-07 | 0.993056 | 1.508158 | down |
| Com_6530_pos | dapdiamide A | C12 H20 N4 O5 | 300.14287 | 0.176288 | -2.504 | 1.08E-06 | 0.986111 | 1.891378 | down |
| Com_6531_pos | Phenylethanolamine | C8 H11 N O | 137.08407 | 0.334548 | -1.57972 | 0.000161 | 0.923611 | 1.216004 | down |
| Com_6568_pos | N-Acetylcytidine | C11 H15 N3 O6 | 285.09563 | 0.29825 | -1.74541 | 3.91E-05 | 0.958333 | 1.194427 | down |
| Com_6587_pos | N-[2-(4-{[(2E)-5-(3,3-Dimethyl-2-oxiranyl)-4-  hydroxy-3-methyl-2-penten-1-yl]oxy}phenyl)  ethyl]benzamide | C25 H31 N O4 | 409.22563 | 0.246575 | -2.0199 | 6.85E-07 | 0.993056 | 1.623499 | down |
| Com_6800_pos | (Z)-Endoxifen | C25 H27 N O2 | 373.20406 | 0.41698 | -1.26195 | 0.018104 | 0.8125 | 1.350184 | down |
| Com_6811_pos | ethyl ethoxalylpropionate | C9 H14 O5 | 202.08415 | 0.363295 | -1.46079 | 0.001465 | 0.854167 | 1.112592 | down |
| Com_6829_pos | Linoleamide | C18 H33 N O | 279.25565 | 0.31482 | -1.6674 | 0.00015 | 0.902778 | 1.401756 | down |
| Com_6881_pos | Civetone | C17 H30 O | 250.22931 | 0.201155 | -2.31362 | 0.000763 | 0.902778 | 1.451918 | down |
| Com_6923_pos | (-)-Prostaglandin E1 | C20 H34 O5 | 354.24038 | 0.247525 | -2.01435 | 4.14E-08 | 0.993056 | 1.513116 | down |
| Com_694_pos | 4-amino-2-hydroxyamino-6-nitrotoluene | C7 H9 N3 O3 | 183.06435 | 0.258348 | -1.95261 | 9.48E-05 | 0.923611 | 1.720509 | down |
| Com_6983_pos | sultiame | C10 H14 N2 O4 S2 | 290.03907 | 0.348101 | -1.52242 | 0.00019 | 0.888889 | 1.063431 | down |
| Com_6993_pos | N-(2-Cyanoethyl)-L-glutamine | C8 H13 N3 O3 | 199.09578 | 0.346633 | -1.52852 | 4.85E-05 | 0.930556 | 1.190852 | down |
| Com_702_pos | 3-(2,4-Cyclopentadien-1-ylidene)-5alpha-  androstan-17beta-ol | C24 H34 O | 338.26031 | 0.197618 | -2.33921 | 3.12E-07 | 0.986111 | 1.790668 | down |
| Com_7095_pos | levorphanol | C17 H23 N O | 257.17761 | 0.317386 | -1.65569 | 1.23E-05 | 0.944444 | 1.334209 | down |
| Com_7144_pos | N-(4Z,7Z,10Z,13Z,16Z,19Z)-docosahexaenoylethanolamine | C24 H37 N O2 | 371.28176 | 0.278371 | -1.84492 | 7.55E-08 | 0.993056 | 1.375877 | down |
| Com_7220_pos | N-Acetyl-L-cysteine | C5 H9 N O3 S | 163.03025 | 0.319326 | -1.6469 | 0.002007 | 0.840278 | 1.175205 | down |
| Com_7272_pos | tretoquinol | C19 H23 N O5 | 345.1568 | 0.106506 | -3.23099 | 9.11E-08 | 1 | 2.16929 | down |
| Com_7294_pos | Oxprenolol | C15 H23 N O3 | 265.16742 | 0.373809 | -1.41963 | 3.73E-05 | 0.951389 | 1.116193 | down |
| Com_7302_pos | Nicorandil | C8 H9 N3 O4 | 211.05898 | 0.346998 | -1.527 | 5.51E-05 | 0.930556 | 1.25908 | down |
| Com_734_pos | (2S)-5-Carbamimidamido-2-(2-oxo-1-azetidinyl)pentanoic acid | C9 H16 N4 O3 | 228.12203 | 0.408219 | -1.29259 | 0.001264 | 0.888889 | 1.005246 | down |
| Com_7364_pos | 3-hydroxyoctanoylcarnitine | C15 H29 N O5 | 303.2041 | 0.203065 | -2.29999 | 2.19E-10 | 1 | 1.670398 | down |
| Com_7378_pos | 5-Formyluracil | C5 H4 N2 O3 | 140.02213 | 0.281241 | -1.83012 | 1.46E-06 | 0.986111 | 1.301296 | down |
| Com_7383_pos | Sepiapterin | C9 H11 N5 O3 | 237.08601 | 0.205261 | -2.28447 | 0.000204 | 0.909722 | 1.581192 | down |
| Com_7391_pos | N-Undecanoylglycine | C13 H25 N O3 | 243.1832 | 0.177772 | -2.4919 | 4.77E-10 | 1 | 1.83153 | down |
| Com_7418_pos | PYROQUILON | C11 H11 N O | 173.084 | 0.193967 | -2.36612 | 9.54E-08 | 1 | 1.616692 | down |
| Com_7430_pos | Malondialdehyde | C3 H4 O2 | 72.02114 | 0.396417 | -1.33491 | 1.11E-05 | 0.951389 | 1.063009 | down |
| Com_7492_pos | Xylitol | C5 H12 O5 | 152.06837 | 0.216442 | -2.20794 | 2.25E-09 | 1 | 1.567318 | down |
| Com_7614_pos | 3-Hydroxy-cis-5-tetradecenoylcarnitine | C21 H39 N O5 | 385.28215 | 0.185998 | -2.42664 | 8.66E-11 | 1 | 1.797708 | down |
| Com_761_pos | N-Acetyl-L-histidine | C8 H11 N3 O3 | 197.08 | 0.34689 | -1.52745 | 3.28E-05 | 0.944444 | 1.120526 | down |
| Com_7629_pos | Butabarbital | C10 H16 N2 O3 | 212.11607 | 0.369431 | -1.43662 | 9.81E-08 | 1 | 1.073643 | down |
| Com_7802_pos | Succinimide | C4 H5 N O2 | 99.03191 | 0.332369 | -1.58914 | 2.03E-05 | 0.958333 | 1.129353 | down |
| Com_783_pos | Cholecalciferol | C27 H44 O | 384.33869 | 0.204243 | -2.29164 | 2.69E-06 | 0.951389 | 1.913496 | down |
| Com_7846_pos | 6721 | C16 H20 N6 | 296.17382 | 0.383344 | -1.38329 | 0.005099 | 0.805556 | 1.247869 | down |
| Com_7848_pos | Atropine | C17 H23 N O3 | 289.16737 | 0.203307 | -2.29827 | 3.03E-07 | 1 | 1.784685 | down |
| Com_788_pos | Tomelukast | C16 H22 N4 O3 | 318.16866 | 0.145211 | -2.78377 | 1.18E-07 | 1 | 2.142209 | down |
| Com_7914_pos | 6-hydroxypseudooxynicotine | C10 H14 N2 O2 | 194.10554 | 0.302934 | -1.72292 | 1.95E-07 | 1 | 1.359932 | down |
| Com_7973_pos | (Ac)2-L-Lys-D-Ala | C13 H23 N3 O5 | 301.16339 | 0.384663 | -1.37833 | 0.000632 | 0.895833 | 1.02617 | down |
| Com_7992_pos | asulam | C8 H10 N2 O4 S | 230.0358 | 0.281697 | -1.82778 | 1.68E-07 | 0.993056 | 1.343494 | down |
| Com_8004_pos | 2,6-Diamino-4-hexenoic acid | C6 H12 N2 O2 | 144.0898 | 0.336831 | -1.5699 | 2.14E-06 | 0.965278 | 1.120442 | down |
| Com_8011_pos | amquinate | C18 H24 N2 O3 | 316.17801 | 0.219943 | -2.1848 | 6.13E-08 | 1 | 1.534489 | down |
| Com_8023_pos | (1'R,3S,3'R,3aS,4S,4aR,7'R,7aR,8R,9'R,9aR,10'R)-4-  Hydroxy-4a,8,9'-trimethyl-4'-methylene-4,4a,7a,8,9,9a-  hexahydro-5'H,14'H-spiro[azuleno[6,5-b]furan-3,13'-  [6]oxatetracyclo[9.2.2.0~1,10~.0~3,7~]pentadecane]  -2,5,5',14'(3aH)-tetrone | C29 H34 O7 | 494.23232 | 0.257311 | -1.95841 | 6.24E-05 | 0.972222 | 1.234816 | down |
| Com_808_pos | 2,3-Bis(octanoyloxy)propanimidic acid | C19 H35 N O5 | 357.25101 | 0.308631 | -1.69605 | 2.86E-09 | 1 | 1.263993 | down |
| Com_8155_pos | sampangine | C15 H8 N2 O | 232.06291 | 0.238335 | -2.06894 | 2.27E-08 | 0.993056 | 1.472302 | down |
| Com_8181_pos | g-Butyrobetaine | C7 H15 N O2 | 145.11023 | 0.308805 | -1.69523 | 2.16E-06 | 0.993056 | 1.194139 | down |
| Com_8197_pos | Hydroxyhexanoycarnitine | C13 H25 N O5 | 275.17288 | 0.287433 | -1.7987 | 4.39E-05 | 0.944444 | 1.348864 | down |
| Com_8276_pos | Geranyllinalool | C20 H34 O | 290.26043 | 0.354492 | -1.49617 | 0.00067 | 0.881944 | 1.063176 | down |
| Com_8398_pos | Tetrahydrogeranylgeranyl diphosphate | C20 H40 O7 P2 | 454.22295 | 0.265827 | -1.91144 | 0.001214 | 0.861111 | 1.235068 | down |
| Com_8537_pos | Valyltyrosine | C14 H20 N2 O4 | 280.14187 | 0.344381 | -1.53792 | 8.42E-06 | 0.944444 | 1.092763 | down |
| Com_8569_pos | Senkyunolide H | C12 H16 O4 | 224.10482 | 0.289336 | -1.78918 | 0.000241 | 0.909722 | 1.455829 | down |
| Com_8583_pos | 4-(Trimethylammonio)-3-(undecanoyloxy)butanoate | C18 H35 N O4 | 329.25588 | 0.188466 | -2.40762 | 5.39E-09 | 0.993056 | 1.70919 | down |
| Com_8635_pos | Dibenzothiophene sulfone | C12 H8 O2 S | 216.02432 | 0.466417 | -1.10031 | 0.001204 | 0.916667 | 1.005833 | down |
| Com_8660_pos | Sarpogrelate | C24 H31 N O6 | 429.21676 | 0.232635 | -2.10386 | 4.98E-06 | 0.944444 | 1.500959 | down |
| Com_8675_pos | Nonivamide | C17 H27 N O3 | 293.19854 | 0.358239 | -1.48101 | 9.93E-06 | 0.965278 | 1.041937 | down |
| Com_8864_pos | Kynurenic acid | C10 H7 N O3 | 189.04253 | 0.40466 | -1.30522 | 0.000113 | 0.909722 | 1.060583 | down |
| Com_8873_pos | Methadone | C21 H27 N O | 309.20487 | 0.213277 | -2.2292 | 2.74E-09 | 1 | 1.579423 | down |
| Com_8945_pos | Cammaconine | C23 H37 N O5 | 407.26642 | 0.248858 | -2.00661 | 6.18E-08 | 1 | 1.453153 | down |
| Com_898_pos | 9-Decenoylcarnitine | C17 H31 N O4 | 313.22478 | 0.264149 | -1.92058 | 2.07E-08 | 1 | 1.437357 | down |
| Com_9011_pos | Butralin | C14 H21 N3 O4 | 295.15272 | 0.380185 | -1.39523 | 0.000412 | 0.895833 | 1.125121 | down |
| Com_9054_pos | 2-(1-Hydroxy-2,4,6-trimethyl-3-oxo-2,3-  dihydro-1H-inden-5-yl)ethyl acetate | C16 H20 O4 | 276.13598 | 0.301962 | -1.72756 | 1.63E-11 | 1 | 1.286354 | down |
| Com_9209_pos | Tandutinib | C31 H42 N6 O4 | 562.32834 | 0.280856 | -1.8321 | 3.00E-06 | 0.972222 | 1.287075 | down |
| Com_9238_pos | 5-Nitro-o-toluidine | C7 H8 N2 O2 | 152.05835 | 0.420718 | -1.24907 | 0.000795 | 0.895833 | 1.047289 | down |
| Com_9252_pos | Teneligliptin | C22 H30 N6 O S | 426.22229 | 0.377005 | -1.40734 | 3.95E-07 | 0.986111 | 1.016599 | down |
| Com_9264_pos | (2E,4E,6E,8E,10E)-1-[5-Hydroxy-2-(2  -hydroxyethyl)-2H-pyrrol-4-yl]-10-  (hydroxymethyl)-2,6,8-trimethyl-2,4,6,8,  10-dodecapentaen-1-one | C22 H29 N O4 | 371.21138 | 0.072163 | -3.7926 | 2.10E-07 | 1 | 2.537709 | down |
| Com_9291_pos | (10E)-9,12,13-Trihydroxy-10-octadecenoic acid | C18 H34 O5 | 330.24008 | 0.317858 | -1.65355 | 2.25E-05 | 0.930556 | 1.202927 | down |
| Com_939_pos | Estradiol enanthate | C25 H36 O3 | 384.26573 | 0.328389 | -1.60652 | 0.00018 | 0.909722 | 1.257738 | down |
| Com_945_pos | Secnidazole | C7 H11 N3 O3 | 185.07991 | 0.26786 | -1.90045 | 4.19E-05 | 0.958333 | 1.274398 | down |
| Com_9495_pos | Lankacidin C | C25 H33 N O7 | 459.22699 | 0.399833 | -1.32253 | 0.000292 | 0.951389 | 1.108691 | down |
| Com_9547_pos | Trp-Phe | C20 H21 N3 O3 | 351.15769 | 0.106999 | -3.22433 | 9.86E-09 | 1 | 2.220799 | down |
| Com_9564_pos | S-[(2E,6E)-farnesyl]-L-cysteine | C18 H31 N O2 S | 325.20623 | 0.38677 | -1.37045 | 1.45E-05 | 0.923611 | 1.175905 | down |
| Com_9591_pos | 2-methylbutyrylcarnitine | C12 H23 N O4 | 245.16238 | 0.419953 | -1.2517 | 0.000228 | 0.909722 | 1.031023 | down |
| Com_9735_pos | UNII:PLI450958N | C28 H35 Cl N2 O3 | 482.23366 | 0.338377 | -1.5633 | 5.54E-08 | 0.993056 | 1.167661 | down |
| Com_9788_pos | Piscerythramine | C26 H29 N O6 | 451.19823 | 0.224832 | -2.15308 | 3.02E-06 | 0.993056 | 1.659226 | down |
| Com_983_pos | 4-Hydroxyprolylleucine | C11 H20 N2 O4 | 244.14202 | 0.305089 | -1.7127 | 2.58E-05 | 0.958333 | 1.335173 | down |
| Com_9875_pos | 2EBS4A61GK | C25 H26 Cl N O | 391.17204 | 0.303903 | -1.71832 | 0.007018 | 0.8125 | 1.052554 | down |
| Com_9880_pos | Rolipram | C16 H21 N O3 | 275.15175 | 0.245914 | -2.02378 | 1.06E-07 | 1 | 1.516003 | down |
| Com_9894_pos | Isocarboxazid | C12 H13 N3 O2 | 231.10059 | 0.086164 | -3.53677 | 2.53E-08 | 1 | 2.476895 | down |
| Com_9902_pos | 5-Methoxy-3-indoleaceate | C11 H11 N O3 | 205.07391 | 0.238615 | -2.06725 | 0.004013 | 0.840278 | 1.539652 | down |
| Com_9908_pos | 8,8-Dimethyl-3-(2-methyl-3-buten-2-yl)-  10-(3-methyl-2-buten-1-yl)-2H,8H-pyrano  [3,2-g]chromen-2-one | C24 H28 O3 | 364.20283 | 0.369292 | -1.43717 | 1.26E-05 | 0.951389 | 1.117564 | down |
| Com_9926_pos | Vargulin | C22 H27 N7 O | 405.22963 | 0.340999 | -1.55216 | 6.13E-06 | 0.944444 | 1.153711 | down |
| Com_9971_pos | 1H-Benzimidazol-5-ol | C7 H6 N2 O | 134.04797 | 0.40098 | -1.3184 | 5.55E-07 | 0.979167 | 1.033876 | down |
| Com_997_pos | 1-O-[(3alpha,5beta,7alpha)-3,7-Dihydroxy-24-  oxocholan-24-yl]-beta-D-galactopyranose | C30 H50 O9 | 554.34492 | 0.277452 | -1.84969 | 0.000526 | 0.888889 | 1.702082 | down |

| **Supplementary Table 14. The significnat differential metabolites in DI contents of SBM20 vs. FM in negative mode (n = 12)** | | | | | | | | |
| --- | --- | --- | --- | --- | --- | --- | --- | --- |
|
| ID | Name_des | Formula | Molecular Weight | log2FC | Pvalue | ROC | VIP | Up.Down |
| Com_1800_neg | FMNH2 | C17 H23 N4 O9 P | 458.12116 | 4.881521947 | 2.53E-08 | 1 | 5.083650443 | up |
| Com_1700_neg | 2-[(2E,6E,10Z)-14,15-Dihydroxy-11-(hydroxymethyl)  -3,7,15-trimethyl-2,6,10-hexadecatrien-1-yl]-2,4,6,9-  tetrahydroxy-5,7-dimethyl-1H-phenalene-1,3(2H)-dione | C35 H46 O9 | 1220.62214 | 4.497002843 | 9.60E-10 | 1 | 5.056271388 | up |
| Com_988_neg | Glycitein | C16 H12 O5 | 284.06819 | 4.142369284 | 2.28E-09 | 1 | 4.441020929 | up |
| Com_1292_neg | 4,5-Dihydroxy-4-(2,3,4-trihydroxytetrahydro-2-furanyl)-  3,4-dihydro-2H-pyrrole-2-carboxylic acid | C9 H13 N O8 | 263.06379 | 3.847585607 | 5.10E-08 | 1 | 3.88940281 | up |
| Com_53_neg | Soyasaponin I | C48 H78 O18 | 942.52271 | 3.619941537 | 6.98E-10 | 1 | 3.885453181 | up |
| Com_4967_neg | olmelin | C16 H12 O5 | 284.06825 | 3.598936557 | 3.22E-08 | 0.99305556 | 3.678394927 | up |
| Com_796_neg | Genistein | C15 H10 O5 | 270.05251 | 3.559529422 | 2.60E-08 | 1 | 3.959916397 | up |
| Com_4426_neg | Ginsenoside Ro | C48 H76 O19 | 956.50175 | 3.552409138 | 2.56E-10 | 1 | 3.823873851 | up |
| Com_1283_neg | 3-(acetamidomethylidene)-2-(hydroxymethyl)succinic acid | C8 H11 N O6 | 217.05829 | 3.469894786 | 1.75E-07 | 0.99305556 | 3.581953448 | up |
| Com_156_neg | 4-Phenolsulfonic acid | C6 H6 O4 S | 173.99861 | 3.357909949 | 1.74E-05 | 0.9375 | 3.751644755 | up |
| Com_559_neg | Daidzein | C15 H10 O4 | 254.05767 | 3.338270717 | 1.25E-09 | 1 | 3.499205027 | up |
| Com_4280_neg | Baicalin | C21 H18 O11 | 446.08484 | 3.2131398 | 5.56E-08 | 1 | 3.244455336 | up |
| Com_4041_neg | Glycitin | C22 H22 O10 | 446.12099 | 3.078329785 | 3.45E-06 | 0.99305556 | 2.919911448 | up |
| Com_7079_neg | Rutin | C27 H30 O16 | 610.15341 | 2.897874825 | 1.19E-06 | 1 | 2.796683983 | up |
| Com_2133_neg | gamma-Glutamyl-gamma-glutamyl-S-methylcysteine | C14 H23 N3 O8 S | 393.12051 | 2.859981661 | 0.00292301 | 0.86805556 | 2.239019618 | up |
| Com_3405_neg | 3,5-Dihydroxy-2-(4-hydroxyphenyl)-4-oxo-3,4-dihydro-  2H-chromen-7-yl hexopyranoside | C21 H22 O11 | 450.11608 | 2.822775075 | 5.37E-05 | 0.92361111 | 2.969279494 | up |
| Com_8213_neg | N-[(2E)-3-(4-Hydroxyphenyl)-2-propenoyl]tryptophan | C20 H18 N2 O4 | 350.12828 | 2.776201243 | 7.02E-09 | 1 | 2.873944809 | up |
| Com_2543_neg | Alanyl-N-(6-amino-2-pyridinyl)-alpha-glutamine | C13 H19 N5 O4 | 309.14223 | 2.733400439 | 0.00207595 | 0.95833333 | 1.895984897 | up |
| Com_7328_neg | Araloside A | C47 H74 O18 | 926.49019 | 2.720958302 | 1.08E-07 | 1 | 2.737302258 | up |
| Com_622_neg | L-Cystine | C6 H12 N2 O4 S2 | 240.02366 | 2.613235067 | 0.002035 | 0.84722222 | 2.389621033 | up |
| Com_5393_neg | Quercitrin | C21 H20 O11 | 448.10046 | 2.52495875 | 5.59E-05 | 0.91666667 | 2.541795571 | up |
| Com_869_neg | Phenol | C6 H6 O | 94.04178 | 2.51006142 | 0.00061963 | 0.88888889 | 2.156506005 | up |
| Com_219_neg | Gluconic acid | C6 H12 O7 | 196.05815 | 2.477216003 | 0.00020675 | 0.86805556 | 2.748676893 | up |
| Com_5545_neg | 3-O-[(2S,3R,4R)-3,4-Dihydroxy-4-(hydroxymethyl)tetrahydro-  2-furanyl]-beta-D-xylopyranosyl-(1->4)-6-deoxy-alpha-L-  mannopyranosyl-(1->2)-1-O-[(2beta,3beta,16alpha)-3-(beta-D-  glucopyranosyloxy)-2,16,24-trihydroxy-28-oxoolean-12-en-28-  yl]-alpha-L-arabinopyranose | C57 H92 O27 | 1208.58267 | 2.452349341 | 3.97E-08 | 0.99305556 | 2.586994929 | up |
| Com_8188_neg | N-Phenylacetylglutamic acid | C13 H15 N O5 | 265.09471 | 2.448347526 | 6.33E-06 | 0.95833333 | 2.512777244 | up |
| Com_7680_neg | Escin IB | C55 H86 O24 | 1130.55166 | 2.343877967 | 1.53E-07 | 0.99305556 | 2.366335097 | up |
| Com_455_neg | methyl alpha-D-mannoside | C7 H14 O6 | 194.07891 | 2.304811538 | 6.42E-05 | 0.94444444 | 2.148220353 | up |
| Com_9879_neg | LTF4 | C28 H44 N2 O8 S | 568.28096 | 2.302763031 | 1.68E-08 | 0.99305556 | 2.376828441 | up |
| Com_209_neg | THREONIC ACID, L- | C4 H8 O5 | 136.03707 | 2.273405977 | 0.00043363 | 0.875 | 2.318618337 | up |
| Com_1242_neg | 2-[(2Z)-2-({4-[(3-Aminopropyl)amino]butyl}imino)-2-  hydroxyethyl]-2-hydroxysuccinic acid | C13 H25 N3 O6 | 319.17416 | 2.106097298 | 7.18E-05 | 0.97222222 | 1.903598665 | up |
| Com_2215_neg | Malonic acid | C3 H4 O4 | 104.01084 | 2.026733431 | 0.00112293 | 0.82638889 | 1.851693685 | up |
| Com_599_neg | epsilon-(gamma-Glutamyl)-lysine | C11 H21 N3 O5 | 275.14784 | 2.01529442 | 0.00234456 | 0.88888889 | 1.479453202 | up |
| Com_1563_neg | soyasapogenol B 3-O-beta-glucuronide | C36 H58 O9 | 634.40939 | 2.010593463 | 3.05E-06 | 0.95833333 | 2.279105428 | up |
| Com_151_neg | L-Histidine | C6 H9 N3 O2 | 155.06948 | 1.981334737 | 0.00209614 | 0.82638889 | 1.622757838 | up |
| Com_360_neg | 2'-Deoxyuridine | C9 H12 N2 O5 | 228.07442 | 1.947403351 | 0.00016747 | 0.91666667 | 2.042037211 | up |
| Com_257_neg | L-Aspartic acid | C4 H7 N O4 | 133.03744 | 1.898455397 | 0.00161854 | 0.90277778 | 1.632103143 | up |
| Com_9679_neg | 6-Deoxy-alpha-L-mannopyranosyl-(1->3)-beta-D-  xylopyranosyl-(1->4)-6-deoxy-alpha-L-mannopyranosyl-  (1->2)-6-deoxy-1-O-[(2beta,3beta,16alpha,17xi)-3-(beta-D-  glucopyranosyloxy)-2,16,23-trihydroxy-28-oxoolean-  12-en-28-yl]-beta-D-galactopyranose | C59 H96 O27 | 1236.6149 | 1.843365409 | 3.66E-05 | 0.93055556 | 1.849528618 | up |
| Com_1592_neg | (Z)-N-[(4Z,7Z,10Z,13Z,16Z,19Z)-1-Hydroxy-4,7,10,13,16,19-  docosahexaen-1-ylidene]-L-phenylalanine | C31 H41 N O3 | 475.30819 | 1.790019308 | 0.01460009 | 0.78472222 | 1.543691646 | up |
| Com_9823_neg | (3beta,16alpha)-16-hydroxy-30-oxo-13,28-epoxyoleanan-3-  yl beta-D-glucopyranosyl-(1->2)-[beta-D-glucopyranosyl-(1->3)  -[beta-D-xylopyranosyl-(1->2)]-beta-D-glucopyranosyl-(1->4)]  -alpha-L-arabinopyranoside | C58 H94 O27 | 1222.60133 | 1.78563927 | 7.85E-08 | 0.97916667 | 1.89070314 | up |
| Com_3773_neg | 4-Pyridoxate | C8 H9 N O4 | 183.05306 | 1.746243733 | 0.00699134 | 0.77083333 | 1.561158561 | up |
| Com_9462_neg | Dihydronaringenin-O-sulphate | C15 H14 O8 S | 354.04103 | 1.717737824 | 0.00240787 | 0.81944444 | 1.618763643 | up |
| Com_306_neg | 2'-Deoxyinosine | C10 H12 N4 O4 | 252.08565 | 1.665452289 | 0.00181883 | 0.86111111 | 1.764741008 | up |
| Com_7426_neg | 2-Phenylethyl 6-O-beta-D-xylopyranosyl-beta-D-glucopyranoside | C19 H28 O10 | 416.16814 | 1.630749322 | 0.00974131 | 0.77083333 | 1.54294659 | up |
| Com_419_neg | Threonine | C4 H9 N O3 | 119.0582 | 1.628602523 | 0.00250956 | 0.86111111 | 1.372151407 | up |
| Com_7133_neg | (1S,6S)-6-Aminooctahydro-1-indolizinyl acetate | C10 H18 N2 O2 | 198.13675 | 1.583810885 | 4.94E-05 | 0.92361111 | 1.743339697 | up |
| Com_665_neg | L-(+)-Valine | C5 H11 N O2 | 117.07889 | 1.58054876 | 0.02452352 | 0.75 | 1.037104978 | up |
| Com_102_neg | L-(-)-methionine | C5 H11 N O2 S | 149.05104 | 1.535629616 | 0.00739613 | 0.80555556 | 1.301506241 | up |
| Com_4773_neg | 3-O-(3-Methylbutanoyl)-beta-D-fructofuranosyl 2-O-acetyl-3-  O-(2-methylbutanoyl)-alpha-D-glucopyranoside | C24 H40 O14 | 552.24458 | 1.446910997 | 0.00134906 | 0.86111111 | 1.779341269 | up |
| Com_2716_neg | (3beta)-3-Hydroxy-23-(sulfooxy)olean-12-en-28-oic acid | C30 H48 O7 S | 552.31125 | 1.424847738 | 0.00131151 | 0.84722222 | 1.685040218 | up |
| Com_6779_neg | 1-O-(Hydroxy{[(6Z,10E,14E)-3,7,11,15,19-pentamethyl-  6,10,14,18-icosatetraen-1-yl]oxy}phosphoryl)-beta-D-xylopyranose | C30 H53 O8 P | 572.34655 | 1.400669109 | 1.47E-05 | 0.94444444 | 1.58419482 | up |
| Com_1046_neg | CP 47,497-C8-Homolog C-8-hydroxy metabolite | C22 H36 O3 | 348.26621 | 1.383256742 | 0.00329172 | 0.84027778 | 1.460256891 | up |
| Com_3076_neg | 6-Aminopenicillanic acid | C8 H12 N2 O3 S | 216.05678 | 1.361914288 | 0.03251319 | 0.73611111 | 1.250213202 | up |
| Com_71_neg | 5-Aminovaleric acid | C5 H11 N O2 | 117.07892 | 1.358722309 | 0.02025004 | 0.76388889 | 1.058367025 | up |
| Com_4182_neg | 1-tetradecanoyl-2-[(5Z,8Z,11Z,14Z)-eicosatetraenoyl]  -sn-glycero-3-phosphocholine | C42 H76 N O8 P | 753.53364 | 1.29250154 | 0.00011797 | 0.94444444 | 1.430873829 | up |
| Com_4463_neg | 6'-oxoparomamine | C12 H23 N3 O7 | 321.15364 | 1.265921115 | 0.0057903 | 0.84722222 | 1.040309375 | up |
| Com_2418_neg | GLY-MET | C7 H14 N2 O3 S | 206.07235 | 1.262703059 | 0.00121401 | 0.88194444 | 1.213366467 | up |
| Com_8455_neg | lipoic acid | C8 H14 O2 S2 | 206.0425 | 1.253367694 | 0.00819326 | 0.78472222 | 1.161105571 | up |
| Com_33_neg | Arachidonic acid | C20 H32 O2 | 304.23991 | 1.235175715 | 0.01166537 | 0.79166667 | 2.601864752 | up |
| Com_113_neg | L-Glutamic acid | C5 H9 N O4 | 147.05313 | 1.207664794 | 0.00288692 | 0.86111111 | 1.058096415 | up |
| Com_1432_neg | Dihydrogeranylgeranyl diphosphate | C20 H38 O7 P2 | 452.20743 | 1.206811893 | 0.03410282 | 0.75 | 1.371204269 | up |
| Com_62_neg | L-Tyrosine | C9 H11 N O3 | 181.07386 | 1.193132361 | 0.0236094 | 0.75694444 | 1.184760461 | up |
| Com_3647_neg | (20S)-17,20-Dihydroxypregn-4-en-3-one | C21 H32 O3 | 332.23504 | 1.19281564 | 0.01427455 | 0.75694444 | 1.090228059 | up |
| Com_3049_neg | Oxadixyl | C14 H18 N2 O4 | 278.12645 | 1.189637923 | 0.00144271 | 0.86111111 | 1.174385928 | up |
| Com_9880_neg | haplophytine | C37 H40 N4 O7 | 652.29164 | 1.171974436 | 0.0001504 | 0.91666667 | 1.21593861 | up |
| Com_2978_neg | Jervine | C27 H39 N O3 | 425.29286 | 1.170062978 | 0.02562369 | 0.77777778 | 1.05123982 | up |
| Com_6120_neg | Prunin | C21 H22 O10 | 434.1211 | 1.161053893 | 0.00763566 | 0.79166667 | 1.306602172 | up |
| Com_9728_neg | (5alpha,11beta,17beta)-9-Fluoro-11,17-dihydroxy-17  -methylandrostan-3-one | C20 H31 F O3 | 338.22621 | 1.088193506 | 0.00370899 | 0.86805556 | 1.143726956 | up |
| Com_4831_neg | (3beta,9beta,17xi,22S)-3-(beta-D-Glucopyranosyloxy)-  26-oxo-22,26-epoxy-9,19-cyclolanost-24-en-28-oic acid | C36 H54 O10 | 646.37294 | 1.086071605 | 0.04510992 | 0.70833333 | 1.142269566 | up |
| Com_9074_neg | (1S,2R,3S,5S,6S,16E,18E,20R,21S)-11-Chloro-21-hydroxy  -12,20-dimethoxy-2,5,9,16-tetramethyl-8,23-dioxo-4,24-  dioxa-9,22-diazatetracyclo[19.3.1.1~10,14~.0~3,5~]  hexacosa-10(26),11,13,16,18-pentaen-6-yl 2-methylpropanoate | C32 H43 Cl N2 O9 | 634.26437 | 1.082528604 | 0.02405519 | 0.74305556 | 1.101076674 | up |
| Com_547_neg | Valylproline | C10 H18 N2 O3 | 214.13155 | 1.079570242 | 0.0012961 | 0.86111111 | 1.146444642 | up |
| Com_1022_neg | THC | C21 H30 O2 | 314.22428 | 1.061295937 | 0.01407282 | 0.78472222 | 1.368012512 | up |
| Com_1812_neg | S-methylglutathione | C11 H19 N3 O6 S | 321.10029 | 1.049784644 | 0.0030508 | 0.82638889 | 1.152530555 | up |
| Com_174_neg | Uridine | C9 H12 N2 O6 | 244.06932 | 1.011698184 | 0.00469888 | 0.82638889 | 1.011359169 | up |
| Com_5609_neg | apronalide | C9 H16 N2 O2 | 184.12111 | -1.003583046 | 0.04688745 | 0.69444444 | 1.034055779 | down |
| Com_9865_neg | sufotidine | C20 H31 N5 O3 S | 421.21275 | -1.010945644 | 0.00426801 | 0.8125 | 1.214174133 | down |
| Com_7370_neg | tenivastatin | C25 H40 O6 | 436.28239 | -1.012433712 | 0.01523125 | 0.79166667 | 1.207311817 | down |
| Com_6546_neg | 4-Chloroaniline | C6 H6 Cl N | 127.01881 | -1.013978984 | 0.01466499 | 0.77083333 | 1.082190203 | down |
| Com_7940_neg | Alosetron | C17 H18 N4 O | 294.1466 | -1.018131893 | 0.00329144 | 0.83333333 | 1.042141265 | down |
| Com_7407_neg | 3-O-(3-Methylbutanoyl)-beta-D-fructofuranosyl 2-O-  acetyl-3-O-(2-methylbutanoyl)-4-O-(3-methylbutanoyl)  -alpha-D-glucopyranoside | C29 H48 O15 | 636.30008 | -1.022129451 | 0.01652704 | 0.74305556 | 1.046502909 | down |
| Com_3786_neg | 3,3-Dimethyl-5-oxo-5-[4-(2-pyrimidinyl)piperazino]  pentanoic acid | C15 H22 N4 O3 | 306.16772 | -1.023374202 | 0.01145394 | 0.76388889 | 1.152222894 | down |
| Com_8854_neg | 4-Chlorophenol | C6 H5 Cl O | 128.00292 | -1.024668704 | 6.64E-06 | 0.96527778 | 1.085200834 | down |
| Com_87_neg | Diethylpyrocarbonate | C6 H10 O5 | 162.05274 | -1.027111016 | 0.00068801 | 0.86805556 | 1.22551753 | down |
| Com_1303_neg | (5-Benzyl-3,6-dioxo-2-piperazinyl)acetic acid | C13 H14 N2 O4 | 262.09513 | -1.030026423 | 0.04605452 | 0.70833333 | 1.093355355 | down |
| Com_6586_neg | 5722 | C23 H36 O5 | 392.25517 | -1.031413595 | 0.00122864 | 0.875 | 1.218116732 | down |
| Com_8613_neg | Tetrandrine | C38 H42 N2 O6 | 622.30417 | -1.035531073 | 0.00351445 | 0.8125 | 1.05593858 | down |
| Com_3879_neg | 7-Mercaptoheptanoylthreonine | C11 H21 N O4 S | 263.11886 | -1.045039002 | 0.00117414 | 0.85416667 | 1.195478541 | down |
| Com_1284_neg | Lithocholic acid taurine conjugate | C26 H45 N O5 S | 483.30188 | -1.045561574 | 0.02441712 | 0.77083333 | 1.331703616 | down |
| Com_3081_neg | THREO-SPHINGOSINE, (-)- | C18 H37 N O2 | 299.28234 | -1.046843986 | 0.00572434 | 0.84722222 | 1.022993449 | down |
| Com_41_neg | Succinic acid | C4 H6 O4 | 118.02653 | -1.05963363 | 0.00736223 | 0.84027778 | 1.293743631 | down |
| Com_5214_neg | Linsitinib | C26 H23 N5 O | 421.19181 | -1.06177724 | 0.00331659 | 0.83333333 | 1.127019386 | down |
| Com_9145_neg | Pefloxacin | C17 H20 F N3 O3 | 333.14779 | -1.06448311 | 4.44E-05 | 0.95138889 | 1.144398094 | down |
| Com_481_neg | lactide | C6 H8 O4 | 144.0422 | -1.065639279 | 0.00189209 | 0.86111111 | 1.349699298 | down |
| Com_1057_neg | persin | C23 H40 O4 | 380.29257 | -1.068646601 | 0.02713179 | 0.75694444 | 1.232785801 | down |
| Com_6273_neg | OXOGESTONE PHENPROPIONATE | C29 H38 O3 | 434.28411 | -1.068704311 | 0.01241565 | 0.77777778 | 1.183311688 | down |
| Com_6375_neg | amquinate | C18 H24 N2 O3 | 316.17862 | -1.071896947 | 0.00793126 | 0.78472222 | 1.273457838 | down |
| Com_8245_neg | (4E)-5-Hydroxy-4-{(2E,4E,6R)-1-hydroxy-6-[(3R,4R,6R)  -6-hydroxy-1,4,8-trimethyl-2,9-dioxabicyclo[3.3.1]non-7-  en-3-yl]-4-methyl-2,4-heptadien-1-ylidene}-2,4-dihydro-  3H-pyrrol-3-one | C22 H29 N O6 | 403.20056 | -1.074500458 | 7.53E-06 | 0.93055556 | 1.081504919 | down |
| Com_9773_neg | GLYCERYL 1,3-DINITRATE | C3 H6 N2 O7 | 182.01801 | -1.078533513 | 0.00279248 | 0.86805556 | 1.131712606 | down |
| Com_10019_neg | [(1S,4S,5S,6R,9S,10R,12R,14R)-4-(Benzoyloxy)-5,6-  dihydroxy-3,11,11,14-tetramethyl-15-oxotetracyclo  [7.5.1.0~1,5~.0~10,12~]pentadeca-2,7-dien-7-yl]methyl  benzoate | C34 H36 O7 | 556.24444 | -1.078565553 | 0.00062983 | 0.90277778 | 1.17261833 | down |
| Com_6374_neg | napelline | C22 H33 N O3 | 359.2462 | -1.081151322 | 0.00014353 | 0.89583333 | 1.096794622 | down |
| Com_4464_neg | Metolachlor ESA | C15 H23 N O5 S | 329.1296 | -1.084797255 | 0.00015835 | 0.93055556 | 1.233382083 | down |
| Com_10392_neg | Leukotriene E3 | C23 H39 N O5 S | 441.25484 | -1.08884759 | 0.00491907 | 0.80555556 | 1.126343429 | down |
| Com_6882_neg | Methyl (1aR,2E,4S,5S,5aS,8aR,9E,10aS)-4-acetoxy-5-  {[(2R,3S)-3-acetoxy-2-hydroxy-2-methylbutanoyl]oxy}-  10-methyl-6-methylene-7-oxo-1a,4,5,5a,6,7,8a,10a-  octahydrooxireno[7,8]cyclodeca[1,2-b]furan-3-carboxylate | C25 H30 O12 | 522.17193 | -1.090922141 | 0.00103725 | 0.86111111 | 1.219211767 | down |
| Com_2777_neg | Tomelukast | C16 H22 N4 O3 | 318.16912 | -1.091747978 | 0.01898986 | 0.74305556 | 1.403964043 | down |
| Com_4991_neg | (3S,6S)-3-(4-Hydroxybenzyl)-6-(hydroxymethyl)  -2,5-piperazinedione | C12 H14 N2 O4 | 250.09514 | -1.100715553 | 0.00162506 | 0.8125 | 1.167105539 | down |
| Com_826_neg | Methyl undecenate | C12 H22 O2 | 198.1619 | -1.103337441 | 0.00208813 | 0.83333333 | 1.031616537 | down |
| Com_693_neg | (3alpha,5beta,7alpha,12beta)-7,12-Dihydroxy-24  -oxo-24-[(2-sulfoethyl)amino]cholane-3-sulfonic acid | C26 H45 N O9 S2 | 579.25314 | -1.107205293 | 0.02546879 | 0.70138889 | 1.28573025 | down |
| Com_7053_neg | cefluprenam | C20 H25 F N8 O6 S2 | 556.13333 | -1.111173034 | 0.00141893 | 0.84722222 | 1.213764482 | down |
| Com_5353_neg | PGF2a ethanolamide | C22 H39 N O5 | 397.28286 | -1.118545167 | 0.00527257 | 0.81944444 | 1.187939422 | down |
| Com_9090_neg | Spenolimycin | C15 H26 N2 O7 | 346.17277 | -1.125316822 | 0.00126838 | 0.84027778 | 1.239582319 | down |
| Com_5125_neg | Homovanillic acid | C9 H10 O4 | 182.05784 | -1.131069175 | 9.88E-05 | 0.89583333 | 1.166560495 | down |
| Com_310_neg | N-Arachidonoyl taurine | C22 H37 N O4 S | 411.24416 | -1.132541734 | 0.0001226 | 0.91666667 | 1.126496448 | down |
| Com_9171_neg | Androsterone glucuronide | C25 H38 O8 | 466.25711 | -1.133527788 | 0.00090293 | 0.85416667 | 1.148028091 | down |
| Com_136_neg | DL-Lactic Acid | C3 H6 O3 | 90.03165 | -1.134407073 | 0.02490873 | 0.74305556 | 1.3339222 | down |
| Com_7262_neg | undecenal | C11 H20 O | 168.15136 | -1.141231442 | 0.01213409 | 0.75694444 | 1.161689121 | down |
| Com_926_neg | AMADINONE ACETATE | C22 H27 Cl O4 | 390.16062 | -1.14785889 | 8.32E-05 | 0.90972222 | 1.313332575 | down |
| Com_3004_neg | 4-(5,6-Dihydroxy-6-methyl-1-hepten-2-yl)-1-  methyl-1,2-cyclohexanediol | C15 H28 O4 | 272.19853 | -1.150977882 | 0.00375701 | 0.80555556 | 1.383707791 | down |
| Com_2500_neg | N-Acetyl-DL-tryptophan | C13 H14 N2 O3 | 246.10023 | -1.156139097 | 0.00027158 | 0.92361111 | 1.184591899 | down |
| Com_1474_neg | 8-hydroxy-7-methylguanine | C6 H7 N5 O2 | 181.05997 | -1.156240826 | 0.00168346 | 0.875 | 1.279639484 | down |
| Com_4826_neg | (2S)-2-[(1-Carboxyethyl)amino]-4-(dimethylsulfonio)butanoate | C9 H17 N O4 S | 235.08767 | -1.15681311 | 5.22E-05 | 0.94444444 | 1.2631237 | down |
| Com_6219_neg | Cocarboxylase | C12 H18 N4 O7 P2 S | 424.03581 | -1.161499376 | 0.00010704 | 0.93055556 | 1.227714341 | down |
| Com_5367_neg | (-)-Epothilone A | C26 H39 N O6 S | 493.24938 | -1.166086341 | 0.01920957 | 0.75694444 | 1.327052387 | down |
| Com_2878_neg | bentazepam | C17 H16 N2 O S | 296.09911 | -1.166216157 | 0.00237928 | 0.84027778 | 1.632384659 | down |
| Com_4415_neg | Methyl Jasmonate | C13 H20 O3 | 224.14109 | -1.175689952 | 0.00380716 | 0.8125 | 1.187223842 | down |
| Com_5106_neg | 4-Aminobenzoic acid | C7 H7 N O2 | 137.04764 | -1.176292788 | 1.19E-07 | 0.99305556 | 1.21257328 | down |
| Com_4910_neg | Pivagabine | C9 H17 N O3 | 187.12078 | -1.1788123 | 0.00080141 | 0.86111111 | 1.194436817 | down |
| Com_2800_neg | LysoPC(18:4(6Z,9Z,12Z,15Z)) | C26 H46 N O7 P | 515.2996 | -1.185790679 | 0.03296445 | 0.75694444 | 1.292042444 | down |
| Com_1311_neg | UDP-N-acetylglucosamine | C17 H27 N3 O17 P2 | 607.08167 | -1.187570609 | 0.00279533 | 0.83333333 | 2.47725939 | down |
| Com_5961_neg | Phthalocyanine | C32 H18 N8 | 514.16371 | -1.189074521 | 0.00797585 | 0.82638889 | 1.291819196 | down |
| Com_7502_neg | Methyl 3-isobutyryl-4-methyl-1,5-bis(3-methyl-2  -buten-1-yl)-4-(4-methyl-3-penten-1-yl)-2-  oxocyclohexanecarboxylate | C29 H46 O4 | 458.33934 | -1.202701214 | 0.00016723 | 0.89583333 | 1.392918951 | down |
| Com_3596_neg | 6-Methoxy-3-(1,3-thiazol-2-yl)-1H-indole | C12 H10 N2 O S | 230.05122 | -1.219366398 | 0.00756439 | 0.79861111 | 1.346173542 | down |
| Com_10193_neg | jamaicamide C | C27 H39 Cl N2 O4 | 490.25895 | -1.220133878 | 0.00497635 | 0.79861111 | 1.267054714 | down |
| Com_421_neg | Pyruvic acid | C3 H4 O3 | 88.01601 | -1.221571568 | 0.00220113 | 0.85416667 | 1.57687956 | down |
| Com_6645_neg | tetrahydrocortisol | C21 H34 O5 | 366.24062 | -1.22317763 | 2.03E-05 | 0.9375 | 1.286765766 | down |
| Com_1990_neg | (Z)-N-[(2S)-2-{(Z)-[(2S,3S,6R,7R)-2,3-Diamino-  1,6,7,8-tetrahydroxy-8-{1-[hydroxy(imino)methyl]-  2-imino-4-imidazolidinyl}octylidene]amino}-1  -hydroxypropylidene]-L-valine | C20 H38 N8 O8 | 518.28078 | -1.223716418 | 1.04E-06 | 0.96527778 | 1.267126978 | down |
| Com_4449_neg | DIETHAMINE | C11 H13 F3 N4 O4 | 322.08928 | -1.227392459 | 0.0495383 | 0.6875 | 1.200580166 | down |
| Com_2005_neg | disobutamide | C23 H38 Cl N3 O | 407.27034 | -1.234969382 | 6.71E-05 | 0.9375 | 1.315889419 | down |
| Com_4965_neg | (1Z)-N-[(3R,4R,5R,6R)-6-{2-[(2R,3S,4R,5R)-3,4-  Dihydroxy-5-(4-hydroxy-2-oxo-1(2H)-pyrimidinyl)  tetrahydro-2-furanyl]-2-hydroxyethyl}-2,4,5-  trihydroxytetrahydro-2H-pyran-3-yl]ethanimidic  acid (non-preferred name) | C17 H25 N3 O11 | 447.14879 | -1.239372266 | 0.00033786 | 0.89583333 | 1.336903173 | down |
| Com_8300_neg | Ulimorelin | C30 H39 F N4 O4 | 538.29304 | -1.254432365 | 0.0007486 | 0.89583333 | 1.476326512 | down |
| Com_6625_neg | Talmapimod | C27 H30 Cl F N4 O3 | 512.19791 | -1.254944138 | 0.00024313 | 0.89583333 | 1.334991792 | down |
| Com_529_neg | Azelnidipine | C33 H34 N4 O6 | 582.24821 | -1.256263889 | 0.00018805 | 0.90972222 | 1.335709219 | down |
| Com_2759_neg | N-Sulfanilyl-3,4-xylamide | C15 H16 N2 O3 S | 304.08804 | -1.26156653 | 0.00762131 | 0.78472222 | 1.424979709 | down |
| Com_3365_neg | Zolpidem | C19 H21 N3 O | 307.16876 | -1.262141277 | 0.00015515 | 0.9375 | 1.434088256 | down |
| Com_239_neg | Arachidic acid | C20 H40 O2 | 312.30266 | -1.285495669 | 0.01088923 | 0.80555556 | 1.241389087 | down |
| Com_4629_neg | Caproylglycine | C12 H23 N O3 | 229.16766 | -1.290730066 | 0.00058722 | 0.88194444 | 1.345358223 | down |
| Com_10100_neg | 2-{[(3alpha,5beta,7alpha,8xi,12alpha)-3,7,12-  Trihydroxy-24-oxocholan-24-yl]amino}  ethyl hydrogen sulfate | C26 H45 N O8 S | 531.28645 | -1.292472931 | 0.00010143 | 0.91666667 | 1.450977837 | down |
| Com_3523_neg | Pyrogallol | C6 H6 O3 | 126.03163 | -1.297795279 | 3.27E-06 | 1 | 1.287835818 | down |
| Com_4789_neg | 10-(4-Sulfophenyl) decanoic acid | C16 H24 O5 S | 328.13441 | -1.300646899 | 9.33E-06 | 0.95138889 | 1.380991828 | down |
| Com_6906_neg | Selenocystathionine | C7 H14 N2 O4 Se | 270.01158 | -1.31010107 | 0.00270699 | 0.86111111 | 1.601008807 | down |
| Com_2904_neg | Ascorbyl palmitate | C22 H38 O7 | 414.26344 | -1.313099698 | 0.00018528 | 0.91666667 | 1.53918129 | down |
| Com_4960_neg | 5-Hydroxy-2,2-dimethyl-7,10-bis(3-methyl-2  -buten-1-yl)-8-(2,4,5-trihydroxyphenyl)-  2H,6H-pyrano[3,2-g]chromen-6-one | C30 H32 O7 | 504.21382 | -1.31434744 | 0.00409807 | 0.80555556 | 1.393318427 | down |
| Com_1897_neg | 2-(alpha-D-mannosyl)-D-glyceric acid | C9 H16 O9 | 268.07912 | -1.318078137 | 0.00763099 | 0.81944444 | 1.341739584 | down |
| Com_6819_neg | PALGLY | C18 H35 N O3 | 313.26154 | -1.337088432 | 0.00023218 | 0.89583333 | 1.581650686 | down |
| Com_6629_neg | fosravuconazole | C23 H20 F2 N5 O5 P S | 547.08668 | -1.338652917 | 0.03214512 | 0.76388889 | 1.748025609 | down |
| Com_516_neg | Xylitol | C5 H12 O5 | 152.06842 | -1.34139273 | 0.00036235 | 0.85416667 | 1.289151211 | down |
| Com_9938_neg | epothilone C | C26 H39 N O5 S | 477.25474 | -1.347337009 | 8.74E-05 | 0.95833333 | 1.610783311 | down |
| Com_1768_neg | Indole-3-pyruvic acid | C11 H9 N O3 | 203.05822 | -1.349080725 | 0.00087607 | 0.89583333 | 1.536665021 | down |
| Com_6434_neg | PYROQUILON | C11 H11 N O | 173.08407 | -1.352244127 | 0.00299559 | 0.81944444 | 1.878061867 | down |
| Com_4981_neg | (1S,4R,5R,6R,6aS,9S,9aE,10aR)-1,5-Dihydroxy  -9-(hydroxymethyl)-3-isopropyl-6,10a-dimethyl  -1,2,4,5,6,6a,7,8,9,10a-decahydrodicyclopenta  [a,d][8]annulen-4-yl alpha-D-glucopyranoside | C26 H42 O9 | 498.28294 | -1.35677218 | 0.00034889 | 0.89583333 | 1.328272231 | down |
| Com_2705_neg | PIPEROPHOS | C14 H28 N O3 P S2 | 353.12404 | -1.357146028 | 0.0005266 | 0.88194444 | 1.437856634 | down |
| Com_3765_neg | 12-O-Tetradecanoylphorbol-13-acetate | C36 H56 O8 | 616.39908 | -1.360206832 | 0.00043871 | 0.875 | 1.550795619 | down |
| Com_9650_neg | Sarpogrelate | C24 H31 N O6 | 429.21602 | -1.362381113 | 4.28E-05 | 0.91666667 | 1.377986683 | down |
| Com_3114_neg | 7369 | C18 H27 N O2 S | 321.17731 | -1.367970372 | 8.12E-05 | 0.9375 | 1.356448171 | down |
| Com_232_neg | Bis(2-ethylhexyl) phthalate | C24 H38 O4 | 390.27695 | -1.377822701 | 0.01349811 | 0.77777778 | 1.498460394 | down |
| Com_10061_neg | Roflumilast | C17 H14 Cl2 F2 N2 O3 | 402.03387 | -1.411739314 | 0.01306418 | 0.82638889 | 1.750890075 | down |
| Com_5576_neg | Loganin | C17 H26 O10 | 390.15262 | -1.416016042 | 2.14E-05 | 0.93055556 | 1.502217254 | down |
| Com_3780_neg | QV1MVO1R | C10 H11 N O4 | 209.0687 | -1.43160717 | 1.97E-07 | 0.99305556 | 1.51961409 | down |
| Com_11_neg | Taurochenodeoxycholic acid | C26 H45 N O6 S | 499.29605 | -1.440358279 | 8.83E-06 | 0.93055556 | 1.505486808 | down |
| Com_9210_neg | 2-methoxyestrone 3-glucosiduronic acid | C25 H32 O9 | 476.2058 | -1.459458328 | 0.00199609 | 0.85416667 | 1.644742682 | down |
| Com_5773_neg | Cuauhtemone | C15 H24 O3 | 252.17239 | -1.461468267 | 8.08E-05 | 0.92361111 | 1.510603916 | down |
| Com_10095_neg | 7-Sulfocholic acid | C24 H40 O8 S | 488.24391 | -1.467250859 | 0.01010141 | 0.82638889 | 1.168738331 | down |
| Com_7456_neg | Deserpidine | C32 H38 N2 O8 | 578.26423 | -1.481807654 | 0.01544259 | 0.75 | 1.478837679 | down |
| Com_4919_neg | trehalose 6-phosphate | C12 H23 O14 P | 422.08229 | -1.496510717 | 7.31E-07 | 0.97222222 | 1.536718565 | down |
| Com_471_neg | D-(+)-Maltose | C12 H22 O11 | 388.12167 | -1.50367863 | 0.0003798 | 0.88888889 | 1.677139109 | down |
| Com_3480_neg | (3alpha,5beta,7alpha,8xi,9xi,12alpha,14xi,25R)  -7-(beta-D-Galactopyranosyloxy)-3,12-  dihydroxycholestan-27-yl acetate | C35 H60 O10 | 640.42016 | -1.50643815 | 0.00190559 | 0.86111111 | 1.498512616 | down |
| Com_10257_neg | Retinyl beta-glucuronide | C26 H38 O7 | 462.26213 | -1.512077616 | 0.0001398 | 0.90277778 | 1.535788696 | down |
| Com_9162_neg | 4-{[3-(Hydroxymethyl)phenyl]amino}-N-  (isopropylcarbamoyl)-3-pyridinesulfonamide | C16 H20 N4 O4 S | 364.11934 | -1.518942974 | 7.33E-06 | 0.97222222 | 1.648494373 | down |
| Com_8821_neg | Mucronine B | C28 H36 N4 O4 | 492.27239 | -1.525320494 | 8.63E-05 | 0.96527778 | 1.377520758 | down |
| Com_6364_neg | 4917 | C17 H18 N4 O3 S | 358.10861 | -1.540457044 | 3.04E-06 | 0.97222222 | 1.601213087 | down |
| Com_3814_neg | N-Acetylneuraminic acid | C11 H19 N O9 | 309.10337 | -1.540687527 | 0.00020414 | 0.88888889 | 1.743108148 | down |
| Com_10206_neg | 4-HYDROXYCARVEDILOL | C24 H26 N2 O5 | 422.18623 | -1.547898886 | 0.00025506 | 0.90972222 | 1.577176666 | down |
| Com_4973_neg | (1S,2R,5S)-2-Isopropyl-5-methylcyclohexyl 3-  oxobutanoate | C14 H24 O3 | 240.17231 | -1.561227182 | 1.75E-06 | 0.95138889 | 1.604083238 | down |
| Com_448_neg | N-Acetylanthranilic acid | C9 H9 N O3 | 179.05823 | -1.564979114 | 7.03E-08 | 1 | 1.632076417 | down |
| Com_383_neg | Levulinic acid | C5 H8 O3 | 116.04729 | -1.570889035 | 1.29E-05 | 0.95138889 | 1.657902363 | down |
| Com_8282_neg | 1-hexadecanal | C16 H32 O | 240.24522 | -1.58037673 | 8.63E-05 | 0.95138889 | 1.913894555 | down |
| Com_8081_neg | 6-O-Phosphonohex-2-ulofuranose | C6 H13 O9 P | 260.02972 | -1.590560553 | 1.52E-05 | 0.95138889 | 1.545470724 | down |
| Com_2662_neg | Acetanilide | C8 H9 N O | 135.06839 | -1.592746677 | 5.53E-08 | 1 | 1.628436813 | down |
| Com_7553_neg | Chaparrin | C20 H28 O7 | 380.18281 | -1.596319593 | 7.99E-07 | 0.97916667 | 1.639936963 | down |
| Com_5366_neg | Undecylic acid | C11 H22 O2 | 186.16192 | -1.625357594 | 6.28E-07 | 0.98611111 | 1.75272101 | down |
| Com_640_neg | D-(+)-Arabitol | C5 H12 O5 | 152.06844 | -1.644598265 | 1.34E-05 | 1 | 1.818201729 | down |
| Com_2178_neg | phenylamine | C6 H7 N | 93.05781 | -1.659476917 | 1.59E-08 | 1 | 1.703234737 | down |
| Com_2740_neg | Desoxymycin | C21 H41 N7 O11 | 567.28447 | -1.699875603 | 4.28E-06 | 0.97916667 | 1.816349958 | down |
| Com_4635_neg | Rilpivirine | C22 H18 N6 | 366.15802 | -1.702747368 | 0.00066817 | 0.88194444 | 2.131620765 | down |
| Com_935_neg | Tixocortol | C21 H30 O4 S | 378.18652 | -1.710922523 | 4.04E-06 | 0.96527778 | 1.860286278 | down |
| Com_1572_neg | Omeprazole | C17 H19 N3 O3 S | 345.11469 | -1.726218815 | 0.00026468 | 0.88888889 | 2.014647111 | down |
| Com_1701_neg | 13-Hydroxykaur-16-en-18-oic acid | C20 H30 O3 | 318.21928 | -1.754889911 | 0.02633216 | 0.82638889 | 2.322353065 | down |
| Com_2896_neg | (2beta,3beta,5beta,20xi,22R)-2,3,14,20,22,25  -Hexahydroxy-6-oxocholest-7-en-26-al | C27 H42 O8 | 494.2874 | -1.766408393 | 2.35E-05 | 0.9375 | 1.841053338 | down |
| Com_8057_neg | 1-Methyl-3-(1,3-thiazol-2-yl)-1H-indole | C12 H10 N2 S | 214.05646 | -1.77283072 | 5.77E-05 | 0.9375 | 1.928686387 | down |
| Com_7935_neg | Capryloylglycine | C10 H19 N O3 | 201.13647 | -1.791326803 | 0.00024164 | 0.90277778 | 1.714307126 | down |
| Com_3632_neg | paracetamol sulfate | C8 H9 N O5 S | 231.01996 | -1.800645195 | 1.66E-06 | 0.99305556 | 1.853285926 | down |
| Com_2827_neg | Aspirin | C9 H8 O4 | 180.04219 | -1.809720938 | 0.00077993 | 0.84027778 | 1.60717356 | down |
| Com_3499_neg | 4-Hydroxyphenylpyruvic acid | C9 H8 O4 | 180.04223 | -1.822601146 | 0.000711 | 0.84027778 | 1.747144942 | down |
| Com_9649_neg | 3'-dephospho-CoA | C21 H35 N7 O13 P2 S | 687.14846 | -1.858533391 | 8.18E-05 | 0.91666667 | 2.531619913 | down |
| Com_5300_neg | Ethyl Butylacetylaminopropionate | C11 H21 N O3 | 215.1521 | -1.863876408 | 0.00036508 | 0.89583333 | 1.766957611 | down |
| Com_9568_neg | piroximone | C11 H11 N3 O2 | 217.08504 | -1.897794419 | 6.08E-05 | 0.96527778 | 2.101531116 | down |
| Com_5092_neg | picrasin B | C21 H28 O6 | 376.18934 | -1.977814977 | 0.00722524 | 0.88194444 | 1.282472122 | down |
| Com_8523_neg | Myriocin | C21 H39 N O6 | 401.27775 | -2.002797288 | 0.00014473 | 0.91666667 | 1.892480227 | down |
| Com_5241_neg | [(2R,3S,4R,5R)-5-(6-Amino-9H-purin-9-yl)-3,4-  dihydroxytetrahydro-2-furanyl]methyl [(2R,3S,4R,5R)  -3,4,5-trihydroxytetrahydro-2-furanyl]methyl  dihydrogen diphosphate | C15 H23 N5 O14 P2 | 559.07208 | -2.004906284 | 6.74E-06 | 0.95138889 | 2.141510926 | down |
| Com_6494_neg | Indole-3-acetic acid | C10 H9 N O2 | 175.06327 | -2.007720331 | 5.17E-05 | 0.92361111 | 2.002255862 | down |
| Com_1636_neg | taurolithocholic acid sulfate | C26 H45 N O8 S2 | 563.25819 | -2.009295526 | 0.00018865 | 0.88888889 | 2.064434017 | down |
| Com_163_neg | Myristyl sulfate | C14 H30 O4 S | 294.18623 | -2.012858696 | 0.02284295 | 0.875 | 1.103754871 | down |
| Com_7387_neg | Hypericin | C30 H16 O8 | 504.08581 | -2.018699856 | 3.50E-06 | 0.98611111 | 1.930946203 | down |
| Com_3126_neg | 3-(indol-3-yl)-2-oxobutyric acid | C12 H11 N O3 | 217.07376 | -2.041705253 | 1.03E-07 | 0.98611111 | 2.193175511 | down |
| Com_8923_neg | Cys-tyr | C12 H16 N2 O4 S | 284.08299 | -2.089137704 | 4.17E-06 | 0.97222222 | 2.203781314 | down |
| Com_9449_neg | triafungin | C13 H10 N4 | 222.09075 | -2.135406059 | 1.98E-05 | 0.95833333 | 2.087411742 | down |
| Com_6924_neg | (S)-2-methylbutanal | C5 H10 O | 86.07313 | -2.225172132 | 5.13E-09 | 1 | 2.284261712 | down |
| Com_6093_neg | UDP-N-acetyl-alpha-muramoyl-L-alanyl-D-glutamic acid | C28 H43 N5 O23 P2 | 879.18245 | -2.379194225 | 0.00055 | 0.84722222 | 2.052911995 | down |
| Com_5560_neg | Mupirocin | C26 H44 O9 | 500.29903 | -2.653724354 | 0.02459028 | 0.88194444 | 1.26779952 | down |
| Com_8024_neg | Buspirone | C21 H31 N5 O2 | 385.24644 | -2.804180116 | 9.85E-07 | 0.97222222 | 2.82259645 | down |
| Com_7885_neg | Cilostazol | C20 H27 N5 O2 | 369.21522 | -3.330548997 | 1.47E-08 | 1 | 3.315173221 | down |

| **Supplementary Table 15. The significnat differential metabolites in DI contents of SBM20 vs. FM in negative mode (n = 12)** | | | | | | | | |
| --- | --- | --- | --- | --- | --- | --- | --- | --- |
|
| ID | Name_des | Formula | FC | log2FC | Pvalue | AUC | VIP | Up.Down |
| Com_1000_neg | 7-Benzyl-4-[(2-hydroxy-4-methylpentanoyl)  amino]-3-isopropyl-5,8-dioxo-2-oxa-6,9-  diazabicyclo[10.2.2]hexadeca-1(14),12,15-  triene-10-carboxylic acid | C30 H39 N3 O7 | 3.257634366 | 1.7038247 | 0.000937 | 0.868056 | 1.243265 | up |
| Com_1022_neg | THC | C21 H30 O2 | 2.962594781 | 1.5668613 | 0.000186 | 0.9375 | 1.300258 | up |
| Com_1046_neg | CP 47,497-C8-Homolog C-8-hydroxy metabolite | C22 H36 O3 | 3.55295292 | 1.8290186 | 7.37E-05 | 0.923611 | 1.269008 | up |
| Com_1110_neg | Ethyl Linoleate | C20 H36 O2 | 7.685815837 | 2.9421984 | 0.005986 | 0.854167 | 1.191004 | up |
| Com_1213_neg | Elaidolinolenic acid | C18 H30 O2 | 3.185244037 | 1.6714039 | 0.002476 | 0.958333 | 1.48269 | up |
| Com_1283_neg | 3-(acetamidomethylidene)-2-(hydroxymethyl)  succinic acid | C8 H11 N O6 | 7.423098558 | 2.8920215 | 3.41E-06 | 0.965278 | 2.379394 | up |
| Com_1292_neg | 4,5-Dihydroxy-4-(2,3,4-trihydroxytetrahydro  -2-furanyl)-3,4-dihydro-2H-pyrrole-2-  carboxylic acid | C9 H13 N O8 | 9.617671232 | 3.2656876 | 8.09E-07 | 0.972222 | 2.548906 | up |
| Com_1432_neg | Dihydrogeranylgeranyl diphosphate | C20 H38 O7 P2 | 3.348719042 | 1.7436093 | 0.000176 | 0.909722 | 1.373415 | up |
| Com_1468_neg | (2S)-3-{[(2R,3R,4R,5R,6R)-3-Acetamido-  5-hydroxy-6-(hydroxymethyl)-4-{[(2R,3R,  4S,5R,6R)-3,4,5-trihydroxy-6-(hydroxym  ethyl)tetrahydro-2H-pyran-2-yl]oxy}tetrah  ydro-2H-pyran-2-yl]oxy}-2-aminopropanoic  acid | C17 H30 N2 O13 | 5.97949617 | 2.5800239 | 0.008264 | 0.763889 | 1.176761 | up |
| Com_1563_neg | soyasapogenol B 3-O-beta-glucuronide | C36 H58 O9 | 4.64583496 | 2.2159379 | 3.22E-07 | 1 | 1.945554 | up |
| Com_156_neg | 4-Phenolsulfonic acid | C6 H6 O4 S | 12.43657015 | 3.6365168 | 0.00045 | 0.875 | 2.539339 | up |
| Com_164_neg | 4-O-(beta-L-Araf)-cis-L-Hyp | C10 H17 N O7 | 7.026749873 | 2.8128575 | 0.00307 | 0.805556 | 1.311235 | up |
| Com_1700_neg | 2-[(2E,6E,10Z)-14,15-Dihydroxy-11-  (hydroxymethyl)-3,7,15-trimethyl-2,6,  10-hexadecatrien-1-yl]-2,4,6,9-tetrahy  droxy-5,7-dimethyl-1H-phenalene-1,3  (2H)-dione | C35 H46 O9 | 20.7211544 | 4.3730325 | 1.22E-09 | 1 | 3.702495 | up |
| Com_1800_neg | FMNH2 | C17 H23 N4 O9 P | 14.31066566 | 3.8390189 | 0.000702 | 0.881944 | 3.073586 | up |
| Com_209_neg | THREONIC ACID, L- | C4 H8 O5 | 3.574225396 | 1.8376306 | 3.45E-05 | 0.923611 | 1.726139 | up |
| Com_218_neg | alpha-Ketoglutaric acid | C5 H6 O5 | 2.721028132 | 1.4441519 | 0.00183 | 0.861111 | 1.180753 | up |
| Com_219_neg | Gluconic acid | C6 H12 O7 | 5.795326624 | 2.53489 | 9.88E-07 | 0.9375 | 2.371642 | up |
| Com_2215_neg | Malonic acid | C3 H4 O4 | 3.712668503 | 1.8924565 | 0.000189 | 0.902778 | 1.521856 | up |
| Com_2543_neg | Alanyl-N-(6-amino-2-pyridinyl)-alpha-  glutamine | C13 H19 N5 O4 | 2.874325043 | 1.5232232 | 0.001565 | 0.902778 | 1.042738 | up |
| Com_2613_neg | Artemotil | C17 H28 O5 | 3.966608894 | 1.9879062 | 0.001453 | 0.854167 | 1.098157 | up |
| Com_270_neg | 2-Acetamido-2-deoxyglucose | C8 H15 N O6 | 4.276094922 | 2.0962939 | 8.13E-07 | 0.979167 | 1.366709 | up |
| Com_33_neg | Arachidonic acid | C20 H32 O2 | 2.17202128 | 1.1190382 | 0.017013 | 0.729167 | 1.584941 | up |
| Com_3405_neg | 3,5-Dihydroxy-2-(4-hydroxyphenyl)-4-  oxo-3,4-dihydro-2H-chromen-7-yl  hexopyranoside | C21 H22 O11 | 4.067452011 | 2.0241253 | 0.00874 | 0.784722 | 1.9887 | up |
| Com_3647_neg | (20S)-17,20-Dihydroxypregn-4-en-3-one | C21 H32 O3 | 2.897385819 | 1.5347518 | 3.68E-05 | 0.923611 | 1.00198 | up |
| Com_3692_neg | 2,6-Dideoxy-3-O-methyl-L-arabino-hexo  pyranose | C7 H14 O4 | 2.457903198 | 1.2974281 | 0.031671 | 0.8125 | 1.020543 | up |
| Com_4041_neg | Glycitin | C22 H22 O10 | 8.97217627 | 3.165458 | 8.93E-08 | 1 | 2.231288 | up |
| Com_4056_neg | oxandrolone | C19 H30 O3 | 2.978551028 | 1.5746107 | 1.12E-07 | 0.993056 | 1.006049 | up |
| Com_4130_neg | 3-hexaprenyl-4,5-dihydroxybenzoic acid | C37 H54 O4 | 3.131685265 | 1.6469392 | 0.000474 | 0.875 | 1.048712 | up |
| Com_422_neg | (2S)-3-Hydroxy-2-({[(3S,4S,5R)-2,3,4-  trihydroxy-5-(hydroxymethyl)tetrahydro-  2-furanyl]methyl}amino)butanoic acid  (non-preferred name) | C10 H19 N O8 | 3.957992217 | 1.9847688 | 2.49E-06 | 0.993056 | 1.360762 | up |
| Com_4280_neg | Baicalin | C21 H18 O11 | 8.002142167 | 3.0003863 | 4.00E-07 | 0.986111 | 2.331912 | up |
| Com_4426_neg | Ginsenoside Ro | C48 H76 O19 | 12.65914221 | 3.6621077 | 7.61E-11 | 1 | 2.898654 | up |
| Com_455_neg | methyl alpha-D-mannoside | C7 H14 O6 | 2.49303478 | 1.317903 | 0.000465 | 0.895833 | 1.07085 | up |
| Com_4581_neg | 6-Decylubiquinone | C19 H30 O4 | 3.100321081 | 1.6324176 | 0.000812 | 0.868056 | 1.070742 | up |
| Com_4773_neg | 3-O-(3-Methylbutanoyl)-beta-D-fructo  furanosyl 2-O-acetyl-3-O-(2-methylbut  anoyl)-alpha-D-glucopyranoside | C24 H40 O14 | 2.291588603 | 1.1963481 | 0.002803 | 0.840278 | 1.461188 | up |
| Com_4967_neg | olmelin | C16 H12 O5 | 8.851454639 | 3.1459146 | 7.44E-06 | 0.979167 | 2.460171 | up |
| Com_5393_neg | Quercitrin | C21 H20 O11 | 4.237344728 | 2.0831605 | 0.001913 | 0.861111 | 1.851043 | up |
| Com_53_neg | Soyasaponin I | C48 H78 O18 | 12.81070617 | 3.6792781 | 1.50E-10 | 1 | 2.953122 | up |
| Com_5545_neg | 3-O-[(2S,3R,4R)-3,4-Dihydroxy-4-  (hydroxymethyl)tetrahydro-2-furanyl]  -beta-D-xylopyranosyl-(1->4)-6-deoxy  -alpha-L-mannopyranosyl-(1->2)-1-O-  [(2beta,3beta,16alpha)-3-(beta-D-  glucopyranosyloxy)-2,16,24-trihydroxy  -28-oxoolean-12-en-28-yl]-alpha-L-  arabinopyranose | C57 H92 O27 | 6.072655295 | 2.6023275 | 9.38E-10 | 1 | 2.095597 | up |
| Com_559_neg | Daidzein | C15 H10 O4 | 8.502109303 | 3.0878208 | 1.98E-09 | 1 | 2.469035 | up |
| Com_622_neg | L-Cystine | C6 H12 N2 O4 S2 | 9.05632585 | 3.1789259 | 1.22E-05 | 0.923611 | 2.126581 | up |
| Com_651_neg | Pantothenic acid | C9 H17 N O5 | 2.515334065 | 1.33075 | 0.00373 | 0.909722 | 1.311911 | up |
| Com_6779_neg | 1-O-(Hydroxy{[(6Z,10E,14E)-3,7,11,  15,19-pentamethyl-6,10,14,18-icosatetraen  -1-yl]oxy}phosphoryl)-beta-D-xylopyranose | C30 H53 O8 P | 3.006362416 | 1.5880189 | 6.04E-05 | 0.916667 | 1.382526 | up |
| Com_7079_neg | Rutin | C27 H30 O16 | 5.170331591 | 2.3702568 | 5.48E-05 | 0.923611 | 1.793912 | up |
| Com_7133_neg | (1S,6S)-6-Aminooctahydro-1-indolizinyl  acetate | C10 H18 N2 O2 | 2.931040282 | 1.5514128 | 0.001164 | 0.868056 | 1.206975 | up |
| Com_7328_neg | Araloside A | C47 H74 O18 | 5.224653495 | 2.3853354 | 1.24E-06 | 1 | 1.856721 | up |
| Com_7680_neg | Escin IB | C55 H86 O24 | 5.271123172 | 2.3981104 | 5.32E-08 | 1 | 1.898326 | up |
| Com_796_neg | Genistein | C15 H10 O5 | 10.45851728 | 3.3866064 | 1.41E-07 | 0.986111 | 2.937547 | up |
| Com_8188_neg | N-Phenylacetylglutamic acid | C13 H15 N O5 | 3.497820928 | 1.8064564 | 0.002343 | 0.833333 | 1.638672 | up |
| Com_8213_neg | N-[(2E)-3-(4-Hydroxyphenyl)-2-propenoyl]  tryptophan | C20 H18 N2 O4 | 5.968158009 | 2.5772857 | 2.90E-09 | 1 | 2.05429 | up |
| Com_8226_neg | Z-Arg-Arg-NHMec | C30 H39 N9 O6 | 3.228822275 | 1.691008 | 0.003309 | 0.826389 | 1.220661 | up |
| Com_869_neg | Phenol | C6 H6 O | 6.593731094 | 2.7210951 | 0.006557 | 0.791667 | 1.504179 | up |
| Com_9074_neg | (1S,2R,3S,5S,6S,16E,18E,20R,21S)-11-  Chloro-21-hydroxy-12,20-dimethoxy-2,  5,9,16-tetramethyl-8,23-dioxo-4,24-dioxa  -9,22-diazatetracyclo[19.3.1.1~10,14~.0~  3,5~]hexacosa-10(26),11,13,16,18-pentaen  -6-yl 2-methylpropanoate | C32 H43 Cl N2 O9 | 2.322040102 | 1.2153929 | 0.000853 | 0.868056 | 1.186983 | up |
| Com_9266_neg | (19R,31R,42Z)-19-[(9Z)-9-Hexadecenoy  loxy]-22,25,28-trihydroxy-22,28-dioxido  -16,34-dioxo-17,21,23,27,29,33-hexaoxa-  22lambda~5~,28lambda~5~-diphosphahe  npentacont-42-en-31-yl (9Z)-9-octadecenoate | C77 H144 O17 P2 | 5.599422215 | 2.485278 | 0.002093 | 0.881944 | 1.340854 | up |
| Com_9407_neg | NICTOFLORIN | C27 H30 O15 | 2.421647466 | 1.2759889 | 0.026343 | 0.708333 | 1.004869 | up |
| Com_9679_neg | 6-Deoxy-alpha-L-mannopyranosyl-(1->3)  -beta-D-xylopyranosyl-(1->4)-6-deoxy-  alpha-L-mannopyranosyl-(1->2)-6-deoxy  -1-O-[(2beta,3beta,16alpha,17xi)-3-(beta-  D-glucopyranosyloxy)-2,16,23-trihydroxy-  28-oxoolean-12-en-28-yl]-beta-D-galactop  yranose | C59 H96 O27 | 3.073219157 | 1.6197507 | 1.14E-06 | 0.979167 | 1.3615 | up |
| Com_9823_neg | (3beta,16alpha)-16-hydroxy-30-oxo-13,28-  epoxyoleanan-3-yl beta-D-glucopyranosyl-  (1->2)-[beta-D-glucopyranosyl-(1->3)-[beta  -D-xylopyranosyl-(1->2)]-beta-D-glucopyr  anosyl-(1->4)]-alpha-L-arabinopyranoside | C58 H94 O27 | 4.206855093 | 2.0727421 | 1.92E-09 | 1 | 1.684179 | up |
| Com_9879_neg | LTF4 | C28 H44 N2 O8 S | 5.368538167 | 2.4245293 | 6.58E-11 | 1 | 1.823357 | up |
| Com_9880_neg | haplophytine | C37 H40 N4 O7 | 2.679184127 | 1.4217937 | 1.66E-06 | 0.958333 | 1.103386 | up |
| Com_988_neg | Glycitein | C16 H12 O5 | 11.96391666 | 3.5806179 | 1.88E-08 | 1 | 2.982005 | up |
| Com_998_neg | Carboprost | C21 H36 O5 | 8.088046406 | 3.0157913 | 0.00034 | 0.902778 | 1.51723 | up |
| Com_10017_neg | (4R,5S,6S,7R,9R,10R,11E,13E,16R)-6-{[(2S  ,3R,4R,5S,6R)-5-{[(2S,4R,5S,6S)-5-Acetoxy  -4-hydroxy-4,6-dimethyltetrahydro-2H-pyran-2  -yl]oxy}-4-(dimethylamino)-3-hydroxy-6-  methyltetrahydro-2H-pyran-2-yl]oxy}-10-hydr  oxy-5-methoxy-9,16-dimethyl-2-oxo-7-(2-oxo  ethyl)oxacyclohexadeca-11,13-dien-4-yl acetate | C39 H63 N O15 | 0.35787733 | -1.482463 | 0.001484 | 0.868056 | 1.123516 | down |
| Com_10049_neg | 2-Amino-3-hydroxy-N-[(6S,9R,10S,11Z,13R,  18aS)-11-hydroxy-6,13-diisopropyl-2,5,9-trime  thyl-1,4,7,14-tetraoxo-2,3,4,5,6,7,9,10,13,14,16,  17,18,18a-tetradecahydro-1H-pyrrolo[2,1-i][1,4  ,7,10,13]oxatetraazacyclohexadecin-10-yl]-4-  methylbenzenecarboximidic acid | C31 H46 N6 O8 | 0.355823022 | -1.490768 | 0.000181 | 0.909722 | 1.143576 | down |
| Com_10061_neg | Roflumilast | C17 H14 Cl2 F2 N2 O3 | 0.142678696 | -2.809158 | 1.58E-05 | 0.930556 | 1.697215 | down |
| Com_10077_neg | (14R)-3beta,5,6beta,10,16-pentahydroxy  grayanotoxan-14-yl acetate | C22 H36 O7 | 0.277471202 | -1.84959 | 6.76E-06 | 0.951389 | 1.307695 | down |
| Com_1007_neg | Methyl N-[5-(4-oxo-3,4-dihydrophthalazin  -1-yl)-1H-benzo[d]imidazol-2-yl]carbamate | C17 H13 N5 O3 | 0.17577458 | -2.508202 | 0.000166 | 0.902778 | 1.825792 | down |
| Com_10095_neg | 7-Sulfocholic acid | C24 H40 O8 S | 0.249253318 | -2.004315 | 0.000397 | 0.9375 | 1.064436 | down |
| Com_10100_neg | 2-{[(3alpha,5beta,7alpha,8xi,12alpha)-3,  7,12-Trihydroxy-24-oxocholan-24-yl]amino}  ethyl hydrogen sulfate | C26 H45 N O8 S | 0.187302831 | -2.416555 | 2.62E-09 | 1 | 1.579741 | down |
| Com_10173_neg | 1-Octen-3-yl 6-O-beta-D-xylopyranosyl-beta  -D-glucopyranoside | C19 H34 O10 | 0.289091573 | -1.790402 | 3.55E-05 | 0.951389 | 1.096931 | down |
| Com_10193_neg | jamaicamide C | C27 H39 Cl N2 O4 | 0.170857032 | -2.549138 | 5.55E-07 | 0.958333 | 1.617586 | down |
| Com_10206_neg | 4-HYDROXYCARVEDILOL | C24 H26 N2 O5 | 0.207605464 | -2.268084 | 1.55E-06 | 0.986111 | 1.371013 | down |
| Com_1021_neg | 2-Acetamido-2,6-dideoxy-alpha-D-galactopy  ranose | C8 H15 N O5 | 0.368469279 | -1.440384 | 0.004796 | 0.826389 | 1.164253 | down |
| Com_10257_neg | Retinyl beta-glucuronide | C26 H38 O7 | 0.241706695 | -2.048671 | 1.80E-06 | 0.958333 | 1.335106 | down |
| Com_10315_neg | (2R)-2-Hydroxy-3-(phosphonooxy)propyl  (13Z,16Z)-13,16-docosadienoate | C25 H47 O7 P | 0.217581123 | -2.200375 | 0.00632 | 0.875 | 1.102884 | down |
| Com_10366_neg | pretazettine | C18 H21 N O5 | 0.299450437 | -1.739611 | 0.000104 | 0.9375 | 1.034514 | down |
| Com_10392_neg | Leukotriene E3 | C23 H39 N O5 S | 0.226146055 | -2.144673 | 1.71E-06 | 0.986111 | 1.315932 | down |
| Com_1057_neg | persin | C23 H40 O4 | 0.213138493 | -2.230137 | 4.69E-05 | 0.9375 | 1.317457 | down |
| Com_1156_neg | Adenosine | C10 H13 N5 O4 | 0.358992079 | -1.477976 | 5.46E-05 | 0.923611 | 1.025614 | down |
| Com_11_neg | Taurochenodeoxycholic acid | C26 H45 N O6 S | 0.308528506 | -1.696524 | 0.000202 | 0.916667 | 1.666266 | down |
| Com_1226_neg | Tauropine | C5 H11 N O5 S | 0.175486913 | -2.510565 | 1.84E-07 | 1 | 1.538226 | down |
| Com_1254_neg | Tetradecanedioic acid | C14 H26 O4 | 0.391089572 | -1.354429 | 0.002856 | 0.819444 | 1.065548 | down |
| Com_1284_neg | Lithocholic acid taurine conjugate | C26 H45 N O5 S | 0.152467766 | -2.713424 | 4.02E-06 | 0.972222 | 1.772547 | down |
| Com_1303_neg | (5-Benzyl-3,6-dioxo-2-piperazinyl)acetic acid | C13 H14 N2 O4 | 0.196959688 | -2.344028 | 5.38E-05 | 0.9375 | 1.588875 | down |
| Com_1311_neg | UDP-N-acetylglucosamine | C17 H27 N3 O17 P2 | 0.154012716 | -2.698879 | 1.54E-05 | 0.951389 | 2.190742 | down |
| Com_1312_neg | Cyproconazole | C15 H18 Cl N3 O | 0.372061332 | -1.426388 | 0.000141 | 0.916667 | 1.024515 | down |
| Com_1339_neg | salnacedin | C12 H13 N O5 S | 0.091231828 | -3.454319 | 1.51E-06 | 0.972222 | 2.397629 | down |
| Com_136_neg | DL-Lactic Acid | C3 H6 O3 | 0.134737531 | -2.891776 | 1.64E-06 | 0.986111 | 1.741 | down |
| Com_1409_neg | (2E)-3-(Acetoxymethyl)-5-[(1S,4aR,8aR)-2-  (hydroxymethyl)-5,5,8a-trimethyl-1,4,4a,5,  6,7,8,8a-octahydro-1-naphthalenyl]-2-pente  noic acid | C22 H34 O5 | 0.178607304 | -2.485137 | 5.73E-08 | 1 | 1.564902 | down |
| Com_1416_neg | spinacine | C7 H9 N3 O2 | 0.160552677 | -2.638881 | 1.62E-05 | 0.944444 | 1.603322 | down |
| Com_1447_neg | Nicotinuric acid | C8 H8 N2 O3 | 0.227217566 | -2.137854 | 4.57E-08 | 1 | 1.426653 | down |
| Com_1460_neg | PIVOPRIL | C16 H27 N O4 S | 0.242668775 | -2.04294 | 8.09E-05 | 0.909722 | 1.474411 | down |
| Com_1474_neg | 8-hydroxy-7-methylguanine | C6 H7 N5 O2 | 0.232730845 | -2.103266 | 8.43E-07 | 0.965278 | 1.33212 | down |
| Com_1486_neg | Linalyl isovalerate | C15 H26 O2 | 0.409813043 | -1.286962 | 0.000582 | 0.875 | 1.128766 | down |
| Com_1534_neg | 5889 | C23 H38 O6 | 0.259102881 | -1.948403 | 0.000321 | 0.854167 | 1.23732 | down |
| Com_1572_neg | Omeprazole | C17 H19 N3 O3 S | 0.059582692 | -4.068963 | 6.92E-11 | 1 | 2.624293 | down |
| Com_1636_neg | taurolithocholic acid sulfate | C26 H45 N O8 S2 | 0.146758343 | -2.768486 | 1.76E-06 | 0.986111 | 1.687377 | down |
| Com_1650_neg | Cascarillin | C22 H32 O7 | 0.3040941 | -1.71741 | 1.20E-05 | 0.944444 | 1.102243 | down |
| Com_1701_neg | 13-Hydroxykaur-16-en-18-oic acid | C20 H30 O3 | 0.064228584 | -3.960641 | 0.001596 | 0.875 | 1.814889 | down |
| Com_1768_neg | Indole-3-pyruvic acid | C11 H9 N O3 | 0.152877459 | -2.709552 | 2.21E-05 | 0.986111 | 2.154915 | down |
| Com_1814_neg | 7086 | C12 H15 N O5 S | 0.009538242 | -6.712061 | 5.29E-06 | 0.986111 | 3.718517 | down |
| Com_1830_neg | N-Acetyl-L-phenylalanine | C11 H13 N O3 | 0.342365596 | -1.54639 | 9.29E-05 | 0.9375 | 1.030556 | down |
| Com_1831_neg | Aganepag isopropyl | C27 H37 N O4 S | 0.142101546 | -2.815006 | 3.06E-09 | 1 | 1.758114 | down |
| Com_1889_neg | N-Acetyl-L-leucine | C8 H15 N O3 | 0.327830526 | -1.608978 | 3.18E-06 | 0.993056 | 1.071952 | down |
| Com_1892_neg | 5-Methylcytosine | C5 H7 N3 O | 0.131369226 | -2.928301 | 1.08E-06 | 0.979167 | 1.88311 | down |
| Com_1899_neg | Oxycarboxin | C12 H13 N O4 S | 0.156529126 | -2.675497 | 4.97E-07 | 0.972222 | 1.720473 | down |
| Com_1946_neg | N-(2-Cyanoethyl)-L-glutamine | C8 H13 N3 O3 | 0.243222953 | -2.039649 | 3.95E-06 | 0.979167 | 1.272129 | down |
| Com_1965_neg | N-(1-Benzylpiperidin-4-yl)-6-phenylth  ieno[3,2-d]pyrimidin-4-amine | C24 H24 N4 S | 0.136710979 | -2.870799 | 5.76E-06 | 0.958333 | 1.911399 | down |
| Com_1990_neg | (Z)-N-[(2S)-2-{(Z)-[(2S,3S,6R,7R)-2,3  -Diamino-1,6,7,8-tetrahydroxy-8-{1-[hydro  xy(imino)methyl]-2-imino-4-imidazolidinyl}  octylidene]amino}-1-hydroxypropylidene]-  L-valine | C20 H38 N8 O8 | 0.269391228 | -1.892225 | 4.87E-07 | 1 | 1.337821 | down |
| Com_2005_neg | disobutamide | C23 H38 Cl N3 O | 0.232987344 | -2.101677 | 5.86E-08 | 0.993056 | 1.400971 | down |
| Com_2070_neg | Cholic acid | C24 H40 O5 | 0.232861557 | -2.102456 | 0.000667 | 0.895833 | 1.590445 | down |
| Com_2091_neg | Homocarnosine | C10 H16 N4 O3 | 0.258472937 | -1.951915 | 5.26E-05 | 0.958333 | 1.145231 | down |
| Com_2178_neg | phenylamine | C6 H7 N | 0.231146235 | -2.113122 | 1.22E-09 | 1 | 1.396439 | down |
| Com_2227_neg | 4-Nitroaniline | C6 H6 N2 O2 | 0.209247429 | -2.256718 | 0.000348 | 0.916667 | 1.221949 | down |
| Com_2243_neg | 1,9b-Dihydroxy-6,6,9a-trimethyl-5,  5a,6,7,8,9,9a,9b-octahydronaphtho  [1,2-c]furan-3(1H)-one | C15 H22 O4 | 0.332635617 | -1.587985 | 5.92E-05 | 0.930556 | 1.092704 | down |
| Com_2260_neg | taurohyocholic acid | C26 H45 N O7 S | 0.393569943 | -1.345308 | 0.002051 | 0.840278 | 1.43159 | down |
| Com_22_neg | Deoxycholic acid | C24 H40 O4 | 0.154041214 | -2.698612 | 2.34E-06 | 0.972222 | 1.775057 | down |
| Com_232_neg | Bis(2-ethylhexyl) phthalate | C24 H38 O4 | 0.072337381 | -3.789115 | 5.34E-06 | 0.972222 | 3.247469 | down |
| Com_2336_neg | Acetochlor ESA | C14 H21 N O5 S | 0.157885405 | -2.66305 | 5.84E-08 | 1 | 1.821401 | down |
| Com_2366_neg | (9alpha,11alpha,13E,15S)-5,6,9,11,15  -Pentahydroxyprost-13-en-1-oic acid | C20 H36 O7 | 0.224585911 | -2.154661 | 1.24E-07 | 0.979167 | 1.375952 | down |
| Com_2414_neg | 2-Amino-3-(1H-imidazol-4-yl)propan  -1-ol | C6 H11 N3 O | 0.135992199 | -2.878404 | 1.45E-06 | 0.972222 | 1.814754 | down |
| Com_2486_neg | delta-guanidinovaleric acid | C6 H13 N3 O2 | 0.140211313 | -2.834325 | 9.14E-07 | 0.979167 | 1.822708 | down |
| Com_2500_neg | N-Acetyl-DL-tryptophan | C13 H14 N2 O3 | 0.218541222 | -2.194023 | 2.55E-07 | 0.993056 | 1.444878 | down |
| Com_2513_neg | Alvocidib | C21 H20 Cl N O5 | 0.161270741 | -2.632443 | 0.000287 | 0.930556 | 1.871499 | down |
| Com_2520_neg | phenyl urea | C7 H8 N2 O | 0.2304476 | -2.117489 | 4.15E-08 | 1 | 1.411269 | down |
| Com_2586_neg | 5-Fluoro MN-18 | C23 H22 F N3 O | 0.355677871 | -1.491357 | 0.008741 | 0.8125 | 1.202293 | down |
| Com_2662_neg | Acetanilide | C8 H9 N O | 0.239496924 | -2.061921 | 2.13E-09 | 1 | 1.360787 | down |
| Com_2690_neg | Undecanoic acid | C11 H22 O2 | 0.387248795 | -1.368667 | 0.002504 | 0.833333 | 1.156665 | down |
| Com_2705_neg | PIPEROPHOS | C14 H28 N O3 P S2 | 0.171987936 | -2.539621 | 1.22E-07 | 0.986111 | 1.659224 | down |
| Com_2740_neg | Desoxymycin | C21 H41 N7 O11 | 0.229316811 | -2.124586 | 2.02E-06 | 0.972222 | 1.583465 | down |
| Com_2759_neg | N-Sulfanilyl-3,4-xylamide | C15 H16 N2 O3 S | 0.120041028 | -3.058401 | 1.59E-07 | 0.993056 | 1.974576 | down |
| Com_2772_neg | S-(5-deoxy-beta-D-ribos-5-yl)-L-  homocysteine | C9 H17 N O6 S | 0.298824691 | -1.742629 | 0.001534 | 0.861111 | 1.263974 | down |
| Com_2773_neg | Dihydrostreptomycin | C21 H41 N7 O12 | 0.377044242 | -1.407194 | 0.001403 | 0.861111 | 1.401611 | down |
| Com_2777_neg | Tomelukast | C16 H22 N4 O3 | 0.126661128 | -2.980954 | 6.82E-07 | 0.972222 | 2.024555 | down |
| Com_2782_neg | 2-(Hydroxymethyl)-1-methyl-3,4,5-  piperidinetriol | C7 H15 N O4 | 0.299298341 | -1.740344 | 3.97E-06 | 0.965278 | 1.077281 | down |
| Com_2827_neg | Aspirin | C9 H8 O4 | 0.188788769 | -2.405155 | 2.13E-05 | 0.951389 | 1.436741 | down |
| Com_2833_neg | Enterostatin | C21 H36 N8 O6 | 0.065397972 | -3.93461 | 3.07E-11 | 1 | 2.604816 | down |
| Com_2878_neg | bentazepam | C17 H16 N2 O S | 0.389842515 | -1.359037 | 0.000856 | 0.868056 | 1.194424 | down |
| Com_2896_neg | (2beta,3beta,5beta,20xi,22R)-2,3,  14,20,22,25-Hexahydroxy-6-oxo  cholest-7-en-26-al | C27 H42 O8 | 0.112442907 | -3.152735 | 1.67E-09 | 0.993056 | 2.028033 | down |
| Com_2898_neg | Brexpiprazole | C25 H27 N3 O2 S | 0.268156736 | -1.898852 | 0.005707 | 0.826389 | 1.030202 | down |
| Com_2900_neg | Asparenomycin A | C14 H16 N2 O6 S | 0.187959258 | -2.411508 | 3.61E-06 | 0.958333 | 1.544506 | down |
| Com_2904_neg | Ascorbyl palmitate | C22 H38 O7 | 0.182781592 | -2.451807 | 4.72E-09 | 1 | 1.63359 | down |
| Com_2915_neg | Salicin 6-phosphate | C13 H19 O10 P | 0.226874911 | -2.140031 | 2.97E-05 | 0.930556 | 1.518277 | down |
| Com_2928_neg | asn-ser | C7 H13 N3 O5 | 0.298429996 | -1.744536 | 1.90E-06 | 0.986111 | 1.060092 | down |
| Com_297_neg | Podecdysone B | C27 H42 O6 | 0.242135947 | -2.046111 | 4.12E-05 | 0.916667 | 1.484857 | down |
| Com_2993_neg | 2444643WXK | C13 H18 O4 | 0.345863947 | -1.531723 | 0.000423 | 0.881944 | 1.083622 | down |
| Com_3004_neg | 4-(5,6-Dihydroxy-6-methyl-1-hepten  -2-yl)-1-methyl-1,2-cyclohexanediol | C15 H28 O4 | 0.159846132 | -2.645244 | 1.57E-08 | 1 | 1.691752 | down |
| Com_3073_neg | Glycerol 3-phosphate | C3 H9 O6 P | 0.276371717 | -1.855318 | 2.37E-06 | 0.972222 | 1.159534 | down |
| Com_3080_neg | p-Cresylsulfate | C7 H8 O4 S | 0.290532972 | -1.783226 | 5.97E-05 | 0.930556 | 1.249697 | down |
| Com_310_neg | N-Arachidonoyl taurine | C22 H37 N O4 S | 0.306754363 | -1.704844 | 1.16E-06 | 0.972222 | 1.118509 | down |
| Com_3114_neg | 7369 | C18 H27 N O2 S | 0.27528656 | -1.860994 | 1.30E-06 | 0.986111 | 1.124297 | down |
| Com_3126_neg | 3-(indol-3-yl)-2-oxobutyric acid | C12 H11 N O3 | 0.178577684 | -2.485376 | 5.61E-07 | 1 | 1.75232 | down |
| Com_3203_neg | 6,7-Dihydropteridine | C6 H6 N4 | 0.170062854 | -2.55586 | 0.000196 | 0.909722 | 1.465285 | down |
| Com_3207_neg | Erisimin | C29 H42 O9 | 0.351744372 | -1.507401 | 0.000401 | 0.895833 | 1.281523 | down |
| Com_3365_neg | Zolpidem | C19 H21 N3 O | 0.204894701 | -2.287045 | 1.79E-09 | 0.993056 | 1.490662 | down |
| Com_3480_neg | (3alpha,5beta,7alpha,8xi,9xi,12alpha  ,14xi,25R)-7-(beta-D-Galactopyranos  yloxy)-3,12-dihydroxycholestan-27-yl  acetate | C35 H60 O10 | 0.242622576 | -2.043214 | 0.000107 | 0.951389 | 1.141974 | down |
| Com_3499_neg | 4-Hydroxyphenylpyruvic acid | C9 H8 O4 | 0.167089085 | -2.581311 | 6.18E-06 | 0.958333 | 1.629705 | down |
| Com_3596_neg | 6-Methoxy-3-(1,3-thiazol-2-yl)-1H-indole | C12 H10 N2 O S | 0.130300265 | -2.940088 | 1.33E-07 | 0.986111 | 1.87144 | down |
| Com_3632_neg | paracetamol sulfate | C8 H9 N O5 S | 0.171821305 | -2.541019 | 7.73E-09 | 1 | 1.629233 | down |
| Com_3699_neg | 4-[(2-Hydroxy-4-methylpentanoyl)amino]  -7-isobutyl-3-isopropyl-5,8-dioxo-2-oxa-  6,9-diazabicyclo[10.2.2]hexadeca-1(14),  12,15-triene-10-carboxylic acid | C27 H41 N3 O7 | 0.311809828 | -1.681262 | 0.000196 | 0.888889 | 1.355586 | down |
| Com_3711_neg | 1,2-Diamino-4-nitrobenzene | C6 H7 N3 O2 | 0.280148125 | -1.835738 | 0.00019 | 0.923611 | 1.07471 | down |
| Com_3765_neg | 12-O-Tetradecanoylphorbol-13-acetate | C36 H56 O8 | 0.209987454 | -2.251625 | 7.92E-07 | 0.972222 | 1.553988 | down |
| Com_3786_neg | 3,3-Dimethyl-5-oxo-5-[4-(2-pyrimidinyl)  piperazino]pentanoic acid | C15 H22 N4 O3 | 0.315700721 | -1.663371 | 0.000109 | 0.930556 | 1.130412 | down |
| Com_380_neg | 2-Aminoethyl (2R)-3-[(1Z)-1-hexadecen-  1-yloxy]-2-hydroxypropyl hydrogen phosphate | C21 H44 N O6 P | 0.375321763 | -1.4138 | 0.000739 | 0.854167 | 1.583437 | down |
| Com_3810_neg | N-Acetyl-L-cysteine | C5 H9 N O3 S | 0.209305445 | -2.256318 | 0.00065 | 0.895833 | 1.43728 | down |
| Com_3814_neg | N-Acetylneuraminic acid | C11 H19 N O9 | 0.121204979 | -3.044479 | 1.12E-09 | 1 | 1.934161 | down |
| Com_3819_neg | Indole-3-butyric acid | C12 H13 N O2 | 0.104156009 | -3.263182 | 2.79E-06 | 0.965278 | 2.187356 | down |
| Com_3823_neg | Manoalide | C25 H36 O5 | 0.335122975 | -1.577237 | 0.000778 | 0.888889 | 1.075986 | down |
| Com_383_neg | Levulinic acid | C5 H8 O3 | 0.303548396 | -1.720002 | 9.20E-05 | 0.916667 | 1.408499 | down |
| Com_4030_neg | 3, 5-Tetradecadiencarnitine | C21 H37 N O4 | 0.351285239 | -1.509285 | 0.000115 | 0.909722 | 1.034001 | down |
| Com_4071_neg | Indole-3-carboxilic acid-O-sulphate | C9 H7 N O5 S | 0.286377614 | -1.804009 | 0.00209 | 0.868056 | 1.339725 | down |
| Com_41_neg | Succinic acid | C4 H6 O4 | 0.256363985 | -1.963734 | 0.000226 | 0.923611 | 1.631585 | down |
| Com_4215_neg | Ethyl 5-hydroxy-6-nitro-4-oxo-8-propyl-  4H-chromene-2-carboxylate | C15 H15 N O7 | 0.078795612 | -3.665741 | 3.44E-07 | 0.979167 | 2.453051 | down |
| Com_4217_neg | 2CX9CN85QM | C13 H21 N O6 S | 0.222457898 | -2.168396 | 5.52E-05 | 0.909722 | 1.435378 | down |
| Com_421_neg | Pyruvic acid | C3 H4 O3 | 0.295679706 | -1.757893 | 9.09E-05 | 0.923611 | 1.348363 | down |
| Com_4286_neg | Rabeprazole | C18 H21 N3 O3 S | 0.164402557 | -2.604695 | 9.23E-08 | 1 | 1.638841 | down |
| Com_4296_neg | Azocyclotin | C20 H35 N3 Sn | 0.320360052 | -1.642234 | 4.89E-05 | 0.951389 | 1.273458 | down |
| Com_4316_neg | 1-(2-Deoxy-alpha-D-erythro-pentofuranosyl)  -2,4-dioxo-1,2,3,4-tetrahydro-5-pyrimidinecar  boxylic acid | C10 H12 N2 O7 | 0.303285199 | -1.721253 | 0.002967 | 0.854167 | 1.019904 | down |
| Com_4415_neg | Methyl Jasmonate | C13 H20 O3 | 0.318351351 | -1.651308 | 0.000227 | 0.881944 | 1.141437 | down |
| Com_4449_neg | DIETHAMINE | C11 H13 F3 N4 O4 | 0.243688462 | -2.03689 | 0.000702 | 0.888889 | 1.311259 | down |
| Com_4450_neg | (1E,4S,5E,9E,11aS,14S,14aR,15S,15aR,16aS,  16bR)-12-Hydroxy-14-(1H-indol-3-ylmethyl)-  4,6,15,15a-tetramethyl-3,4,7,8,14,14a,15,15a,  16a,16b-decahydro-11H-cyclotrideca[d]oxireno  [f]isoindol-11-one | C32 H38 N2 O3 | 0.30999106 | -1.689701 | 5.03E-06 | 1 | 1.004205 | down |
| Com_4451_neg | DL-Histidine | C6 H9 N3 O2 | 0.274571437 | -1.864747 | 4.99E-06 | 0.986111 | 1.14476 | down |
| Com_448_neg | N-Acetylanthranilic acid | C9 H9 N O3 | 0.243039774 | -2.040736 | 1.34E-09 | 1 | 1.360497 | down |
| Com_4522_neg | 3-HYDROXY-MYRISTIC ACID | C14 H28 O3 | 0.370652559 | -1.431861 | 0.000102 | 0.895833 | 1.035334 | down |
| Com_4629_neg | Caproylglycine | C12 H23 N O3 | 0.238353543 | -2.068825 | 2.53E-06 | 1 | 1.267639 | down |
| Com_4635_neg | Rilpivirine | C22 H18 N6 | 0.050766259 | -4.299986 | 5.76E-10 | 1 | 2.907979 | down |
| Com_471_neg | D-(+)-Maltose | C12 H22 O11 | 0.30687941 | -1.704256 | 9.68E-05 | 0.895833 | 1.115643 | down |
| Com_4751_neg | Finasteride | C23 H36 N2 O2 | 0.285086538 | -1.810528 | 2.99E-08 | 1 | 1.259669 | down |
| Com_4789_neg | 10-(4-Sulfophenyl) decanoic acid | C16 H24 O5 S | 0.34081654 | -1.552933 | 1.07E-06 | 0.993056 | 1.022221 | down |
| Com_4845_neg | Teneligliptin | C22 H30 N6 O S | 0.23055748 | -2.116802 | 2.12E-08 | 1 | 1.322357 | down |
| Com_4872_neg | S-{[(1aR,7aS,10aS,10bR)-1a,5-Dimethyl-9-  oxo-1a,2,3,6,7,7a,8,9,10a,10b-decahydroox  ireno[9,10]cyclodeca[1,2-b]furan-8-yl]meth  yl}-L-cysteine | C18 H27 N O5 S | 0.324397759 | -1.624164 | 1.81E-06 | 0.958333 | 1.076161 | down |
| Com_4910_neg | Pivagabine | C9 H17 N O3 | 0.248185008 | -2.010512 | 6.66E-07 | 0.986111 | 1.257434 | down |
| Com_4919_neg | trehalose 6-phosphate | C12 H23 O14 P | 0.345665695 | -1.532551 | 2.17E-06 | 0.965278 | 1.025967 | down |
| Com_4932_neg | 2-(3-Hydroxy-3,4,5,6-tetrahydro-1H-cyclop  enta[c]furan-4-yl)-3-methoxy-3-oxopropanoic acid | C11 H14 O6 | 0.266871803 | -1.905781 | 1.58E-05 | 0.986111 | 1.094492 | down |
| Com_4960_neg | 5-Hydroxy-2,2-dimethyl-7,10-bis(3-methyl-  2-buten-1-yl)-8-(2,4,5-trihydroxyphenyl)-2H,  6H-pyrano[3,2-g]chromen-6-one | C30 H32 O7 | 0.141050382 | -2.825718 | 2.52E-07 | 1 | 1.722957 | down |
| Com_4965_neg | (1Z)-N-[(3R,4R,5R,6R)-6-{2-[(2R,3S,4R,5R)-  3,4-Dihydroxy-5-(4-hydroxy-2-oxo-1(2H)-  pyrimidinyl)tetrahydro-2-furanyl]-2-hydroxy  ethyl}-2,4,5-trihydroxytetrahydro-2H-pyran-3  -yl]ethanimidic acid (non-preferred name) | C17 H25 N3 O11 | 0.219913139 | -2.184994 | 1.11E-05 | 0.958333 | 1.765511 | down |
| Com_4981_neg | (1S,4R,5R,6R,6aS,9S,9aE,10aR)-1,5-Dihydroxy  -9-(hydroxymethyl)-3-isopropyl-6,10a-dimethyl-  1,2,4,5,6,6a,7,8,9,10a-decahydrodicyclopenta[a,d]  [8]annulen-4-yl alpha-D-glucopyranoside | C26 H42 O9 | 0.147638373 | -2.75986 | 2.60E-08 | 0.993056 | 1.807092 | down |
| Com_4991_neg | (3S,6S)-3-(4-Hydroxybenzyl)-6-(hydroxymethyl)-  2,5-piperazinedione | C12 H14 N2 O4 | 0.215201823 | -2.216238 | 1.58E-07 | 0.979167 | 1.42318 | down |
| Com_5014_neg | Pevonedistat | C21 H25 N5 O4 S | 0.42190476 | -1.245011 | 0.000865 | 0.847222 | 1.021312 | down |
| Com_5017_neg | N-acetyl-L-2-aminoadipic acid | C8 H13 N O5 | 0.317430991 | -1.655485 | 4.41E-06 | 0.965278 | 1.020226 | down |
| Com_5040_neg | 3b-Hydroxy-5-cholenoic acid | C24 H38 O3 | 0.285295663 | -1.80947 | 0.000341 | 0.881944 | 1.298418 | down |
| Com_5134_neg | N-(tert-Butoxycarbonyl)-L-leucine | C11 H21 N O4 | 0.389848652 | -1.359014 | 0.011178 | 0.847222 | 1.182768 | down |
| Com_5159_neg | 2-Fucosyllactose | C18 H32 O15 | 0.33029258 | -1.598184 | 0.001676 | 0.861111 | 1.160973 | down |
| Com_516_neg | Xylitol | C5 H12 O5 | 0.292876061 | -1.771638 | 8.32E-05 | 0.944444 | 1.356678 | down |
| Com_5185_neg | MRS1191 | C31 H27 N O4 | 0.123147139 | -3.021545 | 1.08E-07 | 1 | 1.956793 | down |
| Com_5214_neg | Linsitinib | C26 H23 N5 O | 0.201948316 | -2.307942 | 2.22E-07 | 0.986111 | 1.489247 | down |
| Com_5241_neg | [(2R,3S,4R,5R)-5-(6-Amino-9H-purin-9-yl)-3,4  -dihydroxytetrahydro-2-furanyl]methyl [(2R,3S,  4R,5R)-3,4,5-trihydroxytetrahydro-2-furanyl]  methyl dihydrogen diphosphate | C15 H23 N5 O14 P2 | 0.140853599 | -2.827732 | 1.96E-08 | 1 | 1.743171 | down |
| Com_5287_neg | mangostenone B | C28 H30 O6 | 0.238822411 | -2.06599 | 0.00014 | 0.902778 | 1.290821 | down |
| Com_5300_neg | Ethyl Butylacetylaminopropionate | C11 H21 N O3 | 0.153650963 | -2.702271 | 1.12E-05 | 1 | 1.604129 | down |
| Com_5353_neg | PGF2a ethanolamide | C22 H39 N O5 | 0.145846315 | -2.777479 | 5.81E-08 | 0.993056 | 1.759206 | down |
| Com_5366_neg | Undecylic acid | C11 H22 O2 | 0.239833004 | -2.059898 | 4.06E-10 | 1 | 1.333681 | down |
| Com_5367_neg | (-)-Epothilone A | C26 H39 N O6 S | 0.143517606 | -2.8007 | 3.74E-06 | 0.958333 | 1.868323 | down |
| Com_5397_neg | bidisomide | C22 H34 Cl N3 O2 | 0.364105045 | -1.457573 | 0.000606 | 0.902778 | 1.14677 | down |
| Com_5560_neg | Mupirocin | C26 H44 O9 | 0.124120919 | -3.010182 | 0.00588 | 0.944444 | 1.021796 | down |
| Com_5575_neg | 7alpha-Hydroxy-3-oxochol-4-en-24-oic acid | C24 H36 O4 | 0.139994729 | -2.836556 | 1.47E-05 | 0.944444 | 2.029326 | down |
| Com_5576_neg | Loganin | C17 H26 O10 | 0.253625891 | -1.979226 | 7.87E-08 | 1 | 1.262944 | down |
| Com_5580_neg | carpipramine | C28 H38 N4 O | 0.211383206 | -2.242067 | 1.82E-07 | 0.986111 | 1.400257 | down |
| Com_5609_neg | apronalide | C9 H16 N2 O2 | 0.193678924 | -2.368261 | 2.73E-05 | 0.944444 | 1.535713 | down |
| Com_5611_neg | HC BLUE NO. 2 | C12 H19 N3 O5 | 0.162600925 | -2.620593 | 9.43E-05 | 0.909722 | 1.652657 | down |
| Com_5640_neg | (-)-reserpine | C33 H40 N2 O9 | 0.34829235 | -1.521629 | 0.000179 | 0.902778 | 1.059672 | down |
| Com_5676_neg | Limaprost | C22 H36 O5 | 0.219712271 | -2.186313 | 7.12E-08 | 0.993056 | 1.368273 | down |
| Com_572_neg | 3-O-beta-D-Galactopyranosyl-D-arabinose | C11 H20 O10 | 0.395276273 | -1.339067 | 0.011015 | 0.798611 | 1.132538 | down |
| Com_5773_neg | Cuauhtemone | C15 H24 O3 | 0.179132072 | -2.480904 | 6.23E-07 | 0.965278 | 1.742513 | down |
| Com_5798_neg | 1-Hydroxy-1-[(6R)-4-hydroxy-2-imino-1,2,  5,6,7,8-hexahydro-6-pteridinyl]-2-propanyl  beta-D-glucopyranoside | C15 H25 N5 O8 | 0.279148815 | -1.840894 | 0.00012 | 0.944444 | 1.080832 | down |
| Com_587_neg | Methyl 2-[(methylsulfonyl)amino]benzoate | C9 H11 N O4 S | 0.253988517 | -1.977165 | 1.38E-05 | 0.965278 | 1.408121 | down |
| Com_5911_neg | Primidolol | C17 H23 N3 O4 | 0.259201395 | -1.947855 | 0.000563 | 0.902778 | 1.458382 | down |
| Com_593_neg | (+)-CP 55,940 | C24 H40 O3 | 0.293051021 | -1.770776 | 0.003739 | 0.826389 | 1.293576 | down |
| Com_5999_neg | ceronapril | C21 H33 N2 O6 P | 0.26668916 | -1.906769 | 7.51E-06 | 0.9375 | 1.305759 | down |
| Com_6066_neg | Menadiol | C11 H10 O2 | 0.214722932 | -2.219452 | 7.65E-05 | 0.916667 | 1.632285 | down |
| Com_6090_neg | Hexylresorcinol | C12 H18 O2 | 0.351224485 | -1.509535 | 0.000307 | 0.902778 | 1.046614 | down |
| Com_6093_neg | UDP-N-acetyl-alpha-muramoyl-L-alanyl  -D-glutamic acid | C28 H43 N5 O23 P2 | 0.178817682 | -2.483439 | 0.000548 | 0.902778 | 1.277943 | down |
| Com_6160_neg | (5Z,11beta,13E,15S)-11,15-Dihydroxy-  N-(2-hydroxyethyl)-9-oxoprosta-5,13-dien  -1-amide | C22 H37 N O5 | 0.226207426 | -2.144282 | 1.41E-08 | 1 | 1.359449 | down |
| Com_6252_neg | Eliglustat | C23 H36 N2 O4 | 0.213510706 | -2.22762 | 2.53E-07 | 0.979167 | 1.373971 | down |
| Com_6273_neg | OXOGESTONE PHENPROPIONATE | C29 H38 O3 | 0.180256153 | -2.47188 | 3.82E-06 | 0.972222 | 1.71193 | down |
| Com_6298_neg | Sinapyl alcohol | C11 H14 O4 | 0.267221827 | -1.90389 | 0.000137 | 0.909722 | 1.27275 | down |
| Com_630_neg | Succinic anhydride | C4 H4 O3 | 0.327922705 | -1.608572 | 7.35E-05 | 0.930556 | 1.153175 | down |
| Com_6311_neg | 5-(5-(4-(4,5-dihydro-2-oxazoly)phenol  xy)pentyl)-3-methyl osoxazole | C18 H22 N2 O3 | 0.254428948 | -1.974665 | 1.84E-06 | 0.958333 | 1.319242 | down |
| Com_6368_neg | (-)-trans-Methyl dihydrojasmonate | C13 H22 O3 | 0.200420787 | -2.318896 | 2.44E-05 | 0.944444 | 1.541417 | down |
| Com_6375_neg | amquinate | C18 H24 N2 O3 | 0.146626521 | -2.769782 | 5.57E-08 | 1 | 1.770549 | down |
| Com_6377_neg | primidone | C12 H14 N2 O2 | 0.191666806 | -2.383328 | 2.22E-05 | 0.944444 | 1.564059 | down |
| Com_6392_neg | 2GU13C5298 | C29 H38 O9 | 0.23799449 | -2.071 | 4.64E-05 | 0.923611 | 1.34036 | down |
| Com_640_neg | D-(+)-Arabitol | C5 H12 O5 | 0.161373232 | -2.631527 | 2.05E-10 | 1 | 1.717392 | down |
| Com_6434_neg | PYROQUILON | C11 H11 N O | 0.107457067 | -3.218168 | 7.65E-09 | 1 | 2.014046 | down |
| Com_6454_neg | fominoben | C21 H24 Cl N3 O3 | 0.31378467 | -1.672153 | 0.000164 | 0.909722 | 1.144196 | down |
| Com_645_neg | 4-Indolecarbaldehyde | C9 H7 N O | 0.379689785 | -1.397107 | 0.000172 | 0.9375 | 1.034917 | down |
| Com_6477_neg | Amodiaquine | C20 H22 Cl N3 O | 0.176553151 | -2.501826 | 1.17E-05 | 0.9375 | 1.666694 | down |
| Com_6494_neg | Indole-3-acetic acid | C10 H9 N O2 | 0.128355656 | -2.961781 | 1.55E-07 | 0.993056 | 1.810741 | down |
| Com_6536_neg | 5-(5-Methyl-2-furyl)-2,3-dihydro-  1H-pyrrolizine | C12 H13 N O | 0.174408893 | -2.519454 | 1.41E-06 | 0.972222 | 1.557864 | down |
| Com_6546_neg | 4-Chloroaniline | C6 H6 Cl N | 0.26506445 | -1.915585 | 4.53E-05 | 0.958333 | 1.125074 | down |
| Com_6577_neg | plicamine | C26 H26 N2 O6 | 0.282471822 | -1.823821 | 2.98E-06 | 0.993056 | 1.107194 | down |
| Com_6586_neg | 5722 | C23 H36 O5 | 0.216411273 | -2.208152 | 9.02E-09 | 0.993056 | 1.432566 | down |
| Com_6622_neg | 4-(Hydroxy{7-hydroxy-6-methoxy-  8-[(6-methoxy-2-methyl-1,2,3,4-  tetrahydro-7-isoquinolinyl)oxy]-2-  methyl-1,2,3,4-tetrahydro-1-isoquin  olinyl}methyl)-1,2-benzenediol | C29 H34 N2 O7 | 0.310679206 | -1.686502 | 8.04E-06 | 0.965278 | 1.063564 | down |
| Com_6625_neg | Talmapimod | C27 H30 Cl F N4 O3 | 0.147167135 | -2.764473 | 1.20E-09 | 1 | 1.780705 | down |
| Com_6629_neg | fosravuconazole | C23 H20 F2 N5 O5 P S | 0.06806529 | -3.876937 | 2.19E-05 | 0.965278 | 2.119087 | down |
| Com_6632_neg | NORVERAPAMIL | C26 H36 N2 O4 | 0.344000052 | -1.539519 | 0.000137 | 0.923611 | 1.015554 | down |
| Com_6645_neg | tetrahydrocortisol | C21 H34 O5 | 0.215952193 | -2.211216 | 3.65E-08 | 1 | 1.488521 | down |
| Com_6646_neg | Vilanterol | C24 H33 Cl2 N O5 | 0.340994681 | -1.552179 | 0.000298 | 0.881944 | 1.175099 | down |
| Com_6738_neg | beta-D-Mannopyranosyl-(1->4)-2  -acetamido-2-deoxy-beta-D-gluco  pyranosyl-(1->4)-2-acetamido-2-  deoxy-D-glucopyranose | C22 H38 N2 O16 | 0.335800488 | -1.574324 | 8.41E-05 | 0.916667 | 1.031701 | down |
| Com_6815_neg | DEXAMETHASONE PHENYL  PROPIONATE | C31 H37 F O6 | 0.344938083 | -1.535591 | 0.000273 | 0.881944 | 1.220926 | down |
| Com_6819_neg | PALGLY | C18 H35 N O3 | 0.126714553 | -2.980346 | 1.82E-10 | 1 | 1.943867 | down |
| Com_6859_neg | (3R,4S,5S,6S)-6-Carboxy-N-[2-(diphen  ylmethoxy)ethyl]-3,4,5-trihydroxy-N,N  -dimethyltetrahydro-2H-pyran-2-aminium  (non-preferred name) | C23 H30 N O7 | 0.268513134 | -1.896935 | 7.73E-05 | 0.944444 | 1.173443 | down |
| Com_6882_neg | Methyl (1aR,2E,4S,5S,5aS,8aR,9E,10aS)  -4-acetoxy-5-{[(2R,3S)-3-acetoxy-2-hydro  xy-2-methylbutanoyl]oxy}-10-methyl-6-  methylene-7-oxo-1a,4,5,5a,6,7,8a,10a-octah  ydrooxireno[7,8]cyclodeca[1,2-b]furan-  3-carboxylate | C25 H30 O12 | 0.201243045 | -2.312989 | 1.23E-08 | 0.993056 | 1.484795 | down |
| Com_6883_neg | Roxatidine | C17 H26 N2 O3 | 0.323787104 | -1.626883 | 1.35E-05 | 0.958333 | 1.081154 | down |
| Com_6906_neg | Selenocystathionine | C7 H14 N2 O4 Se | 0.15945239 | -2.648802 | 8.37E-07 | 0.979167 | 1.581695 | down |
| Com_6924_neg | (S)-2-methylbutanal | C5 H10 O | 0.168078503 | -2.572793 | 2.47E-10 | 1 | 1.66594 | down |
| Com_693_neg | (3alpha,5beta,7alpha,12beta)-7,12-Dihy  droxy-24-oxo-24-[(2-sulfoethyl)  amino]cholane-3-sulfonic acid | C26 H45 N O9 S2 | 0.222269919 | -2.169615 | 5.77E-05 | 0.930556 | 1.399831 | down |
| Com_6954_neg | (1'R,2R,4S,4'S,5S,6R,8'R,10'E,12'S,  13'S,14'E,16'E,20'R,21'R,24'S)-4,24'-  Dihydroxy-6-isopropyl-21'-methoxy-  5,11',13',22'-tetramethyl-2'-oxo-3,4,5,6  -tetrahydrospiro[pyran-2,6'-[3,7,19]  trioxatetracyclo[15.6.1.1~4,8~.0~20,  24~]pentacosa[10,14,16,22]tetraen]-12'  -yl 2,6-dideoxy-4-O-(2,6-dideoxy-3-O-  methyl-alpha-L-arabino-hexopyranosyl)-3  -O-methyl-alpha-L-arabino-hexopyranoside | C48 H74 O15 | 0.270483039 | -1.88639 | 0.000876 | 0.854167 | 1.335553 | down |
| Com_7024_neg | 12-Hydroxylauric acid | C12 H24 O3 | 0.338943846 | -1.560882 | 2.92E-05 | 0.909722 | 1.136301 | down |
| Com_7053_neg | cefluprenam | C20 H25 F N8 O6 S2 | 0.194845057 | -2.359601 | 5.17E-08 | 1 | 1.510431 | down |
| Com_7159_neg | Glutathionylspermine | C20 H41 N7 O5 S | 0.274706669 | -1.864036 | 1.48E-05 | 0.965278 | 1.132343 | down |
| Com_715_neg | Isoniazid | C6 H7 N3 O | 0.167542164 | -2.577404 | 4.99E-05 | 0.930556 | 1.654772 | down |
| Com_7224_neg | 3-Indolecarboxylic acid | C9 H7 N O2 | 0.346792302 | -1.527856 | 0.001364 | 0.881944 | 1.115545 | down |
| Com_7262_neg | undecenal | C11 H20 O | 0.247638147 | -2.013695 | 4.20E-05 | 0.951389 | 1.302854 | down |
| Com_7370_neg | tenivastatin | C25 H40 O6 | 0.189635882 | -2.398696 | 5.12E-06 | 0.958333 | 1.692789 | down |
| Com_7382_neg | Dixyrazine | C24 H33 N3 O2 S | 0.282268977 | -1.824858 | 1.33E-06 | 0.965278 | 1.171363 | down |
| Com_7387_neg | Hypericin | C30 H16 O8 | 0.265837507 | -1.911383 | 6.39E-06 | 0.958333 | 1.260431 | down |
| Com_7400_neg | Apixaban | C25 H25 N5 O4 | 0.198953837 | -2.329494 | 2.52E-06 | 0.972222 | 1.463012 | down |
| Com_7407_neg | 3-O-(3-Methylbutanoyl)-beta-D-  fructofuranosyl 2-O-acetyl-3-O-(2-  methylbutanoyl)-4-O-(3-methylbu  tanoyl)-alpha-D-glucopyranoside | C29 H48 O15 | 0.249935679 | -2.000371 | 1.95E-05 | 0.972222 | 1.217305 | down |
| Com_7420_neg | 2,3-Naphthalenediol | C10 H8 O2 | 0.225049479 | -2.151686 | 2.06E-05 | 0.944444 | 1.500338 | down |
| Com_7429_neg | BL-P1780 | C21 H27 N3 O6 S | 0.175190426 | -2.513004 | 1.59E-07 | 1 | 1.648382 | down |
| Com_7456_neg | Deserpidine | C32 H38 N2 O8 | 0.220440882 | -2.181536 | 0.0005 | 0.895833 | 1.317833 | down |
| Com_7502_neg | Methyl 3-isobutyryl-4-methyl-1,5-bis  (3-methyl-2-buten-1-yl)-4-(4-methyl-3  -penten-1-yl)-2-oxocyclohexanecarboxylate | C29 H46 O4 | 0.296690152 | -1.752971 | 1.72E-07 | 0.979167 | 1.148057 | down |
| Com_7510_neg | 1,2,4-Trithiolane | C2 H4 S3 | 0.192244282 | -2.378987 | 0.004382 | 0.909722 | 1.193012 | down |
| Com_7519_neg | ISOFENPHOS | C15 H24 N O4 P S | 0.334571596 | -1.579613 | 9.41E-05 | 0.923611 | 1.007324 | down |
| Com_7541_neg | isomigrastatin | C27 H39 N O7 | 0.321825283 | -1.63565 | 9.18E-07 | 0.972222 | 1.050883 | down |
| Com_7542_neg | N-acetylleukotriene E4 | C25 H39 N O6 S | 0.219633377 | -2.186831 | 3.77E-07 | 1 | 1.325685 | down |
| Com_7553_neg | Chaparrin | C20 H28 O7 | 0.236382294 | -2.080806 | 5.30E-09 | 1 | 1.34293 | down |
| Com_7555_neg | Cabergoline | C26 H37 N5 O2 | 0.283307235 | -1.819561 | 2.20E-06 | 0.958333 | 1.217792 | down |
| Com_7568_neg | octocrylene | C24 H27 N O2 | 0.28935449 | -1.78909 | 2.65E-07 | 0.993056 | 1.287892 | down |
| Com_7735_neg | ACONINE | C25 H41 N O9 | 0.188252614 | -2.409258 | 9.63E-08 | 1 | 1.590106 | down |
| Com_7762_neg | (2R,3S)-3-Hydroxy-8-methyl-8-  azabicyclo[3.2.1]octane-2-carboxylic acid | C9 H15 N O3 | 0.410653232 | -1.284007 | 0.002012 | 0.840278 | 1.013857 | down |
| Com_7766_neg | Valerenic acid | C15 H22 O2 | 0.169134304 | -2.563759 | 8.84E-07 | 0.972222 | 1.577022 | down |
| Com_779_neg | Leukotriene E4 | C23 H37 N O5 S | 0.239210294 | -2.063649 | 9.13E-05 | 0.902778 | 1.265528 | down |
| Com_7879_neg | dimethylsulfate | C2 H6 O4 S | 0.3039315 | -1.718182 | 7.61E-06 | 0.951389 | 1.085033 | down |
| Com_7885_neg | Cilostazol | C20 H27 N5 O2 | 0.060391736 | -4.049505 | 3.08E-09 | 1 | 2.456307 | down |
| Com_7935_neg | Capryloylglycine | C10 H19 N O3 | 0.148984427 | -2.746767 | 1.25E-06 | 1 | 1.692782 | down |
| Com_7940_neg | Alosetron | C17 H18 N4 O | 0.221943894 | -2.171733 | 2.49E-07 | 0.993056 | 1.373179 | down |
| Com_7999_neg | Golotimod | C16 H19 N3 O5 | 0.213002972 | -2.231055 | 5.09E-05 | 0.930556 | 1.464062 | down |
| Com_8005_neg | Lysophosphatidylinositol | C25 H49 O12 P | 0.173474469 | -2.527205 | 1.30E-05 | 0.972222 | 1.475887 | down |
| Com_8024_neg | Buspirone | C21 H31 N5 O2 | 0.083614698 | -3.5801 | 3.62E-08 | 1 | 2.150312 | down |
| Com_8057_neg | 1-Methyl-3-(1,3-thiazol-2-yl)-1H-indole | C12 H10 N2 S | 0.106253294 | -3.234421 | 7.88E-10 | 1 | 2.060048 | down |
| Com_8081_neg | 6-O-Phosphonohex-2-ulofuranose | C6 H13 O9 P | 0.273732045 | -1.869164 | 1.59E-06 | 0.979167 | 1.13943 | down |
| Com_8145_neg | 7268 | C16 H30 O6 | 0.37351391 | -1.420766 | 0.001241 | 0.868056 | 1.115127 | down |
| Com_8164_neg | (+)-Simvastatin | C25 H38 O5 | 0.314879015 | -1.66713 | 0.000131 | 0.909722 | 1.080358 | down |
| Com_826_neg | Methyl undecenate | C12 H22 O2 | 0.433823059 | -1.204821 | 0.002334 | 0.826389 | 1.025005 | down |
| Com_8282_neg | 1-hexadecanal | C16 H32 O | 0.303638073 | -1.719575 | 9.33E-05 | 0.965278 | 1.323615 | down |
| Com_8300_neg | Ulimorelin | C30 H39 F N4 O4 | 0.158707782 | -2.655555 | 3.61E-09 | 1 | 1.712227 | down |
| Com_8339_neg | EPERVUDINE | C12 H18 N2 O5 | 0.26918549 | -1.893327 | 0.001263 | 0.868056 | 1.222067 | down |
| Com_8357_neg | Pramiracetam | C14 H27 N3 O2 | 0.268621059 | -1.896356 | 0.000322 | 0.9375 | 1.193787 | down |
| Com_8462_neg | (2S,4E)-2-Amino-5-{N'-[(1Z,2S,3R)-  1,2-dihydroxy-3-(1H-indol-3-yl)buty  lidene]carbamimidamido}-4-pentenoic  acid | C18 H23 N5 O4 | 0.301516945 | -1.729689 | 0.000283 | 0.916667 | 1.185788 | down |
| Com_8468_neg | (1R,4R,13R,14S)-13-Hydroxy-9-(1-  hydroxyethyl)-3-[5-(3-hydroxyphenyl)-  5-methoxy-2-pentanyl]-4,14,16,16-tetram  ethyl-2,6,10,17-tetraoxatricyclo[11.3.1.1  ~1,5~]octadecane-7,11-dione | C32 H48 O10 | 0.232975818 | -2.101748 | 7.57E-05 | 0.916667 | 1.382987 | down |
| Com_8523_neg | Myriocin | C21 H39 N O6 | 0.15773794 | -2.664398 | 3.30E-06 | 1 | 1.592067 | down |
| Com_8536_neg | (3alpha,4alpha,5alpha,9beta,11alpha,  13alpha,14beta,16beta,17Z)-16-Acetoxy  -3,11-dihydroxy-4,8,14-trimethyl-22-oxo  -18-norcholesta-17,24-dien-21-oic acid | C31 H46 O7 | 0.248032354 | -2.0114 | 1.65E-06 | 0.958333 | 1.30835 | down |
| Com_8553_neg | Agavoside A | C33 H52 O9 | 0.304006055 | -1.717828 | 3.49E-06 | 0.979167 | 1.033968 | down |
| Com_8575_neg | Cyanotriphenylborate | C19 H15 B N | 0.350909683 | -1.510828 | 0.000233 | 0.902778 | 1.064309 | down |
| Com_8613_neg | Tetrandrine | C38 H42 N2 O6 | 0.200548724 | -2.317975 | 4.56E-07 | 0.993056 | 1.479407 | down |
| Com_8620_neg | Rupatadine | C26 H26 Cl N3 | 0.403283516 | -1.310134 | 0.006037 | 0.819444 | 1.086511 | down |
| Com_8721_neg | Diethyl phosphate | C4 H11 O4 P | 0.226942288 | -2.139603 | 5.63E-05 | 0.986111 | 1.194551 | down |
| Com_8722_neg | (5beta)-24-Oxo-24-[(2-sulfoethyl)amino]  cholan-3-yl beta-D-glucopyranosiduronic  acid | C32 H53 N O11 S | 0.340805613 | -1.552979 | 0.000506 | 0.881944 | 1.030648 | down |
| Com_8778_neg | riboprine | C15 H21 N5 O4 | 0.219997407 | -2.184442 | 3.50E-05 | 0.958333 | 1.46959 | down |
| Com_8780_neg | 1-(4-Hydroxy-3,5-dimethoxyphenyl)-7-  (4-hydroxy-3-methoxyphenyl)-3,5-  heptanediol | C22 H30 O7 | 0.353784961 | -1.499055 | 0.000312 | 0.875 | 1.227329 | down |
| Com_8821_neg | Mucronine B | C28 H36 N4 O4 | 0.301875978 | -1.727972 | 1.63E-05 | 0.965278 | 1.003439 | down |
| Com_8923_neg | Cys-tyr | C12 H16 N2 O4 S | 0.104147722 | -3.263297 | 1.19E-09 | 1 | 2.15379 | down |
| Com_9090_neg | Spenolimycin | C15 H26 N2 O7 | 0.204555589 | -2.289435 | 3.05E-08 | 1 | 1.441846 | down |
| Com_9094_neg | Apiin | C26 H28 O14 | 0.242348664 | -2.044844 | 3.09E-05 | 0.930556 | 1.467515 | down |
| Com_9145_neg | Pefloxacin | C17 H20 F N3 O3 | 0.358574889 | -1.479654 | 3.88E-06 | 0.972222 | 1.020026 | down |
| Com_9162_neg | 4-{[3-(Hydroxymethyl)phenyl]amino}-  N-(isopropylcarbamoyl)-3-pyridinesul  fonamide | C16 H20 N4 O4 S | 0.312222077 | -1.679356 | 1.23E-07 | 1 | 1.08652 | down |
| Com_9167_neg | oxamniquine | C14 H21 N3 O3 | 0.267971326 | -1.899849 | 0.000513 | 0.902778 | 1.262152 | down |
| Com_9171_neg | Androsterone glucuronide | C25 H38 O8 | 0.112172203 | -3.156213 | 4.77E-10 | 1 | 2.057225 | down |
| Com_9210_neg | 2-methoxyestrone 3-glucosiduronic acid | C25 H32 O9 | 0.175361774 | -2.511594 | 9.00E-07 | 0.979167 | 1.584025 | down |
| Com_9260_neg | (7R,14bR,15aS)-10-Acetyl-7-methyl-  14b-(2-methyl-3-buten-2-yl)-10,14b,  15,15a-tetrahydroindolo[3'',2'':4',5']  pyrrolo[2',1':3,4]pyrazino[2,1-b]  quinazoline-5,8(7H,9aH)-dione | C28 H28 N4 O3 | 0.282589213 | -1.823222 | 0.001891 | 0.847222 | 1.336738 | down |
| Com_926_neg | AMADINONE ACETATE | C22 H27 Cl O4 | 0.363358891 | -1.460533 | 2.49E-05 | 0.916667 | 1.073471 | down |
| Com_9333_neg | 2-Hydroxy-2-(4-methyl-2-oxocyclohexyl)  propyl hexopyranoside | C16 H28 O8 | 0.27722914 | -1.850849 | 0.003015 | 0.861111 | 1.104142 | down |
| Com_9337_neg | Bezafibrate | C19 H20 Cl N O4 | 0.135978798 | -2.878546 | 0.000123 | 0.958333 | 1.524164 | down |
| Com_935_neg | Tixocortol | C21 H30 O4 S | 0.132431215 | -2.916685 | 7.77E-10 | 1 | 1.94572 | down |
| Com_937_neg | Tetraacetylethylenediamine | C10 H16 N2 O4 | 0.19290051 | -2.374071 | 4.34E-05 | 0.930556 | 1.637653 | down |
| Com_9449_neg | triafungin | C13 H10 N4 | 0.132928182 | -2.911281 | 1.30E-07 | 1 | 1.780199 | down |
| Com_9457_neg | BL-P1761 | C21 H27 N3 O5 S | 0.292003669 | -1.775942 | 1.94E-06 | 1 | 1.067666 | down |
| Com_9507_neg | 2,5-Dihydroxy-3-(1H-indol-3-yl)-  6-[2-(2-methyl-3-buten-2-yl)-1H-  indol-3-yl]-1,4-benzoquinone | C27 H22 N2 O4 | 0.350789097 | -1.511324 | 0.000661 | 0.888889 | 1.173449 | down |
| Com_9568_neg | piroximone | C11 H11 N3 O2 | 0.094365059 | -3.405603 | 4.34E-10 | 1 | 2.166097 | down |
| Com_9642_neg | Methylprednisolone aceponate | C27 H36 O7 | 0.250174494 | -1.998993 | 8.54E-06 | 0.972222 | 1.355895 | down |
| Com_9649_neg | 3'-dephospho-CoA | C21 H35 N7 O13 P2 S | 0.094488459 | -3.403718 | 4.87E-13 | 1 | 2.266217 | down |
| Com_9672_neg | FLUOCINONIDE | C26 H32 F2 O7 | 0.412578768 | -1.277259 | 0.00488 | 0.840278 | 1.097959 | down |
| Com_9865_neg | sufotidine | C20 H31 N5 O3 S | 0.26869387 | -1.895965 | 1.14E-06 | 0.979167 | 1.199106 | down |
| Com_9931_neg | Omacetaxine mepesuccinate | C29 H39 N O9 | 0.233422171 | -2.098986 | 1.34E-06 | 0.972222 | 1.453404 | down |
| Com_9938_neg | epothilone C | C26 H39 N O5 S | 0.174486199 | -2.518815 | 1.39E-10 | 1 | 1.685008 | down |
| Com_9940_neg | Tenofovir alafenamide | C21 H29 N6 O5 P | 0.185351844 | -2.431662 | 0.000195 | 0.930556 | 1.334173 | down |

| **Supplementary Table 16. The KEGG pathway enrichment of the differential matabolites in DI contents of SBM20_N vs. FM in positive mode （n=2）** | | | | | | | | |
| --- | --- | --- | --- | --- | --- | --- | --- | --- |
|
| MapID | MapTitle | Pvalue | Adjusted Pv | x1 | x2 | n | N | EnrichDirect |
| map00943 | Isoflavonoid biosynthesis | 0.001497825 | 0.161765052 | 6 | 7 | 54 | 205 | Over |
| map00360 | Phenylalanine metabolism | 0.004426003 | 0.189261177 | 4 | 4 | 54 | 205 | Over |
| map00960 | Tropane, piperidine and pyridine alkaloid biosynthesis | 0.005257255 | 0.189261177 | 5 | 6 | 54 | 205 | Over |
| map01061 | Biosynthesis of phenylpropanoids | 0.031158717 | 0.694196763 | 5 | 8 | 54 | 205 | Over |
| map01063 | Biosynthesis of alkaloids derived from shikimate pathway | 0.055954933 | 0.694196763 | 5 | 9 | 54 | 205 | Over |
| map01064 | Biosynthesis of alkaloids derived from ornithine, lysine and nicotinic acid | 0.080007262 | 0.694196763 | 4 | 7 | 54 | 205 | Over |
| map00591 | Linoleic acid metabolism | 0.170247578 | 0.694196763 | 2 | 3 | 54 | 205 | Over |
| map00310 | Lysine degradation | 0.188377142 | 0.694196763 | 3 | 6 | 54 | 205 | Over |
| map00020 | Citrate cycle (TCA cycle) | 0.263414634 | 0.694196763 | 1 | 1 | 54 | 205 | Over |
| map00062 | Fatty acid elongation | 0.263414634 | 0.694196763 | 1 | 1 | 54 | 205 | Over |
| map00130 | Ubiquinone and other terpenoid-quinone biosynthesis | 0.263414634 | 0.694196763 | 1 | 1 | 54 | 205 | Over |
| map00190 | Oxidative phosphorylation | 0.263414634 | 0.694196763 | 1 | 1 | 54 | 205 | Over |
| map00365 | Furfural degradation | 0.263414634 | 0.694196763 | 1 | 1 | 54 | 205 | Over |
| map00620 | Pyruvate metabolism | 0.263414634 | 0.694196763 | 1 | 1 | 54 | 205 | Over |
| map00640 | Propanoate metabolism | 0.263414634 | 0.694196763 | 1 | 1 | 54 | 205 | Over |
| map00720 | Carbon fixation pathways in prokaryotes | 0.263414634 | 0.694196763 | 1 | 1 | 54 | 205 | Over |
| map00944 | Flavone and flavonol biosynthesis | 0.263414634 | 0.694196763 | 1 | 1 | 54 | 205 | Over |
| map00945 | Stilbenoid, diarylheptanoid and gingerol biosynthesis | 0.263414634 | 0.694196763 | 1 | 1 | 54 | 205 | Over |
| map01052 | Type I polyketide structures | 0.263414634 | 0.694196763 | 1 | 1 | 54 | 205 | Over |
| map01066 | Biosynthesis of alkaloids derived from terpenoid and polyketide | 0.263414634 | 0.694196763 | 1 | 1 | 54 | 205 | Over |
| map03320 | PPAR signaling pathway | 0.263414634 | 0.694196763 | 1 | 1 | 54 | 205 | Over |
| map04114 | Oocyte meiosis | 0.263414634 | 0.694196763 | 1 | 1 | 54 | 205 | Over |
| map04211 | Longevity regulating pathway | 0.263414634 | 0.694196763 | 1 | 1 | 54 | 205 | Over |
| map04218 | Cellular senescence | 0.263414634 | 0.694196763 | 1 | 1 | 54 | 205 | Over |
| map04810 | Regulation of actin cytoskeleton | 0.263414634 | 0.694196763 | 1 | 1 | 54 | 205 | Over |
| map04911 | Insulin secretion | 0.263414634 | 0.694196763 | 1 | 1 | 54 | 205 | Over |
| map04914 | Progesterone-mediated oocyte maturation | 0.263414634 | 0.694196763 | 1 | 1 | 54 | 205 | Over |
| map04927 | Cortisol synthesis and secretion | 0.263414634 | 0.694196763 | 1 | 1 | 54 | 205 | Over |
| map04934 | Cushing's syndrome | 0.263414634 | 0.694196763 | 1 | 1 | 54 | 205 | Over |
| map04970 | Salivary secretion | 0.263414634 | 0.694196763 | 1 | 1 | 54 | 205 | Over |
| map04972 | Pancreatic secretion | 0.263414634 | 0.694196763 | 1 | 1 | 54 | 205 | Over |
| map05033 | Nicotine addiction | 0.263414634 | 0.694196763 | 1 | 1 | 54 | 205 | Over |
| map05224 | Breast cancer | 0.263414634 | 0.694196763 | 1 | 1 | 54 | 205 | Over |
| map05323 | Rheumatoid arthritis | 0.263414634 | 0.694196763 | 1 | 1 | 54 | 205 | Over |
| map07034 | Eicosanoids | 0.263414634 | 0.694196763 | 1 | 1 | 54 | 205 | Over |
| map07229 | Angiotensin receptor and endothelin receptor antagonists | 0.263414634 | 0.694196763 | 1 | 1 | 54 | 205 | Over |
| map00950 | Isoquinoline alkaloid biosynthesis | 0.28365139 | 0.694196763 | 2 | 4 | 54 | 205 | Over |
| map01062 | Biosynthesis of terpenoids and steroids | 0.28365139 | 0.694196763 | 2 | 4 | 54 | 205 | Over |
| map01230 | Biosynthesis of amino acids | 0.28365139 | 0.694196763 | 2 | 4 | 54 | 205 | Over |
| map04024 | cAMP signaling pathway | 0.28365139 | 0.694196763 | 2 | 4 | 54 | 205 | Over |
| map04726 | Serotonergic synapse | 0.28365139 | 0.694196763 | 2 | 4 | 54 | 205 | Over |
| map00140 | Steroid hormone biosynthesis | 0.295676399 | 0.694196763 | 4 | 10 | 54 | 205 | Over |
| map04976 | Bile secretion | 0.295676399 | 0.694196763 | 4 | 10 | 54 | 205 | Over |
| map00040 | Pentose and glucuronate interconversions | 0.458393113 | 0.728036121 | 1 | 2 | 54 | 205 | Over |
| map00073 | Cutin, suberine and wax biosynthesis | 0.458393113 | 0.728036121 | 1 | 2 | 54 | 205 | Over |
| map00300 | Lysine biosynthesis | 0.458393113 | 0.728036121 | 1 | 2 | 54 | 205 | Over |
| map00400 | Phenylalanine, tyrosine and tryptophan biosynthesis | 0.458393113 | 0.728036121 | 1 | 2 | 54 | 205 | Over |
| map00460 | Cyanoamino acid metabolism | 0.458393113 | 0.728036121 | 1 | 2 | 54 | 205 | Over |
| map00564 | Glycerophospholipid metabolism | 0.458393113 | 0.728036121 | 1 | 2 | 54 | 205 | Over |
| map00590 | Arachidonic acid metabolism | 0.458393113 | 0.728036121 | 1 | 2 | 54 | 205 | Over |
| map00621 | Dioxin degradation | 0.458393113 | 0.728036121 | 1 | 2 | 54 | 205 | Over |
| map00630 | Glyoxylate and dicarboxylate metabolism | 0.458393113 | 0.728036121 | 1 | 2 | 54 | 205 | Over |
| map00750 | Vitamin B6 metabolism | 0.458393113 | 0.728036121 | 1 | 2 | 54 | 205 | Over |
| map00920 | Sulfur metabolism | 0.458393113 | 0.728036121 | 1 | 2 | 54 | 205 | Over |
| map00981 | Insect hormone biosynthesis | 0.458393113 | 0.728036121 | 1 | 2 | 54 | 205 | Over |
| map00983 | Drug metabolism - other enzymes | 0.458393113 | 0.728036121 | 1 | 2 | 54 | 205 | Over |
| map01212 | Fatty acid metabolism | 0.458393113 | 0.728036121 | 1 | 2 | 54 | 205 | Over |
| map04725 | Cholinergic synapse | 0.458393113 | 0.728036121 | 1 | 2 | 54 | 205 | Over |
| map04917 | Prolactin signaling pathway | 0.458393113 | 0.728036121 | 1 | 2 | 54 | 205 | Over |
| map04925 | Aldosterone synthesis and secretion | 0.458393113 | 0.728036121 | 1 | 2 | 54 | 205 | Over |
| map04971 | Gastric acid secretion | 0.458393113 | 0.728036121 | 1 | 2 | 54 | 205 | Over |
| map04978 | Mineral absorption | 0.458393113 | 0.728036121 | 1 | 2 | 54 | 205 | Over |
| map07225 | Glucocorticoid and mineralocorticoid receptor agonists/antagonists | 0.458393113 | 0.728036121 | 1 | 2 | 54 | 205 | Over |
| map07226 | Progesterone, androgen and estrogen receptor agonists/antagonists | 0.458393113 | 0.728036121 | 1 | 2 | 54 | 205 | Over |
| map01110 | Biosynthesis of secondary metabolites | 0.469683289 | 0.735156452 | 12 | 37 | 54 | 205 | Over |
| map04750 | Inflammatory mediator regulation of TRP channels | 0.608751702 | 0.913127553 | 2 | 5 | 54 | 205 | Over |
| map05200 | Pathways in cancer | 0.608751702 | 0.913127553 | 2 | 5 | 54 | 205 | Over |
| map05215 | Prostate cancer | 0.608751702 | 0.913127553 | 2 | 5 | 54 | 205 | Over |
| map04080 | Neuroactive ligand-receptor interaction | 0.725146694 | 1 | 3 | 10 | 54 | 205 | Over |
| map04974 | Protein digestion and absorption | 0.725146694 | 1 | 3 | 10 | 54 | 205 | Over |
| map01130 | Biosynthesis of antibiotics | 1 | 1 | 4 | 15 | 54 | 205 | Over |
| map00061 | Fatty acid biosynthesis | 1 | 1 | 1 | 3 | 54 | 205 | Over |
| map00071 | Fatty acid degradation | 1 | 1 | 1 | 3 | 54 | 205 | Over |
| map00100 | Steroid biosynthesis | 1 | 1 | 1 | 3 | 54 | 205 | Over |
| map00650 | Butanoate metabolism | 1 | 1 | 1 | 3 | 54 | 205 | Over |
| map01060 | Biosynthesis of plant secondary metabolites | 1 | 1 | 5 | 18 | 54 | 205 | Over |
| map04212 | Longevity regulating pathway - worm | 1 | 1 | 1 | 3 | 54 | 205 | Over |
| map04216 | Ferroptosis | 1 | 1 | 1 | 3 | 54 | 205 | Over |
| map04721 | Synaptic vesicle cycle | 1 | 1 | 1 | 3 | 54 | 205 | Over |
| map04723 | Retrograde endocannabinoid signaling | 1 | 1 | 1 | 3 | 54 | 205 | Over |
| map04742 | Taste transduction | 1 | 1 | 1 | 3 | 54 | 205 | Over |

| **Supplementary Table 17. The KEGG pathway enrichment of the differential matabolites in DI contents of SBM40_N vs. FM in positive mode (n=12)** | | | | | | | | |
| --- | --- | --- | --- | --- | --- | --- | --- | --- |
|
| MapID | MapTitle | Pvalue | AdjustedPv | x1 | x2 | n | N | EnrichDirect |
| map00943 | Isoflavonoid biosynthesis | 0.006943345 | 0.655389457 | 6 | 7 | 70 | 205 | Over |
| map00591 | Linoleic acid metabolism | 0.038687973 | 0.655389457 | 3 | 3 | 70 | 205 | Over |
| map04976 | Bile secretion | 0.09356001 | 0.655389457 | 6 | 10 | 70 | 205 | Over |
| map00590 | Arachidonic acid metabolism | 0.115494978 | 0.655389457 | 2 | 2 | 70 | 205 | Over |
| map00680 | Methane metabolism | 0.115494978 | 0.655389457 | 2 | 2 | 70 | 205 | Over |
| map04917 | Prolactin signaling pathway | 0.115494978 | 0.655389457 | 2 | 2 | 70 | 205 | Over |
| map04925 | Aldosterone synthesis and secretion | 0.115494978 | 0.655389457 | 2 | 2 | 70 | 205 | Over |
| map00950 | Isoquinoline alkaloid biosynthesis | 0.116255444 | 0.655389457 | 3 | 4 | 70 | 205 | Over |
| map00982 | Drug metabolism - cytochrome P450 | 0.116255444 | 0.655389457 | 3 | 4 | 70 | 205 | Over |
| map01062 | Biosynthesis of terpenoids and steroids | 0.116255444 | 0.655389457 | 3 | 4 | 70 | 205 | Over |
| map04913 | Ovarian steroidogenesis | 0.116255444 | 0.655389457 | 3 | 4 | 70 | 205 | Over |
| map00071 | Fatty acid degradation | 0.269108989 | 0.655389457 | 2 | 3 | 70 | 205 | Over |
| map00100 | Steroid biosynthesis | 0.269108989 | 0.655389457 | 2 | 3 | 70 | 205 | Over |
| map00633 | Nitrotoluene degradation | 0.269108989 | 0.655389457 | 2 | 3 | 70 | 205 | Over |
| map04216 | Ferroptosis | 0.269108989 | 0.655389457 | 2 | 3 | 70 | 205 | Over |
| map04723 | Retrograde endocannabinoid signaling | 0.269108989 | 0.655389457 | 2 | 3 | 70 | 205 | Over |
| map00140 | Steroid hormone biosynthesis | 0.314439597 | 0.655389457 | 5 | 10 | 70 | 205 | Over |
| map00940 | Phenylpropanoid biosynthesis | 0.340378925 | 0.655389457 | 3 | 5 | 70 | 205 | Over |
| map04750 | Inflammatory mediator regulation of TRP channels | 0.340378925 | 0.655389457 | 3 | 5 | 70 | 205 | Over |
| map05200 | Pathways in cancer | 0.340378925 | 0.655389457 | 3 | 5 | 70 | 205 | Over |
| map05215 | Prostate cancer | 0.340378925 | 0.655389457 | 3 | 5 | 70 | 205 | Over |
| map00020 | Citrate cycle (TCA cycle) | 0.341463415 | 0.655389457 | 1 | 1 | 70 | 205 | Over |
| map00062 | Fatty acid elongation | 0.341463415 | 0.655389457 | 1 | 1 | 70 | 205 | Over |
| map00130 | Ubiquinone and other terpenoid-quinone biosynthesis | 0.341463415 | 0.655389457 | 1 | 1 | 70 | 205 | Over |
| map00190 | Oxidative phosphorylation | 0.341463415 | 0.655389457 | 1 | 1 | 70 | 205 | Over |
| map00231 | Puromycin biosynthesis | 0.341463415 | 0.655389457 | 1 | 1 | 70 | 205 | Over |
| map00254 | Aflatoxin biosynthesis | 0.341463415 | 0.655389457 | 1 | 1 | 70 | 205 | Over |
| map00365 | Furfural degradation | 0.341463415 | 0.655389457 | 1 | 1 | 70 | 205 | Over |
| map00620 | Pyruvate metabolism | 0.341463415 | 0.655389457 | 1 | 1 | 70 | 205 | Over |
| map00640 | Propanoate metabolism | 0.341463415 | 0.655389457 | 1 | 1 | 70 | 205 | Over |
| map00720 | Carbon fixation pathways in prokaryotes | 0.341463415 | 0.655389457 | 1 | 1 | 70 | 205 | Over |
| map00902 | Monoterpenoid biosynthesis | 0.341463415 | 0.655389457 | 1 | 1 | 70 | 205 | Over |
| map00904 | Diterpenoid biosynthesis | 0.341463415 | 0.655389457 | 1 | 1 | 70 | 205 | Over |
| map00945 | Stilbenoid, diarylheptanoid and gingerol biosynthesis | 0.341463415 | 0.655389457 | 1 | 1 | 70 | 205 | Over |
| map01052 | Type I polyketide structures | 0.341463415 | 0.655389457 | 1 | 1 | 70 | 205 | Over |
| map01066 | Biosynthesis of alkaloids derived from terpenoid and polyketide | 0.341463415 | 0.655389457 | 1 | 1 | 70 | 205 | Over |
| map03320 | PPAR signaling pathway | 0.341463415 | 0.655389457 | 1 | 1 | 70 | 205 | Over |
| map04114 | Oocyte meiosis | 0.341463415 | 0.655389457 | 1 | 1 | 70 | 205 | Over |
| map04211 | Longevity regulating pathway | 0.341463415 | 0.655389457 | 1 | 1 | 70 | 205 | Over |
| map04217 | Necroptosis | 0.341463415 | 0.655389457 | 1 | 1 | 70 | 205 | Over |
| map04218 | Cellular senescence | 0.341463415 | 0.655389457 | 1 | 1 | 70 | 205 | Over |
| map04611 | Platelet activation | 0.341463415 | 0.655389457 | 1 | 1 | 70 | 205 | Over |
| map04666 | Fc gamma R-mediated phagocytosis | 0.341463415 | 0.655389457 | 1 | 1 | 70 | 205 | Over |
| map04730 | Long-term depression | 0.341463415 | 0.655389457 | 1 | 1 | 70 | 205 | Over |
| map04745 | Phototransduction - fly | 0.341463415 | 0.655389457 | 1 | 1 | 70 | 205 | Over |
| map04810 | Regulation of actin cytoskeleton | 0.341463415 | 0.655389457 | 1 | 1 | 70 | 205 | Over |
| map04911 | Insulin secretion | 0.341463415 | 0.655389457 | 1 | 1 | 70 | 205 | Over |
| map04912 | GnRH signaling pathway | 0.341463415 | 0.655389457 | 1 | 1 | 70 | 205 | Over |
| map04914 | Progesterone-mediated oocyte maturation | 0.341463415 | 0.655389457 | 1 | 1 | 70 | 205 | Over |
| map04921 | Oxytocin signaling pathway | 0.341463415 | 0.655389457 | 1 | 1 | 70 | 205 | Over |
| map04927 | Cortisol synthesis and secretion | 0.341463415 | 0.655389457 | 1 | 1 | 70 | 205 | Over |
| map04934 | Cushing's syndrome | 0.341463415 | 0.655389457 | 1 | 1 | 70 | 205 | Over |
| map04970 | Salivary secretion | 0.341463415 | 0.655389457 | 1 | 1 | 70 | 205 | Over |
| map04972 | Pancreatic secretion | 0.341463415 | 0.655389457 | 1 | 1 | 70 | 205 | Over |
| map05033 | Nicotine addiction | 0.341463415 | 0.655389457 | 1 | 1 | 70 | 205 | Over |
| map05224 | Breast cancer | 0.341463415 | 0.655389457 | 1 | 1 | 70 | 205 | Over |
| map05323 | Rheumatoid arthritis | 0.341463415 | 0.655389457 | 1 | 1 | 70 | 205 | Over |
| map07034 | Eicosanoids | 0.341463415 | 0.655389457 | 1 | 1 | 70 | 205 | Over |
| map07229 | Angiotensin receptor and endothelin receptor antagonists | 0.341463415 | 0.655389457 | 1 | 1 | 70 | 205 | Over |
| map01040 | Biosynthesis of unsaturated fatty acids | 0.449006497 | 0.809572321 | 4 | 8 | 70 | 205 | Over |
| map01061 | Biosynthesis of phenylpropanoids | 0.449006497 | 0.809572321 | 4 | 8 | 70 | 205 | Over |
| map01063 | Biosynthesis of alkaloids derived from shikimate pathway | 0.49443619 | 0.874144803 | 4 | 9 | 70 | 205 | Over |
| map00360 | Phenylalanine metabolism | 0.607159004 | 1 | 2 | 4 | 70 | 205 | Over |
| map04024 | cAMP signaling pathway | 0.607159004 | 1 | 2 | 4 | 70 | 205 | Over |
| map04726 | Serotonergic synapse | 0.607159004 | 1 | 2 | 4 | 70 | 205 | Over |
| map04080 | Neuroactive ligand-receptor interaction | 0.737815065 | 1 | 4 | 10 | 70 | 205 | Over |
| map01110 | Biosynthesis of secondary metabolites | 1 | 1 | 13 | 37 | 70 | 205 | Over |
| map00040 | Pentose and glucuronate interconversions | 1 | 1 | 1 | 2 | 70 | 205 | Over |
| map00052 | Galactose metabolism | 1 | 1 | 1 | 2 | 70 | 205 | Over |
| map00073 | Cutin, suberine and wax biosynthesis | 1 | 1 | 1 | 2 | 70 | 205 | Over |
| map00300 | Lysine biosynthesis | 1 | 1 | 1 | 2 | 70 | 205 | Over |
| map00330 | Arginine and proline metabolism | 1 | 1 | 3 | 8 | 70 | 205 | Over |
| map00564 | Glycerophospholipid metabolism | 1 | 1 | 1 | 2 | 70 | 205 | Over |
| map00630 | Glyoxylate and dicarboxylate metabolism | 1 | 1 | 1 | 2 | 70 | 205 | Over |
| map00750 | Vitamin B6 metabolism | 1 | 1 | 1 | 2 | 70 | 205 | Over |
| map00920 | Sulfur metabolism | 1 | 1 | 1 | 2 | 70 | 205 | Over |
| map00981 | Insect hormone biosynthesis | 1 | 1 | 1 | 2 | 70 | 205 | Over |
| map00983 | Drug metabolism - other enzymes | 1 | 1 | 1 | 2 | 70 | 205 | Over |
| map00984 | Steroid degradation | 1 | 1 | 1 | 2 | 70 | 205 | Over |
| map01212 | Fatty acid metabolism | 1 | 1 | 1 | 2 | 70 | 205 | Over |
| map01522 | Endocrine resistance | 1 | 1 | 1 | 2 | 70 | 205 | Over |
| map02060 | Phosphotransferase system (PTS) | 1 | 1 | 1 | 2 | 70 | 205 | Over |
| map04075 | Plant hormone signal transduction | 1 | 1 | 1 | 2 | 70 | 205 | Over |
| map04270 | Vascular smooth muscle contraction | 1 | 1 | 1 | 2 | 70 | 205 | Over |
| map04664 | Fc epsilon RI signaling pathway | 1 | 1 | 1 | 2 | 70 | 205 | Over |
| map04725 | Cholinergic synapse | 1 | 1 | 1 | 2 | 70 | 205 | Over |
| map04971 | Gastric acid secretion | 1 | 1 | 1 | 2 | 70 | 205 | Over |
| map07225 | Glucocorticoid and mineralocorticoid receptor agonists/antagonists | 1 | 1 | 1 | 2 | 70 | 205 | Over |
| map07226 | Progesterone, androgen and estrogen receptor agonists/antagonists | 1 | 1 | 1 | 2 | 70 | 205 | Over |

| **Supplementary Table 18. The KEGG pathway enrichment of the differential metabolites in DI contents of SBM20_N vs. FM in negative mode （n=12）** | | | | | | | | |
| --- | --- | --- | --- | --- | --- | --- | --- | --- |
|
| MapID | MapTitle | Pvalue | Adjusted Pv | x1 | x2 | n | N | EnrichDirect |
| map00361 | Chlorocyclohexane and chlorobenzene degradation | 0.006699075 | 0.661948573 | 4 | 4 | 46 | 157 | Over |
| map00350 | Tyrosine metabolism | 0.023227947 | 0.661948573 | 5 | 7 | 46 | 157 | Over |
| map00943 | Isoflavonoid biosynthesis | 0.026139526 | 0.661948573 | 4 | 5 | 46 | 157 | Over |
| map00627 | Aminobenzoate degradation | 0.061311395 | 0.661948573 | 4 | 6 | 46 | 157 | Over |
| map00362 | Benzoate degradation | 0.084517393 | 0.661948573 | 2 | 2 | 46 | 157 | Over |
| map00524 | Neomycin, kanamycin and gentamicin biosynthesis | 0.084517393 | 0.661948573 | 2 | 2 | 46 | 157 | Over |
| map00620 | Pyruvate metabolism | 0.084517393 | 0.661948573 | 2 | 2 | 46 | 157 | Over |
| map04723 | Retrograde endocannabinoid signaling | 0.084517393 | 0.661948573 | 2 | 2 | 46 | 157 | Over |
| map00261 | Monobactam biosynthesis | 0.150467995 | 0.661948573 | 3 | 5 | 46 | 157 | Over |
| map00410 | beta-Alanine metabolism | 0.150467995 | 0.661948573 | 3 | 5 | 46 | 157 | Over |
| map00250 | Alanine, aspartate and glutamate metabolism | 0.195468238 | 0.661948573 | 4 | 7 | 46 | 157 | Over |
| map01063 | Biosynthesis of alkaloids derived from shikimate pathway | 0.195468238 | 0.661948573 | 4 | 7 | 46 | 157 | Over |
| map00040 | Pentose and glucuronate interconversions | 0.205568112 | 0.661948573 | 2 | 3 | 46 | 157 | Over |
| map00140 | Steroid hormone biosynthesis | 0.205568112 | 0.661948573 | 2 | 3 | 46 | 157 | Over |
| map00520 | Amino sugar and nucleotide sugar metabolism | 0.205568112 | 0.661948573 | 2 | 3 | 46 | 157 | Over |
| map00590 | Arachidonic acid metabolism | 0.205568112 | 0.661948573 | 2 | 3 | 46 | 157 | Over |
| map00944 | Flavone and flavonol biosynthesis | 0.205568112 | 0.661948573 | 2 | 3 | 46 | 157 | Over |
| map01066 | Biosynthesis of alkaloids derived from terpenoid and polyketide | 0.205568112 | 0.661948573 | 2 | 3 | 46 | 157 | Over |
| map00970 | Aminoacyl-tRNA biosynthesis | 0.233878898 | 0.661948573 | 4 | 8 | 46 | 157 | Over |
| map01061 | Biosynthesis of phenylpropanoids | 0.233878898 | 0.661948573 | 4 | 8 | 46 | 157 | Over |
| map00030 | Pentose phosphate pathway | 0.292993631 | 0.661948573 | 1 | 1 | 46 | 157 | Over |
| map00190 | Oxidative phosphorylation | 0.292993631 | 0.661948573 | 1 | 1 | 46 | 157 | Over |
| map00231 | Puromycin biosynthesis | 0.292993631 | 0.661948573 | 1 | 1 | 46 | 157 | Over |
| map00473 | D-Alanine metabolism | 0.292993631 | 0.661948573 | 1 | 1 | 46 | 157 | Over |
| map00500 | Starch and sucrose metabolism | 0.292993631 | 0.661948573 | 1 | 1 | 46 | 157 | Over |
| map00540 | Lipopolysaccharide biosynthesis | 0.292993631 | 0.661948573 | 1 | 1 | 46 | 157 | Over |
| map00550 | Peptidoglycan biosynthesis | 0.292993631 | 0.661948573 | 1 | 1 | 46 | 157 | Over |
| map00622 | Xylene degradation | 0.292993631 | 0.661948573 | 1 | 1 | 46 | 157 | Over |
| map00710 | Carbon fixation in photosynthetic organisms | 0.292993631 | 0.661948573 | 1 | 1 | 46 | 157 | Over |
| map00740 | Riboflavin metabolism | 0.292993631 | 0.661948573 | 1 | 1 | 46 | 157 | Over |
| map00902 | Monoterpenoid biosynthesis | 0.292993631 | 0.661948573 | 1 | 1 | 46 | 157 | Over |
| map00910 | Nitrogen metabolism | 0.292993631 | 0.661948573 | 1 | 1 | 46 | 157 | Over |
| map00941 | Flavonoid biosynthesis | 0.292993631 | 0.661948573 | 1 | 1 | 46 | 157 | Over |
| map00950 | Isoquinoline alkaloid biosynthesis | 0.292993631 | 0.661948573 | 1 | 1 | 46 | 157 | Over |
| map00965 | Betalain biosynthesis | 0.292993631 | 0.661948573 | 1 | 1 | 46 | 157 | Over |
| map01055 | Biosynthesis of vancomycin group antibiotics | 0.292993631 | 0.661948573 | 1 | 1 | 46 | 157 | Over |
| map01057 | Biosynthesis of type II polyketide products | 0.292993631 | 0.661948573 | 1 | 1 | 46 | 157 | Over |
| map01059 | Biosynthesis of enediyne antibiotics | 0.292993631 | 0.661948573 | 1 | 1 | 46 | 157 | Over |
| map04075 | Plant hormone signal transduction | 0.292993631 | 0.661948573 | 1 | 1 | 46 | 157 | Over |
| map04217 | Necroptosis | 0.292993631 | 0.661948573 | 1 | 1 | 46 | 157 | Over |
| map04611 | Platelet activation | 0.292993631 | 0.661948573 | 1 | 1 | 46 | 157 | Over |
| map04666 | Fc gamma R-mediated phagocytosis | 0.292993631 | 0.661948573 | 1 | 1 | 46 | 157 | Over |
| map04726 | Serotonergic synapse | 0.292993631 | 0.661948573 | 1 | 1 | 46 | 157 | Over |
| map04728 | Dopaminergic synapse | 0.292993631 | 0.661948573 | 1 | 1 | 46 | 157 | Over |
| map04730 | Long-term depression | 0.292993631 | 0.661948573 | 1 | 1 | 46 | 157 | Over |
| map04912 | GnRH signaling pathway | 0.292993631 | 0.661948573 | 1 | 1 | 46 | 157 | Over |
| map04921 | Oxytocin signaling pathway | 0.292993631 | 0.661948573 | 1 | 1 | 46 | 157 | Over |
| map04925 | Aldosterone synthesis and secretion | 0.292993631 | 0.661948573 | 1 | 1 | 46 | 157 | Over |
| map04931 | Insulin resistance | 0.292993631 | 0.661948573 | 1 | 1 | 46 | 157 | Over |
| map04979 | Cholesterol metabolism | 0.292993631 | 0.661948573 | 1 | 1 | 46 | 157 | Over |
| map07011 | Penicillins | 0.292993631 | 0.661948573 | 1 | 1 | 46 | 157 | Over |
| map07012 | Cephalosporins - parenteral agents | 0.292993631 | 0.661948573 | 1 | 1 | 46 | 157 | Over |
| map07216 | Catecholamine transferase inhibitors | 0.292993631 | 0.661948573 | 1 | 1 | 46 | 157 | Over |
| map00270 | Cysteine and methionine metabolism | 0.359531411 | 0.783264859 | 3 | 6 | 46 | 157 | Over |
| map01064 | Biosynthesis of alkaloids derived from ornithine, lysine and nicotinic acid | 0.359531411 | 0.783264859 | 3 | 6 | 46 | 157 | Over |
| map00010 | Glycolysis / Gluconeogenesis | 0.501469868 | 0.784350306 | 1 | 2 | 46 | 157 | Over |
| map00053 | Ascorbate and aldarate metabolism | 0.501469868 | 0.784350306 | 1 | 2 | 46 | 157 | Over |
| map00290 | Valine, leucine and isoleucine biosynthesis | 0.501469868 | 0.784350306 | 1 | 2 | 46 | 157 | Over |
| map00311 | Penicillin and cephalosporin biosynthesis | 0.501469868 | 0.784350306 | 1 | 2 | 46 | 157 | Over |
| map00332 | Carbapenem biosynthesis | 0.501469868 | 0.784350306 | 1 | 2 | 46 | 157 | Over |
| map00401 | Novobiocin biosynthesis | 0.501469868 | 0.784350306 | 1 | 2 | 46 | 157 | Over |
| map00440 | Phosphonate and phosphinate metabolism | 0.501469868 | 0.784350306 | 1 | 2 | 46 | 157 | Over |
| map00471 | D-Glutamine and D-glutamate metabolism | 0.501469868 | 0.784350306 | 1 | 2 | 46 | 157 | Over |
| map00592 | alpha-Linolenic acid metabolism | 0.501469868 | 0.784350306 | 1 | 2 | 46 | 157 | Over |
| map00621 | Dioxin degradation | 0.501469868 | 0.784350306 | 1 | 2 | 46 | 157 | Over |
| map00730 | Thiamine metabolism | 0.501469868 | 0.784350306 | 1 | 2 | 46 | 157 | Over |
| map00750 | Vitamin B6 metabolism | 0.501469868 | 0.784350306 | 1 | 2 | 46 | 157 | Over |
| map00770 | Pantothenate and CoA biosynthesis | 0.501469868 | 0.784350306 | 1 | 2 | 46 | 157 | Over |
| map00790 | Folate biosynthesis | 0.501469868 | 0.784350306 | 1 | 2 | 46 | 157 | Over |
| map00966 | Glucosinolate biosynthesis | 0.501469868 | 0.784350306 | 1 | 2 | 46 | 157 | Over |
| map04270 | Vascular smooth muscle contraction | 0.501469868 | 0.784350306 | 1 | 2 | 46 | 157 | Over |
| map04745 | Phototransduction - fly | 0.501469868 | 0.784350306 | 1 | 2 | 46 | 157 | Over |
| map04750 | Inflammatory mediator regulation of TRP channels | 0.501469868 | 0.784350306 | 1 | 2 | 46 | 157 | Over |
| map04913 | Ovarian steroidogenesis | 0.501469868 | 0.784350306 | 1 | 2 | 46 | 157 | Over |
| map04923 | Regulation of lipolysis in adipocytes | 0.501469868 | 0.784350306 | 1 | 2 | 46 | 157 | Over |
| map05230 | Central carbon metabolism in cancer | 0.501469868 | 0.784350306 | 1 | 2 | 46 | 157 | Over |
| map00020 | Citrate cycle (TCA cycle) | 0.581126086 | 0.886217281 | 2 | 4 | 46 | 157 | Over |
| map00680 | Methane metabolism | 0.581126086 | 0.886217281 | 2 | 4 | 46 | 157 | Over |
| map00220 | Arginine biosynthesis | 0.63040537 | 0.89429599 | 2 | 5 | 46 | 157 | Over |
| map00430 | Taurine and hypotaurine metabolism | 0.63040537 | 0.89429599 | 2 | 5 | 46 | 157 | Over |
| map00460 | Cyanoamino acid metabolism | 0.63040537 | 0.89429599 | 2 | 5 | 46 | 157 | Over |
| map00650 | Butanoate metabolism | 0.63040537 | 0.89429599 | 2 | 5 | 46 | 157 | Over |
| map01062 | Biosynthesis of terpenoids and steroids | 0.63040537 | 0.89429599 | 2 | 5 | 46 | 157 | Over |
| map04216 | Ferroptosis | 0.63040537 | 0.89429599 | 2 | 5 | 46 | 157 | Over |
| map00330 | Arginine and proline metabolism | 0.693165604 | 0.939624485 | 3 | 8 | 46 | 157 | Over |
| map00340 | Histidine metabolism | 0.693165604 | 0.939624485 | 3 | 8 | 46 | 157 | Over |
| map00360 | Phenylalanine metabolism | 0.693165604 | 0.939624485 | 3 | 8 | 46 | 157 | Over |
| map00240 | Pyrimidine metabolism | 0.721995681 | 0.940308803 | 3 | 9 | 46 | 157 | Over |
| map02010 | ABC transporters | 0.721995681 | 0.940308803 | 3 | 9 | 46 | 157 | Over |
| map01110 | Biosynthesis of secondary metabolites | 0.89208101 | 1 | 9 | 28 | 46 | 157 | Over |
| map01120 | Microbial metabolism in diverse environments | 0.956883593 | 1 | 10 | 32 | 46 | 157 | Over |
| map00121 | Secondary bile acid biosynthesis | 1 | 1 | 1 | 3 | 46 | 157 | Over |
| map00130 | Ubiquinone and other terpenoid-quinone biosynthesis | 1 | 1 | 1 | 3 | 46 | 157 | Over |
| map00300 | Lysine biosynthesis | 1 | 1 | 1 | 3 | 46 | 157 | Over |
| map00310 | Lysine degradation | 1 | 1 | 1 | 3 | 46 | 157 | Over |
| map00380 | Tryptophan metabolism | 1 | 1 | 1 | 3 | 46 | 157 | Over |
| map00400 | Phenylalanine, tyrosine and tryptophan biosynthesis | 1 | 1 | 1 | 3 | 46 | 157 | Over |
| map00591 | Linoleic acid metabolism | 1 | 1 | 1 | 3 | 46 | 157 | Over |
| map00720 | Carbon fixation pathways in prokaryotes | 1 | 1 | 1 | 3 | 46 | 157 | Over |
| map00860 | Porphyrin and chlorophyll metabolism | 1 | 1 | 1 | 3 | 46 | 157 | Over |
| map01502 | Vancomycin resistance | 1 | 1 | 1 | 3 | 46 | 157 | Over |
| map04664 | Fc epsilon RI signaling pathway | 1 | 1 | 1 | 3 | 46 | 157 | Over |
| map00260 | Glycine, serine and threonine metabolism | 1 | 1 | 2 | 6 | 46 | 157 | Over |
| map00630 | Glyoxylate and dicarboxylate metabolism | 1 | 1 | 2 | 6 | 46 | 157 | Over |
| map00760 | Nicotinate and nicotinamide metabolism | 1 | 1 | 2 | 6 | 46 | 157 | Over |
| map01065 | Biosynthesis of alkaloids derived from histidine and purine | 1 | 1 | 2 | 6 | 46 | 157 | Over |
| map01070 | Biosynthesis of plant hormones | 1 | 1 | 2 | 6 | 46 | 157 | Over |

| **Supplementary Table 19. The KEGG pathway enrichment of the differential metabolites in DI contents of SBM40_N vs. FM in negative mode （n=2）** | | | | | | | | |
| --- | --- | --- | --- | --- | --- | --- | --- | --- |
|
| MapID | MapTitle | Pvalue | AdjustedPv | x1 | x2 | n | N | EnrichDirect |
| map00943 | Isoflavonoid biosynthesis | 0.006943345 | 0.655389457 | 6 | 7 | 70 | 205 | Over |
| map00591 | Linoleic acid metabolism | 0.038687973 | 0.655389457 | 3 | 3 | 70 | 205 | Over |
| map04976 | Bile secretion | 0.09356001 | 0.655389457 | 6 | 10 | 70 | 205 | Over |
| map00590 | Arachidonic acid metabolism | 0.115494978 | 0.655389457 | 2 | 2 | 70 | 205 | Over |
| map00680 | Methane metabolism | 0.115494978 | 0.655389457 | 2 | 2 | 70 | 205 | Over |
| map04917 | Prolactin signaling pathway | 0.115494978 | 0.655389457 | 2 | 2 | 70 | 205 | Over |
| map04925 | Aldosterone synthesis and secretion | 0.115494978 | 0.655389457 | 2 | 2 | 70 | 205 | Over |
| map00950 | Isoquinoline alkaloid biosynthesis | 0.116255444 | 0.655389457 | 3 | 4 | 70 | 205 | Over |
| map00982 | Drug metabolism - cytochrome P450 | 0.116255444 | 0.655389457 | 3 | 4 | 70 | 205 | Over |
| map01062 | Biosynthesis of terpenoids and steroids | 0.116255444 | 0.655389457 | 3 | 4 | 70 | 205 | Over |
| map04913 | Ovarian steroidogenesis | 0.116255444 | 0.655389457 | 3 | 4 | 70 | 205 | Over |
| map00071 | Fatty acid degradation | 0.269108989 | 0.655389457 | 2 | 3 | 70 | 205 | Over |
| map00100 | Steroid biosynthesis | 0.269108989 | 0.655389457 | 2 | 3 | 70 | 205 | Over |
| map00633 | Nitrotoluene degradation | 0.269108989 | 0.655389457 | 2 | 3 | 70 | 205 | Over |
| map04216 | Ferroptosis | 0.269108989 | 0.655389457 | 2 | 3 | 70 | 205 | Over |
| map04723 | Retrograde endocannabinoid signaling | 0.269108989 | 0.655389457 | 2 | 3 | 70 | 205 | Over |
| map00140 | Steroid hormone biosynthesis | 0.314439597 | 0.655389457 | 5 | 10 | 70 | 205 | Over |
| map00940 | Phenylpropanoid biosynthesis | 0.340378925 | 0.655389457 | 3 | 5 | 70 | 205 | Over |
| map04750 | Inflammatory mediator regulation of TRP channels | 0.340378925 | 0.655389457 | 3 | 5 | 70 | 205 | Over |
| map05200 | Pathways in cancer | 0.340378925 | 0.655389457 | 3 | 5 | 70 | 205 | Over |
| map05215 | Prostate cancer | 0.340378925 | 0.655389457 | 3 | 5 | 70 | 205 | Over |
| map00020 | Citrate cycle (TCA cycle) | 0.341463415 | 0.655389457 | 1 | 1 | 70 | 205 | Over |
| map00062 | Fatty acid elongation | 0.341463415 | 0.655389457 | 1 | 1 | 70 | 205 | Over |
| map00130 | Ubiquinone and other terpenoid-quinone biosynthesis | 0.341463415 | 0.655389457 | 1 | 1 | 70 | 205 | Over |
| map00190 | Oxidative phosphorylation | 0.341463415 | 0.655389457 | 1 | 1 | 70 | 205 | Over |
| map00231 | Puromycin biosynthesis | 0.341463415 | 0.655389457 | 1 | 1 | 70 | 205 | Over |
| map00254 | Aflatoxin biosynthesis | 0.341463415 | 0.655389457 | 1 | 1 | 70 | 205 | Over |
| map00365 | Furfural degradation | 0.341463415 | 0.655389457 | 1 | 1 | 70 | 205 | Over |
| map00620 | Pyruvate metabolism | 0.341463415 | 0.655389457 | 1 | 1 | 70 | 205 | Over |
| map00640 | Propanoate metabolism | 0.341463415 | 0.655389457 | 1 | 1 | 70 | 205 | Over |
| map00720 | Carbon fixation pathways in prokaryotes | 0.341463415 | 0.655389457 | 1 | 1 | 70 | 205 | Over |
| map00902 | Monoterpenoid biosynthesis | 0.341463415 | 0.655389457 | 1 | 1 | 70 | 205 | Over |
| map00904 | Diterpenoid biosynthesis | 0.341463415 | 0.655389457 | 1 | 1 | 70 | 205 | Over |
| map00945 | Stilbenoid, diarylheptanoid and gingerol biosynthesis | 0.341463415 | 0.655389457 | 1 | 1 | 70 | 205 | Over |
| map01052 | Type I polyketide structures | 0.341463415 | 0.655389457 | 1 | 1 | 70 | 205 | Over |
| map01066 | Biosynthesis of alkaloids derived from terpenoid and polyketide | 0.341463415 | 0.655389457 | 1 | 1 | 70 | 205 | Over |
| map03320 | PPAR signaling pathway | 0.341463415 | 0.655389457 | 1 | 1 | 70 | 205 | Over |
| map04114 | Oocyte meiosis | 0.341463415 | 0.655389457 | 1 | 1 | 70 | 205 | Over |
| map04211 | Longevity regulating pathway | 0.341463415 | 0.655389457 | 1 | 1 | 70 | 205 | Over |
| map04217 | Necroptosis | 0.341463415 | 0.655389457 | 1 | 1 | 70 | 205 | Over |
| map04218 | Cellular senescence | 0.341463415 | 0.655389457 | 1 | 1 | 70 | 205 | Over |
| map04611 | Platelet activation | 0.341463415 | 0.655389457 | 1 | 1 | 70 | 205 | Over |
| map04666 | Fc gamma R-mediated phagocytosis | 0.341463415 | 0.655389457 | 1 | 1 | 70 | 205 | Over |
| map04730 | Long-term depression | 0.341463415 | 0.655389457 | 1 | 1 | 70 | 205 | Over |
| map04745 | Phototransduction - fly | 0.341463415 | 0.655389457 | 1 | 1 | 70 | 205 | Over |
| map04810 | Regulation of actin cytoskeleton | 0.341463415 | 0.655389457 | 1 | 1 | 70 | 205 | Over |
| map04911 | Insulin secretion | 0.341463415 | 0.655389457 | 1 | 1 | 70 | 205 | Over |
| map04912 | GnRH signaling pathway | 0.341463415 | 0.655389457 | 1 | 1 | 70 | 205 | Over |
| map04914 | Progesterone-mediated oocyte maturation | 0.341463415 | 0.655389457 | 1 | 1 | 70 | 205 | Over |
| map04921 | Oxytocin signaling pathway | 0.341463415 | 0.655389457 | 1 | 1 | 70 | 205 | Over |
| map04927 | Cortisol synthesis and secretion | 0.341463415 | 0.655389457 | 1 | 1 | 70 | 205 | Over |
| map04934 | Cushing's syndrome | 0.341463415 | 0.655389457 | 1 | 1 | 70 | 205 | Over |
| map04970 | Salivary secretion | 0.341463415 | 0.655389457 | 1 | 1 | 70 | 205 | Over |
| map04972 | Pancreatic secretion | 0.341463415 | 0.655389457 | 1 | 1 | 70 | 205 | Over |
| map05033 | Nicotine addiction | 0.341463415 | 0.655389457 | 1 | 1 | 70 | 205 | Over |
| map05224 | Breast cancer | 0.341463415 | 0.655389457 | 1 | 1 | 70 | 205 | Over |
| map05323 | Rheumatoid arthritis | 0.341463415 | 0.655389457 | 1 | 1 | 70 | 205 | Over |
| map07034 | Eicosanoids | 0.341463415 | 0.655389457 | 1 | 1 | 70 | 205 | Over |
| map07229 | Angiotensin receptor and endothelin receptor antagonists | 0.341463415 | 0.655389457 | 1 | 1 | 70 | 205 | Over |
| map01040 | Biosynthesis of unsaturated fatty acids | 0.449006497 | 0.809572321 | 4 | 8 | 70 | 205 | Over |
| map01061 | Biosynthesis of phenylpropanoids | 0.449006497 | 0.809572321 | 4 | 8 | 70 | 205 | Over |
| map01063 | Biosynthesis of alkaloids derived from shikimate pathway | 0.49443619 | 0.874144803 | 4 | 9 | 70 | 205 | Over |
| map00360 | Phenylalanine metabolism | 0.607159004 | 1 | 2 | 4 | 70 | 205 | Over |
| map04024 | cAMP signaling pathway | 0.607159004 | 1 | 2 | 4 | 70 | 205 | Over |
| map04726 | Serotonergic synapse | 0.607159004 | 1 | 2 | 4 | 70 | 205 | Over |
| map04080 | Neuroactive ligand-receptor interaction | 0.737815065 | 1 | 4 | 10 | 70 | 205 | Over |
| map01110 | Biosynthesis of secondary metabolites | 1 | 1 | 13 | 37 | 70 | 205 | Over |
| map00040 | Pentose and glucuronate interconversions | 1 | 1 | 1 | 2 | 70 | 205 | Over |
| map00052 | Galactose metabolism | 1 | 1 | 1 | 2 | 70 | 205 | Over |
| map00073 | Cutin, suberine and wax biosynthesis | 1 | 1 | 1 | 2 | 70 | 205 | Over |
| map00300 | Lysine biosynthesis | 1 | 1 | 1 | 2 | 70 | 205 | Over |
| map00330 | Arginine and proline metabolism | 1 | 1 | 3 | 8 | 70 | 205 | Over |
| map00564 | Glycerophospholipid metabolism | 1 | 1 | 1 | 2 | 70 | 205 | Over |
| map00630 | Glyoxylate and dicarboxylate metabolism | 1 | 1 | 1 | 2 | 70 | 205 | Over |
| map00750 | Vitamin B6 metabolism | 1 | 1 | 1 | 2 | 70 | 205 | Over |
| map00920 | Sulfur metabolism | 1 | 1 | 1 | 2 | 70 | 205 | Over |
| map00981 | Insect hormone biosynthesis | 1 | 1 | 1 | 2 | 70 | 205 | Over |
| map00983 | Drug metabolism - other enzymes | 1 | 1 | 1 | 2 | 70 | 205 | Over |
| map00984 | Steroid degradation | 1 | 1 | 1 | 2 | 70 | 205 | Over |
| map01212 | Fatty acid metabolism | 1 | 1 | 1 | 2 | 70 | 205 | Over |
| map01522 | Endocrine resistance | 1 | 1 | 1 | 2 | 70 | 205 | Over |
| map02060 | Phosphotransferase system (PTS) | 1 | 1 | 1 | 2 | 70 | 205 | Over |
| map04075 | Plant hormone signal transduction | 1 | 1 | 1 | 2 | 70 | 205 | Over |
| map04270 | Vascular smooth muscle contraction | 1 | 1 | 1 | 2 | 70 | 205 | Over |
| map04664 | Fc epsilon RI signaling pathway | 1 | 1 | 1 | 2 | 70 | 205 | Over |
| map04725 | Cholinergic synapse | 1 | 1 | 1 | 2 | 70 | 205 | Over |
| map04971 | Gastric acid secretion | 1 | 1 | 1 | 2 | 70 | 205 | Over |
| map07225 | Glucocorticoid and mineralocorticoid receptor agonists/antagonists | 1 | 1 | 1 | 2 | 70 | 205 | Over |
| map07226 | Progesterone, androgen and estrogen receptor agonists/antagonists | 1 | 1 | 1 | 2 | 70 | 205 | Over |

**Supplementary Table 20. The 10 most influential metabolites identified as the potential biomarkers in DI contents of SBM20_N vs. FM in positive mode (n = 12)**

| Metabolite | Retention time (min) | Measured Mass (Da) | Calculated mass (Da) | Elemental composition | Scan mode | Log2FC | VIP |
| --- | --- | --- | --- | --- | --- | --- | --- |
| 5-methylbenzimidazole^a^ | 7.110 | 132.06830 | 132.06875 | C_8_H_8_N_2_ | + | 5.129712976 | 5.315462186 |
| Glycitin^a,b^ | 8.397 | 446.12096 | 446.12123 | C_22_H_22_O_10_ | + | 4.38287613 | 4.537198885 |
| Byakangelicol^a^ | 6.999 | 316.09427 | 316.09469 | C_17_ H_16_O_6_ | + | 4.292366261 | 4.68168822 |
| Genistin^b^ | 8.829 | 432.10525 | 432.10438 | C_21_H_20_ O_10_ | + | 3.955143065 | 4.408600168 |
| Malonylglycitin^a^ | 9.355 | 532.12162 | 532.12169 | C_25_H_24_O_13_ | + | 3.909536595 | 4.164903052 |
| Genistein 4'-O-glucuronide^b^ | 8.918 | 446.08458 | 446.08491 | C_21_H_18_O_11_ | + | 3.79367654 | 4.486938629 |
| Glycitein^b^ | 9.738 | 284.06800 | 284.06847 | C_16_H_12_O_5_ | + | 3.777010085 | 4.172300022 |
| amfonelic acid^a^ | 8.825 | 308.11558 | 308.11609 | C_18_H_16_N_23_ | + | -3.748451505 | 4.220201888 |
| 3,4-dihydroxyphenylacetic acid^a^ | 11.785 | 168.04218 | 168.04225 | C_8_H_8_O_4_ | + | 3.617849766 | 4.050711024 |
| Daidzein^a,b^ | 8.221 | 254.05760 | 254.05790 | C_15_H_10_O_4_ | + | 3.583147386 | 4.390419608 |

Note: ^a^ The metabolite was confirmed by comparison to the metabolites of the ChemSpider Database. ^b^ The metabolite was identified by comparison to the metabolites of the mzCloud Database. Note: FM, fish meal control group; SBM20_N, 20% soybean meal protein replacement level to FM diet.

**Supplementary Table 21. The 10 most influential metabolites identified as the potential biomarkers in DI contents of SBM40_N vs. FM in positive mode (n = 12)**

| Metabolite | Retention time (min) | Measured Mass (Da) | Calculated mass (Da) | Elemental composition | Scan mode | Log2FC | VIP |
| --- | --- | --- | --- | --- | --- | --- | --- |
| amfonelic acid^a^ | 8.825 | 308.11558 | 308.11609 | C18H16N2O3 | + | -5.515474982 | 4.051252209 |
| Genistin^a,b^ | 8.829 | 432.10525 | 432.10438 | C21H20O10 | + | 5.018772047 | 3.592423962 |
| Glycitin^a,b^ | 8.397 | 446.12096 | 446.12130 | C22H22O10 | + | 4.966728245 | 3.66802547 |
| 3-Methoxyflavone^a,b^ | 13.674 | 274.05944 | 252.07864 | C16H12O3 | + | -4.538611574 | 3.728456138 |
| Daidzin^a,b^ | 8.225 | 416.11026 | 416.11073 | C21H20O9 | + | 4.482810579 | 3.174317493 |
| 5-methylbenzimidazole^a,b^ | 7.114 | 132.0683 | 132.06875 | C8H8N2 | + | 4.456220089 | 3.128085638 |
| Glycitein^a,b^ | 9.738 | 284.068 | 284.06847 | C16H12O5 | + | 4.179668333 | 3.232877287 |
| Malonylglycitin^a,b^ | 9.355 | 532.12162 | 532.12169 | C25H24O13 | + | 4.061989013 | 3.058492432 |
| Genistein 4'-O-glucuronide^a,b^ | 8.918 | 446.08458 | 446.08491 | C21H18O11 | + | 4.025281755 | 3.385268352 |
| Genistein^a,b^ | 8.908 | 270.05249 | 270.05282 | C15H10O5 | + | 3.967463991 | 3.282617249 |

Note: ^a^ The metabolite was confirmed by comparison to the metabolites of the ChemSpider Database. ^b^ The metabolite was identified by comparison to the metabolites of the mzCloud Database. FM, fish meal control group; SBM40_N, 40% soybean meal protein replacement level to FM diet.

**Supplementary Table 22.** **The 10 most influential metabolites identified as the potential biomarkers of SBM20_N vs. FM in negative mode (n = 12)**

| Metabolite | Retention time (min) | Measured Mass (Da) | Calculated mass (Da) | Elemental composition | Scan mode | Log2FC | VIP |
| --- | --- | --- | --- | --- | --- | --- | --- |
| FMNH_2_^a^ | 9.859 | 458.12116 | 458.12027 | C_17_H_23_N_4_O_9_P | ﹣ | 4.881521947 | 5.083650443 |
| Glycitein^b^ | 8.701 | 284.06819 | 284.06847 | C_16_H_12_O_5_ | ﹣ | 4.142369284 | 4.441020929 |
| Soyasaponin I^a,b^ | 12.309 | 942.52271 | 942.51882 | C_48_H_78_O_18_ | ﹣ | 3.619941537 | 3.885453181 |
| olmelin^a^ | 7.425 | 284.06825 | 284.06847 | C_16_H_12_O_5_ | ﹣ | 3.598936557 | 3.678394927 |
| Genistein^a,b^ | 7.707 | 270.05251 | 270.05282 | C_15_H_10_O_5_ | ﹣ | 3.559529422 | 3.959916397 |
| Ginsenoside Ro^a^ | 12.418 | 956.50175 | 956.49808 | C_48_H_76_O_19_ | ﹣ | 3.552409138 | 3.823873851 |
| 4-Phenolsulfonic acid^a,b^ | 3.721 | 173.99861 | 173.99868 | C_6_H_6_O4_S_ | ﹣ | 3.357909949 | 3.751644755 |
| Daidzein^a,b^ | 8.563 | 254.05767 | 254.05791 | C_15_H_10_O_4_ | ﹣ | 3.338270717 | 3.499205027 |
| Baicalin^a^ | 8.217 | 446.08484 | 446.08491 | C_21_H_18_O_11_ | ﹣ | 3.2131398 | 3.244455336 |
| Glycitin^a^ | 8.433 | 446.12099 | 446.12130 | C_22_H_22_O_10_ | ﹣ | 3.078329785 | 2.919911448 |

Note: same as Supplementary Table 20.

**Supplementary Table 23.** **The 10 most influential metabolites identified as the potential biomarkers of SBM40_N vs. FM in negative mode (n = 12)**

| Metabolite | Retention time (min) | Measured Mass (Da) | Calculated mass (Da) | Elemental composition | Scan mode | Log2FC | VIP |
| --- | --- | --- | --- | --- | --- | --- | --- |
| FMNH2^a^ | 9.859 | 458.12116 | 458.12027 | C17 H23 N4 O9 P | ﹣ | 3.839018875 | 3.073585751 |
| Soyasaponin I ^a,b^ | 12.309 | 942.52271 | 942.5188157 | C48 H78 O18 | ﹣ | 3.679278099 | 2.953122401 |
| Ginsenoside Ro ^a^ | 12.418 | 956.50175 | 956.4980802 | C48 H76 O19 | ﹣ | 3.662107746 | 2.898653817 |
| 4-Phenolsulfonic acid^a,b^ | 3.721 | 173.99861 | 173.9986797 | C6 H6 O4 S | ﹣ | 3.636516758 | 2.53933941 |
| Glycitein ^b^ | 8.701 | 284.06819 | 284.0684735 | C16 H12 O5 | ﹣ | 3.580617861 | 2.982005477 |
| Genistein ^a,b^ | 7.707 | 270.05251 | 270.0528234 | C15 H10 O5 | ﹣ | 3.386606428 | 2.937547132 |
| Cys-tyr ^a^ | 8.583 | 284.08299 | 284.083078 | C12 H16 N2 O4 S | ﹣ | -3.263296814 | 2.153790432 |
| Indole-3-butyric acid ^a^ | 8.889 | 203.09459 | 203.0946287 | C12 H13 N O2 | ﹣ | -3.263182025 | 2.1873558 |
| Glycitin ^a^ | 8.433 | 446.12099 | 446.1212969 | C22 H22 O10 | ﹣ | 3.165457964 | 2.231287951 |
| olmelin ^a^ | 7.425 | 284.06825 | 284.0684735 | C16 H12 O5 | ﹣ | 3.145914566 | 2.460171178 |

Note: same as Supplementary Table 21.

**Supplementary Table 24. Intensities of metabolites identified as the potential biomarkers in DI contents of SBM20_N vs. FM in positive mode (n = 12)**

| Biomarker | FM | SBM20_N |
| --- | --- | --- |
| 5-methylbenzimidazole | 11691.36±3978.87^a^ | 409319.65±29383.60^b^ |
| Glycitin | 14214.56±2806.90^a^ | 296558.68±37212.86^b^ |
| Byakangelicol | 19782.31±2754.54^a^ | 387621.60±61469.44^b^ |
| Genistin | 19332.62±2253.35^a^ | 299852.3±29083.10^b^ |
| Malonylglycitin | 33055.54±5972.64^a^ | 496743.4±69637.05^b^ |
| Genistein 4'-O-glucuronide | 417717.7±54172.23^a^ | 5792864.00±343256.67^b^ |
| Glycitein | 35776.77±6769.80a | 490449.62±55914.87^b^ |
| amfonelic acid | 1093944.37±133559.70^a^ | 81395.22±16899.95^b^ |
| 3,4-dihydroxyphenylacetic acid | 81395.22±6255.79^a^ | 424795.71±67915.34^b^ |
| Daidzein | 177591.10±40117.01^a^ | 2128413.49±125899.10^b^ |

Note: FM, fish meal control group; SBM20_N, 20% soybean meal protein replacement level to FM diet. Values in the same row with different superscripts are significantly different (*P* < 0.05).

**Supplementary Table 25. Intensities of metabolites identified as the potential biomarkers in DI contents of SBM20_N vs. FM in negative mode (n = 12)**

| Biomarker | FM | SBM20_N |
| --- | --- | --- |
| FMNH2 | 9721.54±798.91^a^ | 286562.70±26442.98^b^ |
| Glycitein | 44046.61±4587.75^a^ | 777839.30±78241.61^b^ |
| Soyasaponin I | 3087021.98±307856.00^a^ | 37953389.40±5926490.67^b^ |
| olmelin | 3502.78±473.40^a^ | 42442.42±10279.30^b^ |
| Genistein | 59153.21±5299.56^a^ | 697434.60±101261.20^b^ |
| Ginsenoside Ro | 6207.55±522.03^a^ | 72828.62±7085.61^b^ |
| 4-Phenolsulfonic acid | 283920.96±36412.50^a^ | 4911046.37±1066969.32^b^ |
[truncated: 140,860 more chars]
